# “To speak or not to speak”: A qualitative analysis on the attitude and willingness of women to start conversations about voluntary medical male circumcision with their partners in a peri-urban area, South Africa

**DOI:** 10.1371/journal.pone.0210480

**Published:** 2019-01-25

**Authors:** Candice M. Chetty-Makkan, Jonathan M. Grund, Reuben Munyai, Vuyokazi Gadla, Violet Chihota, Mpho Maraisane, Salome Charalambous

**Affiliations:** 1 The Aurum Institute, Parktown, South Africa; 2 Division of Global HIV & TB, Centers for Disease Control and Prevention, Pretoria, South Africa; 3 School of Public Health, Faculty of Health Sciences, University of the Witwatersrand, Johannesburg, South Africa; University of California Los Angeles, UNITED STATES

## Abstract

**Introduction:**

Voluntary medical male circumcision (VMMC) reduces the risk of HIV infection in heterosexual men and has long-term indirect protection for women, yet VMMC uptake in South Africa remains low (49.8%) in men (25–49 years). We explored the attitude and willingness of women to start conversations on VMMC with their sexual partners in a South African peri-urban setting to increase VMMC uptake.

**Methods:**

Thirty women with median age of 30 years (inter-quartile range 26–33 years) were interviewed in a language of their choice. Key questions included: types of approach to use, gender roles, benefits and barriers to introducing the topic of VMMC, and perceptions of VMMC. Interviews were digitally-recorded, transcribed, and translated. Through a standard iterative process, a codebook was developed (QSR NVIVO 10 software) and inductive thematic analysis applied.

**Results:**

Most women were willing talk to their sexual partners about circumcision, but indicated that the decision to circumcise remained that of their sexual partner. Women felt that they should encourage their partners, show more interest in circumcision, be patient, speak in a caring and respectful tone, choose a correct time when their partner was relaxed and talk in a private space about VMMC. Using magazine/newspaper articles, pamphlets or advertisements were identified as tools that could aid their discussion. Substantial barriers to initiating conversations on VMMC included accusations by partner on infidelity, fear of gender-based violence, cultural restrictions and hesitation to speak to a mature partner about circumcision.

**Conclusions:**

Women need to ensure that before talking to their partner about circumcision, the environment and approach that they use are conducive. Female social network forums could be used to educate women on conversation techniques, skills to use when talking to their partners and how to address communication challenges about circumcision. Involvement of women in VMMC awareness campaigns could encourage circumcision uptake among men.

## Introduction

In 2009, the voluntary medical male circumcision (VMMC) for HIV prevention programme began in South Africa, a country with high Human Immunodeficiency Virus (HIV) prevalence and low VMMC coverage [1,2]. VMMC reduces the risk of HIV infection in heterosexual men [3–6]. The 2012 South African Human Science Research Council survey showed that across all nine provinces, uptake of circumcision in men aged 25–49 years was low (49.8%) when compared to those aged between 15–24 years (80.2%) [7]. The prevalence of HIV in South Africa is high among men aged 25–49 years (25.7%) [7] and women aged 20–34 years old (31.6%) [7]. Although VMMC could offer long-term indirect protection for women [8], VMMC uptake among mature adult men in sub-Saharan Africa remains low. Some challenges were reduced acceptability of the procedure, poor communication regarding the benefits of VMMC and concerns about circumcising at a mature age [7–11]. Data on the total distribution of number of circumcisions conducted between 2011 and 2016 from a routine VMMC service delivery programme in South Africa showed lower number of circumcisions among men aged ≥25 years: 32.9% (13,154/40,038) compared to those ≤24 years 67.1% (26,884/40,038) (internal program data from the Aurum Institute). Since the uptake of VMMC is low among men ≥25 years, exploring female attitudes toward circumcision of their male partners could contribute to improving current HIV prevention packages [12–14].

Qualitative and quantitative studies have described women as being supportive of their partners’ decision to circumcise [13,15–19]. One study in Kenya and another in Tanzania showed that women who were in stable relationships were able to discuss and influence their partner’s decision to circumcise [18,19]. While most studies explored the perceptions that women had regarding the relationship between HIV risk and VMMC and how this could influence sexual decision making, there are few that assessed how women could start a conversation on circumcision with their sexual partners [13–15,17,20–22].

There is a need to explore how women could introduce VMMC to their partners [16,23,24]. Communication about VMMC between couples encourages mutual decision making on VMMC [14,17–19,25]. However, in certain cultures that practice traditional circumcision, like in South Africa, women or uncircumcised men are not allowed to discuss the rituals of male circumcision [26]. Women may in general have limited knowledge of circumcision which possibly prevents them from speaking to their male partners about this issue [13,25]. Some challenges that women experienced in past studies included poor communication with their partners about VMMC and limited access to information on VMMC, especially in communities where VMMC is not part of the culture [13,16,19,25]. One Kenyan study that explored the role of women in VMMC reported that the majority of women discussed the positive and negative aspects of VMMC with their partners before they were circumcised [19]. Yet, the type of approach that these women used to start a conversation on VMMC with their partners were not described.

This sub-study took place as part of a larger implementation science research study (Imbizo study) that aimed to increase the proportion of men aged 25–49 years who accessed VMMC services [27]. Here, we explore the attitude and willingness of women to start a conversation on medical male circumcision with their sexual partners.

## Methods

**Fig 1:**
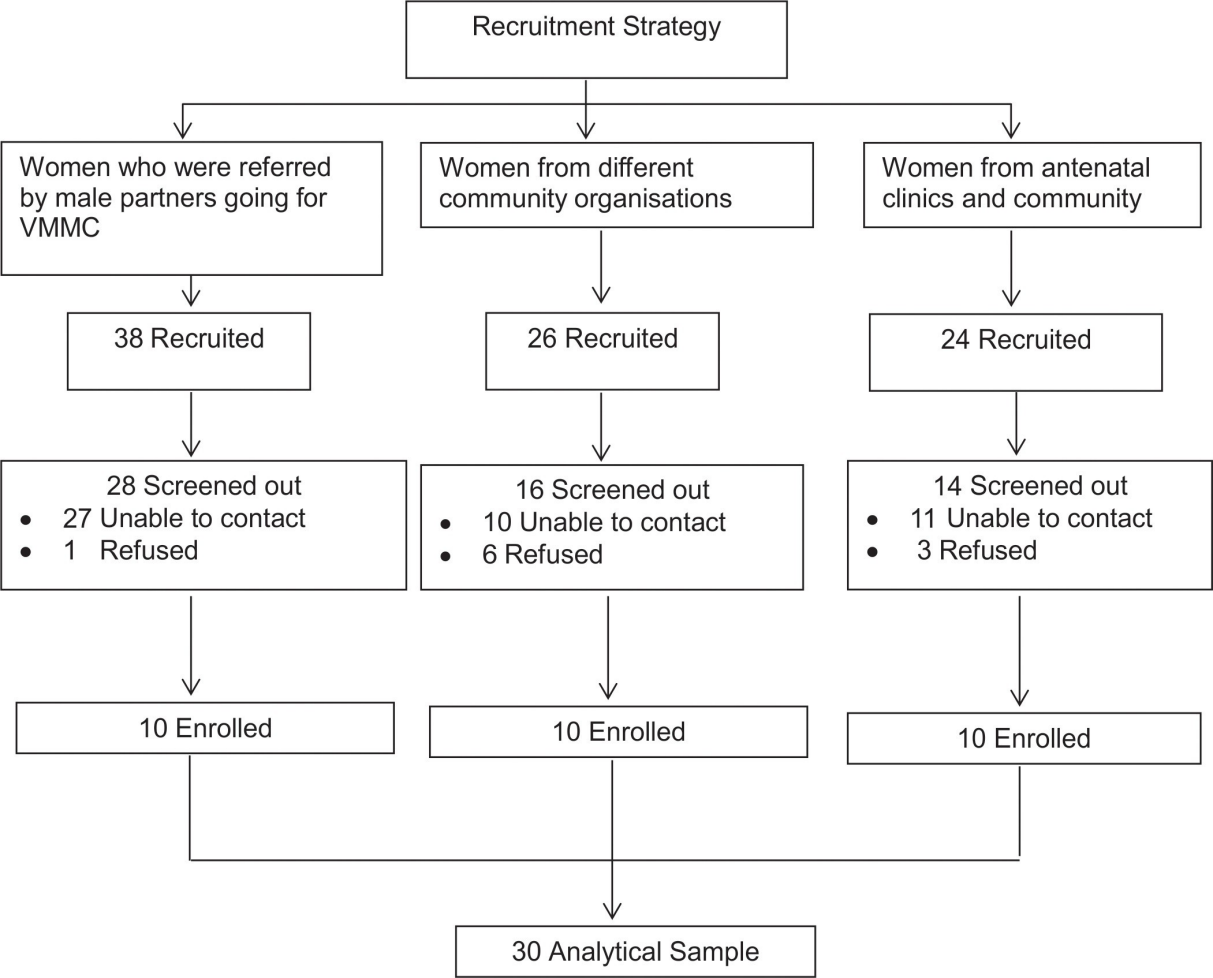
Flow of study procedures of the enrolled sample from 1 April 2014 to 31 October 2014.

This sub-study took place from April to October 2014 at a peri-urban site, Ekurhuleni North, Gauteng province in South Africa. We used three approaches to recruit women purposively into the study in order to ensure diverse participation. First, contact information and permission to invite female partners for an interview were obtained from circumcised men enrolled in the Imbizo main study. Second, a list of non-governmental organizations that focused on women within the community was obtained and appointments arranged with those who agreed to be interviewed. Finally, female patients from antenatal clinics and those from the surrounding community were individually invited to participate. Contact information, including names and phone numbers, were recorded for all interested participants and used to set up appointments. Fig 1 describes the flow of study procedures for the enrolled sample from 1 April 2014 to 31 October 2014.

Appointments were scheduled with women who were interested in the study and who agreed to participate in the interview at a time that was convenient for them. Participants were eligible if they were aged ≥18 years, agreed to be interviewed with digital recording of the session, and able to communicate in one of the study languages. All participants provided written informed consent prior to enrolment. Illiterate participants provided a thumbprint to acknowledge understanding in the presence of a witness. Participants were only interviewed once. Participants consented to their direct quotations being published.

A total of 30 women were enrolled, with a median age of 30 years (interquartile range 26–33 years). Since the 30 interviews started to reveal similar information, this was an indication of saturation being reached. The interviews lasted approximately two hours each. Most women preferred the interviews to be conducted in the local language of Sepedi (46.7%) or isiZulu (36.7%), with a minority choosing English (16.7%) which were the most common languages in the study area. Fifteen (50%) out of the 30 women voluntarily disclosed their birthplace during their interview–the majority (66.7%) were from the peri-urban area where the study was conducted while five (33.3%) were from rural settings of other provinces but currently lived in the peri-urban area. A total of 20 (66.7%) women disclosed that their partners were circumcised while only 3 (10%) indicated that their partners were not circumcised. Seven women (23.3%) did not disclose the circumcision status of their partner.

### Ethical considerations

Approval was obtained from the University of the Witwatersrand Human Research Ethics Committee (M130711) and the Centers for Disease Control and Prevention Institutional Review Board (6546).

### Data collection

All participants who signed the informed consent form, also agreed verbally for the session to be digitally recorded. An interview guide was used to explore the role of women in introducing VMMC (S1 Table.) that included questions on: the types of approach to use when introducing the topic of VMMC; gender roles; benefits and barriers when speaking about VMMC; understanding of and perception of VMMC. The trained interviewers were employed by the Aurum Institute. Interviewers were trained on qualitative data collection by an experienced co-investigator. Male and female interviewers had professional relationships with participants and their interaction ended after data collection was completed. Interviewers maintained an objective attitude during data collection and this was monitored during debrief sessions. Only the interviewer and the participant were present during the session. Interviewers completed process notes after each interview that detailed the progression of the session and the demeanour of the participant during the interview. All participants who completed the interview were reimbursed $10.64 (R100.00) to compensate them for their time and travel.

### Data analysis

In-depth interviews were transcribed verbatim, and translated into English where applicable. Most of the interviews that were conducted in a local language were outsourced to a registered, accredited translation company. The translation company that is based in South Africa transcribed the digital recordings to the local language and then translated these into English. The quality team at the Aurum Institute checked the transcriptions. All transcripts were available in English before the coding and analysis took place. Digital recordings and process notes were reviewed during the analysis. QSR NVIVO 10 was used for the analysis of in-depth interviews [28]. A grounded theory approach was used as the theoretical framework [29,30]. Thematic analysis was used to highlight the emerging inductive themes. An iterative approach was used to develop the codebook and index the transcripts from which categories, themes and patterns emerged from the data [29]. A co-investigator with a doctorate level qualification developed the coding scheme using the English transcripts. For reliability, the original transcript and codes were sent to two independent reviewers. The independent reviewers were part of the investigator team with medical, masters and doctorate level qualifications. The reviewers categorised the original transcript according to the codebook and suggested new codes as needed [31]. Codes where both reviewers agreed to a particular code were retained while those that lacked intercoder reliability were dropped from the analysis [30]. The codebook was revised and the themes finalised which are displayed as direct quotes [32]. Participants did not provide feedback on the findings.

## Results

### Main theme: Willingness of women to start a conversation on VMMC

Most women were willing to start a conversation on VMMC with their sexual partners. Substantial sub-themes that emerged were the approach that women should use and challenges to starting a conversation on VMMC.

#### Sub-theme: Approach that women should use when starting a conversation on VMMC

Most women felt that they should encourage their partners, show more interest in circumcision, be patient, speak in a caring and respectful tone, choose a correct time when their partner was relaxed and talk in a private space about VMMC. This seemed to be a salient theme across all categories of women, but women from the community antenatal clinics emphasised the importance of using an acceptable approach when raising the topic of circumcision with partners. One participant described how timing and the approach to discussing VMMC with her partner was crucial:

*“…They [Women] need to know the good approach… woman need to be patient with their partners*, *and avoid harassing him and give him time and chance and keep on emphasizing that it’s for his health… Don’t talk about it being angry*, *don’t talk about it when he is not happy*, *don’t just say you were with your friends and your friend told you… You must target the days he is so happy*. *Talk about it when you are relaxed [and] happy*, *talk about it after food maybe when you are in the bedroom*. *Say it nicely”*.—Aged 42 years, recruited from the community antenatal clinic.

Although women were willing to raise the topic of VMMC with their partners, they still felt that circumcision remained ultimately the decision of the man.

*“…because I use[d] to tell him now and again*, *maybe it took him [partner] a year to decide to do it*, *and I was telling him that you need to do this*. *… I was telling him [partner] about things like that and he was also doing his research as well*. *And he ended up deciding to do it*.*”–*Aged 35 years, recruited from community organisation

Providing detailed information on the benefits of VMMC to their partners by using magazine/newspaper articles, pamphlets or advertisements were further identified as tools that women could use to aid their discussion with their partners. In general, women did not provide specific detail of what should be included in the material, instead they felt that their partners should be exposed to circumcision material. Most women emphasised the importance of using multiple opportunities to direct their partner’s attention to available VMMC information. One participant described in detail the type of materials that could be used when introducing VMMC with her partner:

*“Since there’s a lot out there about circumcision*, *it’s even advertised in the roads*, *so you can start in that way… women have to show more interest in these things about circumcision*. *She can use photos if she chooses… These pictures can be hung on the walls in the house*, *and saved on her phone… She could have CDs or something that she can play or watch in his presence*. *She could also surf the internet on the topic… This will attract her partner’s attention*.*”* Aged 24 years, recruited from the community antenatal clinic.

A soft theme that emerged was knowledge that women had on the benefits of the VMMC procedure. Almost all women felt that VMMC was a safe procedure, promoted good hygiene and reduced sexually transmitted infections or HIV. In addition, more than half of the women felt that the services provided at the clinic were free, safe and better when compared to traditional circumcision. Only five women were also aware of the benefits of VMMC to their personal health such as prevention of cervical cancer. One participant described her knowledge regarding the health benefits of the VMMC procedure:

*“…The way I understand*, *circumcision reduces many diseases*. *And in women*, *we are often told that it reduces cervical cancer… Men may be infected by the gonorrhoea AIDS*, *and the STDs*. *Any disease that a man may be infected with easily… …”—*Aged 32 years, recruited as partner of a circumcised man

Another participant described how she felt that the counselling, health screening and follow-up visits during the VMMC procedure was beneficial:

*“…*, *it's [circumcision] much better than at the mountains [traditional circumcision] because you get counselling before and you also tested for diseases that you might have*, *like high blood and HIV and they also they you CD4 count to see whether it's fine for you to be circumcised or not*. *After circumcision you go for check-up to see how you are recovering*, *whether you recovering nicely or if there are infections*.*”*—Aged 31 years, recruited from community organisation.

Although most women were willing to speak to their partners about VMMC, there were also dominant challenges to talking about circumcision that emerged as a concern.

#### Sub-theme: Barriers to women starting a conversation on VMMC with their partners

Substantial barriers to initiating conversations on VMMC included accusations by partner on infidelity, fear of gender-based violence, cultural restrictions and hesitation to speak to a mature partner about circumcision. One participant described her fear as follows:

*“To me it [circumcision] sounded good because it’s what I wanted him to do*, *but was scared to tell him”*—Aged 27 years, recruited as partner of a circumcised man

Some women, especially those who were partners of a circumcised man felt that if they introduced circumcision to their partner, he may have suspected infidelity. Two participants described the fear of being suspected of infidelity as follows:

*“If you ask him about circumcision*, *he will ask you which circumcised man have you engaged in sexual intercourse with*. *Where did you see him*?*”*—Aged 32 years, recruited as partner of a circumcised man:

*“…some man will say*: *so you slept with a man who is circumcised*, *because you are saying there is difference between a man who is circumcised and a man who is not circumcised… And what is your experience or did you sleep with someone who got circumcised…”*—Aged 42 years, recruited from the community antenatal clinic:

Other participants were concerned about gender based violence if they started a conversation on circumcision with their partners. One participant described her fear as follows:

*“…Another one [partner] will just beat you up the moment you start talking about it [circumcision]…”—*Aged 31 years, recruited from community organisation

Women also seemed afraid to talk to their partners about circumcision due to cultural restrictions. Some cultures do not believe in circumcision while others that practice traditional circumcision consider it to be a secretive process that is applicable to men only. Concerns of breaking traditional expectations were predominantly expressed by women from antennal clinics and those from community organisations. One participant described how cultural restrictions could prevent females from speaking to their partners about VMMC:

*“… Some Zulu men tell us that we don't do that [circumcision]*. *And then we went to others*: *That [circumcision] is none of your business*, *you shouldn't be telling me about circumcision*. *You are not a man you know nothing about circumcision*! *So there's nothing I will say to you!'*- Aged 31 years old, recruited from community organisation

Another theme that emerged was hesitation to speak to male partners who were of a mature age. This sub-theme was salient among women who were recruited from the community antenatal clinics. Some women felt that men of a mature age would be embarrassed to access circumcision services, which is described as follows:

*“… when you go when you are old*, *it was an embarrassment… yes it was an embarrassment that you [are] circumcising with kids…”*—Aged 29 years, recruited from the community antenatal clinic

## Discussion

In South Africa, women need to ensure that before talking to their male partner’s about circumcision, the environment and approach that they use are conducive. Although the male partner makes the ultimate decision, some viable options that women could use when talking to their partners about circumcision could include a gentle, respectful approach and general media circumcision resources.

Women may have the ability to influence their partners to circumcise depending on the type of approach that they use. Other studies from Africa confirmed that women were likely to influence the uptake of VMMC among mature men [16,17,19,20]. Focus group discussions that were conducted in Tanzania, described women as being a source of information to their partners, and they also felt that the approach to be used needs to be indirect and careful using “soft” language [22]. Although previous studies reported that women supported their partner’s decision to circumcise which influenced decision-making around VMMC, few described in detail the approach and behaviour that women could use when introducing the topic of VMMC [13,15–18,33]. Women felt that they could influence men to circumcise by using multiple resources and opportunities to create awareness on circumcision in a respectful manner instead of being forceful. Women from our study felt that if men were respected and did not feel demoralised, they would be more willing to listen to options around VMMC. However, contrary to other studies in Tanzania and Kenya, women in our study did not report direct insistence on partners being circumcised as the approach to follow [14,20].

A novel finding from our study was the importance of identifying the right location and ideal opportunity to speak to men about circumcision. When a suitable place and time is identified, media resources could be used to aid the discussion on circumcision. This platform of providing detailed information on VMMC could allow male partner’s the time and space needed to reach an independent decision on circumcision. Other studies confirmed the advantage of using media advertising and a pleasant approach when talking about circumcision [18,22]; while our study showed that creating the right environment is also important when talking about this sensitive issue.

Communication on circumcision needs to include issues that are important to men and women. A study conducted in Zambia that evaluated attitudes, knowledge and preferences about VMMC among men and their partners highlighted that joint discussions between the couple had a positive impact on men’s reported readiness to be circumcised[17]. The gender role power dynamics could influence how women talk to men about circumcision. Depending on the type of relationship a woman shares with her partner, using the appropriate approach, choosing the right environment and time could be vital when talking about circumcision.

Our study showed that women felt at risk of being accused of infidelity and subject to physical abuse if they raised or introduced the topic of circumcision with their male partners. This finding was different to past research which showed that accusation of infidelity primarily took place during VMMC’s post-operative period which led to gender based violence [8,33]. In certain socio-cultural contexts, women are excluded from discussions around VMMC [13], therefore male partners could feel that traditional expectations are challenged if informed by a female about circumcision.

Hesitation to speak to mature partners about circumcision was another challenge, as circumcision is generally associated with younger boys. Our findings were confirmed by a qualitative study that was conducted in Tanzania with 142 participants where it was reported that adult men were ashamed of accessing clinic services with younger boys as they were married and had children of their own [34]. Based on our findings we piloted an exclusive adult clinic to provide an environment where mature men could feel comfortable to access VMMC services. Results of this intervention are reported in full elsewhere [27]. Women could encounter communication challenges and resistance when speaking to mature men about circumcision, thus learning constructive skills could assist them in creating awareness on VMMC.

Limitations of the study were that women could have provided responses that were socially desirable and acceptable even if it was contradictory to their true beliefs, as some were referred to the study by their male partners. Another limitation is that most women who were interviewed in the study had partners who were circumcised which could limit generalizability of the results. However, in order to obtain comprehensive information and to explore this topic limiting biases, we recruited three categories of women (i) partners of men who were circumcised (ii) women accessing antenatal services in the community (iii) women involved in community organisations. These women had varied backgrounds and different experiences with understanding circumcision practices, which helped us gain a detailed perspective on their attitudes toward circumcision. By reassuring the participants of confidentiality, building trust and probing using different techniques, we attempted to mitigate some of the limitations. Some of the study strengths included how creating the right environment and using appropriate communication techniques could assist women to promote VMMC awareness with their partners.

## Conclusion

Women were willing to speak to their partners about VMMC and felt that the manner in which they spoke and interacted with their sexual partners could encourage them to consider circumcision. Educating women on conversation techniques, types of approach and behaviour could assist them when communicating with their partner on VMMC. In addition, creating opportunities for discussion within female social network forums, highlighting to women on how to identify and use multiple opportunities of directing their partner’s attention to circumcision could promote VMMC awareness. Media resources could be tailored to provide women with skills on how to approach and behave when raising the topic of circumcision with their partners. These platforms could also assist women when communication challenges around circumcision emerge. Assessing acceptance of VMMC among adult men when VMMC is introduced to them by their female partners needs to be further assessed. Women in the study reported that gender based violence could be a risk when talking about circumcision, therefore interventions to improve respect and promote gender equality are important. Creating an environment conducive to discussing issues on VMMC with women, enhancing communication and behavioural skills of when raising the topic of VMMC with their partners could be beneficial to the VMMC programme.

## Supporting information

S1 Table IDI Guide. (DOCX)

S2 Table ISSM COREQ Checklist. (PDF)

S1 File De-identified transcripts. (ZIP)
