# Supplementary material for: “To speak or not to speak”: A qualitative analysis on the attitude and willingness of women to start conversations about voluntary medical male circumcision with their partners in a peri-urban area, South Africa
Source: PLoS One. 2019 Jan 25;14(1):e0210480. doi: 10.1371/journal.pone.0210480 (PMC6347244; doi:10.1371/journal.pone.0210480)
Supplement: S1 Table — (DOCX) [file pone.0210480.s001.docx]

S2 IDI Guide

| In-Depth Interview Guide   - 1. What do you understand by the term male circumcision?   2. Can you tell me about the different types of male circumcision?   3. Have you thought about telling your partner or family member about medical male circumcision? Why or why not?   4. What would it mean if the man in the relationship suggested that he wants to go for circumcision? Would it be any different if the woman brought this up? If yes, how so?   5. What could be some ways that a person like yourself could bring up the topic of male circumcision to your partner or family member? (Any other ways?)   6. What approach should a person like yourself AVOID when suggesting that your partner or family member go for male circumcision? Why is that so?   7. (If not already mentioned) Tell me about any experiences you have had trying to tell your partner or family member about male circumcision? (If yes: How did this come about?   8. Do you think that male circumcision is a good idea? Why or why not?   9. What do you think are the benefits are of male circumcision for couples? Why so?   10. In a relationship, who do you think should be responsible for raising the topic of male circumcision? Why is that so?   11. How would a man’s decision to be circumcised affect your opinion of him as a person? Would your opinion be more favourable? Unfavourable? Or Neutral? Why?   12. It is time for us to close, this part of the interview, but before we do, is there anything else about this topic that we haven’t discussed, that you feel is important to say? |
| --- |
