# Supplementary material for: “To speak or not to speak”: A qualitative analysis on the attitude and willingness of women to start conversations about voluntary medical male circumcision with their partners in a peri-urban area, South Africa
Source: PLoS One. 2019 Jan 25;14(1):e0210480. doi: 10.1371/journal.pone.0210480 (PMC6347244; doi:10.1371/journal.pone.0210480)
Supplement: S1 File — (ZIP) [file pone.0210480.s003.zip › QF001_QC2.docx]

PARTICIPANT ID QF001

RA: ok 02 may 2014 QF number 1 so can you please… as we discussed about the research

P: uhm

RA: so do you still agree to continue with the research

P: yes

RA: so as we discussed that I am going to ask you some questions, there is no right or wrong answer

P: yes

RA: it is just what you think is correct

P: yes

RA: so what do you understand by the word circumcision?

P: circumcision is when a man goes to cut his foreskin

RA: when a man goes to cut his foreskin

P: yes to get his foreskin cut yes

RA: uhm

P: yes

RA: is… is that what you understand about circumcision

P: yes

RA: ok so can you tell me about the different type’s circumcision?

P: there’s the one done at the clinic or hospital, and doctor, and the one done at the mountain, the traditional one

RA: uhm ok

P: yes those are the ones I know

RA: ok can you please tell me a bit more about both of them

P: how they differ??

RA: the way you think they differ or similar, any which way

P: uhm… they are not the same. They differ in a way that at the clinic when they circumcise you

RA: uhm

P: I think there is an injection that they inject you so that you do not feel pain

RA: mmm

P: and then they cut within… they do it in one day. They… a person goes there, they cut him, stitch him and do everything then goes back home. The one at the mountain they go there and get cut, I don’t know how they do it but they stay 3 weeks or 6 weeks at the mountain until they are done then come back

RA: ok so you saying they differ with time

P: yes and safety. The one at the mountain I don’t know about it safety because the one at the clinic they test before things like HIV or other sicknesses, and then the mountain one I don’t know if they check those thing cause there are no nurses so it means the mountain one they cut them without knowing their health status and that is a risk, the clinic one is safer because they cut you knowing that you have to be cut knowing your health status

RA: mmm so you saying they differ with time…

P: and safety and… yes safety

RA: ok and that at the clinic they get tested before they cut the foreskin

P: mmm and the mountain one maybe they feel pain and the clinic one they don’t feel pain

RA: ok what do you mean when you say they don’t feel pain?

P: at the clinic they inject them before

RA: ok

P: at the mountain there is no injections

RA: there are no injections

P: it means they just cut them

RA: ok mmm… so have you ever thought of telling your partner or any family member about circumcision? If you have thought about it or never thought about it can you tell me how it came about for you to think about it or not think about telling your partner or family member?

P: to think about telling someone urh it’s not easy, like my boyfriend had not went, he only went I think last week, a month ago

RA: mmm mmm

P: but I didn’t tell him, I never told him. He is the one who thought that he wants to go, I just supported him with the decision he took, for me to tell him it was difficult for me, you cannot just tell someone that go get circumcised, go and get cut no, he will ask where do you get the idea for me to get circumcised, it means there is someone else who is circumcised (P laughs) so it’s not easy to tell someone so I just supported the decision he took and said to him you doing a good thing go

RA: so you saying the reason you never thought of telling him was…

P: it’s not easy

RA: mmm

P: to just start and tell someone

RA: what do you think makes it not easy to tell someone

P: I’m afraid. Maybe he will think that maybe…maybe like sex I am not satisfied or something, that’s why I was afraid. It’s just not simple to tell someone to go maybe a child yes, a child you can tell but boyfriend no.

RA: ooh so you saying its different telling a partner…

P: boyfriend and a child yes

RA: a partner and your child or your siblings

P: yes it’s not the same, a child I can tell but my partner I won’t tell

RA: ok so do you think other women might be having the same reason not to tell their partners

P: mmm yes I think so because the person will think that you disrespecting him. You disrespecting him you telling him to go to some place yoh ( both laughs), I won’t tell him maybe I can talk to him like trying to show him that it’s a good thing but not telling him straight, I will just go around it but straight no.

RA: so you think it would be easier if you just give him urh… knowledge only that…

P: yes that after circumcision it’s going to be like this and this and this but not tell him straight that go

RA: not telling him straight ok, but what do you think can make it easier for women to tell their partners about circumcision? What do you think needs to change or be introduced?

P: it has to be talked about but I don’t know how you can tell one but it is right for them to go

RA: ooh so you think it’s important that…

P: for them to go yes it is important

RA: ok urh… you think its important reasons being

P: urh like cleanliness

RA: ok

P: if that thing is not cut aai I don’t think it’s easy to wash it (both laughs) but if he’s circumcised there is nothing preventing him to bath, but if he’s not circumcised some dirt maybe inside they stay there and cannot be cleaned, even sicknesses like when you are uncircumcised you get them simple maybe like if he sleeps with somebody else who has HIV, STI those kind of things it is easy to enter him but if circumcised then no

RA: ok

P: yes they are reduced, it does not prevent but it reduces

RA: mmm so back then you talked about tests, that at the clinic they test for HIV so do you think that it is important for a person to get tested before circumcising?

P: ok it is important I didn’t know that they test before

RA: mmm

P: I didn’t know the importance of why they should get tested before they circumcise so now for them to test them they say a person who is positive they are not supposed to circumcise them without knowing their CD4 count so if you not tested then they might cut him only to find that his CD4 count is low maybe he will not get healed or he will get sick

RA: ok maybe he will not get healed or he will get sick ok… ok so if a man in a relationship is the one that mention about circumcision and then he says… he says, ok do you think it’s different if a man or a woman suggest circumcision?

P: me or partner?

RA: or partner

P: it’s different if it’s a man saying that he wants to go it is better if it’s him who chooses for himself, if it’s me that says he must go he will go just to make me happy. He must go when it’s him who made the choose for himself that he wants to go so I will support him but not me telling him to go

RA: what do you think is different when a man suggests it from a woman suggesting it?

P: when its him who suggests it he will take responsibility for the outcome, if it was from him he will take all of the responsibility, if it was me who said go he will blame me and say it is you who said I must go isn’t that they feel pain after (P laughs), he will say it’s because of me no no

RA: ooh when he feels pain he will say its becau…

P: yes I wouldn’t have done this it’s you who said I must go do it you see now, if it’s him who made the choice he will bear the pain knowing that he’s the one who made a choice for himself

RA: ok so if he came up with the idea he will bear the pain?

P: yes and if I came up with the idea he will blame me, it’s you and you things I wouldn’t have went but if its him who went on his own its better

RA: just thinking does it benefit men more than women?

P: it benefits them (P laughs) not us, the thing is he’s the one who going to be safe like he will no longer be at risk of getting a lot of sicknesses

RA: mmm ok what kind of sicknesses?

P: STD, STI and other sexually transmitted infections

RA: ok mmm so what do you think are some ways that a women like yourself can use to talk to their partners about circumcision? What do you think could be some ways that women like yourself can bring about circumcision?

P: mmm yoh hai that I don’t know

RA: just thinking about it how you can start talking to a man, you can think of the partner that you have now or your siblings or any other man, what way do you think a man would accept or take serious when told about circumcision by a woman?

P: I can say it concerning men not specifically a partner, partner I would not tell, a child you can tell, you teach him that like they start to cut … from 9 years? Kids?

RA: 10

P: yes so maybe when he starts reaching that age you… you will start… especially a child around the age of 14 years is the one I can sit down and talk to him so I will tell him that since he’s getting old maybe he has started dating who knows, so when it’s like this you should go and cut so that you can… like even… first thing you wash it simple so if you not circumcised you can’t wash well then I will also tell him about… teach him about… about HIV and AIDS and their risk. I will tell him the reasons why he must go cut that the reasons are to reduce the risk of getting HIV and other sexually transmitted infections. I will tell him that when he circumcise it reduces the chances of him being at risk so that’s why I will tell him to go cut.

RA: mmm

P: yes but a child not partner

RA: ok so (giggles) you think that it’s easy to tell a child not…

P: a child is not the same as an adult. An adult I can tell someone who is not my partner but my partner I won’t tell no no…

RA: ok so how do they differ when telling somebody else, somebody who is not your partner?

P: another man its simple he’s not my man, imagine telling my partner that go circumcise he will want to know reasons, I will tell him and he will think maybe that there’s someone else that I’m dating and the person is circumcise and there’s a difference there, it will cause disputes so its better I keep quite.

RA: ok mmm… ok so what ways do you think a woman who wants to tell her partner about circumcision is not supposed to … put it in that way?

P: maybe …

RA: there are ways that you can tell him about, what ways should a woman not even use to talk about circumcision?

P: maybe the one u should use to tell him to circumcise is telling him about things like sicknesses

RA: mmm

P: and even bathing like cleanliness, the one you should not tell him about is sex that so and so, there you don’t go but you should just tell him about cleanliness only

RA: ok

P: to say that it’s easy to wash it but sometimes that can also get him angry, he will say that ooh you saying I smell bad ( both laughs) that’s why you saying I should cut, just tell him that it reduces those things

RA: ok so you think if you tell him about sex that’s can will make him angry?

P: yes or about being clean, bathing also can make him angry he will say it means he smells bad you see

RA: ok so all of them have…

P: mmm you know people are not the same you might say to someone go and get cut cause you will bath simple and he gets angry and say it means I have been smelling bad all along and you were afraid to tell me.

RA: ooh so you mean it depends on what kind of person they are

P: the kind yes some… if someone does to care you can tell them… it depends what kind of person they are, you will study your partner on things that make him angry and what does not make him angry and know how to tell him

RA: ok

P: mmm

RA: so you study him first but you think the better way to tell him is about bathing

P: yes

RA: and health than to tell him about sex. Ok so can you tell me about…have you ever… talked to your partner about circumcision? It can be the one you with now or a previous one; can please tell me about the experience?

P: we never spoke about it. We only spoke about it when he has decided that he wants to go and I said go you doing the right thing.

RA: ok

P: mmm so cause even with the… the… when you bath you won’t struggle, you own have to pull what what, you bath simple then with sicknesses will not enter you easy, yes you doing the right thing so then after when he got back I said aai it looks nicer than before (both laughs) at least now there’s a difference

RA: ok

P: now its good looking and when you bath you don’t struggle

RA: ooh so that was the time you talked to him about circumcision

P: yes I spoke to him at the time when he told me that he wants to go then I said you doing the right thing go then when he came back I said its good looking; you don’t struggle when taking a bath and it’s nicer there’s a difference

RA: ok so you never spoke to him… and just started to talk to him about it, he came up with it

P: yes he came up with it why he decided that he wants to go I don’t know

RA: ok mmm but just thinking do you think circumcision it’s a good idea?

P: yes it is right, it’s good

RA: ok what makes you say that?

P: mmm (P laughs) because of what I talked about sicknesses, to bath and safety, when it comes to sex I don’t see a difference

RA: ok when it comes to sex you don’t see a difference?

P: difference no

RA: ok so according to you circumcision is only for…

P: for being clean, for sexually transmitted infections, and what what, when it comes to sex for me aai, yah but I can talk about previous… relationship that has past yes a person who is not circumcised sometime you find that they hurt you but… yah even to hurt it hurts that skin when its busy doing this aai you end up feeling pain, and end up not wanting anything, you afraid to tell him that this thing of yours is disturbing me

RA: mmm ok so in other words you saying there’s a difference when it comes to…

P: yes in a way when it comes to sex there is a difference that thing hurts, it does like this… mmm so it scratches you, you end up having an infection maybe

RA: mmm ok but if… what do you think are the benefits of circumcision for people in a relationship? How do they benefit?

P: in a relationship?

RA: mmm people who are in a relationship, how do they benefit from circumcision?

P: maybe it saves the relationship, sometimes one day maybe you will date someone who is from there then the one that hurts you, you won’t stay you will leave, some women don’t even want someone who is not circumcised

RA: what could be…

P: at least when you are circumcised at least they will love you a bit more (both laughs), if you are not circumcised some will tell you that a man who is not circumcised I don’t want him (both laughs)

RA: what do you think could be the reasons for some women to say they don’t want someone who is not circumcised?

P: well they say this thing… sex its … it’s not nice

RA: they say it’s not nice

P: mmm

RA: ok

P: the one that is cut and the one that is not, they say that I do not know

RA: ok so it’s about the sex

P: mmm

RA: mmm ok… but thinking about people who are in a relationship who do you think should start to talk about circumcision?

P: a man

RA: a man

P: mmm isn’t he is the one who is supposed to cut obvious circumcision it’s a man’s thing he’s the one who is supposed to know that he must to do this… go get cut

RA: but like you said that some women do not want a man who is not circumcised, so do you think a man should raise the topic all the time like maybe if it’s a man that is not even thinking about it

P: on my side I won’t tell him

RA: on your side

P: mmm

RA: but in a relationship…

P: some women tell them to go cut this thing but on my side I’m not comfortable (p laughs) with it, mmm

RA: but I’m just saying in a relationship even if it’s not you

P: I think if we love each other and we in a relationship I think he… we must talk because if we don’t talk you will dump him and go find someone who is circumcised yes so it’s better to tell him to go cut if he want to be with you he will go get cut to save our relationship

RA: so you saying if a woman urh… doesn’t want a man who is uncircumcised and her partner is in uncircumcised, if she loves him she will…

P: she will tell him

RA: tell him

P: if she wants to be with him isn’t that when you want to be with him you make him the way you want him to be yes you will tell him to go

RA: you make him the way you want him to be…

P: each and every man isn’t that a person will tell you that I want a man that dress like this, so you the one who is supposed to make him dress the way you want him to dress if you love him

RA: ok mmm, so when a man decides that he wants to get circumcised, what does it mean to a woman? Like when a man says I have decided that I want to circumcise how does it sound to a woman?

P: I tell myself that maybe he saw that when a man is not circumcised maybe he saw that he is not right, yes cause you find that maybe when men are sitting and you are talking about circumcision and so on obvious he will not comment because he is not circumcised and will be affected by something when they talk, the men might not know that he is not circumcised but cause he knows you find that he feels otherwise and thinks that they are saying things because they know that he is not circumcised so if he is circumcised at least when other men talk he will comment that I also… so if he’s not what will he say

RA: ok

P: it is also in another way when a man is old and uncircumcised, if they find out that he’s not circumcised there nothing else that he will tell them, they will say you are not a man

RA: so if you not circumcised you are not a man?

P: no you might be a complete man but no a man is supposed to circumcise either at the mountain or clinic but he must

RA: ok so being a man is getting circumcised

P: yes according to our tradition isn’t it that a man… to be a man is when you from the mountain if you not from there you are not a man

RA: then…

P: but now I don’t like the one at the mountain but just because its tradition but I won’t take my child to the mountain

RA: so then like you saying its tradition a man is supposed to go to the mountain if a man is from the clinic is he still called a man?

P: according to me he’s a man, isn’t that they go there to be cut that thing even if they are saying that they teach those rules and what not, I don’t know but the main thing they go there for is to be cut

RA: mmm ok so the rules are not important than being cut

P: to me it’s not important because they don’t even tell people, how you will know what they teach them

RA: mmm ok

P: it’s only them who knows, to me the thing of rules is not important because the rules are kept secret it’s only them who knows what they teach them but what is left is that they go there to be cut

RA: ok but I mean when a man has taken a decision to circumcise and tell his wife how does it sound? Does she receive the news in a good way or bad?

P: to me it sounded good because it’s what I wanted him to do but was scared to tell him, I just said good shot he has made things easier for me in telling him to circumcised, I wanted him to go but didn’t know how to tell him

RA: ok…so

P: when he said I have decided to do this thing I said aai at least he went, and he was not told by me but he went and it’s what I wanted

RA: mmm ok now we going to close this part of the research but is there anything that you feel that we did not talk about? Concerning circumcision

P: mmm, no what I can ask is that why do they start to circumcise from 9 years? They… below 9 years they say that they do it at the hospital, why don’t they do it there?

RA: mmm ok I will answer you when we finish, remind me if I forget

P: mmm
