# Supplementary material for: “To speak or not to speak”: A qualitative analysis on the attitude and willingness of women to start conversations about voluntary medical male circumcision with their partners in a peri-urban area, South Africa
Source: PLoS One. 2019 Jan 25;14(1):e0210480. doi: 10.1371/journal.pone.0210480 (PMC6347244; doi:10.1371/journal.pone.0210480)
Supplement: S1 File — (ZIP) [file pone.0210480.s003.zip › QF002_QC2.docx]

Participant ID (P): QF002

RA: Thank you for showing an interest to participate in our study. Will you allow to be audio recorded?

P: Yes I agree to be audio recorded.

RA: Okay I am going to ask you question, what you know about circumcision?

P: I don’t know much because I’ve never meet someone to tell me about it. I used to hear at the clinic sometimes when clinic staff comes from the clinic to give us posters, poster for circumcision so I used to see those. Most of the things I don’t know.

RA: Okay so some of things you don’t know about circumcision. You were only aware when the clinic staff came and gave you posters.

P: Yeah they also told us to bring the children and elders to come at the clinic to circumcise.

RA: Okay are there any different types of circumcision that you know?

P: Eh eh, I don’t know

RA: By the time you get the posters, on the posters is there anything that they wrote about different types of male circumcision? Different types of circumcision you know?

P: I’ve never seen them.

RA: You’ve never heard about male circumcision, and the traditional circumcision?

P: I used to hear saying like that. That there are people who go to for traditional mountain and medical male circumcision

RA: What difference do you know, between the traditional circumcision and MMC?

P: The mountain one I don’t know. I know about the Medical Male Circumcision.

RA: What do know about medical male circumcision?

P: The doctor removes the foreskin. They cut so that it cannot keep the infection, because it keeps the infections, sickness and HIV/AIDS that’s what I can say.

RA: Have you ever thought about telling your partner or your family member about medical male

Circumcision?

P: Yes. I’ve tried to talk to my partner. I saw that he is interested he came. Now I am trying to motivate my brother but he is saying no he won’t come, when I ask why he says he will not come he says he’s scared

RA: Your brother said he’s scared?

P: Yes

RA: Okay do you think a person can benefit in medical male circumcision? What are the benefits of medical male circumcision?

P: The thing is that at the clinic you they are able to give you pills if you feel some pains because when you go to the mountain they won’t give you the pills. So you get the pains until it get healed by itself. It’s better at the clinic because they gave you things to drink and kill the pain.

RA: If your partner can say to you he wants to do medical male circumcision or to circumcise do you think that it will be the better if that is raised by men or female can they be able to talk about circumcision? Whom do you think should raise the topic about circumcision between men and the women?

P: Hm men are the one who should talk more about this. Us as women we should encourage that they should do so that we can be able to reduce the sickness out there

RA: What would it mean if a topic about circumcision is raised or a female suggest a topic rather than by men maybe? If there are women motivates men to circumcise. What would it mean?

P: It can reduce the sickness too much, because most of the people are sick of HIV, STI’s and STD’s so it can reduce there.

RA: So people are infected with STD, so it can reduce sickness

P: hmm it can reduce the sickness

RA: As a person who tried to speak to about circumcision, at the beginning you said you spoke to your partner, you spoke to your brother. Was there any difference when you started to raise the topic? As you said at the beginning again you told your brother even though your brother was scared but your partner was happy about that

P: As I started the topic and I said how about going to the clinic to circumcise, I saw that he wanted that as well. We spoke about it he said he will go as he was able to come. To my brother I don’t know what to do because his partner tried to tell him to come he said he’s scared .and it’s been a while she tried to tell him.

RA: If you talk about circumcision to your brother or to your partner what are some of things would you avoid, that you never mention by the time you talk about circumcision?

P: Nothing I told him everything about the sickness that is out there. I showed him an example like that he also saw that is something that is there.

RA: So by the time you talk to your partner and you also told your partner about circumcision, is there any difference that you saw between the two of them? As your partner was happy and go for circumcision but your brother doubt as if he doesn’t want to circumcise. When you think what was difficult about your brother not to go for circumcision?

P: I don’t know what is difficult I don’t want to lie I don’t know. He just says he’s scared he used to hear that they say there is an injection. So those are things that he scared of. Obvious he scared of the pain

RA: Oh he’s scared of pain and the injection

P: Yeah

RA: For a couple, like you what do you think are the benefits of circumcision as you went for circumcision? What are the benefits as he circumcised? What are the benefits that maybe you were not getting by the time he was not circumcised, if now there is any difference

P: Currently I don’t see because we have been sexually active. It means I will see when he’s healed. I haven’t seen anything.

RA: You haven’t seen any benefits as he’s already circumcised?

P: I haven’t seen

RA: Who do you think should promote the topic about circumcision between man and woman?

P: The woman supposed to tell her partner. It’s her who supposed to show him. To sit down and talk about it is the one who should encourage more

RA: Okay alright thank you for answering the questions that you answered we’ve to come to close the first part of an interview. I don’t know if there is anything you want to discussed or something that you want to ask
