# Supplementary material for: “To speak or not to speak”: A qualitative analysis on the attitude and willingness of women to start conversations about voluntary medical male circumcision with their partners in a peri-urban area, South Africa
Source: PLoS One. 2019 Jan 25;14(1):e0210480. doi: 10.1371/journal.pone.0210480 (PMC6347244; doi:10.1371/journal.pone.0210480)
Supplement: S1 File — (ZIP) [file pone.0210480.s003.zip › QF003_QC2.docx]

Participant ID (P): QF003

RA: (Placing recorder on the table) Eh can you give me permission to record this interview?

P: Yes I agree

RA: Okay, eh now we are going to start in this manner our interview. Eh I would love just…just for us to talk about circumcision, about medical male circumcision, eh you… can you tell me what is it you understand when we talk about medical male circumcision?

P: What I understand or what I see is right?

RA: What you understand about medical male circumcision, if you were to explain to me let’s say I was a person who does not know about circumcision, medical circumcision what would you say about medical male circumcision?

P: I would tell you that it is important that if you are a man to circumcise because this thing the foreskin is the one that enables diseases to enter males, so it is important that if you are a man you must circumcise.

RA: Okay eh so now what would you say are the types of circumcision?

P: it is the one of going to the mountain and then the one to go to the clinic

RA: Oh can you explain to me the difference, the difference of going to the clinic and that of going to the mountain just a little

P: Even though I do not have much experience but going to the mountain…me as my husband is from there, he told me that when they are at the mountain they just circumcise them, they just cut them only there is no doctor, no pills there is nothing. So at the clinic I saw it with my own eyes as my kids went there, when they finished circumcising them they gave the pills for the pain, pain killers so that they don’t feel too much pain and even when they circumcise them they inject them first so that they don’t feel that pain.

RA: Mmmh mmmh mmmh

P: Mmmh so I saw that the one at the clinic is important

RA: Eh now I hear you say that your husband went to the mountain eh do you perhaps know his reasons for him to choose to go to the mountain besides going to the clinic?

P: Okay no there he did not decide for himself, it concerned his family, the religion of his family

RA: Oh

P: He went because of his culture because he is Ndebele

RA: Oh okay so Ndebele’s in their culture-

(Participant spoke while RA was probing: They go to the mountain) is it eh a must that they go to the mountain? Oh okay so he did not have a choice in the matter?

P: He did not have a choice because even during that time this one from the clinic was not was not known much just like now

RA: Oh okay so the problem was that the promotion of the one at the clinic was not yet big?

P: Yes but even if it is like that still Ndebele’s still go to the mountain

RA: So what does he say to you between going to the clinic and going to the mountain?

P: He does not understand this one of the clinic because the children just because they went to the clinic

RA: Uhm

P: He says again that they will go back again to the mountain, so me too that’s why I’m fighting with him that they are not going

RA: So for them when you think that culturally, eh to them medical, to circumcise traditionally what does it symbolize what does it mean?

P: They say when you do it according to tradition you become a man, you become strong, you must that pain, you must feel those pains and be a man. So at the clinic they say its women, they call them women because they get doctors who inject them you see? They have not got manhood and then at the mountain they learn about their rules which they learn there, so when they go to the clinic the doctors do not give them those rules like the ones they get at the mountain.

RA: So your feelings surrounding- regarding this thing to say no going- if you go to the clinic you are a woman, what are your feelings?

P: Hheyi I don’t want to get involved with the one of the mountain, I see them as right- they are right to go to the clinic and the way death occurs at the mountain, we watch the news so I also won’t take my child and send them to the mountain to die because I do not know what is killing them so much, I don’t know how they cut them, so the doctor is better because at least the doctor has studied for this he knows what he is doing.

RA: So you, at the time he went to the mountain news about circumcision is it something maybe that you have discussed before he went to the mountain or for him it was something that was there because it was a culture, have you spoken about it before he went to the mountain?

P: Hmmm mmm we had never talked about it, just I- he told me that you know what I am going to the mountain, we just knew that Ndebele’s go to the mountain, what they do when they arrive there we did not know until this circum- from clinic appeared its then I understood that oh as this person was at the mountain he was going to do the same thing which was done to these children, so for me I don’t see the difference, I don’t see the difference

RA: Okay so, so in regards to that you had not talked about it, did you maybe have thoughts in regards to circumcision before he went to the mountain or did you know about it or you had an opinion about it?

P: No, I did not have any knowledge, I did not know anything about it and I did not even pay attention that a man had a foreskin until I saw when he got back from the mountain that hawu he said they removed this thing that was here on top, it was then I also saw that oh it is like this, I did not know, I did not have any knowledge none.

RA: Oh okay, okay so now the thing I would love to know is that eh if, if you had knowledge before, let’s say you had knowledge about it before, eh you think that it would be easy to talk to your husband about the topic of circumcision if you think about that time before they promoted the clinic? You think it would be easy to talk to him about the topic of circumcision?

P: Yes I was going to ask him that hawu husband, when you sitting while other men are going to the clinic to circumcise what about you? I was going to ask him and I was going to encourage him to go, if it wasn’t for the culture I would encourage him to come to the clinic to do it.

RA: You feel that as a man he would accept what is coming from you to say ey…man

P: (Laughing) no I do not see it, I do not see it but I was going to encourage him just

RA: Mmm

P: Even if he would not go with me when he comes, hides himself in a corner but as long as I would have tried

RA: Mmm you think that, what would make him not comfortable that the issue was brought up by you maybe or eyi?

P: Mmm is the way he is stubborn with the children, I see that even if it was him he would not agree.

RA: So in your view do you think that it is like that with all men, that men maybe are stubborn if the issue of circumcision is brought up by females?

P: In my view it’s like they are all like that, because I remember one day I was travelling in a taxi talking about the topic of circumcision and we saw the Aquarium bus parked so we were talking about the topic of circumcision. I said but this thing is important you know? The driver said to me what is its importance? I said just just you know to remove this foreskin to show that you are a man ayi he responded rough and said there is no such thing I am a Zulu I will not do this thing, at home they do not do this thing. I said it is better because in the bible it was there this circumcision of males it is there in the bible so your reasons for not doing it I do not see it, he said he just won’t do it in his life this thing is just dirt.

RA: (Giggle)

P: it is the same

RA: Mmm

P: Eh

RA: Mmm, When you think that culture maybe what role in your view culture plays on issues of circumcision and that women can show themselves as well and have an opinion in regards to circumcise do you think that culture does have a role that it plays that women must not have a voice and as well that men not to hear women?

P: I think culture that culture does oppress women because even if it is done there they do not include females, they speak as men only, women are just not included there so we do not we don’t we cannot raise the voice according to culture but at least according to the clinic we are able to raise the voice, because if the man does not want to you can just take your children and go with them, you do allow us to enter at the clinic as mothers.

RA: Mmm mmm

P: So I see that the one at clinic is right

RA: Mmm

P: Ya culture has oppression too much

RA: So the time your husband went, you just-, yours was to- how was it for you?

P: At that time like we were still growing up

RA: Mmm

P: We were still growing up so even when he went I did not care because I was staying at home and he was also staying at his home, so for me I just didn’t care.

RA: Whoah so at that time you didn’t care?

P: Eh I didn’t care unlike now as I am living with him at the house

RA: Mmm okay alright so now if we give, I give you this kind of a situation, let us say now you have the information about the clinic

P: Mmm

RA: And you and you, let us say your husband was someone who had not circumcised what strategy would you use to try and encourage him?

P: It means I would encourage him and if he ends up not agreeing I would try and come to the clinic,

RA: Mmm

P: And get in contact with those at the clinic and tell them that could they please come and encourage him but it must not be known that I am the one who came; it must look like they are entering house to house.

RA: Mmm mmm mmm

P: To encourage men

RA: Mmm

P: Maybe in that way to him it would be easy,

RA: Mmm

P: Mmm

RA: So others eh maybe your brothers, in your family have you tried to talk to them about circumcision?

P: Ah me, my brother just went when he was still young

RA: Mmm

P: It seems like it was something they spoke about in the streets with his friends

RA: Mmm

P: So he just went to the hospital back then, he went on his own and they did it to him and the hospital called to say that there is a person who is here

RA: Mmm

P: Come, and when we got there we found that no he was here to circumcise. So there is no other brother I have.

RA: Oh okay

P: Mmm

RA: Eh so if you were to give us maybe advise neh

P: Mmm

RA: that eh which approach maybe when you going to talk with a male in regards to circumcision, which approach are you not supposed to use as a female in order to try and encourage a man?

P: (Laughing) Eyi you see that one is difficult (Laughing) you know that is difficult eish I think that when you talk to him maybe tell him about the diseases that you sometimes get when you sleep with him

RA: Mmm

P: I see this as something that will be better that will make him to be encouraged neh, tell him you know what as I was sleeping with you maybe yesterday I got a pain and eh you must be like a person who really is going to the clinic for that thing then maybe they call him as well isn’t when they call both of you they check you

RA: Mmm mmm

P: Maybe the- the sister at the clinic they tell him that you know what you can do like this and circumcise

RA: Mmm

P: This problem that we usually have you won’t have any more and maybe it can encourage them as well.

RA: Mmm

P: I don’t know eyi I don’t know

RA: So because-

P: It’s difficult that one

RA: (Laughing)

P: (Laughing)

RA: Oh okay eh now now eh because in this situation where you say that you want to take the children, have you taken them, the father he no mmhhmmm still wants that no no the children must continue to go to that place there eh how was the experience when you first told the father that you going to take the children to go and circumcise, how did he take it? How did you bring it to him?

P: I told him and said you know what you see that these Ndebele things cost money because when a child goes when he comes back you must buy a cow, when he is there you must buy food, a goat. You see it is costly so I am thinking that we should take the children to the clinic because it is free and then he said he is not getting involved anymore it means he does not have any child in this house

RA: Awu

P: I said look the child is seventeen now, if the child is going there at the mountain when he is twenty five you see that it is more painful now?

RA: Mmm

P: Is it not better to go now while he is young? Since then he says he is not getting involved with the children it means they are mine alone

RA: Mmm, now you think that tradition, traditional circumcision on its own eh as far as expense which one is better?

P: It is the one from the clinic because I don’t spend anything

RA: Mmm

P: I do not spend anything even when he returns from the clinic there is nothing that I do, even food he eats everything unlike those when they are at the mountain they don’t eat certain things, food with a lot of oil they do not eat you see things like that so us at the clinic they did not tell us about all these things.

RA: Mmm

P: They said the child can eat everything

RA: Mmm mmm

P: So for me the clinic it’s, it’s the best

RA: Mmm so if- if- if you look at the advantages of going to the clinic against maybe the advantages of going to the mountain you can say whe- whe- where is the advantage on on the one from the mountain and the one from the clinic and I know you have already said you prefer the one from the clinic but if you can weigh just which one you see has disadvantages ke?

P: Mmm

RA: To them

P: I say the one from the clinic is number one, the one from the mountain down it must just end, it is killing our children the one the one from the mountain, the disadvantages of it is that it kills the children and it has a season that you go in summer, you go in winter, summer you do can’t go so the clinic its summer, its winter you go everyday they do not put a time for a person, it is always busy working.

RA: Okay

P: So I say forward with the clinic

RA: Alright so eh do you think male circumcision is a good idea?

P: Too much it is good but I am not saying if you are circumcised you won’t get HIV

RA: Mmm

P: But it is important

RA: Eh your reasons for you supporting it?

P: it is good, things like drop it is not easy for us to get as well, it is not easy to have drop and all these funny diseases, I am not saying you won’t have HIV but the drop and STD it just won’t be easy to get them

RA: So is- is there is there a sickness that women also get if a man is not- is not circumcised?

P: Eyi In my view it’s like sicknesses stay there on his thing on his foreskin

RA: Mmm mmm

P: E because after you sleep with him and you appear now you are also sick, and you get all these things

RA: Mmm... To females or or any experiences? In- what sickness? Maybe which is common?

P: Ey I don’t know, I have not gotten sick (Laughing)

RA: Whoah okay but eh when you think other females you have spoken to what do- do- do they say? Or people just general feel just of females

P: Eish most just I would hear them complain about the womb I don’t know what causes that

RA: Mmm

P: Mmm

RA: Mmm

P: So with us when we are sick most of the time we throw the blame to men

RA: Mmm

P: When you get sick down there you say no the man infected me you see

RA: Mmm

P: Men also I know that they blame females but if he is not circumcised no then in my view they will get sick worse.

RA: So could you say the benefits of circumcision for people who are married or people who are in are relationship what are they?

P: The benefits?

RA: Ya, or p-p-p people- a couple where the man is circumcised what are the benefits?

P: No in my view just that just everything is right (Laughing)

RA: Mmm

P: And there is a difference even in the sex

RA: Awu?

P: Mmm

RA: Mmm

P: There is a difference

RA: So the benefits are in sex?

P: Mmm

RA: And then eh eh for you females…if- if you can try just to explain to me a little that…

P: (Laughing)

RA: For you as a female

P: Eh

RA: The benefit you feel

P: Mmm

RA: Of a man who is circumcised and one who is not circumcised

P: Mmm

RA: What is it?

P: Eish you see now (Laughing) you see you talking other things (Laughing) No I am not comfortable with that one (Laughing)

RA: Whoah okay okay no problem there is no problem neh?

P: Ya

RA: And then eh in your- in your view eh for people that are in a relationship eh who is supposed to take the responsibility to bring this topic of circumcision?

P: To people who are in a relationship?

RA: Eh

P: Like husband and wife?

RA: Yes yes

P: Eh it’s the female if the man does not care, sorry I think the female has the right to start this topic

RA: Mmm why do you say it is the woman maybe who- who-…?

P: You know us in most of the time let me say just like me I stay in the house most of the time, watching TV I see those advertisement that eyi to circumcise is important so much you see?

RA: Mmm

P: So the man most of the time is not in the house

RA: Mmm

P: I would tell him that heyi you know I saw here on the TV they were talking about such a thing to circumcise don’t you think you should go as well?

RA: Mmm

P: You see and hear from him first that how he becomes

RA: So you think as far as women, a woman is more attentive to things that are on tv-

P: Yes

RA: Over men?

P: Actually many things women pay attention to more than men

RA: Okay okay

P: Mmm like now I said that I paid attention that my children must go now to get circumcised

RA: Mmm

P: More than their father who is waiting on that time that time which- which we don’t know when it going to come

RA: Oh okay

P: You see (Sniffing)

RA: Oh okay, okay, eh and then how would I- if you can tell me neh that if your husband was the one neh who brought the topic to you that no I eh want to go to go circumcise eh, your opinion of him how- how- how would you feel?

P: Yho I would be happy

RA: Mmm

P: I would be more than happy

RA: Mmm

P: If it is him who comes with it, I would be happy and even go with him

RA: (Laughing)

P: (Laughing)

RA: Eh…

P: (Laughing)

RA: So what would make you happy? What is it that would make you happy?

P: that he is going to circumcise

RA: Mmm mmm

P: I would be made happy just by that word that he is going to get circumcised; you see I encourage it more just because I said it is there in the bible.

RA: Whoa

P: That a male must get circumcised

RA: So so according to faith in in the bible eh circumcision you view it as something that is right?

P: It is something right

RA: E-e-e- right

P: Mmm

RA: Oh okay okay on that faith you believe that when you read it the bible, circumcision is it- what is it- your understanding when you read it in the bible they say it is good where? What is it good for or just your understanding about circumcision according to the bible

P: (Laughing) No

RA: If I can get just a bit

P: Eish you know what I do not know, I do not know how to explain it

RA: Okay okay

P: I do not know how to explain it but if it was not good God would not have sent this person that he must take all the male children and take them to the mountain in order to be circumcised it says itself straight that they must be cut in front.

RA: Mmm

P: So for me that’s why I say it’s good because even in the bible it is there

RA: Now you know because I am thinking that maybe eh according to culture they they can still take it and say okay because there was no clinic there that is way we are continuing with these things

P: Eish

RA: Of tradition, in your view does it not become a problem maybe?

P: The problem is that now it seems like they are not doing it in the right way, because if the word comes out that there are some that die at the mountain it means it is no longer something right

RA: Mmm

P: There is just nothing that is right

RA: Mmm okay okay, eh now eh we are going to the end just, just on these questions which I had in regards to to- to your thoughts about circumcision maybe those I did not ask you about or maybe you feel that no I would like to add on this issue of of circumcision?

P: In my view I think you covered every question about circumcision

RA: So also also your feelings regarding it is there something maybe you feel that you have not said about circumcision?

P: No not in my view, I don’t know as we continue with the issue maybe they will keep coming as well

RA: Oh okay

P: Mmm

RA: Okay

P: Whoa

RA: Eya

P: You know what?

RA: Mmm

P: Isn’t I don’t know that because, those who circumcise at the clinic that maybe they cut until where? I am speaking in regards to if it can still be right? Isn’t it’s a question will it be right if they cut again according to tradition?

RA: Eh…I think that because- isn’t at the clinic they do full, it’s a full circumcision I do not think that eish it can still be right that they continue to go again because they are already circumcised at the clinic they leave nothing.

P: Okay

RA: Yes

P: Thank you

RA: Okay and then (Audio paused)
