# Supplementary material for: “To speak or not to speak”: A qualitative analysis on the attitude and willingness of women to start conversations about voluntary medical male circumcision with their partners in a peri-urban area, South Africa
Source: PLoS One. 2019 Jan 25;14(1):e0210480. doi: 10.1371/journal.pone.0210480 (PMC6347244; doi:10.1371/journal.pone.0210480)
Supplement: S1 File — (ZIP) [file pone.0210480.s003.zip › QF004_QC2.docx]

PARTICIANT (P): QF004

RA: ok so mam, will you allow me to audio record this research.do you agree?

P: I allow you to record

RA: so tell me ma’am, do you know about the {} (name of clinic) clinic

P: yes I know so much

RA: you know?

P: yes

RA: do you know that there is a circumcision clinic at {} (name of clinic)?

P: yes

RA: have you been there?

P: I took kids there

RA: oh, you took kids

P: yes

RA: do you have children

P: I took my child and my two sisters children

RA: oh, you have boys

P: yes

RA: how old are they?

P: the other one is now 14

RA: oh

P: yes

RA: okay

P: and then, the other one was 16

RA: yes

P: the other one was 15

RA: okay

P: yes

RA: so do you still remember the day you accompanied them?

P: yes, I remember

RA: oh

P: is it easy to find the clinic or it is not easy?

P: it is easy because it is close and

RA: ya

P: the time we went, it was not busy

RA: yes

P: when I went I was going to book for them, but when I got there they did them the same time

RA: yes, oh okay, so it sometimes gets busy?

P: yes like in June

RA: yes

P: September

RA: yes

P: it is busy. June, so if you want your child to go during the time the schools…. July, you have to book around April. If you can book in July

RA: yes

P: there is no space. And it gets full, people come from far away

RA: oh

P: others wake up at 3 am

RA: oh, it get full

P: yes, it gets full

RA: okay, so but the time you came it was not full. What do you think causes the place to be full in June and September?

P: the schools will be closed. And people want their children to heal before they go back to school and also that, they like June and July because they believe that the child will get healed he won’t get septic, it’s that it won’t … what can I say it’s that I won’t get septic.

RA: yes

P: yes. So they take it the same way as the traditional one. Isn’t it they were taking kids in July?

RA: yes

P: they like it and that the time is longer because the children close for 4 weeks or 3 weeks

RA: yes

P: so they take it that the child will be healed; when he goes back to school he will be right

RA: oh

P: yes

RA: so, if it’s in June the children heal fast?

P: yes

RA: what do you think makes them heal fast in June? If he goes…

P: it will be winter

RA: yes

P: it’s not hot

RA: oh, when it is winter they heal fast

P: yes

RA: okay, so, I hear you saying it’s the same as the traditional one?

P: yes

RA: what is it, are there different types of circumcision?

P: yes. Previously, even now it’s still there, isn’t it they take them to initiation?

RA: yes

P: and if it is high they can see what they can do before they can take him there, they don’t just take him it’s the same as the traditional one they just take kids. I saw them checking their penises to see that it is right or what. They take them inside

RA: oh

P: even though they don’t allow the parent to enter, with my child I asked him about what they were doing and he told me that they check.

RA: oh

P: so I think it is right because they check to see how the child is

RA: okay

P: but the old one, they don’t check them before. That is why there are deaths and this one I never heard someone saying their child died

RA: yes

P: I work with people, they always take their children

RA: yes

P: I like that they also give the children pills, they drink, and they teach them on how to clean it. As I am his mother, I don’t bath him, I just boil the water for him, and pour it for him, and then he will clean himself. Even the bandage they show them how to remove it

RA: yes

P: and after two days they go back to check, to see how the kids are doing

RA: okay

P: yes

RA: so you are saying that’s the way they differ?

P: yes

RA: okay, but then when you are looking at the similarities, what is it that the traditional one doing

P: the similarity can be the way they are cutting

RA: yes

P: that could be the only similarity that these ones are cutting, and the others are also cutting, but it’s not that different

RA: it’s not different?

P: yes

RA: okay, when you say it’s not different you mean with procedure or?

P: with the procedure. Isn’t it that they would be gone for a long time and this one I go with him and then come back with him right? And I never heard that hey you have to abstain from this before you go there, and I never heard that hey you don’t have to cook for him such things, no

RA: oh, you are saying which one you need to abstain

P: the tradition

RA: the tradition, okay they don’t…

P: if I’m like this as a forty something year old woman, I’m not supposed to cook for him. But with mine I did cook for him. Imagine isn’t it I told you that took all three of them at the same time

RA: yes

P: I was cooking for them, and taking care of them I was preparing water for them to bath but they didn’t…, they could walk, I came back with them walking

RA: they say you don’t cook for the one who went to the mountain?

P: yes you don’t cook for him

RA: oh

P: yes. Like if a woman went for appointments, they say she will supress him he won’t heal, she will make him not to heal. But with the modern one there are no problems

RA: okay. When you say she went for appointments what do you mean

P: menstruation. I’m talking about menstruation

RA: oh

P: yes

RA: oh, if a woman is menstruating she is not supposed to cook for him?

P: yes. But this one doesn’t have a problem; even if you have appointments you can cook and give them food. I didn’t see them having a problem

RA: yes

P: yes

RA: okay. So you say you liked it because it is free?

P: yes. It’s free. And even there they give them food. After they are done they give them food before we go and they give them fruits

RA: yes

P: that why I say I liked it. And the staff also, yoh! they treat people very well they are all smiling , starting from… when you enter, they treat people very well. They are not short tempered. Even when they are teaching there they make jokes. A child feels free and not fears

RA: they don’t fear

P: and another thing when I was there. Even with, with the ones that were in front of me I didn’t see a child that was crying. They all came out happy, and right they don’t cry

RA: okay, so do you think it is important to check them before they circumcise here at the clinic

P: yes it is important because you will find that there is one that has a problem. Yes so in that way they are doing well

RA: what kind of a problem if you think

P: isn’t that some will have sores; they will have sores on the penis, so how will they do it if he has sores. So if they check first they are doing good, they will see if that child is right

RA: okay

P: yes

RA: so do you have a partner

P: yes

RA: oh, ok so who told you children about circumcision?

P: I was a t the clinic and they were talking that the clinic is free here, so I came back and told them. My partner took the first one, and then I took the second one and my nephews

RA: okay. But I mean who came up with this circumcision topic between you and your partner

P: oh, I always thought that my boy will go, and when he was at school, isn’t it they are with friends, and the people from {} (name of organisation) usually visit the schools

RA: yes

P: and he said all his friends when, and he said I should take him, then I took him, I wasn’t in a hurry to take him

RA: so he is the one who asked

P: yes he is the one who asked, yes, and then I called my sisters. The other one lives in Pretoria and the other one is at home. They brought them and I took them all

RA: oh, you heard from the clinic

P: I heard when is started that there is a free clinic

RA: okay. But do you think it is important for a male to circumcise?

P: yes

RA: okay. So you think it’s important in what way?

P: firstly, the life that we are living is too fast in a way that older men don’t want to sit still. So I think it reduces that you can have diseases, the risk of HIV

RA: ye s

P: yes. I think it is important in a way that it is reducing diseases, the STD’s

RA: yes

P: yes, you see

RA: when you say fast what do you mean?

P: its that, truly speaking I don’t know if it is the food that we eat these days or what. Men these days they don’t want to sit still, like now, I have a partner

RA: yes

P : you can find that outside he has other women outside. And not one, so the way it is, I think it helps in preventing those diseases, they don’t catch diseases, it’s that, it’s the same as a person that is not circumcised

RA” oh

P: yes. Its helps a bit. I think it’s better, it helps

RA: oh. For a man that has many women?

P: yes

RA: yes, and for the one that has one partner?

P: it is also right for him. Yes, you won’t know, you may find that he has diseases. So that man won’t affect her

RA: oh

P: yes

RA: so if a man is circumcised he does not get diseases

P: yes, he doesn’t get infected

RA: oh, so did you think about this at first, before your child asked you, is it something you have thought about

P: yes, I was thinking about it, let me talk about the first born, I was telling myself that I will come, I remember one day I came here to the hospital and when I got here they told me that it was already full. So when this {} (name of clinic) clinic came, I saw that it felt for us, truly speaking, its saw heard our suffering because here in the hospital it was only the hospital and it’s like they were taking about 70 people. If you want to take your child in June you have to book in December. Imagine

RA: yes

P: yes. And then…it’s that people couldn’t… even… at the doctor they say it’s around 600 to 700

RA: yes

P: at that time you are not working

RA: okay

P: you are depending on the father. And you find that you can’t afford for the child to go, when {} (name of organisation) came I saw that is has helped a lot

RA: but what is it that made you think about circumcision for your child? Isn’t it that at the beginning you said if you have multiple partners you can be protected. But for a child that is 14 what are the benefits?

P: it is also going to help him, when he grows up he will have girlfriends, so it will help in preventing him from getting diseases. And that some other time, even himself when he is with his friends they will laugh at him when he is not circumcised, so when he goes to them he will be a man

RA: if he is circumcised he is a man

P: from circumcision, yes, he is a man

RA: okay, he is a man in what way

P: isn’t it even the kids when they are circumcised, when you enter their bedrooms while bathing they cover themselves with the bathing cloth

RA: yes

P: yes, it’s not the same as before.it that he can see he is now a man

RA: oh, okay

P: yes, when you enter, you knock, they cover themselves and then say come in. when you go in you find that they have covered themselves. But before they were not doing that

RA: oh, okay

P: it’s that they don’t want me to see or what, I don’t know. It’s that they actually don’t want me to see, but it is very important

RA: so when you say that they can see that they are now me t you are talking about the ones that went to the clinic or…?

P: I’m talking about the ones that are from the clinic, medical circumcision, yes, not the one… im talking about {} (name of organisation), the one in the clinic. Im not talking about…

RA: the clinic?

P: I’m talking about the clinic

RA: so what do you think made you child to come to you instead of going to his father to tell you that he want to circumcise? As a boy to come to you as his mother

P: oh, it is because I am open to the children. I can advise them on anything about life. I always talk to them. And that most of the time their father is not with them, he is always at work. And when he is off he goes and drinks alcohol, and the kids then the truth is that most of the time their responsibility is mine, let me talk the truth, I am taking care of my children. The man is not taking care, I take care. When the kids what anything they tell me, and when they have problems they tell me.

RA: oh, okay. But do you think there is any difference if a woman talks to a man about circumcision that if he was told by another man

P: I don’t know there

RA: so now like as the child came to you instead of his father, I’m saying maybe, if it was you who started, or it was his father who told him, do you think it would be different? Like how does a man feel if he was told by a woman or a man?

P: truly speaking, I think a woman plays a big role more than a man in children. And also in the community women play a big part… I dint know but I think men are sometimes shy to tell their children

RA: yes, oh

P: but I think the most responsibility is with women. You know I also see when I have called a meeting; there will be 36 women and 2 men. So when I look at it most of the time it is women who carry the responsibility

RA: it is women?

P: yes, men don’t care. It is not that they are not doing it but most of the time it is us women. And that if you are a woman and you don’t stand up for your kids, it’s like you kids will not be the same as other children

RA: okay. You call a meeting, it’s the meeting for?

P: for crèche, because I work with people

RA: oh, so you think women are…?

P: they are most important…, I agree it is men who go for circumcision but encouragement comes from us women

RA: it’s from women?

P: yes. Even when I took the kids there, I found that there is one man and mostly it was women

RA: oh at the clinic?

P: yes. They were accompanying the children

RA: oh, okay

P: yes

RA: but in a couple, people in a relationship, who do you think is responsible for raising the circumcision topic, to start talking about it?

P: okay. With the older ones, a man is supposed to say that he want to go for circumcision

RA: yes

P: so that there are no problems

RA: oh, okay a man has to start?

P: yes, a man has to start. Otherwise when you see that your partner is not going, you have a right to ask him to go, don’t just sit go

RA: but how do you think the man would feel if it is his partner that is telling him to go and do this thing?

P: the other one would be discouraged but the other one would see that this woman loves me

RA: yes

P: the way she is talking it shows that my partner loves me she wants us to healthy

RA: okay

P: the other one would take it for granted and think that they are educated, and most of them they are educated, he will see that his partner loves him. But the old fashioned ones they would even kill you. He would also beat you up

RAL okay

P: the ones these days they understand

RA: so also a man that went to school and the one that didn’t…

P: the one that didn’t there is a difference

RA: what is the difference?

P: that this one is still behind on syllabus because he didn’t go to school. And this one because he went to school, he can see that his partner also has rights on me, she can help me to go to the clinic

RA: oh, okay. But what are ways that you think a woman can tell a man about circumcision?

P: you can tell him when you are happy that I was reading a magazine or I was reading a newspaper and I saw them talking about circumcision, how about you also go, without shouting at him talking to him nicely

RA: okay

P: yes, and even say I can accompany you ill wait outside

RA: okay.

So what do you think in a relationship makes it easy for a woman to talk to her partner about circumcision, and in others it is not easy?

P: that’s why I saw with those that are educated it becomes easy, he can tell you everything and with the one that is not you find that he is secretive about other things and not telling you. And that these days we watch TV’s and listen to the radios and it has made life easy unlike before. Because in previous days he would think that maybe you are undermining him or you are forcing him you see. These days he will understand and say you saw that it is important, and see the love that you have for him. But the old one firstly he would say that you are disrespecting him, and say you don’t love him, how can you ask such things

RA: yes. Oh, okay. So what are the things that a woman has to avoid when telling a ma about circumcision? The ones that you feel that is a woman can mention a man would take it otherwise

P: oh, like telling him about his penis that eish it is not supposed to be like this, it is not satisfying me or what, I think that is another thing that could make him angry. But if you tell him nicely that you saw on the TV advert, and that there was a man and a woman, and say you liked the way it was, I think you should also go, they are also advertising it you should go. And you will be helping me as a woman; you would have helped me as a woman.

RA: how does it help a woman?

P: it helps because he won’t get diseases and if he doesn’t get diseases a woman won’t get infected when they sleep together, a woman won’t get the diseases

RA: okay

P: and there is also HIV, if he doesn’t get HIV I won’t get HIV. But if he didn’t go, if he gets them and comes home I will get them

RA: you will get it

P: yes, and the life is shortened. We both leave the kids

RA: okay, so if you tell a man about satisfying, that you don’t satisfy me he will get angry? That is what you…

P: he will get angry too much

RA: okay, but do you think circumcision is a good idea

P: yes, it is goof

RA: what is good about it?

P: there is something that I saw form people who are from there; I will talk about my kids

RA: okay

P: kids that are circumcised, when they come back it grows, and our children, our girls want the one that grows

RA: oh, okay

P: it is that they will say hee! So and so has a small one, you see. When he is from there it grows fast, that is why I tell you that my kids cover themselves. It is because it is not the same as before

RA: oh, okay. So when he is circumcised it grows?

P: yes, in speed. I don’t know why but that is what I saw that there is a change, when you go for circumcision there is a change and it is bigger in width

RA: women love that?

P: yes, they like it that way. Children these days, our girls

RA: okay

P: sometimes you find that they say it is a whip, if you are not circumcised it is a whip

RA: why do they say it is a whip?

P: I said that one is bigger; it is that when they cut that skin it is just long, they don’t like that

RA: oh, okay. But do you think there is anything that we didn’t talk about with regards to circumcision

P: I think we covered everything
