# Supplementary material for: “To speak or not to speak”: A qualitative analysis on the attitude and willingness of women to start conversations about voluntary medical male circumcision with their partners in a peri-urban area, South Africa
Source: PLoS One. 2019 Jan 25;14(1):e0210480. doi: 10.1371/journal.pone.0210480 (PMC6347244; doi:10.1371/journal.pone.0210480)
Supplement: S1 File — (ZIP) [file pone.0210480.s003.zip › QF005_QC2.docx]

PARTICIPANT ID QF005

RA: Ok so mam we thank you for agreeing to take part in this research urh… so do you agree that we can audio record while talking during this research?

P: yes I agree

RA: you agree

P: yes

RA: ooh ok so mama tell me… can you tell me a bit about you? Anything that you can tell me about you

P: as a mother I love my life for me to live well

RA: yah

P: and not have diseases such as HIV

RA: mmm

P: and cancers

RA: yah

P: and other infectious diseases

RA: mmm

P: mmm

RA: ok ooh so you… not getting diseases is something important to you

P: yes

RA: ooh ok but mama do you know about… about circumcision

P: yes I know about circumcision

RA: mmm what can you tell me about circumcision

P: a man he…when he comes back from circumcision

RA: yah

P: he becomes more safe reason being isn’t his manhood is not… it doesn’t have that thing that covers the front, it means you cannot carry diseases

RA: ok

P: yes his manhood is clean

RA: ooh ok

P: yes

RA: so … when a man comes back from circumcision he becomes safe

P: yes

RA: ok can you tell me more about how he becomes safe

P: he becomes safe in a way of… of… of meeting with a woman

RA: ok

P: when he… his manhood cannot carry her dirt

RA: mmm

P: that would stay in him and cause other diseases

RA: mmm

P: yes when he doesn’t have that thing in the front it means he becomes clean he cannot… isn’t he will not, the diseases will be covered by…

RA: ooh ok

P: yes

RA: what do you mean by the thing in front?

P: on the manhood there is a skin that covers the manhood so that it is not in the open

RA: yah

P: so that is what is cut during circumcision

RA: ooh

P: yah it is the one that is dangerous to men and diseases

RA: ooh ok

P: yes

RA: so during circumcision they cut…

P: they cut that skin

RA: ooh ok

P: yes

RA: so you saying when the skin is there it carries…

P: it carries dirt

RA: yah

P: yes isn’t that dirt will… will form diseases

RA: mmm

P: then when you meet with a woman you can carry diseases that she got from somebody else then infect the other

RA: mmm

P: yes even on him he can get diseases

RA: ok

P: when he meets with a woman who is sick it takes the disease and hold it but when the skin is not there it becomes clean

RA: mmm when you say dirt what do you… what do you mean

P: dirt

RA: ooh

P: I mean dirt

RA: you mean dirt ok

P: mmm

RA: ooh dirt that he gets…

P: when he has sex

RA: ooh ok… ok so this dirt is found during sex

P: mmm

RA: ok so you saying during circumcision they cut that skin

P: yes

RA: they remove it there

P: yes

RA: ooh ok how many ways do you know of circumcision?

P: I only know this one of cutting only i… I don’t know any other

RA: mmm

P: mmm

RA: ooh you only know the one they cut only

P: yes

RA: ok…ok is that what you understand about circumcision

P: yes

RA: ok…ok so urh… have you ever thought of telling your partner or anyone in your family or your child about circumcision?

P: yes I wanted my kids to circumcise because I saw that it is safe so me and my family are safe

RA: yah

P: I understood that my kids have to be safe too

RA: mmm

P: mmm

RA: ok when you talk about safe, safe from what

P: from circumcision

RA: yah

P: in my family my husband is circumcised so I saw it safe for my kids to circumcise also

RA: mmm

P: mmm

RA: are you still talking about diseases when you talk about being safe?

P: yes

RA: or you talking about something else

P: I’m talking about diseases

RA: ooh so is it only your kids that you told about…

P: about circumcision

RA: mmm

P: yes it is only them that I told because others are relative’s

RA: ooh you…

P: relative’s

RA: have you told them

P: mmm

RA: ooh

P: mmm

RA: does the relatives have parents or not

P: some of them don’t have parents

RA: ooh that’s why you…

P: isn’t that now you find that a child takes a long time without circumcising you see now this things at the rural arear they are, they are not suppose to work before doing this thing

RA: ooh you saying…

P: a child can grow up isn’t at home circumcision was done culturally, in a way of culture

RA: ok

P: yes now you find that those thing are no longer there in other villages for kids to go circumcise somewhere, they are no longer there totally

RA: ooh ok

P: it’s the same with us where we stay it is no longer there

RA: its no longer there

P: yah for kids to go circumcise somewhere so kids grow up and end up old and working without circumcising

RA: yah

P: so when they get to Gauteng they find that thing is there and are supposed to go there and get help

RA: mmm

P: mmm

RA: so can you least explain to me about the traditional one you talking about

P: isn’t the cultural one they do it the same way as the western one they cut that thing

RA: yah

P: but it’s a thing that they don’t stay with us at home they stay there at the veld until they heal

RA: yes

P: mmm

RA: ooh ok

P: just like that time no one knew what was done yah it was a secret now we can talk about it, it is no longer *Koma*

RA: ooh ok

P: :yes

RA: what does the word Koma mean?

P: it means secret

RA: ooh it means secret

P: it’s a secret, it was men’s secret

RA: ok

P: as if a man was born like that naturally

RA: ooh

P: mmm

RA: they made it seem like a man is born like that

P: he is born like that

RA: ok mmm… so ok looking at the traditional one and the one here at urh… the clinic I heard you saying they are the same because they cut the foreskin and then what did you say they differ with? That the one from the clinic you stay with him at home, the traditional one he stays there till he heals

P: mmm

RA: what are some of the things they differ or are the same by?

P: they differ only because isn’t wherever you are at the veld, as the parent you don’t see him

RA: yah

P: and then this one at the clinic the modern one its nicer cause the child does it and come back at home

RA: mmm

P: the pain that he feel you can see as the parent that my child feels this pain

RA: mmm

P: I can help him this way and this way

RA: ok

P : yes so the western one the clinic one its nicer

RA: ooh the clinic one its nicer

P: mmm

RA: because he comes back home

P: yes

RA: ok so you saying they feel pain when they are from there

P: yes

RA: ok can you please explain to me a bit about what kind of pain you talking about

P: isn’t now that the child was cut

RA: yah

P: for him to heal he has to feel pain, everything when it heals

RA: mmm

P: it… to say where they cut him to heal

RA: yah

P: a child has to feel pain, so when he feels pain then you buy pain killer and give them to him

RA: ooh

P: then he becomes a bit better until he is alright

RA: ooh they don’t give them

P: mmm

RA: they don’t give them there

P: for the first day they give them

RA: ooh they give them

P: isn’t that people are not the same

RA: mmm

P: others heal fast others don’t heal fast, they give them even when they go for checkup they give them

RA: mmm ok

P: because it is possible to find that he is not able to bare the pain and he finish the pills then you are supposed to buy them

RA: ooh then you buy others

P: yes

RA: ok so… ok lets go a bit back where you were saying circumcision was a secret, who was it kept from? Who was not supposed to know about circumcision?

P: its women

RA: women only?

P: yes

RA: what do you think was the reason? For it to be kept from women only

P: we actually don’t know what they were thinking at the beginning when they said it should be a secret that’s why I’m saying they wanted it to seem like a man was just like that naturally

RA: yah

P: so that its not like a person is born like this and then he removes something and he is left like this

RA: ooh

P: yes

RA: ooh for women only

P: mmm

RA: ooh ok. Can you please explain to me about the cleanness that you were talking about earlier when you said a man becomes clean after circumcision, how does he become clean?

P: isn’t that when a man has removed that foreskin

RA: mmm

P: when he bath he just bath then it’s done, when he has that foreskin how will he bath

RA: ok

P: you cant bath well isn’t that it is covering, how will he bath inside when its covering so when he has removed the skin when he bath he is done

RA: ooh

P: yes there is no way that it can cover dirt

RA: ok and then when you say they feel pain were you talking about the ones that did the traditional one or…

P: all of them I’m sure the thing is the ones that stay there we can’t see them

RA: yah

P: they come back already healed we talking about the ones… about…about… about all of them we include all

RA: ooh all of them feel pain

P: mmm

RA: ooh ok mmm…so but can you tell me a bit about the time you told your kids about circumcision on what happened or how they reacted?

P: I explained to them about HIV and said… things… HIV was not there back in the days

RA: yah

P: you couldn’t see who is who, who has not circumcised but now that there is HIV

RA: mmm

P: it is important for you to circumcise because, reason being

RA: yah

P: you will be infected with HIV

RA: mmm

P: yes then they understood they didn’t even wait for me to… that time I was working as a domestic worker when I came back I found that they went

RA: mmm

P: only to find that the other one could not do it without me

RA: ooh

P: mmm

RA: is… ok how old are they?

P: the other one is 17

RA: mmm

P: the other one is 19

RA: mmm

P: mmm

RA: ooh you just spoke to them and the other one went by himself

P: ooh yah I spoke to them and then it was a thing of I have to save money cause it was not yet available at {} (name of clinic)

RA: ok

P: so I had to save money for them to go to a special doctor, when I was still busy saving money for the two them to go it was then offered at {} (name of clinic) before I knew it that it is available, the older one followed his peers and went

RA: mmm so do you have a partner?

P: yes

RA: ooh he is there

P: yes he is the old fashioned ones that did their thing during their times

RA: what do you mean by old fashioned?

P: I mean is the ones that… that got circumcised the traditional way

RA: the traditional way

P: mmm

RA: ok so ok how did your children take it that their father is present however you are the one who told them about circumcision?

P: they didn’t take it otherwise because like now

RA: mmm

P: he is there, I’m here with them, their father is at home holding his part of the job

RA: yah

P: so that time I was with them they didn’t want to know a lot of things

RA: ooh ok

P: they didn’t ask me why don’t you tell our father why are you saying this mmm

RA: mmm

P: they just phoned when they came back then I told their father that they have done so and they are back

RA: ok

P: mmm

RA: ooh did you talk to their father before talking to them

P: yes

RA: what did he say urh…

P: he just felt pity for them

RA: he just…

P: yes he asked if they will bear the pain and I said yes if they want it they will bare the pain

RA: ooh

P: yes

RA: didn’t he take it otherwise that you are the one who told them

P: mhmm he just felt pity for them

RA: mmm

P: to say he is not around when they go to get cut

RA: ooh he is not around

P: yes he doesn’t stay with them he stays at home

RA: ooh ok but how do you think he would feel if lets say he was not circumcised and you are the one who told him first about circumcision?

P: I don’t think it would be difficult for him

RA: yah

P: mmhm according to how men come to the clinic I saw women and men, I didn’t see the men having a difficulty isn’t this thing you talk about it before you do it

RA: ok

P: you agree about it when you in peoples sight there is no problem

RA: mmm

P: mmm

RA: ok but how do you think a man would take it if his partner suggests that he should circumcise?

P: it will depend on how you tell him

RA: ok

P: yes

RA: how do you think you should tell him in order for him to understand?

P: in a way of diseases

RA: ok

P: yes in a way of talking about diseases

RA: yah

P: mmm

RA: ok about diseases only

P: yes about diseases only isn’t in another way you will find that he starts thinking to say how does this woman take me, who did she meet and where

RA: yah

P: im not like him where will I go you see but if you talk about diseases he will understand that my wife understands

RA: ok

P: yes

RA: you will tell him about diseases, what will you say about diseases?

P: I will tell him that you should know that me and you no one knows where one is been and who is been somewhere

RA: mmm

P: one might come with diseases, what if its you

RA: yah

P: how will it be because you are a man and goes all over

RA: (RA laughs) a man goes all over?

P: yes a man goes all over

RA: ok

P: mmm isn’t a woman a lot of times

RA: yah

P: since back then we knew that a woman knows that if I go all over I will come back with… I will come back with a baggage but now that there is prevention

RA: mmm

P: they are the same we will talk about diseases

RA: mmm

P: so we have to protect against this diseases so its better you circumcise

RA: ok

P: mmm

RA: ooh a woman comes back with a baggage

P: yes but now they prevent there is no time for a woman to come back with a baggage, its just to say we are afraid of diseases

RA: ooh what are you talking about when you say baggage

P: about pregnancy

RA: ooh ok so you saying if you want to tell a man about circumcision you can tell him about diseases

P: mmm

RA: only

P: mmm

RA: ok what are the ways you think a woman should not use to tell a man about circumcision, ways that you think can make a man angry or he might take it otherwise when you tell him that way?

P: isn’t when you tell him about circumcision

RA: mmm

P: you tell him in a way that is like this man is… is like its not himself he is not the same as other men

RA: ok

P: mmm

RA: he is not the same as other men

P: he is different from other men in a way that he is not like other men

RA: ok

P: you will tell him why you soo sure, why have you not circumcised, who did you see cause you are his wife

RA: yah

P: yes

RA: ok ooh he will think that urh…urh… you saw

P: I saw someone somewhere somewhere

RA: mmm ok when you say, he will think that he is not the same as other men do you mean men that can be partners…

P: isn’t that you would have seen, you would have met another man who is circumcised

RA: yah

P: your man not circumcised

RA: mmm

P: mmm now you saying to him why are you like this

RA: mmm

P: you see even when you talk about circumcision he will be angry and say no

RA: ooh ok

P: yes

RA: ooh so other men you mean men that can be partners not your children or your siblings as they are men too

P: yes I mean… I mean partners

RA: mmm

P: yes

RA: mmm but then your siblings or children what are the ways that you think you should avoid when telling them about circumcision?

P: you will tell them… about…about ways I should not use to tell them

RA: your children or siblings and not your partner which ways do you think any woman should avoid

P: to tell children

RA: mmm

P: isn’t I will not talk to children about sex

RA: ok

P: when talking to a child I cannot tell him about sex I have to tell him about diseases he will choose which diseases they are talking about

RA: yah

P: yes

RA: what could be the reason not to tell him about sex

P: the reason why I don’t tell him about sex is that he is still under age

RA: yah

P: mmm I cannot tell him about sex

RA: mmm

P: I just have to tell him that I have to take you somewhere to prevent sicknesses

RA: mmm

P: yes but when he has reached the stage that…

RA: mmm

P: he is having sex I will tell him straight that you enter here and there you don’t know what you might get

RA: (RA laughs) ok

P: mmm

RA: ooh so you saying for kids and adults it’s the same you have to talk about diseases

P: about diseases yes

RA: and not talk about… about sex

P: mmm

RA: ok ok but what do you think are the benefits of… of circumcision in a relationship, to partners?

P: to people in a relationship the benefit is it… they will not infect each other

RA: ok

P: mmm and to be clean and to clean his manhood it has to always be clean

RA: mmm

P: yes and to prevent diseases such as cancer

RA: ok

P: yes

RA: cancer for women or for…

P: for men

RA: ooh for men

P: yes

RA: ooh ok so circumcision also prevent cancer for men

P: mmm

RA: ok

P: even to love each other, they will love each other freely

RA: mmm

P: yes

RA: in which way

P: isn’t he will know that he is safe

RA: mmm

P: mmm he will love freely taking care of himself

RA: yah

P: mmm

RA: yah

P: mmm

RA: ok so how do you think circumcision prevent cancer in men?

P: like I said that

RA: yah

P: when a man circumcise they cut that thing

RA: yah

P: because that thing the chance…that thing will be infected by diseases isn’t, other times it infects… by diseases it is not visible it is covered by that thing when its not yet cut you see

RA: ooh

P: yes they will… will…they will find out after a long time that the person has cancer

RA: ooh

P: yes

RA: ok so by being free you mean? Can you please explain a bit about being free when you said a man becomes free when it comes to…

P: when it comes to love

RA: yah

P: isn’t he will be knowing that one of the things about love is circumcision so he will be circumcised

RA: mmm

P: it means I have taken steps to be clean

RA: ooh

P: yes to say he is not at risk in his life

RA: mmm ok but just thinking do you think circumcision is a good idea

P: yes circumcision is a good idea

RA: mmm

P: mmm it reduces diseases such as HIV

RA: ok

P: yes

RA: how does it reduce such diseases as HIV?

P: isn’t that you will not

RA: yah

P: when you circumcise you have to first do… they… they counsel you first and then after being counselled you understand very well that this disease… this disease exists

RA: mmm ooh when they talk to you, you can see that ooh ok

P: this diseases its true they exists, even after circumcision you understand that you have to behave well

RA: ooh ok so you saying because a person gets counselled first before circumcising, they talk to him so he is able to open his eyes and see that the diseases do exist

P: mmm

RA: ok but what do you think are the benefits of circumcision to women? To women

P: to women

RA: mmm

P: isn’t that when a man is circumcised and when he is not

RA: mmm

P: when he is carrying diseases he will infect his woman isn’t

RA: ok

P: he was with somebody else the previous day

RA: yah

P: today, yesterday he was with {} (name of person) today he is with {} {} (name of person)

RA: mmm

P: today he will meet with {} (name of person) you see that {} (name of person) diseases infected {} (name of person)

RA: mmm

P: he carried them by that thing… he will mmm

RA: ok I heard you talking about men sleeping here and there

P: mmm

RA: so do you think circumcision is important to those men or men in general

P: it is important to all men in general

RA: mmm

P: but it becomes important in general to say you have not removed that thing so its useless

RA: yah

P: yes

RA: ok when you have not removed its useless

P: yes

RA: mmm but just thinking who do you think should start telling the other about circumcision in a relationship?

P: it’s a woman

RA: a woman

P: mmm

RA: ok reason being? Why do you think a woman should start telling a man to he should go get circumcised?

P: isn’t at home…at home it starts with a woman

RA: yah

P: yah and a lot of times a woman is the one who takes care of patients at home

RA: ok

P: mmm

RA: is it only a woman who takes… who care for patients at home

P: yes

RA: mmm ok a man does not play a part there

P: even the cleanliness at home it’s a woman who sees it

RA: ok what do you mean by cleanliness at home

P: about… we… we include everything isn’t that when someone is sick at home it has to be clean now it’s a woman who has to clean all this things, she washes, do the ironing, she sweeps

RA: mmm so do you think…

P: isn’t that when this diseases has infected you they need all this things to be perfect

RA: mmm

P: mmm

RA: so you saying a woman is the one who takes responsibility in telling her partner that…

P: yes to say now diseases, let us start that thing

RA: mmm but the ones that are afraid to tell their partners what do you think can cause that? Those they are in relationship with

P: I fail to understand because when you are in a relationship with someone it mean you are free when you with them

RA: mmm

P: now when you are unable to tell him what is making it difficult, I fail to understand

RA: you don’t know what could be the cause

P: mmm

RA: ok so when a man is… when a man is… and when I say a man I mean a partn… it can be a partner it can be your child it can be you siblings, if they come to you and tell you that he has taken a decision to circumcise as a woman what would you think of him

P: there is nothing I should think because I’m going to understand that he can understand how life is

RA: yah

P: mmm

RA: ok he understands life

P: mmm

RA: ok what do you mean when you say he understands life

P: isn’t that when we busy talking we are crying about diseases its another thing that is better in a persons life

RA: mmm

P: the child I will understand that my child want to be safe

RA: mmm

P: he understands that he will be in a relationship and others things now this and that is necessary

RA: mmm

P: mmm

RA: ok so you will understand that he understands that he has to do something like that in his life

P: mmm

RA: ok urh… let me do this… so ok but then when a man tells you that he wants to circumcise him being the first to say it, would your respond be favorable to the idea or be in-between or disagree with it?

P: I will ask him questions

RA: yah

P: like why do you want to circumcise, then he can explain then I agree

RA: yah

P: he will explain its importance to him, he cannot just say I want to circumcise and I just say its ok you can go

RA: ooh you will not just agree

P: no I have to find out why he wants to go for circum

RA: (RA laughs) why not just agree

P: no its not possible

RA: its not possible

P: no you just agree maybe I don’t understand what is circum

RA: (RA laughs) ooh ok so you actually want him to explain what it is

P: mmm

RA: ok but after that you agree

P: yes

RA: ooh ok but do you think there is a difference if a man suggests or a woman suggests first or you think it’s the same if it’s a man or woman who suggests it

P: any of them is ok

RA: any of them is ok

P: mmm its even supposed to be suggested by a man

RA: its supposed to be suggested by a man

P: yes isn’t him that is circumcising

RA: yah

P: mmm by a man is alright (RA laughs)

RA: its alright

P: mmm

RA: what is the difference when it’s a man

P: isn’t the man is the one who circumcise not a woman, its nicer when is the person who is going to do it suggesting it

RA: mmm

P: mmm

RA: ok…ok but I think we are urh… we are going to close this part of the research

P: mmm

RA: and then is there anything that you think we did not discuss? What we were discussing… is there anything you think we did not talk about?

P: mmm mmm

RA: you don’t think there is anything else, so we are going to close this first part of the research
