# Supplementary material for: “To speak or not to speak”: A qualitative analysis on the attitude and willingness of women to start conversations about voluntary medical male circumcision with their partners in a peri-urban area, South Africa
Source: PLoS One. 2019 Jan 25;14(1):e0210480. doi: 10.1371/journal.pone.0210480 (PMC6347244; doi:10.1371/journal.pone.0210480)
Supplement: S1 File — (ZIP) [file pone.0210480.s003.zip › QF006_QC2.docx]

**FACILITATOR**: QF006.

**FACILITATOR**: Ok as we have indicated that we are going to record this interview, do you still agree that we record this interview?

**PARTICIPANT:** Yes, I agree that we record the interview.

**FACILITATOR**: Ok, so the first thing that we shall do is to ask you questions and then and you will reply the way you understand it. There is no right and wrong answers on the questions that I am asking you. You answer the way you understand the question.

**PARTICIPANT:** I understand.

**FACILITATOR**: Ok. The next thing that we are going to do is that I am going to give you cards and then you are going to pack them the way you understand they should be, ok? And then we shall discuss why you pack them in that manner. And then the last thing that we are going to do is that we are going to show you messages that correspond to the picture and thereafter you will comment about them.

**PARTICIPANT:** Ok.

**FACILITATOR**: Ok. First thing please tell me about you. Anything that you can tell me about yourself?

**PARTICIPANT:** Mm. I am the resident of {} (participant address), I grew up here in {} (participant address. But I even stayed back home in {} (participant address).

**FACILITATOR**: Is it, you come from {} (participant address)?

**PARTICIPANT:** Yes, I come from {} (participant address).

**FACILITATOR**: Ok.

**PARTICIPANT:** I did my matric there and then I returned back here in {} (participant address).

**FACILITATOR**: You only did matric in {} (participant address)?

**PARTICIPANT:** Yes, I did matric in {} (participant address).

**FACILITATOR**: Alright, and then what about other grades?

**PARTICIPANT:** I completed them here.

**FACILITATOR**: Alright. Ok. So do you have children?

**PARTICIPANT:** Yes, I have one child.

**FACILITATOR**: Is he a boy or a girl?

**PARTICIPANT:** A girl.

**FACILITATOR**: A girl?

**PARTICIPANT:** Yes, she is a girl.

**FACILITATOR**: Ok, how did you know about {} (name of clinic)? Did you use it?

**PARTICIPANT:** Yes, it is my clinic and I like it very much.

**FACILITATOR**: Ok. Did you {know} that there is a circumcision clinic here in {} (name of area)?

**PARTICIPANT:** Yes. When I started seeing it I asked myself a question, is it, circumcision here in the neighbourhood? Is circumcision practised in western culture? We only knew that circumcision is only practised in Sepedi only, in Zulu culture or…or that is in your culture.

**FACILITATOR**: Alright.

**PARTICIPANT:** Therefore when I saw {} (name of clinic), hey, I thought it is more interesting.

**FACILITATOR**: Ok, you thought it is more interesting?

**PARTICIPANT:** Yes. I thought it is more interesting and I then came here and asked that I hear that circumcision is practised here in {} (name of clinic). Then I found more friendly people who explained more about it.

**FACILITATOR**: Alright, ok. Who were you asking for?

**PARTICIPANT:** I was asking on behalf of my husband and my sister’s son.

**FACILITATOR**: Ok, and your sister’s son?

**PARTICIPANT:** Yes.

**FACILITATOR**: Mm. Ok. Then you are saying that you know about the traditional circumcision?

**PARTICIPANT:** Yes, I knew about the traditional circumcision and did not know whether there is a western circumcision. I only knew about the traditional one. The initiates stay there and we do not see them.

**FACILITATOR**: Ok, they were staying where you do not see them?

**PARTICIPANT:** Yes. We did not know that a man can go to the circumcision school and come back and stay at home while healing. No.

**FACILITATOR**: Ok. Then traditionally?

**PARTICIPANT:**  Yes.

**FACILITATOR**: Ok, so you say there are two ways for circumcision, is it? There is a traditional and western way?

**PARTICIPANT:** Yes.

**FACILITATOR**: Ok, how are they similar or different?

**PARTICIPANT:** When I was busy doing my research, I asked about circumcision and they referred me to {} (name of clinic). It was for the first time to hear about this and then I decided to do more research on this.

**FACILITATOR**: Mm.

**PARTICIPANT:** I was comparing this with the traditional circumcision. This is where I understood that the western circumcision is more advanced and the patient heal very quick that those coming from the traditional circumcision as they go for check-ups and supplied with the medicines to heal their wounds. But I really do not know more about the Sepedi circumcision. The initiates go there and come back after there months.

**FACILITATOR**: Hehehe!! They come after three months?

**PARTICIPANT:** Yes. The initiates go there for a long time and come back after three months. Therefore I do not know what they are doing there.

**FACILITATOR**: Mm.

**PARTICIPANT:** They come back after three months.

**FACILITATOR**: Mm.

**PARTICIPANT:** But they come back looking unhealthy.

**FACILITATOR**: Mm. What do you mean when you say they come looking unhealthy?

**PARTICIPANT:** Yes. They come back unhealed and the initiates just stay at home and we do not know what they are doing at home.

**FACILITATOR**: Mm.

**PARTICIPANT:** Yes, in the western circumcision they explain to you what food you have to give to the circumcised person, the pills and then you treat the wound with salty water

**FACILITATOR**: Ok, then in the traditional one they do not say anything?
**PARTICIPANT:** No, they just keep quiet. That is where I become confused.

**FACILITATOR**: Mm.

**PARTICIPANT:** Yes.

**FACILITATOR**: Ok. So the way you have seen the difference between them is that in the traditional one they stay there for about 3 months whereas in the western one you come back very early?

**PARTICIPANT:** Yes.

**FACILITATOR**: Ok. Mm. So what is it that you understand about this circumcision? Let’s say circumcision in general?

**PARTICIPANT:** The way I understand, circumcision reduces many diseases. And in women we are often told that t reduces cervical cancer.

**FACILITATOR**: Ok.

**PARTICIPANT:** When the man has gone to the circumcision.

**FACILITATOR**: Ok, in women?

**PARTICIPANT:** Yes.

**FACILITATOR**: So it does not reduce diseases in…?

**PARTICIPANT:** No, it also reduces diseases in men.

**FACILITATOR**: Yaa.

**PARTICIPANT:** Yes, this means that when a man has not gone to the circumcision there are risks of being infected by the diseases, both in a woman and a man.

**FACILITATOR**: Ok, when a man has not gone to the circumcision there are many risks that both men and woman may be infected by the diseases. What kind of diseases that you know about?

**PARTICIPANT:** Men may be infected by the ghonnorea, AIDS, and the STDs. Any disease that a man may be infected with easily.

**FACILITATOR**: May be infected easily?

**PARTICIPANT:** Yes.

**FACILITATOR**: Mm. Ok did you come to the clinic and ask about it. So when you get home did explain this to him?

**PARTICIPANT:** Yes, when I got home I explained to him that when I was moving on the street I heard people saying that at {} (name of clinic) circumcision is being done, would you like to go?

**FACILITATOR**: Mm.

**PARTICIPANT:** He said that he will come back and respond to me. When he comes back he told me that he has made up his mind. I then said what did you think {} (name of participant’s partner)? He said that he has decided to go to the circumcision today. Ok, then I accompanied him there. When we get there I asked question on his behalf.

**FACILITATOR**: Mm.

**PARTICIPANT:** Alright we went there in June. In June when we got there we found many people on the queue and we came again in August. And then at the end of August he then suggested we go there again. I did not suggest that we go again but he did make this suggestion? He told me that let’s go to the clinic.

**FACILITATOR**: Mm.

**PARTICIPANT:** I then realised that a poor man has understood. We then got to the clinic and asked for information again. The explained everything to us. The booked him for Wednesday.

**FACILITATOR**: Mm.

**PARTICIPANT:** Thereafter a poor man came to the clinic, and then we took him to the clinic for counselling. I then told him that given the fact that if you are not circumcised there is a possibility that you can be infected by many diseases, I want us to go and do the blood tests.

**FACILITATOR**: Mm. Ok.

**PARTICIPANT:** Yes, he did agree with what I was suggesting and we went to do the tests. After the tests, we then went back and he was taken to the surgery.

**FACILITATOR**: Mm. Ok, you just told him and he accepted?

**PARTICIPANT**: He asked me to give him time. I explained to him that by doing this we shall avoid many diseases and then I understand that things will be enjoyable in the house.

**FACILITATOR**: What do you mean by enjoyable?

**PARTICIPANT:** They say that when a man has gone to the circumcision, sex is even enjoyable. I am still waiting for that enjoyment.

**FACILITATOR**: *Hehehe*! Where did you hear that?

**PARTICIPANT:** The thing is that as women we sit down and talk about things?

**FACILITATOR**: Ok.

**PARTICIPANT:** Yes, as women we sit down and discuss about life.

**FACILITATOR**: So, so this for women whose husbands have already went for circumcision or…?

**PARTICIPANT:** Some of the women their husband were the same as mine because they did not circumcise.

**FACILITATOR**: Ok.

And thereafter everyone took his husband to the clinic. Some of them have already gone to the circumcision.

**FACILITATOR**: Mm.

**PARTICIPANT:** Yes.

**FACILITATOR**: Ok. So you started by doing blood tests with him?

**PARTICIPANT:** Yes.

**FACILITATOR**: What made you think that it is important for you to do blood test first?

**PARTICIPANT:** No, we knew that if he has not gone to the circumcision at his age it might be highly risky for him to be infected with the diseases. He may be infected with many diseases, STD, etc. Therefore we thought that it could be better for us to do the test first before we could go.

**FACILITATOR**: Mm. Ok. When speaking about AIDS..?

**PARTICIPANT:** Yes we are older people.

**FACILITATOR**: Hehehe! Older people?

**PARTICIPANT:** Yes.

**FACILITATOR**: Ok, but did you think about this thing of circumcision before you discussed about it..?

**PARTICIPANT:** No, I was just thinking about it but not knowing where to start. And I thought that he will not agree to go to the traditional circumcision school.

**FACILITATOR**: Ok.

**PARTICIPANT:** Because he will argue that he cannot go there and stay with children. So I understood that at the clinic he will be on his own with his doctors and nurses.

**FACILITATOR**: Alright, when you say children do you mean he will be staying with children there?

**PARTICIPANT:** Yes and he will not feel comfortable.

**FACILITATOR**: Ok, but why because even here at the clinic children are coming for circumcision? Will he not have a problem there?

**PARTICIPANT:** No he don’t have a problem and he never had a problem.

**FACILITATOR**: Mm.

**PARTICIPANT:** Because I think that they are not the same. Yes I am not sure but they cannot just be the same.

**FACILITATOR**: Ok. He did not have a problem? Ok… but is he the only man that you discussed with regarding the circumcision issues?

**PARTICIPANT:** No, there are other two that I have discussed with them about this.

**FACILITATOR**: Ok, are these the people whom you are related to or what?

**PARTICIPANT:** Yes, these are the people whom I am related to.

**FACILITATOR**: How?

**PARTICIPANT:** The first one is my sister’s son and the second one is my husband’s friend.

**FACILITATOR**: Mm. Ok, your husband’s friend?

**PARTICIPANT:** Yes.

**FACILITATOR**: When you were discussing with him, how did you discuss these with him?

**PARTICIPANT:** I started by taking the pamphlet from the clinic.

**FACILITATOR**: Mm.

**PARTICIPANT:** When I arrived at home he was also arriving to visit my husband and he wanted to know about the pamphlet. He then said that he did not go to the circumcision. I then said that is not the problem. I said you need not suffer these days because we have a clinic here in {} (name of area).

**FACILITATOR**: Ok, he did not hesitate to tell you?

Yes! He did not hesitate to tell me. When he saw me reading the pamphlet, I told him because it is no longer a secret. In the past this was a secret and we did not know what is happening.

**FACILITATOR**: Mm.

Yes and now it is no longer a secret.

**FACILITATOR**: When it was a secret in the past, what was the secret for?

**PARTICIPANT:** We did not know that they were going to the circumcision. We just knew that they were going to the ritual and then come back.

**FACILITATOR**: Ok, you did not know what they were doing?

**PARTICIPANT:** Yes.

**FACILITATOR**: Ok, you only know that they are going there and then come back?

**PARTICIPANT:** What we knew was that they are going to the ritual and they would stay there for three months and come back.

**FACILITATOR**: Mm.

**PARTICIPANT:** We did not know actually what they were doing there.

**FACILITATOR**: Ok. So you talked about the tradition. In which tradition do you belong to?

**PARTICIPANT:** Sepedi.

**FACILITATOR**: So can you explain to me about the Bapedi people and how they are associated with circumcision?

**PARTICIPANT:** Yes, we do have circumcision in Sepedi.

**FACILITATOR**: Mm.

**PARTICIPANT:** And even as I am I am one of the royal family. They say even my father was practising.

**FACILITATOR**: Yaa. Practising what?

**PARTICIPANT:** He was the owner of the circumcision school when he was still alive.

**FACILITATOR**: Ok, like you he was …?

**PARTICIPANT:** Yes, people were circumcised at home.

**FACILITATOR**: Ok. Ok. At home as in the yard?

**PARTICIPANT:** Yes.

**FACILITATOR**: Ok, in the yard?

**PARTICIPANT:** Yes.

**FACILITATOR**: So how did they go about it? Were they staying in the yard?

**PARTICIPANT:** They were staying in the mountain.

**FACILITATOR**: Ok.

**PARTICIPANT:** And then they will come back during the graduation ceremony for them to prepare to go to school. They were not staying at home.

**FACILITATOR**: Ok.

**PARTICIPANT:** Yes. They were coming at home at the end of the initiation school. They will sleep at home and tomorrow their mothers would come and fetch them.

**FACILITATOR**: So do you think this is what prepared you to talk about circumcision to you husband and his friend?

**PARTICIPANT:** Yes this is because in the past this was a secret and now it is no longer a secret you can talk about it with anyone and explain it to him/her. In the past you were not allowed to tell a man to go to the circumcision.

**FACILITATOR**: Mm.

**PARTICIPANT:** Because he will ask you what am I going to do at the circumcision school? In the past it was a secret. Even the children who are going to the circumcision school, they do not tell them what they are going to do. What the only hear is when they say let’s go to the circumcision school and get the-peanuts.

**FACILITATOR**: Hehe! They are going to give them peanuts?

**PARTICIPANT:** Yes hehehe!

**FACILITATOR**: So this makes it difficult to speak with a man and persuade him to go to the circumcision school?

**PARTICIPANT:** Yes.

**FACILITATOR**: Because you did not know what was happening there.

**PARTICIPANT:** Therefore after having the circumcision pamphlet, he just asked me what they were discussing about circumcision? I then said to him that it better for us to sit down. I then said that in circumcision they refer to a person who is having a foreskin and he has to go to the circumcision so that it could be removed.

**FACILITATOR**: Mm.

**PARTICIPANT:** Yes.

**FACILITATOR**: Ok. But why do you think they wanted to keep it secret? In your thinking?

**PARTICIPANT:** I am not sure maybe they did not want to scare men who wanted to go to the circumcision or maybe they did not want us women to know what they were doing there? I have not yet understood what was the reason.

**FACILITATOR**: Mm. Ok. And then in your own thinking, and in people who have a relationship, what do you think who must talk about the issue of circumcision? A man or a woman?

**PARTICIPANT:** I think men are not brave enough than women.

**FACILITATOR**: Ok?

**PARTICIPANT:** Men are scared of a challenge.

**FACILITATOR**: Mm.

**PARTICIPANT:** Just like that.

**FACILITATOR**: What do you mean by Challenge? *Hehe*!!

**PARTICIPANT:** A man will just think that ok, when I arrived there at home and tell him about the issue of circumcision, what if she will leave me and go to other men who are circumcised? What if he is going to have doubts about me? But we have seen that not all men are the same.

**FACILITATOR**: Mm.

**PARTICIPANT:** Sometime as women we are afraid of asking a man about circumcision because we think how is it going to be? But I think that to us women as long as that is the western circumcision, it is easy because there are pamphlets that can explain everything to them.

**FACILITATOR**: Mm. So as far as you are concerned who must talk about it?

**PARTICIPANT:** I think it is we women.

**FACILITATOR**: Why?

PARTICIPANT: That is why I am saying that men are scared of a challenge. …Also our doubts are not the solutions.

**FACILITATOR**: Mm.

**PARTICIPANT:** Yes.

**FACILITATOR**: So how do you think a wife will take it if a husband thinks about it?

**PARTICIPANT:** Women are not the same and we do not have the same beliefs.

**FACILITATOR**: Mm.

**PARTICIPANT:** Ok before you could say anything you ask a woman what are her beliefs? Women do not have the same beliefs. There are some women who do not believe in circumcision be it the traditional or the western one, they do not care. They just see life continuing as normal.

**FACILITATOR**: Ok. It depends on the beliefs of a woman?

**PARTICIPANT:** Yes. Like the culture of some people do not belief in circumcision.

**FACILITATOR**: Mm.

**PARTICIPANT:** If as a woman you tell a man about circumcision, *ijoo*, he will kill you.

**FACILITATOR**: Ok, it depends on someone’s culture?

**PARTICIPANT:** Yes, I think maybe women who are…Let me give an example of rural women.

**FACILITATOR**: Yaa!

**PARTICIPANT:** They do not know about Western circumcision.

**FACILITATOR**: Mm.

**PARTICIPANT:** It is that archaic tradition where a wife was supposed to sleep separately from his husband whereas a man was expected to sleep on the bed. I then he will call you one by one to come and have sex on the bed. In that situation, it is not simple to talk to him about circumcision.

**FACILITATOR**: Yaa. Ok, what made it difficult is the issue of culture?

**PARTICIPANT:** I think culture is one of them.

**FACILITATOR**: Mm. What do you think are other issues that cause this?

**PARTICIPANT:** The problem can be on the issue of circumcision. I mean the issue of culture? A husband will ask you how did you know about circumcision. How do you know about them? How dare you tell me about circumcision issues. Where did you see them? Do you understand?

**FACILITATOR**: Mm.

**PARTICIPANT:** So, nowadays we are thankful about the modern way of circumcision. We often get pamphlets in the taxis, wherever, at the clinics and the shops where they discuss about circumcision. Then he is able to understand that these things are no longer secret. Even if you do not say anything to him but just give him the pamphlet, he will read it on his own. Maybe he will as you something after reading it.

**FACILITATOR**: Mm.

**PARTICIPANT:** If you ask him about circumcision he will ask you which circumcised man have you engaged in sexual intercourse with. Where did you see him? Just like that.

**FACILITATOR**: Mm. Ok, so first thing first he will think that you have a boyfriend outside?

**PARTICIPANT:** Yes. He will say that you already have a boyfriend.

**FACILITATOR**: Mm. Given the fact that you think that it is a woman who should initiate this discussion, what method do you think could be simple for a woman to tell his husband? How should a woman tell her husband about this?

**PARTICIPANT:** That is why I am saying that western way of doing things has made things easier for us because you get a pamphlet that discusses about circumcision. So yo take it home with you.

**FACILITATOR**: Mm.

**PARTICIPANT:** When you arrive at home you give it to him so that he will read it on his own. You can tell him that at the clinic they gave us these pamphlets you make keep yourself busy by reading them. Because if you just give it to him he will ask you where must he take it to? He will ask you who told you that I want papers? You see. It is better to say to him that they gave us these pamphlets at the clinic just keep yourself busy by reading them I am still preparing food.

**FACILITATOR**: Mm.

**PARTICIPANT:** He will read and understand.

**FACILITATOR**: Mm.

**PARTICIPANT:** That pamphlet is discussing about everything. It tells you what diseases can you avoid, etc.

**FACILITATOR**: Ok.

**PARTICIPANT:** It guides you to live certain way of life. After reading the pamphlet, he will say ok let me also go. Thereafter he will ask you where can he go for the circumcision.

**FACILITATOR**: Mm.

**PARTICIPANT:** And you will explain to him that here at {} (name of clinic) they do circumcision. Thereafter he will be able to go there.

**FACILITATOR**: Mm. But what if it is a man who starts talking about the issue of circumcision? Then how would a woman consider him?

**PARTICIPANT:** If think women will like that.

**FACILITATOR**: Mm.

**PARTICIPANT:** Yes.

**FACILITATOR**: They will like it?

**PARTICIPANT:** Women will be happy because a husband will be considering the fact that by going to circumcision they will avoid diseases in the family. He will understand that if he is infected by the disease he will pass the disease to the wife.

**FACILITATOR**: Mm.

**PARTICIPANT:** So if the husband has gone to the circumcision we shall decrease the disease infection in both of us and we shall be safe and you still need to continue and tell your wife to use condoms when having sex.

**FACILITATOR**: Mm.

**PARTICIPANT:** Circumcision does not prevent AIDS. It only prevents fewer diseases.

**FACILITATOR**: Mm. Ok, as you have spoken with your husband and your husband’s friend, how different was it to speak to these two men? Or what were the things that you spoke about that were similar when you were speaking to both of them? Or perhaps they were different where one spoke about something different from your what your husband said? How was it when you were talking to both of them?

**PARTICIPANT:** I think it was a little different because when speaking to your husband’s friend you cannot just go deeper into facts. It is not the same as speaking to your husband where everything should be straight talk about something that you know.

**FACILITATOR**: Yaa!

**PARTICIPANT:** Isn’t that he told you talk he has not gone to the circumcision?

**FACILITATOR**: Mm.

**PARTICIPANT:** So you cannot just go into details. What you have to do is just to inform him that circumcision is performed nearer here in {} (name of clinic).

**FACILITATOR**: Ok, and that is it.

**PARTICIPANT:** Yes, But… as long as you have gone to the clinic. The pamphlet will inform you about everything, and it will inform you that after circumcision there will still be a risk for the STD’s and that does not mean that you do not have to use condoms.

**FACILITATOR**: Yaa.

**PARTICIPANT:** Just continue using your condoms because circumcision does not prevent diseases such as AIDS, etc. You will continue getting those diseases as long as you do not use condoms.

**FACILITATOR**: Mm. You went deeper into details with you husband regarding these?

**PARTICIPANT:** Yes.

**FACILITATOR**: Alright. Ok. Then what if it is a wife who tells a husband about circumcision? What method do you think she must avoid in telling her husband about circumcision? That is things that she must not talk about when telling her husband about circumcision?

**PARTICIPANT:** Yes there are things that you must not tell him. For example, do not scare him by saying that when you get to the surgery they are going to cut your thing with a scissors. Those are the things that are going to make him change his mind. There is no one who want to feel pain on a very sensitive area.

**FACILITATOR**: Mm.

**PARTICIPANT:** If you say that, then he will say no I cannot stand the scissor.

**FACILITATOR**: Mm.

**PARTICIPANT:** He will say in his mind that he cannot stand the doctor’s needle? And then he will say no I am no longer going. She must tell her husband in a polite way that will encourage him to go to the circumcision.

**FACILITATOR**: Yes.

**PARTICIPANT:** And you must also tell him that you will accompany him to do circumcision just the way you did with your own husband.

**FACILITATOR**: Mm. Ok to show him that you support him?

**PARTICIPANT:** Yes.

**FACILITATOR**: So you will go with him?

**PARTICIPANT:** Yes**.**

**FACILITATOR**: Ok. So didn’t he have a problem when you were accompanying him to the circumcision?

**PARTICIPANT:** No. He did not have a problem. I went with him and we did everything. Even today we came together for a his check-up.

**FACILITATOR**: Mm. Ok. So as you said that circumcision plays a role in preventing other diseases and some STD’s, etc. what do you think are other benefits of circumcision to people?

**PARTICIPANT: Yes**, as we said it prevents different diseases, people are not the same. You will find that you can relax and wait for your husband whereas he is busy having sex with other ladies without your knowledge.

**FACILITATOR**: Ok.

**PARTICIPANT:** When you say people are not made the same, those are men and women.

**FACILITATOR**: Ok.

**PARTICIPANT:** Not that this will be done by one person. So as long as he is not circumcised, he will come back carrying those diseases. Sometimes it may happen that the condom may explode. It may explode and take this for granted without knowing the consequences that it may bring. So some people understand that when the condom explode you have to rush to the clinic before 24 hours expires. So not all people know about this emergency. One will just think that he/she will get into the shower quickly to wash off whatever the disease.

**FACILITATOR**: Hehe!

**PARTICIPANT:** Hehe! So it does not work that way. So after they have removed the foreskin during circumcision, the foreskin is no longer there to keep the diseases inside the penis.

**FACILITATOR**: Ok. Another benefit that you spoke about in the beginning was that there is difference when having sex?

**PARTICIPANT:** Yes, they told us that sex is very enjoyable (*tsokompela)*. Hehehe!

**FACILITATOR**: What is *tsokompela*?

**PARTICIPANT:** Tsokompela is a Shangaan word that means enjoyable.

**FACILITATOR**: Ok.

**PARTICIPANT:** Yes.

**FACILITATOR**: Ok. Could you please explain what women say about what is happening during sex?

**PARTICIPANT:** They said sex is enjoyable and I do not remember some of the things that they said. I am still waiting for such pleasure after seven weeks.

**FACILITATOR**: Ok.

**PARTICIPANT:** After seven weeks I will be able to say whether what they are saying is true or not.

**FACILITATOR**: Ok.

**PARTICIPANT:** They say sex is very enjoyable after circumcision**.**

**FACILITATOR**: But do you think what they are saying is true?

**PARTICIPANT:** I am still waiting to feel this. I will be able to say hey, yes indeed sex is very enjoyable.

**FACILITATOR**: Mm.

**PARTICIPANT:** Yes.

**FACILITATOR**: Ok. But do you think circumcision is a good idea?

**PARTICIPANT:** Yes, it is a very good idea. It is a good idea that is why I was brave to bring my husband for circumcision.

**FACILITATOR**: Why do you think it is a good idea?

**PARTICIPANT:** To decrease disease infection is one of the most important things.

**FACILITATOR**: Ok.

**PARTICIPANT:** But the fact that sex is enjoyable is not something that I can assure you about because I am still waiting for my husband to get healed.

**FACILITATOR**: Hehehe!

**PARTICIPANT:** Mm.

**FACILITATOR**: Ok I will go back to where you were speaking about western and traditional circumcision?

**PARTICIPANT:** Mm.

**FACILITATOR**: So did you say that in the western or modern circumcision patients heal very quickly?

**PARTICIPANT:** Yes.

**FACILITATOR**: What do you think is the reason for them to heal very quickly as compared to the traditional circumcision?

**PARTICIPANT:** I think the western circumcision is very important. Patient use their medicines, the go back to the clinic for check-ups. They also explain to them on how to treat their wound.

**FACILITATOR**: Ok on how to treat the wound?

**PARTICIPANT:** Yes.

**FACILITATOR**: Ok they go for check-ups and on how to treat the wound?

**PARTICIPANT:** Yes, I already know about this. I have already indicated that I accompanied my husband to the clinic and after he has being circumcised, I asked them on how to go about assisting him in getting the wound healed. I also asked them whether he will be able to go back to work immediately or not. They say that he can be able to go back to work after 3 days.

**FACILITATOR**: Ok

**PARTICIPANT:** They said he is going to be able to work freely without feeling any pain.

**FACILITATOR**: Ok.

**PARTICIPANT:** They said he must treat the wound by pouring two teaspoon salt into the cup of warm water. The said he must the underwear cloth to sterilise the wound because the cloth is very soft. The also assured me that he will heal fast and be able to go back to work sooner and be like other men who have gone to this type of circumcision. They warned us not to have sexual intercourse before six weeks expires.

**FACILITATOR**: Ok.

**PARTICIPANT:** Even though he feels that he is healed and there are no longer the stitches, he still has to wait for six weeks.

**FACILITATOR**: OK, they also explained this to you?

**PARTICIPANT:** Yes, but I asked the questions. This is because I wanted to make sure that when he is at home I am able to take care of him.

**FACILITATOR**: So how old is your sister’s son whom you talked to him about circumcision?

**PARTICIPANT:** He is twelve years old.

**FACILITATOR**: Ok he is twelve years old. He is still young.

**PARTICIPANT:** Yes he is still young.

**FACILITATOR**: So how did you talk to him?

**PARTICIPANT:** Yes, I explained to him. I have already assisted him and he is now a healed active young man.

**FACILITATOR**: Ok. When you started talking to him what did you say about circumcision?

**PARTICIPANT:** My sister wanted to take him to the traditional circumcision and I told her that it is better for her to take him to the {} (name of clinic) to get circumcised.

**FACILITATOR**: Yes.

**PARTICIPANT:** Ad we are going to know how do we deal with his stitches and wound so that it cannot have infection and all the things. This is because it will be the western way of treating wounds and we shall be able to be careful.

**FACILITATOR**: Ok, your sister wanted to take him to the traditional circumcision? Why did she want to take him to the traditional circumcision?

**PARTICIPANT:** I did not ask her reasons. The thing is that people’s beliefs are not the same.

**FACILITATOR**: How did you explain to her to an extent that she ended up changing her mind of taking her son to the traditional circumcision school to the clinic?

**PARTICIPANT:** I explained to her that the child’s wound is not going to be infected by the germs because he will be under great care. I told her that if she wants to take her child to the clinic it is better for to take her to the {} (name of clinic). Perhaps she still believes that her family is still organising a traditional circumcision school.

**FACILITATOR**: Ok, at her home? Ok, as you said that your father was organising it in the past?

**PARTICIPANT:** Yes.

**FACILITATOR**: Ok, she did not want to use the different one from the one that she is used to?

**PARTICIPANT:** Yes. But she did understand and took her child to the clinic.

**FACILITATOR**: Mm. Then thereafter didn’t she take her son to the traditional circumcision school?

**PARTICIPANT:** No. She did not.

**FACILITATOR**: Mm. So does your sister have a husband?

**PARTICIPANT:** Yes she has a husband.

**FACILITATOR**: Alright, Ok. How did her husband interpret this when he saw two women busy initiating the issue of circumcision to his son without him being involved? How did he take this?

**PARTICIPANT:** What I understand is that perhaps he did understand that the child health should come first and that things are no longer done as it was in the past.

**FACILITATOR**: Mm.

**PARTICIPANT:** Nowadays we know how the clinic could help you with regard to circumcision. If you meet challenges you can be able to go back to the clinic to your surgeon and ask him that after your circumcision your experienced certain problems. He will be able to assist you.

**FACILITATOR**: Mm.

**PARTICIPANT:** My sister’s husband understood that the child health comes first so to him it was better for him to allow us to take the child to the clinic to be circumcised. He never gave us any problem.

**FACILITATOR**: Mm. Ok, but to the son, you indicated that in a relationship the woman should initiate a discussion or start talking about the issue of circumcision. If he is a boy child do you think it is father or a mother who should talk about those issues?

**PARTICIPANT:** All the long they were supposed to tell you reasons why you should go to the circumcision. They were just telling you that you are now getting older boy you have to go to the circumcision school so we are taking you there tomorrow. Thereafter, in the morning after you woke up the take you there.

**FACILITATOR**: Mm.

**PARTICIPANT:** I think all the long prospective initiates did not know what was happening at the circumcision school. You were also not supposed to ask questions. Even if you happen to ask questions about circumcision they were just telling you that they are going to feed you with peanuts and then you’ll agree and go.

**FACILITATOR**: Mm. Ok. Who was telling you this? A father or a mother?

**PARTICIPANT:** A father. A boy will be told by the father and a girl will be told by a mother.

**FACILITATOR**: Alright, even the woman go there? Alright, ok and then how does female circumcision ritual work?

**PARTICIPANT:** That is why I am saying that in the past it was kept a secret. That is why we were not asking about what was happening in men’s traditional circumcision school.

**FACILITATOR**: Ok.

**PARTICIPANT:** That is why they were making sure that even women go to the traditional circumcision school. What we knew was that women were also going to the traditional school but nothing was done to them.

**FACILITATOR**: Nothing is done to them at the circumcision school? The way you say the reason for women to go to the traditional circumcision school was just a disguise that people do not wonder why only men go to the circumcision school?

**PARTICIPANT:** Yes.

**FACILITATOR**: Ok. But what do they do when they get there?

**PARTICIPANT:** Women initiates come back home every day. They come back home to have their lunchboxes. They are doing nothing there. They come back home and take their lunchboxes. They also collect firewood and make fire.

**FACILITATOR**: Mm.

**PARTICIPANT:** Thereafter they sleep there and early in the morning they come back home.

**FACILITATOR**: Alright. Ok, so but what are the similarities and differences between a male and a female initiation school?

**PARTICIPANT:** They are very different.

**FACILITATOR**: Mm.

**PARTICIPANT:** This is because we do not actually see men initiates. We only see them when they graduate. So the female initiation school is not far we are able to see that there is a female initiation school.

**FACILITATOR**: Mm.

**PARTICIPANT:** Yes.

**FACILITATOR**: Ok.

**PARTICIPANT:** So we do not see male initiation school. We do not even know to which planet they have gone to.

**FACILITATOR**: Hehehe! What do you mean by planet?

**PARTICIPANT:** Yes, what you only know is that they have gone to the circumcision school.

**FACILITATOR**: Ok, anyway I think we are at the end of the first part of this research. Do you think there is something that we did not discuss about? We are done. Ok. So as I have said that we are going to do three things, we are done with the first one. And then in the next task I am going to give you cards. You are going to put these cards into groups. So we are going to put each group the way we understand they belong together.
