# Supplementary material for: “To speak or not to speak”: A qualitative analysis on the attitude and willingness of women to start conversations about voluntary medical male circumcision with their partners in a peri-urban area, South Africa
Source: PLoS One. 2019 Jan 25;14(1):e0210480. doi: 10.1371/journal.pone.0210480 (PMC6347244; doi:10.1371/journal.pone.0210480)
Supplement: S1 File — (ZIP) [file pone.0210480.s003.zip › QF007_QC.docx]

PARTICIPANT ID QF007

RA: Ok so mam as we discussed that we are going to record urh the interview as we speak do you still agree that we can record as we continue with the research?

P: yes I agree

RA: you still agree

P: mmm

RA: ok so but before we continue what can you tell me about you, can you tell me about you a bit

P: about me

RA: yah

P: I don’t understand what you mean

RA: anything that you can tell me

P: what I love and what I don’t like

RA: anything that you can tell me about you

P: ok what I love is to live with people

RA: yah

P: like to share problems, to live together

RA: yah

P: yah

RA: ooh to share problems with people

P: yah if I have a problem

RA: yah ooh to tell people that I have this kind of a problem

P: not everyone

RA: ooh

P: let me say a friend, to take out that pain isn’t you have to tell your friend so you can be free in life

RA: yah ok so what do you mean by living together with people

P: mmm living with people isn’t urh its important let me talk about our neighbors’

RA: yah

P: yes and then it is important to live with them

RA: so is most of your neighbors men or women?

P: its women

RA: ooh its women you live with

P: yah

RA: you talk with them

P: yah we talk even with men and discuss

RA: what do you talk about when talking to men

P: no it’s like isn’t when you see something

RA: yah

P: something you don’t understand on the way

RA: mmm

P: let me give you that example then we can talk about it that this thing what is happening then there is some topics that come up

RA: ooh ok so where do you stay?

P: I stay here in {} (participant address)

RA: did you grow up here?

P: urh urh

RA: where did you grow up

P: I grew up in {} (participant address)

RA: ooh {} (participant address)

P: mmm at {} (participant address)

RA: ooh so when did you move to {} (participant address)?

P: I moved to {} (participant address) in 2009

RA: 2009

P: mmm

RA: mmm ok but did you know about the clinic here in {} (name of area) or have you ever used it before?

P: use it

RA: mmm

P: I can say I know it cause before it was here in {} (clinic address) then it moved this side

RA: ooh okay

P: yah so I come a long way with it until they built it this side

RA: mmm but did you know that there is a circumcision clinic in here

P: no it wasn’t there that time

RA: that time it was not there

P: it wasn’t there it started to be here in {} (clinic address) I think its 3 years if im not mistaken

RA: ooh so you know that it’s there

P: mmm

RA: but then what do you understand about circumcision? Anything that you understand when you hear the word circumcision

P: ok I understand this urh… isn’t when it comes to that we hear rumors most of the time from people who come from the mountain that this and that happen, others will tell you that when you are at the mountain we eat potato chips so and so its nice.

RA: mmm

P: why? They encourage others to go

RA: mmm

P: they encourage this people to go

RA: ooh ok you only hear rumors why do you say its rumors?

P: no isn’t I didn’t go there

RA: yah

P: isn’t if you are from there and I’m a man you can lie to me and say hey man that place is nice, we eat so and so we live like this so I can have a feeling to want to go

RA: mmm so what do you think is the reason for them to lie about it?

P: the reason for them to lie is that now it’s not a lot of kids that want to go there, isn’t even when you trick a child you will trick him by a small thing and then he ends up there

RA: mmm ooh they are tricking them

P: like yes like example there is one of my sibling

RA: yah

P: when his friends went he didn’t want to but they ended up sitting down with him and tricked him, and my family didn’t agree with it why? We didn’t see the importance of him going there but then he escaped to there

RA: mmm

P: he was coming from school and threw his books through the window and ran away to there why? He wanted to see what is happening there

RA: mmm ok so the family didn’t agree for him to go

P: we didn’t agreed

RA: what was the reason to not agree?

P: no the reason was that when you don’t know you will not agree cause a lot of times we just heard about kids dying there things like that

RA: mmm

P: but at the end I asked someone and he said when a child want to go there you have to accept as a parent so that he can come back

RA: ooh ok

P: cause I think this thing is true cause when my brother got there he got sick why? Isn’t we didn’t agree that its healthy or what is happening with it we didn’t free our hearts

RA: ooh ok so you have to free your heart

P: yah especially the mountain one

RA: ooh especially the mountain one

P: yes

RA: which other one do you know?

P: no isn’t mmm now there is two of them

RA: okay

P: now they come to the clinic

RA: mmm

P: yes there are some who come to the clinic and there is others who go to the mountain

RA: the mountain mmm so how many are you in your family?

P: my family

RA: yah

P: we were three we are now two

RA: ooh

P: our older brother passed away but he didn’t go

RA: ok

P: but he was on the way there unfortunately he never made it

RA: when you say he was on the way there do you mean he was going there or…

P: he had made up his mind to go there

RA: ooh

P: but he was already old

RA: okay so was he the only boy

P: they are two

RA: and the one you said went

P: he went this young one

RA: okay so who was not agreeing in the family? Who was saying no to the young one in going to the mountain

P: the young one

RA: yah

P: he didn’t go to the mountain he went to the doctor

RA: ooh ok

P: mmm isn’t a lot of times this things they do them in a traditional way culturally

RA: yah

P: yes

RA: ok how do they do them culturally?

P: ok isn’t you see Pedi people go to the mountain

RA: mmm

P: so going to the mountain is their believe to go there

RA: mmm

P: even the Ndebele on is there

RA: yah

P: you can’t go to the Ndebele one if you are Pedi and do the Ndebele culture

RA: ooh ok so you mean when one is from another culture he cannot go and circumcise in another culture

P: urh urh I don’t think it’s possible unless you are married, you got married to a Ndebele then you will do as they do

RA: mmm

P: if you are married in the Pedi culture you will do as they do

RA: okay how do they differ?

P: no the difference I would lie what I know is that Pedi people go to the mountain but the Ndebele one I don’t know if the stay in the yard or what

RA: yah

P: but women stay in the yard both the Pedi and Ndebele one, they stay in the yard but the male one I don’t know

RA: ooh there is a female one and the male one

P: mmm

RA: okay ooh and then you saying the men stay at the mountain and women stay in the yard

P: yes they stay in the yard

RA: and then how are they similar?

P: the male and female one, they differ cause men stay at the mountain

RA: yah

P: so women stay at home

RA: ooh they differ that way

P: yes

RA: ooh but then do you know what the females do

P: I didn’t go there so I would lie

RA: ok

P: I don’t know what they do but you know rumors, they also came to us and try to trick us to go to say let us go there is so and so, it is up to you whether you want to go or not but if you want and want to see those things you will go

RA: so are you Pedi

P: yes I’m Pedi

RA: so in Sepedi culture if I heard you well back there you said it’s a Sepedi culture

P: yes it’s something that is there

RA: yah

P: yes it is there okay then we are going to live like this so we are going to do this

RA: ooh ok but then you are saying in your family you didn’t agree with

P: yes isn’t it’s from the elders, they didn’t agree with it cause

RA: yah

P: they didn’t know its importance

RA: of it

P: yes but now they know what is the importance of going there.

RA: mmm ok so the clinic one and the mountain one urh how do they differ or how are they similar?

P: the clinic one I’m not sure

RA: okay

P: because first of all when they get here I don’t know how you talk to them or he just come in and do it they he leaves or you counsel them first or you talk to them to say do you know what you are doing and what is your reason

RA: mmm

P: yah

RA: ooh the clinic one you are not sure

P: I’m not sure of what you tell them or he just get here, you do him then he leaves without knowing the reason why he is doing it

RA: mmm and then the mountain one?

P: the mountain one

RA: yah

P: the way they say it, they say they get there and they teach them laws

RA: ok

P: isn’t they teach them laws and then they say when you enter you are now a man they are will teach you laws to say when you are a man you have to do this, respect your woman just like that

RA: mmm

P: but I don’t… then other things I just assume

RA: mmm

P: mmm what they teach you there is respect

RA: ok ooh they teach them respect

P: mmm

RA: to respect who

P: anybody

RA: anybody

P: mmm

RA: ooh that is the law they teach them?

P: yah they teach them the law that… isn’t urh… maybe they tell them that when you have entered here you should know that you are a man then for example isn’t a lot of times the mountain one you find that they do it when it’s cold so it means they put them

RA: yah

P: they… what is it so that they can be strong in other words it means they should be able to stand pain, even where it’s hot they have to enter

RA: mmm

P: mmm

RA: ooh the reason they do it when its cold is for them to be strong?

P: no the reason is that they were showing them then I believe that they are showing them that you see neh where you have entered then I think that for them to open it when its cold

RA: mmm

P: there is no one who can agree to go to the mountain when it’s cold and stay there

RA: yah

P: they went there because they are strong in other words

RA: ooh

P: yes why don’t they say women should go in winter, women go in summer it means there is a difference there

RA: ooh ok

P: and then for example let me say you are walking with your wife if you are wearing a jacket you are going to give it to her so that she can be warm, you are either mmm get cold it doesn’t matter

RA: ooh so you think the reason they do it in winter is so that a man can be strong and be able to take care of the woman

P: yes they tell him that a man can stand pain

RA: yah

P: they give you that thing

RA: ooh ok so can you tell me more about being a man

P: isn’t being a man

RA: yah

P: isn’t we are still on the issue of the mountain

RA: ok

P: so I said that there is no one who can agree to going to the mountain when it’s cold

RA: yah

P: they choose winter for men to go there

RA: ok

P: so they can stand the pain until the period they gave them

RA: ooh only if one is a man he has to be strong

P: yes he goes there

RA: ooh

P: in winter

RA: mmm but have you ever spoken to any man about circumcision before?

P: mmm mmm

RA: you have never spoken to any man even if it was one

P: no isn’t you just hear them talking

RA: mmm

P: when they are busy talking that hey man it’s like this that side

RA: yah

P: yes I’m human too I can hear what is happening there

RA: mmm

P: a lot of times men don’t talk, they don’t tell women to say what is happening others we can hear and steal a bit that ooh this and that is happening in that place

RA: ooh they don’t tell women?

P: it’s not easy they don’t tell women a lot of times

RA: what could be the reason

P: I don’t know their reason

RA: ok even when you ask they don’t tell

P: no they will not tell you (RA laughs) they will not say

RA: ooh you still talking about the mountain one or which place are you talking about?

P: the mountain

RA: ooh the mountain one only

P: mmm

RA: ok but do you have kids

P: yes I have two kids

RA: is it boys or girls

P: girls

RA: its only girls

P: mmm

RA: mmm so at home there is only one boy

P: mmm

RA: so you also didn’t talk to him about circumcision

P: when he went last year

RA: yah

P: its last of last year then I sat with him down with my mother and father

RA: yah

P: we spoke to him and said: we want to take you to the doctor the reason being

RA: yah

P: it’s because isn’t you see nowadays, isn’t you have to trick him to say nowadays when its like this it means you are growing up

RA: mmm

P: yes so we live in a world there is problems if you don’t go there

RA: mmm

P: and then not to say when you have been there you have to play rough

RA: mmm what do you mean by there? When you say when you have been there

P: no isn’t you explain ti him that we are taking you to the doctor

RA: mmm

P: and then at the doctor they are going to remove the skin on you

RA: yah

P: isn’t they prevent you from diseases the day you are old and meet a woman so that it is not easy to get diseases

RA: mmm

P: so that it doesn’t contain diseases in other words

RA: ok how old is he?

P: he is 16 now

RA: he is 16

P: mmm

RA: so who was telling him those things

P: me and my mother and father

RA: ooh the three of you

P: mmm

RA: ok so how did he take it when you told him

P: he agreed

RA: yah

P: then I took him there

RA: ooh you took him to the doctor

P: yah

RA: ooh ok so he didn’t have a problem

P: he didn’t have a problem. The problem was when he was supposed to go for checkups, he didn’t understand it

RA: mmm ok so when you told him about circumcision did you tell him about diseases only or is there anything else that you told him about?

P: mmm about diseases only

RA: diseases only

P: mmm

RA: ok but just thinking let’s say people who are in a relationship who do you think should start talking about circumcision?

P: isn’t a woman supposed to start talking about it

RA: ok

P: isn’t for it to be stressed too much on men it’s because of that skin cause they say it can hold diseases

RA: yah

P: ok a woman and a man should know why I will give him a reason

RA: ok

P: that is there and we can point at it

RA: ok

P: yes

RA: oh so you feel that it is a woman that has to tell a man about circumcision

P: yes

RA: oh, have you tried talking to your partner about it

P: no he had already circumcised

RA: oh he ha already gone

P: mhm

RA: but then I you tell a man if it is a woman that starts to tell a man about this circumcision what are the ways that you think she has to tell him

P: if he is not circumcised

RA: mhm, if he is not and it is a couple

P: no isn’t it the first thing I will sit down with him

RA: ya

P: then I will already know that he is not circumcised

RA: pmhm

P: yes, before I go to him must think about the approach

RA: mhm

P: isn’t it

RA: ok

P: then I will sit down with him

RA: mhm

P: then I will sit down with him then explain to him that he knows about such a thing

RA: yes

P: isn’t it, then after I will tell him that if you don’t go this thing can be a danger

RA: ya

P: isn’t it

RA: mhm, mhm

P: so it will be from him that he agreed to go or not, it is dangerous ok let me say im not si

RA: ya

P: then you know a person they have their own things and go get diseases

RA: mhm

P: then come back and infect you

RA: mhm

P: and then I will explain to him that not that you come from there

RA: mhm

P: it means you are protected

RA: ya

P: no

RA: oh you are going to explain in a good way

P: yes

RA: mhm

P: and that according to me that thing has to be removed

RA: according to you

P: yes it must be removed

RA: mhm

P: yes

RA: (Laughing) oh you just take out HIV

P: yes

RA: but what are the ways that a woman is not supposed to tell a man about when she is telling him about circumcision what are the things that you feel that when a woman is telling a man about circumcision she has to avoid and not talk about them. Isn’t it you are saying you are going to tell her in a good way so what is it that you think a woman is not supposed to mention

P: nit tell him

RA: like not tell him you said diseases you can tell him about diseases what is it that a woman is not supposed to talk about when telling a man about circumcision

P: mhm, I think not talking also has questions there it means you are afraid of him in other words

RA: ya

P: I understand that if you live with a person you take out what is in your lungs

RA: ya

P: yes, I think when you are a man I’m not supposed to be afraid of you

RA: ya

P: yes there has to be a space where by you and I can talk

RA: ya

P: I can tell myself that I’m clever

RA: ya

P: so we communicate then if you don’t agree you don’t

RA: so you tell him that you are not afraid of him

P: no not that I’m afraid of him like I don’t respect him

RA: y

P: when there is something I want to say I must say it

RA: mhm, but then when you just think of this circumcision do you think it is a good idea a good one generally

P: it’s a good idea. I can say it is good

RA: ya

P: the reason they end up saying you must do circumcision there is a reason

RA: what is the reason

P: they cant just say circumcise when there is nothing showing the importance of this

RA: mhm, so what do you think are the reasons

P: it is what makes…

RA: ya

P: not to say that when you have circumcised you have to go out

RA: ya

P: that it means your life is safe

RA: ya

P: you can play the way you want mhm it doesn’t mean that

RA: mhm, so but except being safe what else do you feel that can be a benefit of circumcision. Is there anything that you can think of now. Or lets say you are a couple what are the benefits of people who are in a relationship

P: I believe that diseases it prevents that…

RA: oh it prevents diseases only

P: mhm

RA: ok

P: isn’t it in a man most of the time he pulls the foreskin

RA: ok

P: you find that there is dirt at the front

RA: mhm

P: I believe that they remove that thing so that it doesn’t hold dirt inside

RA: mhm

P: ok

RA: when you say dirt you refer to what

P: to…

RA: I heard you talking about diseases, the dirt you mean diseases or you mean something different to this one

P: isn’t it there is

RA: ya

P: le me say sperms here when you pull this thing

RA: ya

P: you find they are inside

RA: ok

P: and you have to bath and it means every day when you bath will you be able to pull that thing to bath

RA: ya

P: no, so when they have removed that thing I think

RA: ya so what are the benefits to a woman from a man that is circumcised

P: of a woman

RA: what does a woman benefit

P: ah, even if you can write down we will get back to it

RA: you are still thinking

P: yes

RA: ok, no just what a woman feels o women feel in general what you think are the benefits of circumcision

P: I don’t know

RA: ok, no its fine. Ok err… but then lets say a man tells his wife that he wants to go for circumcision how would a woman take it how do you think a woman would feel , the woman that is being told about circumcision

P: I believe if a man tells a woman he wants to circumcise

RA: yes

P: if a woman refuses I believe that a woman doesn’t like for him to go there

RA: mhm ok

P: she doesn’t understand the reason why he has to go there

RA: mhm she doesn’t understand the reason

P: yes

RA: but then how do you think she will perceive her man what is it that could change

P: sometimes you can think that for the fact that he is thinking of going there it is because he is sick or…

RA: ya

P: the reason for wanting to go there

RA: mhm

P: the reason is… you can ask yourself questions that maybe he is sick he is hiding it from me… maybe he want to go there and come back to say no they gave this diseases there

RA: oh the other thing that a woman can think of is that the man is sick

P: he is now sick or what

RA: ok

P: isn’t it she doesn’t have better understanding of why he wants to go there

RA: mhm

P: but then when you already know there is no problem

RA: mhm

P: yes I can also ask him if I don’t know I can ask him for the reason about why he wants to go there

RA: yes

P: because you also can’t go there without knowing why you are going

RA: ok, so you can have questions on why he is going there

P: yes

RA: mhm

P: or ask him if you want to go there what the reason for going there is

RA: mhm

P: yes, isn’t it you wouldn’t know you will explain to me that this is the reason I want to go there

RA: mhm, ok so it there anything else that you think we can talk about with regards to circumcision

P: no

RA: so but if you see a person talking about circumcision we spoke about traditional circumcision at the beginning neh

P: mhm

RA: we spoke about that it is done in winter do you think there can be a [problem if it is done in summer

P: mhm

RA: when you just think of it

P: you see this one from the clinic

RA: mhm

P: when I speak of the one from the clinic I believe when I look at it, it is done anytime

RA: ya

P: so I have a question of why

RA: mhm

P: why is it that at the clinic it is anytime?

RA: mhm

P: so when I sat down with my brother he explained to me that in winter it heals fast and in summer it takes time

RA: oh you heal faster in winter

P: mhm

RA: ok so do you think the times are the same if a person is circumcised at the mountain or the clinic

P: I’m not sure if they are the same

RA: mhm

P: isn’t it at the clinic it takes 2 to 3 days so I don’t know if they are the same

RA: so have you had any personal experience on the traditional circumcision

P: no

RA: oh its just the things that you heard on the news

P: that is happening

RA: so now I said we are going to do 3 things today so I think we are going to close this part of the research and then we are going to the 2^nd^ thing, what I will do I will give you cards that have short messages then you are going to sort them into groups according to the way you think they go together or talk about one thing you put them in one place according to how they are
