# Supplementary material for: “To speak or not to speak”: A qualitative analysis on the attitude and willingness of women to start conversations about voluntary medical male circumcision with their partners in a peri-urban area, South Africa
Source: PLoS One. 2019 Jan 25;14(1):e0210480. doi: 10.1371/journal.pone.0210480 (PMC6347244; doi:10.1371/journal.pone.0210480)
Supplement: S1 File — (ZIP) [file pone.0210480.s003.zip › QF008_QC2.docx]

Participant ID: QF008

RA: Maa’m can you allow me to record the interview.

P: Ok.

RA: Thank you maa’m, we will first start with the questions that I told you about so that I can hear what opinions you have about male circumcision, and can you tell me what do you understand about medical male circumcision?

P: You mean something I know?

RA: Yes.

P: Mostly is its importance as it is helping men not to easily get disease because they are not like us in nature. We have things inside us that are protecting us from getting some diseases; they have to get circumcised because if they remove the foreskin then that is their protection you see rather than us as women we have, we have a lot of things in our womb, that are able to protect us so that we don’t get the diseases that we not supposed to get.

RA: Ok so that all, can you tell me the types of male circumcision you know?

P: Going to the Clinic and the Traditional one, the one that man goes to the mountains. And that one is traditional.

RA: Ok now let’s talk about the Clinic one what is it that you understand about Clinic one, what is happening.

P: The Clinic one I understand it because firstly the environment is clean and even where they are doing it it is clean and they neutralize everything, any weapon that they will use they… sterilizing it before they use it. They don’t use any kind of a sharp thing they use specific things for that thing when they start circumcising a man, they have got pills and they have got injections for ease up the pain, meaning they have everything to protect people so that they don’t die or to be sick. Or to be more and get sick and get diseases that he is not supposed to be having. Even food this person will be eating food that is from home, the right food on the day he is coming for circumcision so that eat something that is right, it’s not like going in the bush for more than a month, may be some have asthmatic and may be some have heart attacker anything it’s a danger that can cost him you see? Because there is no one to take care of him there even doctors, nurses it’s just him and may be with one person who has the sharp thing to use in a man’s body.

RA: Ok maa’m I understand I would also like to know what do you understand about the traditional one?

P: Traditional circumcision has a lot of dangers however there are people doing it, because whatever sharp things they are using they are not sterilized and the environment our kids are staying in is not healthy, it’s In the bush and sometimes it’s cold and sometimes it’s very hot so even the water they drink there it’s the water that the get in the rivers and the water that is from the river is a danger before you get circumcised. So those are the disadvantages and some are dangerous to them because whatever they are using to circumcise them it’s not sterilized and it’s a danger for you and your body. Before they do that you are in danger because sometimes they are using one thing for all of us and we have different types of blood and we are not the same you see that? Like some man have diseases and they are not checked before they get circumcised they just take kids to the mountains and find out that some have heart attack and some have low blood and some have high blood you see so those are some dangerous things in our kids.

RA: Ok I get that you talked about different types of circumcision but tell me did you talk with your partner at home about medical male circumcision?

P: Hmm no he got circumcised traditionally long time ago.

RA: Ok so the time he was going for circumcision were you dating already?

P: No.

RA: Ok so now that you are together have you ever talked about circumcision with him?

P: Ja we were talking about it because we have kids and they are boys and he told me that he will not take his kids to the traditional one because it has lot of things. Like… just witchcraft he is worried of witchcraft that’s why he refused and said I prefer my kids to go to the Clinic ‘I don’t mind that they go to the Clinic’ because Clinic is more safe, they will not be staying in the bush and they will not be eating unhealthy food and they will not be experiencing cold you see and be cut by unclean things because now they want money, so circumcising kids because you want money out of that so he said no i will not take my kids there in the mountain rather take them to the Clinic.

RA: So were you happy to raise that topic like to suggest that kids my go for circumcision or how was his reaction when you talked about it?

P: No he did not have a problem because he did it, but he was against that his kids must do it the way he did it. No there is no problem about that, but not in that way, because it was hard for us. It was difficult for us. It was not safe. We did it and we survived and we are healthy but so far now we see it on TV what is happening so we are questioning our salves what went wrong, because our kids are dying and these things were not happening during our time of circumcision.

RA: Ok would you say it was easy for you to talk about it?

P: Yes for me it was easy because he also did circumcision it was easy for us.

RA: So if you can tell me what do you think of the challenges that you can face as women when you are talking about circumcision, and talking about it in man?

P: There is lot because sometimes when you tell some man will say so you slept with a man who is circumcised, because you are saying there is difference between a man who is circumcised and a man who is not circumcised. That’s the first question they ask you, you know how man are. So that is the challenge I think woman are facing if you are telling me to go and do it what is it you know about it. And what is your experience or did you sleep with someone who got circumcised, so I think that’s the biggest challenge that it’s not easy for woman to just talk about this topic. I’m telling you it’s not easy. A man will think of so many things in their mind of why out of the blue you telling me to go to the Clinic or what is it that you see? Is there any difference? You know the circumcised one? So it becomes difficult for you to answer all of that.

RA: Now do you think that more man who are not circumcised when they their partners are telling them about circumcision they think that they are cheating?

P: Yes they think that you once slept with someone who is circumcised.

RA: Now in that kind of a situation what do woman need to do to convince man to consider circumcision?

P: I think what is important woman need to be patient with their partners, and avoid harassing him and give him time and chance and keep on emphasizing that it’s for his health and it’s for my goodness. I also want you to be right, and me as well. It’s not like I went out and taste a man who is circumcised, it’s just that I’m concerned about your health because this is important as it will protect us from diseases. I think patience is the best in a man because a man takes time to understand something. It’s just like when you say you want to buy a furniture, you can go and check the furniture in the shop and you go home and tell him that I saw something like this and like this and I want it, he will never buy you that same time he will tell you that he doesn’t have money, eish you woman you love things and if you keep on telling him that you want that bed he will end up buying it for you, and he will say go and buy it and if you can look at that it’s not nice , so you just have to be patient, so if you are patient he will end up seeing that my wife is serious about this so it means it’s important, she will want to ask outside and they will say no your wife is brilliant, she is wise so he will wait in the right way and we already know about it , some they say they did it already and they are happy now as a family.

RA: So from your opinion is circumcision a good idea or not?

P: Ja I think it is a nice thing.

RA: Why are you saying that?

P: For their protection because most of the time we are leaving in a time where there is lot of sickness around us. So it’s not nice to get sick so easily where as there is cure because they are saying cure not getting… and now it’s easy cause there is knowledge out there , it’s not like the old days when we use to circumcise just to gain power and to be a real man and you feel that you are a man. Now there is reason that you really really have to do this, because there is many diseases out there even doctors they don’t understand them just like HIV is it is still surprising all of us.

RA: I’m hearing you saying its old thinking when you want to do circumcision, what are you saying about that?

P: Can you see this is about man. It was not so much about even your protection. They didn’t tell us when they were circumcising us what is it that they were protecting us from, they say to be a man its fine you can sleep with around. Now they are teaching them that although you did this but there is still a danger, you don’t have to sleep around as if you don’t think properly. Because really it doesn’t mean that you are protected you can still get an HIV if you don’t use a condom. You will get it, so it’s been long telling you that you are a man.

RA: So another question I have for you, it says as far as what you heard from others, what are the benefits for the couple if a man is circumcised?

P: The benefits?

RA: Yes

P: I will benefit because you are circumcised. And I know that you are protected and I will be protected as well. You see, it’s not going to be easy for me to get diseases because it is also not easy for you to get diseases because you did something right.

RA: So it’s the protection benefit. Are there any other benefits besides protection that are there for a couple?

P: Ya even the quality of becoming together as well, there is difference in a man he becomes confident, and there is a difference between now and before he got circumcised. Can you see the difference?

RA: Now I want to know between two people in a relationship who has to talk about circumcision.

P: We both have a right to talk about it because at the end of the day it will benefit us both of us. But he will never talk about it as it is not easy for him to talk about it. And even you, if you want to talk about you just have to get an angle in term of talking about it. Don’t talk about it being angry, don’t talk about it when he is not happy, don’t just say you were with your friends and your friend told you that her partner is circumcised and it’s nice in the bedroom. No! He will be angry if you approach it that way. You must target the days he is so happy. Talk about it when you are relaxed happy talk about it after food maybe when you are in the bedroom. Say it nicely and maybe say don’t you think of going to the Clinic and do circumcision? He might ask you why are you saying that, and you will just say it can be a nice thing to do. And give him more reasons as to why are you saying that, then (inaudible). You must give reason as much as you can, explain, explain and explain and then you can tell him that if he is not satisfied he can go to the Clinic and get pamphlet as they will explain everything and then you leave him like that. Maybe he will say I will see. Then you leave him like that and then try and check him if he managed to go or not, maybe he will tell you that he went and he got the pamphlet and I will see when I can go there, you need to have a nice approach and avoid comparing him with someone else no a man doesn’t want that because he will think that you gossip about him with your friends. Just try and get a way to talk to him as your husband so that you will be fine.

RA: So that what you can say from your understanding, what is making it difficult for man to talk about circumcision with their partners?

P: Most of the time the man feel like they will be undermining themselves when they talk about this issue with their partners. They think that you will say they are not man enough, if I can tell you that ever since I was born I never went to the mountain so when he is talking like this he feels very young and they think that you will say he is not a real man. That’s they mentality they have. You find out that you stayed with a man for more than 10 years and now you want to tell him that he must get circumcised then he believes that you will disrespect him and say he is not man enough. That’s the main reason they don’t want to talk about this topic. They think that you will undermine them and may be say they are not man enough.

RA: So if let’s say for example your partner was not circumcised ne, and decide to get circumcised, how would you view him as he decided to get circumcised?

P: I was gonna be happy because now I have information why people must get circumcised, the only thing he knew was that you get circumcised so that you become a man.so now you are aware that when a man gets circumcised then its gona benefit both of you not him only but you as well. So that’s what will make me accept it and support him as well when he is saying he wants to do something like this.

RA: Is there anything else I didn’t ask you or something you would like to add and that you would like us to know in relation to this topic, topic about circumcision.

P: Hmm let me think, I think you need to help women in terms of how they can approach man about this topic. They need to be educated in terms of how they can approach their partners about this matter. They need to know the good approach, because if you are comparing him with other man that are circumcised already he might feel that you are undermining him and it’s not gonna work that way, and he will never listen to you. So the approach is very important in terms how woman talk to their partners about this topic so that man end up considering it, because the negative approach you give him, he will also give you the negative approach as well.

RA: Hmm so how do man talk with woman… (Interjection)

P: I mean its women who must have a good way of talking with their partners.

RA: Hm.

P: This means that one must be emotionally stable and make sure that you don’t impose you need to be emotionally stable, check and have timing and she must talk about it when he is happy , and when you are done eating and when you are in the bedroom relaxed then you talk about it. Then I think you can start the topic. Don’t start the topic while you are still in the dining room with kids. No! Because you might spoil everything. Maybe kids will start to ask questions that why Dad is not yet circumcised, so you also need to respect your kids as well. Even kids they talk about these things they will talk about their father that he is not circumcised and other people will have an idea now that he is not yet circumcised. You see this might affect the kids on the other side. So a man doesn’t like to be undermined by a woman they want to be treated as man in the house , so then it’s how it can go.
