# Supplementary material for: “To speak or not to speak”: A qualitative analysis on the attitude and willingness of women to start conversations about voluntary medical male circumcision with their partners in a peri-urban area, South Africa
Source: PLoS One. 2019 Jan 25;14(1):e0210480. doi: 10.1371/journal.pone.0210480 (PMC6347244; doi:10.1371/journal.pone.0210480)
Supplement: S1 File — (ZIP) [file pone.0210480.s003.zip › QF009_QC2.docx]

**PARTICIPANT ID:** QF009

**FACILITATOR**: Ok so madam, as we have said that we are going to record this interview, so do you agree that we record the interview?

**PARTICIPANT:** Yes I agree.

**FACILITATOR:** Ok, do you agree?

**PARTICIPANT**: Yes.

**FACILITATOR:** Ok, so the first thing as I have said is that I am going to ask you some questions and then you will give me answers the way you understand. There is no right or wrong answer you just answer the way you think. Please tell me anything about yourself.

**PARTICIPANT:** I am staying here in {} (participant address).

**FACILITATOR**: Yes.

**PARTICIPANT:** I am Mopedi and I am staying with my family.

**FACILITATOR**: Ok.

**PARTICIPANT:** But originally I come from {} (participant address).

**FACILITATOR**: Ok you originally come from {} (participant address) and you are Mopedi?

**PARTICIPANT:** Yes.

**FACILITATOR**: Ok, how was it growing in {} (participant address)?

**PARTICIPANT:** It was nice.

**FACILITATOR**: Mm. And then how different it is from growing here in {} (participant address)?

**PARTICIPANT:** It is different because here many things are available and when you need them you will just find them nearer. This means this are just near and it is not the same as at home. There are things that when you need them you only get them after a very long time.

**FACILITATOR**: Ok.

**PARTICIPANT:** It is at the rural village there.

**FACILITATOR**: What kind of things will you struggle to get there in the rural village?

**PARTICIPANT:** Things such as schools. Back at home you walk a long time to school. But here our schools are just nearer.

**FACILITATOR**: Ok here they are nearer?

**PARTICIPANT:** Yes and eve transport is easily accessible. Back at home you take a long time waiting for a transport. But here transport is plenty.

**FACILITATOR**: Ok, it is plenty? So you said you stay with your family? Who are you stating with?

**PARTICIPANT:** I am staying with my husband and kids.

**FACILITATOR**: Ok, who are your kids? A boy or a girl?

**PARTICIPANT:** A girl and a boy.

**FACILITATOR**: Ok, how many years do they have?

**PARTICIPANT:** A boy has thirteen years and the girl is only turning two years on the 12^th^ of October.

**FACILITATOR**: So did you know about {} (name of clinic)?

**PARTICIPANT:** Yes, I knew about it.

**FACILITATOR**: Did you use it before?

**PARTICIPANT:** Yes.

**FACILITATOR**: Do you know that there is a circumcision clinic here?

**PARTICIPANT:** Yes.

**FACILITATOR**: Ok, what is it that you know about circumcision? The moment you here about the word circumcision what do you understand?

**PARTICIPANT:** What I understand about circumcision is that there is a skin that is removed from the penis.

**FACILITATOR**: Mm. Ok. The skin?

**PARTICIPANT:** Yes.

**FACILITATOR**: How is this skin removed?

**PARTICIPANT:** I hear that it is cut out.

**FACILITATOR**: Ok. Where is this skin?

**PARTICIPANT:** On the penis.

**FACILITATOR**: On the penis?

**PARTICIPANT:** Yes.

**FACILITATOR**: Ok, so how circumcision types do you know?

**PARTICIPANT:** There are two types that I know.

**FACILITATOR**: Do you know two types?

**PARTICIPANT:** Yes.

**FACILITATOR**: Yaa. What are they?

**PARTICIPANT:** Traditional and medical circumcision.

**FACILITATOR**: Traditional and medical circumcision?

**PARTICIPANT:** Yes.

**FACILITATOR**: Ok, So can you please explain both these circumcisions? Or maybe how are they similar or different? Or how are their procedures? Anything that you know about both of them.

**PARTICIPANT:** In the traditional circumcision people who perform circumcision are not well trained on how to perform circumcision.

**FACILITATOR**: Mm. Ok.

**PARTICIPANT:** They do not have enough information.

**FACILITATOR**: Oo, ok.

**PARTICIPANT:** And the other thing is that they are using traditional medicine. Then in medical circumcision people who are performing it do have enough information.

**FACILITATOR**: Yes.

**PARTICIPANT:** They know all the procedures that are performed so that the process of circumcision could be complete.

**FACILITATOR**: Ok so that the procedures could be a success?

**PARTICIPANT:** Yes.

**FACILITATOR**: Ok, so what information do you think the traditional surgeons need or do you think they lack?

**PARTICIPANT:** I think that when they cut the skin there is somewhere where they are not supposed to cut. That is they do not follow the correct procedure and ultimately the initiates end up dying because of botched circumcision.

**FACILITATOR**: Ok.

**PARTICIPANT:** You will find that sometimes the initiate does not heal to an extent that they take him to the hospital.

**FACILITATOR**: Ok.

**PARTICIPANT:** This is because his family members become worried when they see that he is not recovering.

**FACILITATOR**: Ok, the do not have enough information to be able to perform the operation?

**PARTICIPANT:** Yes.

**FACILITATOR**: Mm. And then when you say they use traditional medicine what are you referring to? What kind of medicine are you referring?

**PARTICIPANT:** The herbs. What they are using. They sometimes drink some of them and some of them they smear the whole body with it.

**FACILITATOR**: Yes. Then what do you think are their purpose?

**PARTICIPANT:** Hey, I do not know their purpose because in most cases their circumcision ritual is a secret. The circumcision process often does not come to an end.

**FACILITATOR**: Ok, it does not come to an end?

**PARTICIPANT:** No.

**FACILITATOR**: What are the reason that the process does not come to an end?

**PARTICIPANT:** Perhaps the herbs that they put on their bodies are not suitable for their bodies. They are not suitable to be used to heal the wound.

**FACILITATOR**: Mm. Ok, and they give them the medicines to heal?

**PARTICIPANT:** Yes so that they could heal.

**FACILITATOR**: Ok. So what do you think is being done correctly here at the clinic? If you know?

**PARTICIPANT:** They explain to you that when you are going to be circumcised what you’ll need to expect.

**FACILITATOR**: Yes.

**PARTICIPANT:** So that you know what is going to happen.

**FACILITATOR**: Mm.

**PARTICIPANT:** What will be its treatment and for how long is it going to take?

**FACILITATOR**: Ok.

**PARTICIPANT:** That is before they perform circumcision they explain everything to you.

**FACILITATOR**: Ok. Before they circumcise you they explain everything to you?

**PARTICIPANT:** Yes.

**FACILITATOR**: Is that the difference between traditional circumcision and medical circumcision that is performed at the clinic.

**PARTICIPANT:** Yes because in traditional circumcision for example they would just tell the twelve year old boys to come to the circumcision school without explaining anything to them. They just take them to the initiation school and when they get there they are circumcised. The young boys do not even know why they are been circumcised.

**FACILITATOR**: Ok, this is not discussed beforehand?

**PARTICIPANT:** It is not something that is discussed first so that people could understand that something is going to be done on them.

**FACILITATOR**: Is it?

**PARTICIPANT:** Yes.

**FACILITATOR**: Ok a young boy is just taken there without his concerned or the concerned of his parents?

**PARTICIPANT:** Yes, some of the parents do agree that their children be taken to the circumcision school but some of the parents just hear that their kids have gone there without their concerned.

**FACILITATOR**: Ok, then who decides that a particular child has to go to the initiation school?

**PARTICIPANT:** The parent has to decide.

**FACILITATOR**: Yes. Isn’t that you say that in traditional circumcision some of the boys are taken there without the concern of the parents?

**PARTICIPANT:** Yes.

**FACILITATOR**: So who comes with the idea that the boys have to go there without the parents’ knowledge?

**PARTICIPANT:** The parents who like the tradition.

**FACILITATOR**: It is the parents?

**PARTICIPANT:** Yes, it is the parents.

**FACILITATOR**: Ok. But what about those parents who do not know that their children have been taken there?

**PARTICIPANT:** The boy will go there with his friends. When a poor boy has gone there without the permission of the parents, the owners of the initiation school do no bring him back home and try to talk with his parents about this. The parents will just hear that the child has gone to the circumcision school.

**FACILITATOR**: Ok.

**PARTICIPANT:** That is why I am saying that in the traditional circumcision they do not have enough information on how the process should be handled.

**FACILITATOR**: Yes!

**PARTICIPANT:** If they were taught correctly they cannot just allow a boy to come to their ritual without the concerned of his parents. Instead they should bring back the child home.

**FACILITATOR**: Mm. So do you think it is important for the parent to know when a child wants to go to the circumcision school?

**PARTICIPANT:** Yes it is important.

**FACILITATOR**: Why are you saying that it is important?
**PARTICIPANT:** Because circumcision protects against many things.

**FACILITATOR**: Yes!

**PARTICIPANT:** These may be against the diseases and cancers.

**FACILITATOR**: Yes. Ok, you think circumcision prevents against the diseases?

**PARTICIPANT:** Yes.

**FACILITATOR**: Ok, what kind of diseases are being prevented by circumcision?

**PARTICIPANT:** By this I mean that circumcision prevents you from being infected with disease easily.

**FACILITATOR**: Ok.

**PARTICIPANT:** This means that circumcision does not prevents diseases completely but that diseases not be able to infect a person very easily. It is because of circumcision that diseases are not powerful to a man who is circumcised. If a circumcised person is infected with sexually transmitted diseases, such person does not become weak easily.

**FACILITATOR**: Mm. Ok. But what disease can you mention that you know about?

**PARTICIPANT:** And… HIV. They are not that plenty. And also even sexually transmitted diseases are not able to infect him.

**FACILITATOR**: Mm. Ok. But have you ever thought of telling a man about circumcision? Be it you son, your partner or your brother.

**PARTICIPANT:** Yes I told my son before I could take him to the clinic for circumcision.

**FACILITATOR**: Ok have you already taken him for circumcision?

**PARTICIPANT:** Yes.

**FACILITATOR**: Ok, and you told him about it before?

**PARTICIPANT:** Yes, in told him.

**FACILITATOR**: Ok could you please briefly tell me about that experience? How did you tell him? How did you decide that it is time to tell him something like this?

**PARTICIPANT:** I told him when he was still 10 years old. I told him that I am going to take you to the doctor to be circumcised.

**FACILITATOR**: Yes.

**PARTICIPANT:** The thing is that at the time when I was telling him about this, the teachers have already told them about circumcision at school. He already knew what was happening. He asked me whether he will go to the clinic and come back the same day. Whether he is going to heal etc. I told him that there are things that they are going to give them to you. I told him that they are going to give you some medicines that are going to help you heal. I even assured him that it is not going to take long for him to heal and that this is not going affect him when eating food and when playing.

**FACILITATOR**: Ok.

**PARTICIPANT:** I told him that after three days of circumcision he will go back to his normal life where he will be able to play like any other child. I assured him that his friends will not be able to see that he just comes from the circumcision procedure.

**FACILITATOR**: Ok. So how was his response when you were telling him this?

**PARTICIPANT:** He was happy to hear this but at the time when I say let’s go to the clinic for circumcision he looked nervous. He was not really sure of what is going to happen there.

**FACILITATOR**: Ok.

**PARTICIPANT:** That is what made him nervous.

**FACILITATOR**: Mm. Ok. So who told him about circumcision, yourself or his father?

**PARTICIPANT:** He was told by me.

**FACILITATOR**: Ok. So how did his father said about this?

**PARTICIPANT:** No he did not say anything. He just phone and asked that he heard that his son is going to be circumcised.

**FACILITATOR**: Ok.

**PARTICIPANT:** He just spoke to his son on the phone and assured him that he will come and see him when he comes back but he has not told me anything.

**FACILITATOR**: Ok.

**PARTICIPANT:** His father was also nervous. His fear was that his son was still young for circumcision but I told him that in medical circumcision we take him to the circumcision procedures when he is still young.

**FACILITATOR**: Ok. So why do you think it was important to tell him about the issue of circumcision?

**PARTICIPANT:** I was afraid that when he comes back he will ask me so many questions. So I thought it was better to explain these to him before he could go there.

**FACILITATOR**: Ok. But what initially came to your mind that you need to tell your son about circumcision and what to expect in circumcision. And also what made you make your mind or decide that your son should go to the circumcision?

**PARTICIPANT:** The thing is that I was trying to avoid the fact he does do away with this traditional circumcision.

**FACILITATOR**: Yaa. Ok, you were avoiding that he does not go anywhere.

**PARTICIPANT:** I told him about the way they do it in the traditional circumcision and that if he goes there are chances that he may not come back. I told him that with medical circumcision I am taking him to the doctor and that the operation is performed that same day and you come back home.

**FACILITATOR**: Mm. Ok. So how did his father interpret this because it was supposed to be him as the father to tell his son about circumcision issues and not the mother?

**PARTICIPANT:** He did not have any problem because he was always busy at work.

**FACILITATOR**: Ok.

**PARTICIPANT:** So as I have already planned this, I decided to speak to him about circumcision because it was the right time to do so. At that time I was still staying with him at home.

**FACILITATOR**: Ok you were still staying at home with him?

**PARTICIPANT:** Yes.

**FACILITATOR**: But do you think there is a difference between when a woman starts talking about circumcision and when a man starts talking about it? A man may be your child, your partner and a brother. Do you think it is different when it was started by a woman?

**PARTICIPANT:** I do not think is different. No.

**FACILITATOR**: Why do you say that there is no difference?

**PARTICIPANT:** Even if it was a man who started talking about it he was still going to explain the details to him the way I did as a woman.

**FACILITATOR**: Ok.

**PARTICIPANT:** Yes.

**FACILITATOR**: Ok, you think that he was going to explain the word you did?

**PARTICIPANT:** Yes.

**FACILITATOR**: But in people who are in a love relationship, who do you think should start talking about the issue of circumcision?
**PARTICIPANT:** Hey, in this case a man should start this conversation.

**FACILITATOR**: *Hehehe*, ok it is the man who should start the conversation?

**PARTICIPANT:** Yes.

**FACILITATOR**: What is the reason for that?

**PARTICIPANT:** I think it must be a man because in this case because the two are in a relationship and they are the concerned parties. They must start talking about it because circumcision is performed on them.

**FACILITATOR**: Mm. Ok, isn’t it that you said it is not a problem in a young boy?

**PARTICIPANT:** Yes it is not a problem in a young child.

**FACILITATOR**: In the relationship what would be the difference if a woman initiates such kind of a discussion regarding circumcision?

**PARTICIPANT:** In some cases men would like that some issues under discussion should always favour them. If that is not the case it would appear that you are undermining them. Especially with this issue of circumcision. If you start such discussion it is obvious that he will feel that you are undermining him. He might become suspicious that you someone somewhere who is already circumcised that is why you initiate such kind of a discussion. So it is better if he is the one to start the discussion. The other thing is that in some of the men, circumcision is not practiced in their religion so if you start talking about such a discussion it would appear that you are belittling him.

**FACILITATOR**: Ok, he will think that you have seen someone who is circumcised. You are undermining him. Mm. Ok. So you said that it might happen that at his home circumcision might not be practised because of his religion. What do you mean by that?

**PARTICIPANT:** I mean that where he comes from, at his family, they do not practice circumcision. But there are those that they do practice it whereas other don’t.

**FACILITATOR**: What could be the reason that some practice circumcision whereas other do not practice it?

**PARTICIPANT:** Let me give my own example, my husband is not circumcised. If I am going to say to him that let’s take this child do to circumcision what will you say if he would ask you whether your father is circumcised what would you say? He was never told about the practice of circumcision to start with.

**FACILITATOR**: Ok.

**PARTICIPANT:** And during his time there were not medical circumcision that are being practised nowadays.

**FACILITATOR**: Ok.

**PARTICIPANT:** There was no way in which he could have gone to the doctor to be circumcised because medical male circumcision was not practised then.

**FACILITATOR**: Mm. Ok at his home they did not discuss anything with him regarding circumcision?

**PARTICIPANT:** Yes they did not discuss anything.

**FACILITATOR**: Ok, perhaps they did not discuss it in his family or because of the fact that they did not discuss anything with him?

**PARTICIPANT:** I do not know because even his brothers are not circumcised.

**FACILITATOR**: Ok even his brothers are not circumcised?

**PARTICIPANT:** Yes.

**FACILITATOR**: Ok. So but does he have a problem when his son has gone for circumcision?
**PARTICIPANT:** No, he did not have any problem.

**FACILITATOR**: Ok, but given the fact that his son is still a family member and that at his family at home they do not practice circumcision, isn’t this a problem to him?

**PARTICIPANT:** No, this is not a problem. He does understand why his son should be circumcised. Before we could take him for circumcision we visit a doctor only to ask about the procedure in circumcision. He knew everything about the circumcision procedure before we could take him for operation. I do not think he will have a problem because everything was explained to him before.

**FACILITATOR**: Mm. Ok, so could you please explain to me about your experience when you were telling your husband about circumcision?

**PARTICIPANT:** When I was telling him?

**FACILITATOR**: Yes how did you tell him?

**PARTICIPANT:** When I questioned him?

**FACILITATOR**: That he must go for circumcision.

**PARTICIPANT:** I asked him why he went to the circumcision.

**FACILITATOR**: Yes!

**PARTICIPANT:** He told me that no one told him about circumcision at his home and he said that there was no way in which he could just go for circumcision on his own without the approval of the parents.

**FACILITATOR**: Yes!

**PARTICIPANT:** That is what he only said.

**FACILITATOR**: Ok, that is what he said?

**PARTICIPANT:** Yes.

**FACILITATOR**: Ok. So didn’t he misinterpret this as an act of undermining him or maybe you did try to explain to him in a very polite manner?

**PARTICIPANT:** The thing is that after answer in that fashion we never went deep into it.

**FACILITATOR**: Ok.

**PARTICIPANT:** I think if I went into detail about this he could have thought that I do undermine him that is why I was so careful in my words.

**FACILITATOR**: Mm.

**PARTICIPANT:** It is because we briefly spoke about it that is why it did not raise any problem.

**FACILITATOR**: So how difference it was to when you told a child to when you told a father about it?
**PARTICIPANT:** The child did not have any problem. I just thought that a child just think that everything that his parents tell him about is just right because his parents are guiding him in life. He did not have any problem.

**FACILITATOR**: Ok he did not have a problem?

**PARTICIPANT:** Yes.

**FACILITATOR**: Ok, what is your understanding about the fact that the family of your husband is not practising circumcision and that even your husband did not go for circumcision. Is your understanding that even your husband did not want to do it himself or is this because of his family religion?
**PARTICIPANT:** At his family they do not use traditional medicine that is why it was not possible for him to go to the traditional circumcision school because there they use traditional medicine.

**FACILITATOR**: Mm.

**PARTICIPANT:** The other reason is that where we come from the last traditional circumcision school was held back in 1982. Even when people wanted to take their children to the initiation school they were taking them to other villages. I think maybe this is what made him not to know more about circumcision.

**FACILITATOR**: Ok the initiation school is no longer held?

**PARTICIPANT:** Yes, it is no longer held.

**FACILITATOR**: What is the problem for that?

**PARTICIPANT:** They only held one the other year and this was because the chief was supposed to go and study at the university to do a course specialising in African leadership and that is why they held a special circumcision school for him. I really do not know the reason why is there no longer initiation school at home.

**FACILITATOR**: Ok. Ok. Do you a chief where you come from?

**PARTICIPANT:** Yes.

**FACILITATOR**: Ok. So is the chief playing a role in the issue of circumcision?

**PARTICIPANT:** Yes, initially the initiation school was held in the royal place. They were circumcised in the royal place.

**FACILITATOR**: Ok, they were going to the chief to be circumcised?

**PARTICIPANT:** Yes they were going to the chief.

**FACILITATOR**: Ok, were they staying at the royal place.

**PARTICIPANT:** Yes they were staying there.

**FACILITATOR**: Ok, they were doing all these things at the royal place?

**PARTICIPANT:** Yes, just there.

**FACILITATOR**: Ok. But how do you think a woman should tell a man about the issue of circumcision? The appropriate way in which a woman should use to tell a man about the issue of circumcision to an extent that such man would clearly understand the need for circumcision. How can she tell him about it?

**PARTICIPANT:** He can explain to him about the benefits and the importance of circumcision.

**FACILITATOR**: Ok.

**PARTICIPANT:** Yes. If I have enough information I will tell him about its importance and why it is necessary for him to go and circumcise.

**FACILITATOR**: You can tell him about its importance.

**PARTICIPANT:** Yes.

**FACILITATOR**: Like what? About its importance and the benefits?

**PARTICIPANT:** I would tell him that there are cancers that men suffers from and that circumcision may be able to prevent those cancers.

**FACILITATOR**: Ok.

**PARTICIPANT:** I would also tell him that if he is circumcised some of the diseases may not be powerful if it happens that he becomes infected.

**FACILITATOR**: Ok, those are the things that you will explain to him about?

**PARTICIPANT:** Yes.

**FACILITATOR**: Ok, and then what are the things that a woman should say to his husband when coming to the issue of circumcision? What is it that a wife should never say to his husband when discussing about circumcision?

**PARTICIPANT:** It is about sex.

**FACILITATOR**: Yaa.

**PARTICIPANT:** You cannot say to him that he must go for circumcision in order for you to be satisfied.

**FACILITATOR**: *Hehehe*! Ok, what do you think it could be a problem there?
**PARTICIPANT:** There might be many questions.

**FACILITATOR**: Yes.

**PARTICIPANT:** A husband may ask you what you mean when you say I must go for circumcision for satisfaction purposes. He would want to know where I experienced such satisfaction. Where do I see difference between a circumcised man and uncircumcised man? He may ask many questions?

**FACILITATOR**: Ok. So a woman should not say that?

**PARTICIPANT:** Yes she must not.

**FACILITATOR**: Ok. So do you think there is difference in that?

**PARTICIPANT:** No there is no difference.

**FACILITATOR**: Don’t you see any difference there?

**PARTICIPANT:** Yes.

**FACILITATOR**: Ok. But previously I heard you speaking about the issue of woman circumcision?

**PARTICIPANT:** Yes woman were going to the initiation school.

**FACILITATOR**: Is it?

**PARTICIPANT:** But I do not know how their circumcision ritual was performed. I do not know anything about their initiation. But they were going there.

**FACILITATOR**: Ok, they were also going to the traditional initiation school. So don’t you know anything about it?

**PARTICIPANT:** Yes, I do not know anything.

**FACILITATOR**: Don’t you know something about it?

**PARTICIPANT:** No I do not know anything?

**FACILITATOR**: Mm. But according to you do you think circumcision is a good thing?

**PARTICIPANT:** Yes, it is a good thing.

**FACILITATOR**: Mm. What makes you think it is a good thing?
**PARTICIPANT:** The thing is that we know why it is necessary for people to be circumcised.

**FACILITATOR**: Mm. You spoke about the issue of diseases, to prevent diseases, etc. Is there anything that you think circumcision is beneficial?

**PARTICIPANT:** I only think about the prevention of diseases.

**FACILITATOR**: Mm. Ok, but to people who are in love what do you think circumcision will benefit them? If there are some that you could add except circumcision? In people who are in a relationship what do you think will be the benefits of circumcision? To both a man and a wife. To people who are in love. What do you think are the benefits of circumcision?

**PARTICIPANT**: To both of them?

**FACILITATOR**: Yes.

**PARTICIPANT:** I do not understand what you mean?

**FACILITATOR**: To the people who are in a relation what do you think will be the benefits of circumcision? To both of them?
**PARTICIPANT:** Hey I do not know.

**FACILITATOR**: Don’t you know? Ok. Isn’t that you spoke about the issue of disease prevention, etc. Don’t you think about some other benefits to the couple in a relationship when a man is circumcised?

**PARTICIPANT:** To a man circumcision can help him to be strong. In some cases a man can be passive because he is not circumcised.

**FACILITATOR**: Ok.

**PARTICIPANT:** I think that circumcision boost him somewhere to be strong so that he does not experience early ejaculation.

**FACILITATOR**: Ok. What do you mean when you say he is passive?

**PARTICIPANT:** I do not know how to explain this.

**FACILITATOR**: The way you can?

**PARTICIPANT:** You know what?

**FACILITATOR**: Yes!

**PARTICIPANT:** No I do not know how to explain it?

**FACILITATOR**: Ok you say it boosts in early ejaculation?

**PARTICIPANT:** Yes.

**FACILITATOR**: How?

**PARTICIPANT:** That is, it helps. Hey, I do not know how to put it clearly.

**FACILITATOR**: No, come on. The way you can put it. Ok, there is not right or wrong answer.

**PARTICIPANT**: As I said that it helps him not to be passive.

**FACILITATOR**: I just want to understand what you mean by the word passive. I can misinterpret the word passive in that context. I want to understand what you mean by the word passive?

**PARTICIPANT:** That is, circumcision can make both involved in sex to be free. It makes a man to be free.

**FACILITATOR**: Ok.

**PARTICIPANT:** If he is not circumcised he is not free.

**FACILITATOR**: What does he do when he is not free?

**PARTICIPANT:** You will find that the person ejaculate early.

**FACILITATOR**: Ok.

**PARTICIPANT:** Let me put it that way.

**FACILITATOR**: Can we move on? Ok, don’t you want to say more about that?

**PARTICIPANT:** Do you want me to explain this way.

**FACILITATOR**: Ok, do you think when a man is circumcised this could help him not ejaculate early?

**PARTICIPANT:** Yes he must have power.

**FACILITATOR**: Ok, so as you have said your husband has not gone to the circumcision, so if he can decide one day that he wants to go for circumcision. How will you interpret such a decision? Or what will you think about him?

**PARTICIPANT:** I will support him.

**FACILITATOR**: Yes! So how will you take him for if he tells you that he has decided to for circumcision. Your interpretation about his decision to go for circumcision.

**PARTICIPANT:** The thing is that I will support him because I will see that he understand the benefits of circumcision and the other thing is that he will be having information on why he should go for circumcision.

**FACILITATOR**: Yes!

**PARTICIPANT:** The thing is that no one is forcing him there.

**FACILITATOR**: Mm, so you will support such a decision?

**PARTICIPANT:** Yes.

**FACILITATOR**: Mm. But do you think this will not change your attitudes towards her?

**PARTICIPANT:** No.

**FACILITATOR**: Ok. So why would you support him on that decision?

**PARTICIPANT:** I see this right decision.

**FACILITATOR**: Ok then the reason?

**PARTICIPANT:** I will not think anything of him. I know sometimes people are tempted especially if the wife is far from the husband. Men are usually vibey people, the like going to relax and have their beers. If I am not near to where he is staying at that moment, there are things that might happen. Then this is where circumcision could help him.

**FACILITATOR**: Ok. What do you refer to when you say things might happen?

**PARTICIPANT:** That is why I am saying that a man may be tempted because when he gets to the tavern he meets ladies and they may ultimately engage in sexual intercourse. Therefore this is where circumcision would help.

**FACILITATOR**: Yes. Ok you will support the idea of going to the circumcision because it would prevent some diseases.

**PARTICIPANT:** Yes, because he always go and relax with his friends at the tavern.

**FACILITATOR**: Ok, is he drinking the beer?

**PARTICIPANT:** Yes.

**FACILITATOR**: Ok. But then is there anything that you think we have not discussed on the issue of circumcision?

**PARTICIPANT:** No.

**FACILITATOR**: Don’t you think of anything. Ok. Previously you mentioned that you are a Mopedi by ethnicity, so in Sepedi culture how do you interpret the issue of circumcision?

**PARTICIPANT:** Nowadays we take our children to the medical circumcision at the clinics. As I have said, the last Sepedi initiation school back home was held in 1982.

**FACILITATOR**: Ok.

**PARTICIPANT:** I think nowadays the Pedi people prefer medical male circumcision.

**FACILITATOR**: Oo, ok. So what was happening is Pedi culture was that it was just a ritual that people should go for circumcision?

**PARTICIPANT:** But traditional male circumcision is still practised in some of the areas such as in Zebediela, Mashashane, etc.

**FACILITATOR**: Ok. There are some Pedi people who are still practising traditional male circumcision while some are no longer practising it?

**PARTICIPANT:** Yes some are no longer practising it. In GaMashashane they are still practising it.

**FACILITATOR**: Mm. Ok, not all Bapedi people practice circumcision?

**PARTICIPANT:** Yes.

**FACILITATOR**: What do you think might be the reason that some of the Bapedi people do no practice circumcision whereas some of them do practice it?

**PARTICIPANT:** I think some of them have realised that it is no longer safe as before. That is they are no longer sure that their children will be fine if they take them to the traditional male circumcision school.

**FACILITATOR**: Mm. Ok. So you think it differs according to people’s religion? Do you think it all depends whether people are Bapedi or the Zulus, etc. when it come to the issue of circumcision?

**PARTICIPANT:** I do not think they are different because they are doing one thing.

**FACILITATOR**: You do not see any difference? Ok. So how will someone’s tradition affect his decision about wanting to be circumcised?

**PARTICIPANT:** I do not think it will affect his decision.

**FACILITATOR**: Yes.

**PARTICIPANT:** Mm.

**FACILITATOR**: Ok, which ethnicity does your husband belong to?

**PARTICIPANT:** He is Mopedi.

**FACILITATOR**: Ok he is Mopedi?

**PARTICIPANT:** Yes.

**FACILITATOR**: So hasn’t the tradition influence his decisions and convictions about the issue of circumcision?

**PARTICIPANT:** No.

**FACILITATOR**: He just decided not to go?

**PARTICIPANT:** The fact is that he did not have information about circumcision. Let me say that he did not have information about circumcision ever since he was still younger.

**FACILITATOR**: Ok.

**PARTICIPANT:** If he wanted to go he would have asked his parents that why are other people going to the circumcision whereas he is not allowed to go. The thing is that nobody was going to the circumcision there.
