# Supplementary material for: “To speak or not to speak”: A qualitative analysis on the attitude and willingness of women to start conversations about voluntary medical male circumcision with their partners in a peri-urban area, South Africa
Source: PLoS One. 2019 Jan 25;14(1):e0210480. doi: 10.1371/journal.pone.0210480 (PMC6347244; doi:10.1371/journal.pone.0210480)
Supplement: S1 File — (ZIP) [file pone.0210480.s003.zip › QF010_QC2.docx]

**PARTCIPANT ID NUMBER:** QF010

**FACILITATOR**:  Thank you for coming here to participate in our project. Now I’m going to ask you to give me permission to record our conversation.

**PARTICIPANT:** Okay.

**FACILITATOR**: Do you give me the permission?

**PARTICIPANT**: Okay.

**FACILITATOR**: Okay, please tell me a little about yourself.

**PARTICIPANT**: Okay, I’m a mother of one. Who is married and has been saved (Christian)

**FACILITATOR**: Okay, so how old is your child?

**PARTICIPANT**: He’s five years old.

**FACILITATOR**: He’s five years old?

**PARTICIPANT**: Okay.

**FACILITATOR**: So, can you tell me what you know about male circumcision?

**PARTICIPANT**: Okay, what I know about male circumcision is that it’s...it’s...[inaudible: I don’t if I may explain it a bit in English]. It’s a surgical procedure in which they remove the upper part, the forehead (referring to foreskin) of a man and then they will stitch the remaining part. That’s how I know it. And then, it also prevent HIV transmission, STIs and it’s easier to notice the symptoms of HIV AND STIs.

**FACILITATOR**: Okay, I hear you saying upperpart. Where in the upper part are you referring to? Where do they remove it?

**PARTICIPANT**: They remove the forehead, the skin on top of a man’s penis.

**FACILITATOR**: Oh! They remove it from a man’s penis?

**PARTICIPANT**: Yes.

**FACILITATOR**: Okay. Can you tell me about where men go to when they undergo circumcision?

**PARTICIPANT**: From what I know, they do it at certain selected clinics as well as at some district hospitals.

**FACILITATOR**: Oh! So, male circumcision is performed in surgeries and in the districts..?

**PARTICIPANT**: Yes.

**FACILITATOR**: And then traditionally?

**PARTICIPANT**: Traditionally I don’t know.

**FACILITATOR**: But what do you know about the traditional one?

**PARTICIPANT**: Traditionally, I know that men go to the mountain and stay there for a month to get circumcised and then come back.

**FACILITATOR**: I hear you talking about the traditional one, saying here they...So with circumcision in the mountain where do they go and what do they do? Perhaps you might tell me what is it that they do there.

**PARTICIPANT**: When men go through circumcision traditionally, they are taught about cultural ways, about being a man and how to grow. But from what I know it’s too risky if you take a child to the mountain. It’s what I’ve heard.

**FACILITATOR**: Okay, can you clearly relate some of the stories you’ve heard to me?

**PARTICIPANT**: Okay, I once read a story about this other man from Kwa-Zulu Natal who once went to the mountain and got circumcised, and now as we speak he doesn’t have a penis at all. They had to rush him to the hospital to remove the penis and I don’t know all the details on how they removed it. And then another one was beaten, they beat him up since he didn’t know the circumcision laws and then his one hand had to be removed. It’s is very risky to take a child there and when he gets there he gets killed. These are things which are happening and they push us to keep a close eye on them.

**FACILITATOR**: And then what happens with the other circumcision?

**PARTICIPANT**: Okay. What I know about surgical circumcision is that you take a child there and then you’ll go back home with him to take care of him. And they’ll teach you. They’ll teach you on how to care for the child when you get there.

**FACILITATOR**: How long does it take when a person has went to get circumcised? You can tell me anything you understand.
**PARTICIPANT**: Okay. From what I know, when they perform the surgery, it takes about 30 minutes after they’ve explained to you.  It’s about 30 minutes to do surgery and after that…or six weeks for a person to recover. And then you aren’t supposed to have sex at all if you’re a grown up person...for about that six weeks because it’s risky. And then even masturbation, they aren’t supposed to masturbate at all.

**FACILITATOR**: So, I hear you talking about a surgery, so what are you referring to when you say surgery? What can you tell me about surgery?

**PARTICIPANT**: By surgery I mean when modern equipment are used for cutting them. That process they do using modern things is known as surgery.

**FACILITATOR**: Oh. Yes, so you mean medical circumcision?

**PARTICIPANT**: Yes.

**FACILITATOR**: Can you tell me if you’ve ever thought about telling a family member or a partner about circumcision?

**PARTICIPANT**: No, we’ve never talked about the medical one, we talked a lot about the mountain one….the traditional one. We talk about the risks, since I have a boy child I came to a decision that when he grows up I wouldn’t want to take him to the mountain. We also communicate as partners and say “my child here are the options”. As long as we talk as people about the consequences associated with circumcision that takes place in the mountain. We weigh the options.

**FACILITATOR**: So, how is it like talking to your partner? How does he take it since you’re a female person? How is it like to him?

**PARTICIPANT**: He doesn’t have much of a problem because he grew up in a community where they do it. When he was growing up, they did it traditionally. So, he never did the medical one. So, he knows all about the advantages of why you should do it. So as for my partner, I don’t think he has a problem about it at all. It’s the one which he encourages a lot when he considers how his children will be impacted afterwards.

**FACILITATOR**: So, you’re saying your partner went there?
**PARTICIPANT**: Yes.

**FACILITATOR**: What has changed now that makes him not to want to take his child? It’s like, he went to that place, so, doesn’t he ever think of taking him there?

**PARTICIPANT**: It’s like, the experience he got there, it’s not the kind that he’d like his child to go through. They beat them up, severely. It’s like they initiate them into becoming men, they beat them up severely.

**FACILITATOR**: Mm.

**PARTICIPANT**: So, he doesn’t want to see his child going through what he went through.

**FACILITATOR**: So, he’s not about to send his son to get circumcised in the mountains? Now, what is it that he’s seen or heard?

**PARTICIPANT**: They don’t normally talk about it a lot. The medical one?

**FACILITATOR**: Yes the medical one.

**PARTICIPANT**: He thinks the medical one is safe but knows nothing about it. But he perceives it as safe than taking a child to the mountains.

**FACILITATOR**: Mm. What does he consider safe about it since he knows nothing about it? What would make him think that it’s safe?

**PARTICIPANT**: Okay, firstly, it’s the fact that when a child is done you’ll sleep with them under the same roof and care for him. You’re able to see changes that might be happening to the child. And you’re able to get help quickly if you see something happening. It’s very important that the child comes back home with you when he’s done. You’re able to explain to him, to offer him support before he goes there and when he comes back from there…you’re able to offer support to him.

**FACILITATOR**: Okay, and then do you find it easy to talk to a male person about circumcision?

**PARTICIPANT**: Hai, I find it easy.

**FACILITATOR**: Okay, I hear you saying you’re able to talk to your partner. If it’s your brother or someone not related to you, how would talking to them about circumcision be like?

**PARTICIPANT**: To be honest I don’t regard it as such a burdensome thing.

**FACILITATOR**: Mm.

**PARTICIPANT**: It can be easy talking to him about it because at the end it helps his life.

**FACILITATOR**: But, how do you approach him? I hear you saying it’s easy, how do you initiate it? How do you approach a male person to talk to him about circumcision?

**PARTICIPANT**: Since there’s a lot out there about circumcision, it’s even advertised in the roads, so you can start in that way…on what they know about circumcision.

**FACILITATOR**: Mm.

**PARTICIPANT**: While he’s going...you’re able to approach him and give him information about how important circumcision is.

**FACILITATOR**: Okay. And the more he comes to you, you’re also able to ask him if he’s been through circumcision.

**PARTICIPANT**: Mm.

**FACILITATOR**: So, how do you first approach him? Like do you ask him if he underwent circumcision? Do you just start it like that? How should you approach him to talk about it?

**PARTICIPANT**: The way I’d start is by bringing up the topic so we may talk about it and I’d ask him if he knows about circumcision since they advertise about it a lot. The way he responds is the more I’ll tell him about...how it can bring hope.

**FACILITATOR**: Okay.

**PARTICIPANT**: If there’s a part he knows. If he doesn’t know...I’ll be able to inform him about it.

**FACILITATOR**: So, how can you give information to someone who wants to hear nothing about male circumcision? How can you give information to him?

**PARTICIPANT**: Okay, firstly, I’d inform him about the risks associated with not being circumcised.

**FACILITATOR**: Mm.

**PARTICIPANT**: The risks, everyone is scared of problems.

**FACILITATOR**: Mm.

**PARTICIPANT**: So I can begin by telling him about the risks. How dangerous it is not to be circumcised as a male person. And I’ll also tell him about the benefits you’ll get when you’ve circumcised.

**FACILITATOR**: Mm.

**PARTICIPANT**: About how important it is to get circumcised as a male person.

**FACILITATOR**: So can you tell me about those risks of not being circumcised? What are they?

**PARTICIPANT**: Uu! [laughs] I don’t know much about the risks of not being circumcised, but, it’s this thing which they call...which is on a male person’s private part. When it’s covered up in that way it can gather up filth and the filth can stay there for a long time. So, if it has been removed, it becomes easy for a male person to notice if there’s anything on it.

**FACILITATOR**: Okay, and then the benefits?

**PARTICIPANT**: The benefits are that no filth can gather up on it. You’ll be able to easily notice if there’s something happening to your private part.

**FACILITATOR**: Okay. Now, I hear you talking about risks. How does male circumcision benefit women? Can you tell me if you know what it is?

**PARTICIPANT**: Okay, for women I only know of one, one about the bedroom. When you’re busy in the bedroom, that thing of a man keeps on going back and forth. It’s disturbing, it’s much nicer when that thing isn’t there. When it’s there it’s too disturbing.

**FACILITATOR**: So, do you mean to say that sex becomes more enjoyable when that foreskin isn’t there?

**PARTICIPANT**: Yes.

**FACILITATOR**: So, those are the risks?

**PARTICIPANT**: Those are the benefits.

**FACILITATOR**: Okay, those are the benefits.

**PARTICIPANT**: Yes.

**FACILITATOR**: So, what are the risks that affect women when her partner is uncircumcised?

**PARTICIPANT**: I don’t know. For women?

**FACILITATOR**: Or when men have been circumcised, how does it help women?

**PARTICIPANT**: I don’t know. [Laughs]

**FACILITATOR**: Okay. So if you want to talk to a man about circumcision, what are you not supposed to say? What must you avoid so that a man can undergo circumcision? What words are you not allowed to say?

**PARTICIPANT**: Okay, the words you’re not allowed to say are words like the pain involved there, hey pain, it takes time to recover. You must avoid such words. You will scare him off. You must make sure that you avoid words that will scare him off. You must use words of encouragement.

**FACILITATOR**: Mm. So, you’re saying that to encourage a man you mustn’t tell him about what happens. Or you shouldn’t tell him about the pain.

**0PARTICIPANT**: Tell him about what is going to happen but in a way that won’t scare him off.

**FACILITATOR**: Mm.

**PARTICIPANT**: You can use them but use them in way that is worth it in the end.

**FACILITATOR**: Mm. So you show him that after he has been circumcised, what’s going to happen next?

**PARTICIPANT**: The importance of circumcision than the pain, the processes that are going to happen after that.

**FACILITATOR**: Okay, do you think that male circumcision is a good idea? Is that what you think?

**PARTICIPANT**: Yes.

**FACILITATOR**: What makes you think that?

**PARTICIPANT**: As I’ve already mentioned, the other risks, the longer that man’s thing...that skin... it’s very dangerous because bacteria breeds there.

**FACILITATOR**: Ok. So tell me, when people are in a relationship, who is supposed to bring up the topic on circumcision?

**PARTICIPANT**: I think it’s the woman.

**FACILITATOR**: Who do you think should bring up the issue of circumcision between the two couples?

**PARTICIPANT**: Usually I encourage women to start the conversation. So if it’s a woman who initiates the chat, the man gets encouraged. He will be encouraged as he will see that “hey, my wife supports the idea of me doing this…”

**FACILITATOR**: So, you think that men will feel comfortable with women starting the conversation about circumcision?

**PARTICIPANT**: Yes.

**FACILITATOR**: What would make it easy?

**PARTICIPANT**: Because the man could be too scared to start the conversation about circumcision with the woman even if he wanted to. He would think about how his partner would handle such a topic. When the woman starts the conversation, and she expresses her views, the man will find it easier to ask questions and fully participate in the chat.

**FACILITATOR**: So what do you think makes men feel that women won’t be comfortable with this conversation?

**PARTICIPANT**: It can be many things. Perhaps, it could be that the wife is not enjoyable in the bedroom. Even if the woman is the one who initiates the chat, even if it’s having to satisfy the woman, it’s as if the woman isn’t enjoyable or something. It’ll be like he doesn’t take care of her very well. So, if she’s the one who initiates it, it could encourage the man a lot...for him to get circumcised so that they might enjoy a great sex life.

**FACILITATOR**: Another reason?

**PARTICIPANT**: What I mean is that, if the woman starts the conversation, the man will find it easier to participate.

**FACILITATOR**: Mm. Won’t it cause the man to think that there’s a problem?

**PARTICIPANT**: Even if he thinks there is a problem, ultimately he will wonder why his partner is suddenly interested in this topic. He would feel awkward that his partner started the conversation , but even though she started it, his confidence will be boosted.

**FACILITATOR**: So, if it was the man who started the conversation how would it turn out?

**PARTICIPANT**: I don’t think that’s much of a problem. Uu, he, I don’t think it could be a problem.

**FACILITATOR**: You don’t think there is a problem? Okay. So, how would lovers benefit from circumcision?

**PARTICIPANT**: This is what we were talking about when we mentioned the benefits, that when the upper part is removed, sex becomes more enjoyable than when it’s not removed.

**FACILITATOR**: So you can’t make any additions to that one, are there no other benefits that you are aware of?

**PARTICIPANT**: I don’t know of any others.

**FACILITATOR**: What would a woman think, about a man who decides to undergo circumcision? Would she have a change of heart or will her attitudes towards him change?

**PARTICIPANT**: Well, as people we have different ways of thinking. I would be under the impression that he wouldn’t get circumcised on a whim. We have to talk about it first. It’s very important that men should talk about it before they get circumcised.

**FACILITATOR**: So in your opinion, what are the thoughts that a woman has when her partner undergoes circumcision?

**PARTICIPANT**: The woman would ask herself many question, such as “why now?” and “why didn’t he get circumcised when he was younger?”

**FACILITATOR**: What should a woman do when her partner doesn’t want to get circumcised, in what ways can she approach the conversation? What can she do to inform him about the topic without asking him?

**PARTICIPANT**: Without talking about it?

**FACILITATOR**: Yes.

**PARTICIPANT**: What needs to happen is that women have to show more interest in these things about circumcision. She can use photos if she chooses. She can use these to show him that this is how she prefers to be safe. These pictures can be hung on the walls in the house, and saved on her phone. By doing this she is making him ask himself, “why is she interested in such things?" She could have CDs or something that she can play or watch in his presence. She could also surf the internet on the topic, and when he asks she can just say that she wants more information about it. This will attract her partner’s attention.

**FACILITATOR**: Mm.

**PARTICIPANT**: Mm. You could hang a big framed picture on the wall, and then every day when your partner comes back from work he’ll see it and notice that you like it. Then you’ll say that you like it and he shouldn’t remove it. Even when he is alone, he’ll ask himself as to why you’re interested in this thing. He’ll start discussing the importance of circumcision.

**FACILITATOR**: I remember you mentioning that your husband underwent traditional circumcision, right?

**PARTICIPANT**: Mm.

**FACILITATOR**: I just wanted to know, since your husband underwent circumcision at the mountains, how will your decision to have your son undergo medical circumcision affect his culture?

**PARTICIPANT**: He is not affected badly by this, he actually supports it a lot because he has first-hand experience of what happens at the mountains, and as a result he doesn’t want it to happen to his child.

**FACILITATOR**: Mm.

**PARTICIPANT**: And I told you about that story that I read, about that man who had lost his manhood. He lost it because of the things that happened at the mountains. The other one doesn’t have a hand. The other reason is that your child can die and never return home. That isn’t something that a parent would want for their child. This is why he supports the idea of medical circumcision.

**FACILITATOR**: Mm. But is there no way that culture is affected by medical circumcision? Do you think we can convince people to choose one and not the other?

**PARTICIPANT**: Traditionally it would seem bad as the Bapedi are very proud of their culture, and it could kill it. They could think that western culture will end up killing their culture.

**FACILITATOR**: Mm.

**PARTICIPANT**: So to ensure it doesn’t cause too many deaths, they must educate traditional leaders about what the process of circumcision is like. About this thing, about children returning with missing parts, injured because they have been beaten or dead. And this will help other people to take their children there because nowadays everybody is scared to send their children.

**FACILITATOR**: A lot of people are starting to take their children for medical circumcision. Okay, for things to change should the people who work in traditional circumcision be taken to the medical one?

**PARTICIPANT**: Yes. To teach them as well. They don’t know anything about medical circumcision.

**FACILITATOR**: Yes.

**PARTICIPANT**: So, the only option that they have is traditional circumcision. When the child returns with a problem...these type of things happen. Especially in the rural areas, where it takes time to get to the clinic

**FACILITATOR**: I hear you say that people don’t know about circumcision. How come you know these things about circumcision?

**PARTICIPANT**: The medical one or the traditional one?

**FACILITATOR**: I am talking about circumcision.

**PARTICIPANT**: Okay, how I found out about circumcision. I knew of it when I was growing up. It’s routine, it’s something that happens every year, in June they send children of a kind to undergo circumcision to the mountains. So that’s how I knew that they had to undergo initiation to get circumcised. I knew about the medical one when I moved to Gauteng. While I have been living here I heard people talking about it and I started knowing about it. But the traditional one, I have known about since I was growing up.

**FACILITATOR**: Okay, how did you know about this one?
**PARTICIPANT**: I knew about it when I came to the clinic. I came to the clinic and I heard them talking about it, they said that there is medical circumcision and I thought that I would like to know about it because I have a son. I started to google it.

**FACILITATOR**: Mm.

**PARTICIPANT**: Yes. I want to know more about it and how it works. That’s how I got information on it... somebody told us that children between the ages of 12 and 15 years should be registered for circumcision.

**FACILITATOR**: So in your opinion, how can we reach out to people or what are the other ways in which we can reach those people who don’t know about circumcision?

**PARTICIPANT**: Especially in the rural areas I think that there should be campaigns, every month if it’s possible or they should go door to door informing people about circumcision

**FACILITATOR**: Mm.

**PARTICIPANT**: The dangers of the one done at the mountain. They should inform people about it so that they can weigh their options. They must be available all over.

**FACILITATOR**: So people must be told about circumcision. What other ways can they do it without going door to door?

**PARTICIPANT**: Apart from going door to door they can target schools. There should be meetings especially for children. There should be meetings at schools for parents in which they are informed about circumcision. Days should be set aside, one or two days in which they can try to inform parents about circumcision.

**FACILITATOR**: Okay.

**PARTICIPANT**: They must also be knowledgeable of the fact that there is medical circumcision as well as traditional circumcision.

**FACILITATOR**: Mm.

**PARTICIPANT**: Even on TV they can show it because people like such things. Even if there could be a programme or show that they can slot in so that they can inform people about circumcision, and how traditional and medical circumcision work.

**FACILITATOR**: Yes.

**PARTICIPANT**: Explain to them that it could take up to a month. Maybe, let’s say they undergo circumcision in June, they will do it in April so that awareness about circumcision can reach the people because, in the rural areas people have TVs they can hear it on their TVs and their radios.

**FACILITATOR**: Okay. So at home, in your opinion, who is supposed to start the conversation about circumcision between the mother and the father, who’s supposed to start talking about... start the conversation about with the children?

**PARTICIPANT**: Okay, at home the conversation should be started by the mother and the father because they’ll be discussing it between themselves, but when the matter goes to the children, it should be started by the father because they are all men. Yes they are all men, I think that the child will feel comfortable. He understands what he is talking about . And then he will share some of his experiences. He’ll share the advantages and the disadvantages and answer the questions that the child will ask. Other things a mother won’t know.

**FACILITATOR**: Mm.

**PARTICIPANT**: In some instances the father will give the child options between traditional circumcision and medical circumcision. The father has all the answers. The father has undergone the traditional one and then, he can educate himself about the medical one. So if the child says that he wants to undergo traditional circumcision. He’ll be able to give him answers. He can also give him answers about medical circumcision

**FACILITATOR**: Now, maybe the father hasn’t undergone circumcision, let’s say he will tell him about the things that he has come across. Do you think that it will be easy for him to tell his children to go, or what?

**PARTICIPANT**: To tell you the truth, it won’t be easy.

**FACILITATOR**: Mm.

**PARTICIPANT**: The child will ask, “have you undergone circumcision?” The child can also sense if the father is talking about something he hasn’t experienced. So the father has to be the one who goes first so that he can explain to his son that, he has gone and in his opinion the importance of circumcision is this. I have also been in the stage that you are in. You must know what the difference is if you do get circumcised and when you don’t. I think it is important that the father goes first.

**FACILITATOR**: Okay, if it was the mother who talked to the children? In your opinion, what makes children decide to go or to not go?

**PARTICIPANT**: I know that there are women who don’t have husbands at home. They have to take on the role of the father.

**FACILITATOR**: Mm.

**PARTICIPANT**: So the best thing to do is to try your best at informing your child about circumcision and to find more information about it. If you feel that you are unsuccessful, find a man who is clued up on it. He will explain it to you so that you can explain it to your children.

**FACILITATOR**: Yes, about circumcision or anything else. Okay, now if it’s the child who starts the conversation about circumcision before the parents start talking about, how will the atmosphere be like at home?

**PARTICIPANT**: Ok, both parents are there right? The mother and the father? And they haven’t spoken about it yet?

**FACILITATOR**: Yes.

**PARTICIPANT**: I think that’s good as well. It could be awkward if the child starts talking about it, if you’re a responsible parent, take steps and make sure that you provide information for your child. Look for more information on it. If you don’t have any answers for your child tell him that you’ll find them for him. If you have the answers, give your child the answers.

**FACILITATOR**: Okay, thank you it’s time to finish off our interview. But before we close, is there anything that you would like discuss that you feel I haven’t mentioned or that I’ve overlooked? Or that you think is important that we should talk about?

**PARTICIPANT:**  About circumcision?

**PARTICIPANT**: Okay, it’s about when children are still small, I have heard people say children of the age of two or three years, are you able to have them undergo medical circumcision? So I understand that even a 18 month old child can be circumcised early. Or is there a specific age that a child should get circumcised.

**FACILITATOR**: I don’t know about the age but I have heard that they go. But here in our clinic we start at the age of 10 years and above.

**PARTICIPANT**: Okay. Another thing is, we discussed the benefits earlier, I only knew one even if I am not sure whether it’s right. Can you tell me more about the benefits of getting circumcised? Especially for women, that as a woman what would do I gain when my partner is circumcised?
