# Supplementary material for: “To speak or not to speak”: A qualitative analysis on the attitude and willingness of women to start conversations about voluntary medical male circumcision with their partners in a peri-urban area, South Africa
Source: PLoS One. 2019 Jan 25;14(1):e0210480. doi: 10.1371/journal.pone.0210480 (PMC6347244; doi:10.1371/journal.pone.0210480)
Supplement: S1 File — (ZIP) [file pone.0210480.s003.zip › QF012_QC2.docx]

Participant ID (P): QF 012

RA: Mmm do you agree that I record you with an audio recorder when we do our interview?

P: Yes I agree

RA: whoah okay eh can we please talk now about your knowledge and the things you think about medical circumcision *neh*, what do you understand or what is your understanding about medical circumcision?

P: In my view is that medical circumcision is something that is right and important that they do it because you must be healthy in my understanding you must cut the foreskin and be healthy medically so that other diseases do not catch you.

RA: Oh okay

P: Mmm

RA: So medical circumcision what does it mean?

P: Medical circumcision in my view it is something that is right

RA: Mmm

P: Eh

RA: Oh alright uhm…can you tell me about the types of circumcision that you know, how many are they?

P: Mmm…

RA: The types of medical circumcision

P: *He!* I don’t want to tell you, I do not know them

RA: You do not know the types? Okay what other type of medical circumcision I mean what other type of circumcision do you know beside this medical one?

P: *Hha!* Me I know the medical because people always talk and say there is one where you go to the mountain

RA: Mmm

P: Eh

RA: What knowledge do you have about the one of going to the mountain?

P: Mmm the knowledge about it I do not have

RA: You do not have the knowledge of it?

P: Eh

RA: Oh okay uhm…have you thought about telling the person you involved with or a person in the family uhm about medical circumcision?

P: Eh I thought about it, the child in our house is the person we told and he went and registered at the clinic that he must go it is important for his lie

RA: Oh okay

P: And that he must know himself and his status so that many sicknesses will not enter him

RA: [Coughing] you say so that many sicknesses will not enter him?

P: Enter him because there are many sicknesses that can enter him

RA: What kind of sickness? And how can it enter him?

P: Like HIV like there are many just dirt that stays on him

RA: Hmm

P: Many

RA: Okay

P: Mmh

RA: What made you tell the child that he must come medically circumcise? What caused you to tell him?

P: It is because children of today do wrong things

RA: Mhm

P: So I saw well that it is better that we advise him while he is 18 to go and see the doctor

RA: How old?

P: 18

RA: 18?

P: Eh

RA: Okay what does it mean that he comes when he is 18?

P: It is just that we see that the time is now right because he must know now that in life the thing that is wrong and right. It is the time he must go to the doctors before he gets sicknesses

RA: Which is the right time to come and medically circumcise?

P: In my view is that from 16 that is right to go to the doctor

RA: why do you say that from 16 it is right?

P: Cause in these times children of today they do wrong things and we do not go with them they do different things. They travel you find a child was up to no good and did not talk then he starts getting sick he does not say what he was doing there outside

RA: Okay you say in these times children do wrong things, what are these things that are wrong which children do?

P: Children travel you find that a child is going to sleep outside and not talking with his parents and want maybe they are influenced by friends they go where ever tomorrow he wakes up and says he was sleeping with a person without knowing you find that the person is sick and did not tell him.

RA: Mhm alright now ehm what could it mean if a male suggests that he will do circumcision to you could it make a difference if this thing is said by a female? Or it will not make a difference?

P: It will make a difference because a female will make him understand, she will try to talk with him and explain if can make a big difference if it is done by a female

RA: What is the difference?

P: Talk with people like to explain to them ya, sit down with them and talk with the and explain to them that it is like this and that maybe that go to the clinic it is important that you circumcise so that your lives will remain right, it is important that they go so that even diseases do not enter them

RA: Mmm so you say it can make a difference if a female says it? Why do you say that?

P: Because a female is able to talk with a male, she explains. It is important that it is done by females

RA: Mhm you say a female is able to talk how? She is able to talk how? Or a female can say it in which way to a male this issue?

P: [six seconds silence] she can be able to sit with him and explain to him

RA: Mmm

P: Eh sit down and explain the situation, how important is it that he does circumcision

RA: Okay alright mmm…so it can make a difference if a female says it?

P: Eh it can make a difference

RA: If a female says it?

P: Female eh

RA: In a relationship?

P: In a relationship yes

RA: If a male says it to a female how is it like that?

P: Even there, there is no problem but a female it’s gonna be more different

RA: Mmm

P: Eh

RA: What will make it to be different?

P: Cause a male will not maybe another explain enough and a woman will be able to talk with you and understand, she will be patient with you to explain that if you have gone there what must you do.

RA: Okay what ways a person like you can bring this topic of male circumcision to a partner or a member of the family? Any other way you thinking just any way that you thinking of

P: To explain to my family?

RA: Eh

P: Me I would explain to my family about this one that…it’s a good thing to go and circumcise. It is important to a family to a family so that we can be protected from many things and it is important that we must always know in what status are we in. We must know that we are sick or we are not sick so we can know that we are safe at all times.

RA: Okay

P: Mmm

RA: Now you say you will tell them that it must be done to always know whether we are sick or we are not sick

P: Mmm

RA: Medical circumcision makes you know that your are sick or not sick?

P: Cause obvious if you are going to go to the doctor they will start by testing you before, they will ask you and test you and then tell you

RA: Mm okay…alright uhm what approach uhm or way a person like you must avoid when they tell their partner or family member that they must go and medically circumcisie?

P: [nine seconds] mhm

RA: Which approach or way you must avoid as a female when you raise the issue that a male must come and medically circumcise what must you not say when you tell them?

P: The thing you must not the thing you must avoid maybe the first thing you must not have a bad attitude towards a person

RA: Okay

P: You must always be patient for him and talk properly and avoid just avoid just talking wrong things to him or avoid just that even if he asks you something and you become rough for him you must just have a soft touch and make him understand.

RA: What are they? Which is a bad attitude?

P: To those, you find that when you talk to a person you want to force them. When they ask you questions you don’t talk to him in a good manner and now you must be patient and make him understand you must not have that thing. When you are a person you must get used to not having a bad attitude, be humble to people all the time.

RA: Mhm

P: Eh

RA: When you are humble how do you become? How do you say this thing, how do you put it in a humble way?

P: You must be patient, and understand a person when they speak and you too understand him and listen carefully.

RA: Mhm

P: The thing they are speaking,

RA: When you listen carefully to a person what do you do?

P: They are talking and you are listening when you do not understand you ask them that “no here I do not understand what you say can you please repeat it for me so that I can understand” if you do that then you are listening

RA: Okay. Alright then and then you said again a female must have a soft touch you even used that word. What does it mean to have a soft touch?

P: It means you treat a person well, when you talk to them you listen to yourself and do not have noise towards them

RA: Mhm

P: Eh

RA: You listening to yourself how?

P: You talk to them properly after all there are some people you find that when you talk to a person and they want to ask a question you know them “No I said this and that and explained like this” no you find that maybe other men maybe want to circumcise they are old they will want explanations and you must explain that baba this is what is done and again even if they ask you questions you must listen carefully and explain to them and be patient with them, do not be harsh for them.

RA: Okay

P: Ya

RA: Can you give me an example now talk as if you were talking to a male to come and circumcise medically how are you supposed to put it this thing? Just make an example just make a picture from your mind on how you would start

P: [Giggling]

RA: Mhm isn’t here is a person who is a man is present here you must tell him how are you going to say it to him?

P: Okay you start by greeting him [giggling]

RA: Mmm

P: “Greetings baba, how are you, we are good ehm…who [not clear]”

RA: Ya isn’t you have already said you must talk with him properly and listen carefully to him I want to see this thing that a woman must do

P: [Sigh] you greet them first “Greetings baba, how are you? Uhm here there is a doctor who cuts the foreskin. Uhm we were asking baba if you would like that they cut your foreskin as well.”

RA: Mhm

P: So cause another will ask you “Why do you say if we like?”

RA: Mhm

P: Uhm you explain to him that “Okay baba to cut the foreskin is something that is very important cause diseases that are there enter in different ways and they are many baba it can happen that if you go to the doctor he will help you with those diseases

RA: Okay

P: Eh

RA: It is the way a female must go with to a person who is a man in raising this issue of medical circumcision?

P: Eh

RA: Oh okay alright then. If you have not mentioned, if you have not said it at all can you please tell me the things you experienced the time you were trying to raise this issue of medical circumcision to the male family member or a male you are involved with

P: The thing I experienced with my family member yo there are many. When he got back from hospital like cause the first thing he explained to him about his status that he is alright he is negative and I saw him like he was happy seeing that it is really safe to go to the doctor it is something very important and he was alright he did not have like any sickness that there is something that is bothering him like because he did something wrong no he was safe and relaxed.

RA: Oh okay so maybe you can tell me again the experience you had when you tried to tell a male maybe your partner or a family member about this issue that eh here is male circumcision it is done like this and this. Have you ever done that besides to your brother? Another person maybe maybe your partner or a person-

P: No

RA: Or or your friend?

P: No I have never done it

RA: You have never done it?

P: Hmm mhm.

RA: Oh okay, the one with your family member how did it happen?

P: Whoah it happened there was- we went to the clinic we had the flu so there was another woman who had pamphlets

RA: Mmm

P: Saying that “Mothers I kindly request whoever would like to register their child there is circumcision at the {} (name of clinic), I will give you pamphlets and you will go to the clinic and you will get explanations on how”. We took the child of the house in two weeks’ time he went with my mother and they arrived and registered, they gave him a date when he should return again here at the clinic

RA: Oh okay

P: Eh

RA: So he returned again?

P: He returned again to the clinic

RA: Oh okay

P: Mhm

RA: So how was- how was it coming here and him returning here, was he alright? How was he?

P: He was alright; you know there was nothing wrong that happened he was safe just he was alright

RA: Oh okay

P: Mhm

RA: And then how did you feel that he came?

P: We felt happy cause he came to the doctor and it’s a good thing that you stay when you come to the clinic. In my view it is safe to do circumcision at the clinic because you are safe it is the right place.

RA: Oh okay why do you say the clinic is the right place?

P: You find maybe that you went to the mountain; they will not check you find that maybe you are HIV, how will they know? So at the clinic it is better that when you go and do it they first test you to know how you are

RA: Mhm

P: Eh

RA: Mhm alright, so that they know how you are, where does that help?

P: They first test you first

RA: Mhm

P: Eh and then they will tell you you’re results when you return

RA: Mhm

P: Eh

RA: How does it help that you first tested first?

P: Eyi in my life you know what sister you must indeed test it helps that at the clinic you get there and test

RA: Oh okay, now you think that circumcision is the right idea or wrong? It is not the right idea?

P: Mm. To circumcise is the right idea so that males can remain safe in the world

RA: What do you mean when you say males?

P: They will be protected from many diseases

RA: Okay what kind of diseases?

P: STD, HIV

RA: Okay

P: Mhm

RA: What do you think are the benefits of circumcision for a couple or two people that are involved?

P: The benefits of circumcision for a couple in my view is that one must go so that we can know in a relationship if one is wrong I will also be able to go to the hospital to check myself

RA: Mmm.

P: You find that maybe the man has HIV I will also be able to go , so that we will be able to remain together and know that how safe we are.

RA: Know what?

P: That we are safe with our diseases, it can happen that you find that one has it and another does not have it the man must know also that he must take his treatment and stay right. In my view it is something beautiful that a person goes to the clinic and circumcise and they test him, it’s a good thing.

RA: So the benefit of circumcision is what? For a couple?

P: For a couple is to know yourself where you are and that which sickness you have. That’s a benefit to remain knowing themselves that which disease do they have?

RA: Oh okay, if you come circumcise you will just know what disease you have?

P: No well the doctor will ask you, they will ask you and then they test you. There are many diseases even if you do not have HIV there are many things that could be there. They will test you maybe you find that you have high blood or maybe you have sugar diabetes. They are gonna test you then they do it

RA: Oh okay

P: Mhm.

RA: Alright eh now between two people that are involved who must raise the issue of circumcision?

P: It is the woman, she is the one who must raise it and be able to tell her partner

RA: The woman?

P: Eh telling the male

RA: Oh can we talk in detail about that, why do you say a female must talk about it?

P: Like tell a person and explain to her partner and tell them that they must go and it is something right to circumcise and it is more safe at the hospital

RA: Mhm

P: Eh

RA: Why must it be a female to raise this issue and not a male?

P: because she stays with him. It is important that she tells him because it is the person she is used to communicate with and talk with him eh

RA: Alright so if it is not the person like you say it is the person she is used to talk to so it is a person she is not used to talk with, will she not be able to talk about this issue?

P: Another he will not listen to, he will tell himself that “Ah he is just telling me” so a female that stays with him will explain to him and sit him down and explain to him.

RA: Mmm

P: Eh

RA: So you say another he will not to?

P: Eh

RA: Which one is that?

P: You find that it is a male from the street telling him to go and circumcise he will tell him that “No I will go” so a woman will explain to him not that she is forcing him she must tell him how important it is to go to the hospital to circumcise. He will listen and then go

RA: Mmm, the woman you are talking about is it a woman he is involved with or it can be a woman who is his relative?-

P: His relative-

RA: Or family

P: He will understand eh he will understand even if you are involved with him he will listen to you

RA: Mmm

P: But if you are an outsider he will not take you serious he will say okay and leave it but if you are his relative he will understand that he must go to the clinic

RA: Mmm

P: Eh

RA: So this person he is involved with or his relative how must they say it?

P: Eh eh

RA: To him when they tell him how much they say it?

P: They must first explain that “Brother there is a clinic they circumcise there at {} (name of clinic) can we please go brother and hear what they will explain to you how to start”

RA: Mmm

P: Then he will wake up in the morning then we go

RA: Okay eh…the mind of a man in him wanting to circumcise medically how could it be to a female if a male comes to a female saying “I want to go medically circumcise” would the female be in favor of this thing the male is saying or she won’t be in favor or she could be neutral to the thing he is saying?

P: Mmm…she could be in favor the female

RA: Why do you say that?

P: It’s because females understand too much, they understand too much so that’s why I say maybe she can be in favor

RA: What things can she do that show that she is in favor?

P: Maybe she can try and help him and go with him to the clinic

RA: Okay

P: Yes

RA: Why why will a female try and help him in that way to go with him to the clinic?

P: Isnt he wants help that she must come with him to the doctor so that maybe he will want to understand as well why a male comes to the clinic for circumcision maybe she will need explanations as well.

RA: Mmm

P: She will want to understand

RA: Whoah to come with him when he comes here to the clinic is there a difference it makes?

P: There is a huge difference because she has come with him, you get knowledge you didn’t know of

RA: Mmm

P: A huge difference

RA: Okay to a male is there a difference that it makes that as a female you brought him here to the clinic?

P: Eh him as well there is a huge one, you see there are some people who care about me in my life because here she is coming with me

RA: Alright…okay mmm ehm mmm now is the time that we go to part- to activity number two, do you remember as I was explaining that we have three activities?

P: Mmm

RA: Now we are finishing our first activity and we are going to enter to the second activity but before we go to the second activity can I please ask you if there is anything you feel that is important that we did not talk about which you wish to add to this thing we are talking about?

P: No there is none

RA: Okay thank you sister we are now going to our activity two, I will explain to you how it goes isn’t?

P: Okay

RA: Alright

[Audio paused]
