# Supplementary material for: “To speak or not to speak”: A qualitative analysis on the attitude and willingness of women to start conversations about voluntary medical male circumcision with their partners in a peri-urban area, South Africa
Source: PLoS One. 2019 Jan 25;14(1):e0210480. doi: 10.1371/journal.pone.0210480 (PMC6347244; doi:10.1371/journal.pone.0210480)
Supplement: S1 File — (ZIP) [file pone.0210480.s003.zip › QF014_QC2.docx]

Participant ID (P): QF014

RA: Will you allow me to audio record the interview?

P: Yes I allow you to audio record the interview

RA: Okay thank you for participating in our study. May you please tell me as a person who’s working with community; tell me the different types of people that you are working with are you working with men or women?

P: Okay as I’m working with community…okay as I’m working with community I working with men and women and working with children as well

RA: Okay actually in the community what are doing?

P: Okay in the community what are doing we teach the community especially (sneezes)…sorry in the community we teach people about HIV, diabetic and other diseases we teach them but mostly we based on teaching them about HIV cause we want to see this diseases being reduced too much

RA: Okay what are the challenges that you are facing in the community?

P: The challenges that we are facing is that people are in denial too much. Even if we’ve been preaching and saying this diseases is there and leaving, we trying to help them to use protection. But most of the time people are in denial even if a person is trying to, sometimes you can see the symptoms and try to explain to a person and a person will tell you about the ancestors and go back in the olden days, culture

RA: Alright and then to work with men, because I had that you also work with men and children. How is it to work with men?

P: Men in most cases they don’t go to the clinic. As I said men they don’t go tom the clinic it means they don’t go to test but there are men who goes. When you motivate a person and say no sister I’m going to the clinic tomorrow. Others they don’t want they will tell you that my wife is pregnant , And she tested she’s HIV negative it means I’m also HIV negative. So that is the most challenge for men they don’t want to go the clinic

RA: Alright okay may you please tell me about your understanding male circumcision?

P: Okay my understanding about male circumcision since you working with community and I’m working about heath related issues, I can say in the beginning I wasn’t having a problem whether a person is circumcised or not circumcised. But male circumcision it’s not 100% but it can reduce a person not get HIV and STI’s anything that is related with sexually.

RA: Okay alright what are the different types of male circumcision that you know?

P: The different types that I know for male circumcisions are Sotho’s, Xhosa’s Venda’s and Ndebele’s.

RA: Okay what types of circumcision that they are using?

P: Okay in most cases I can they use the mountain. Cause in most cases we believe in in different things but in most cases they follow the traditional way.

RA: Okay can you tell me about your understanding about traditional circumcision?

P: Oh my understanding with traditional circumcision in nowadays I can say traditional circumcision is not safe because in most cases when you look even on TV people are dying. Others, others, they don’t cut it means when they say they are cutting them they are not cutting them well. They don’t check them may be one them and others came up HIV positive. Like last I don’t know if I’m allowed to talk in cutting edge I was watching their episode, I think its three weeks back a person was talking saying there others they are beating them. Where I was shocked and see how dangerous it is to circumcise in the mountain one of them said he doesn’t have his manhood, his manhood became so hard in an unusually way and fell he doesn’t know how. He said he used to have girlfriends and his girlfriend used to visit him cause they had the rumours that he doesn’t have his manhood. I was so shocked that going to the mountain they cost your life.

RA: So people who came to visit and heard that he doesn’t have his manhood so they came to make sure that he doesn’t have his manhood?

P: Yes just to make sure, it’s because it’s something that she had it from someone else and now it’s my boyfriend so I can see since he came from the mountain do you still have your manhood

RA: So was he able to explain the situation after his people came to see that reality that it is true.

P:Him by the time he explain he said he feel …the way he explain that he said he was going to the mountain to be the man so when come be become less man. He said when his girlfriend came he will go around, even if you can see what he want. That’s why he decided to leave his village and go to other village. Even in that village he doesn’t stay with other people he will stay alone in the room. So according to me I just saw this is traumatized him cause he didn’t get what he thought he will get. Cause the other one came without a feet. And the way the showed there it showed that they were beaten u, they don’t eat they took the spray with a paint that show an abuse there. I don’t know if they all get abused or I was so shocked cause even me I got a boy I don’t think I will take him there according to me.

RA: Okay you spoke more about the mountain. Is there anything that you know about circumcision that you know beside the mountain?

P: Another thing that I know about circumcision its Medical Male Circumcision. But I’m not sure at the hospital if immediately after birth if you can ask them if they circumcise him?

RA: Okay what is your understanding about what you said Medical Male Circumcision?

P: As I said medical, I think the way they do things I think their place is sterile. They won’t cut using one thing the whole day. If they cut they will throw that thing and the place is clean I think the germs are not there.

RA: As you are saying you had a boy you don’t think to take him to the mountain, you think your child can circumcise? If you think to circumcise which can you use?

P: As his father never went to the mountain he came here I think that me too when the time comes for circumcision, he will do medical. If ever they enforce to go there, I will take him to medical then from medical they will take him I don’t know what will they do because they said he came as a man.

RA: Have you ever thought telling your partner about medical male circumcision or a male family member about circumcision?

P: To my partner I was luckily because he understood, I think I was pregnant then that’s when I persuade him. I used to tell him about it I used it as a joke he was a having a foreskin I used to say it release tears this thing just as a joke neh so then I was luckily because the others of {} (name of NGO) came to motivate, he took it and stood up and go I don’t know whether it’s because I used to joke and say it release tears I don’t know even now he like to say to my son I have to take you for circumcision you will end up releasing tears. It was like that he listened to me and come to circumcise.

RA: So for you to use tears what were you mean to use joke, when you were saying it release tears?

P: I meant to say when he’s done sleeping you find that he’s releasing sperms so they will remain the foreskin then I said it release tears. Is it when you are crying there will be one drop of tears so its tears like that. And he used to say don’t fool me around just playing. But I think he took it from there maybe he saw why this person every day is always busy I’m going to prove him and do what he want me to do.

RA: So was that the way you motivated him to use a joke so that he comes and circumcise

P: I can say that’s correct that’s the way to use as a joke , men they don’t want to talk with them badly and go and remove the foreskin, you have to tease them just to make him understand cause if you can tell him to say go and circumcise this. He will say why because he doesn’t see anything wrong but if you have a way of approaching him you can be able to see if it is okay, this thing is wrong. You know they are like kids you can fool them with sweets, so that’s why I’m saying you have to find the way of approaching him it depends how do you know your partner when you think.

RA: By the time you approach him about this and you were using as a joke how did he feel?

P: Okay he…the time I said when I was talking about tears he was laughing because then I was with my sister just talking then this sister ii find that she also saying “ooh *Dikeledi*” I laugh. So the time we are done with sex I said *Dikeledi*. He asked what‘s that I said what it’s it he laughed. From then he stayed with it and motivate him and says I have to go and do this. that’s was when he decided to go I can’t remember when he was cross, my son used to say when he’s playing ii have to go and remove this it make *dikeledi*.

RA: What is *dikeledi*

P: *Dikeledi* is tears

RA: Oh

P: I used to say like that when I’m joking he used to ask what is it I will say its tears don’t you see is coming and it becomes as joke at home.

RA: So which language is that when you say *Dikeledi?*

P*: Dikeledi* is a Sepedi or Sotho

RA: As you used to talk there as women have you ever discuss about challenges that you come across when you motivate your men to go for circumcision. How can you advise a person who want to motivate his partner to go for circumcision, and you find that from man ‘side he never thought to go and circumcise.

P: As our topic it will start as a women you find that we are sitting discussing as much as I said I was sitting with my friend discussing that my partner haven’t done it she also said *ohh dikeledi* she laughed. Others they do meet those challenges there are different cultures like me I’m a Zulu when you tell him so others they just talk straight and it becomes a fight so that’s why I’m saying you can make it as a joke a little bit at home even him when he’s sitting he will say why this person always saying this? At the end of the day he will finally make up his mind to say its fine let me go and surprise her and she will hear when I’m saying I’m coming from there or to surprise her by telling her that tomorrow wake me up I’m having an appointment with the clinic

RA: In other words it’s very important how to approach your partner about circumcision?

P: Yes as much we work with the community, people and guys you can find them sitting in the corner talking especially when they see us with condoms .As we are persuading them and they say our choice and we are say take these chocolates they will say chocolates they say we want lovers, and we say lovers what do you mean by lovers? They will say no this one it’s easy to bust as he keep on saying it’s easy to bust then we will ask why it bust may you don’t know how to use it, he will say no is this things you see the lovers comes from SABS, and we say these things are our condoms, our chocolates and these chocolates are not the same the only different is that they don’t have flavors. We show them the SAB sticker and we say also your lover does have this. Then you will see a person taking them. Sometimes you berg them and say just in case you don’t have money for lovers and there’s a baby says take it. That’s why we are saying you have to joke with them, is it a person is laughing when start the topic. When he’s laughing you start the topic and say we got a clinic so and so. And its summer now we have to remove the polo necks it’s no longer cold. He laughs you see. We put it as if we’re joking .From then a person will laugh when his alone he will think about this and motivated to go the clinic and motivate others you see but they feel offended. So if he arrives there no one will know you circumcised and what no you will decide to go the clinic to do this RA: Do you think if women can go out and spread a message about circumcision within the community as you also teach about HIV/AIDS , sometimes you talk about condoms , and they tell you they don’t want choice they want lovers, then if women can go and teach ,encourage men to come and circumcise do you think it can be better if they can have a way to talk to men like the way of using s joke?

P: According to me I think if you can use a joke a person by the time you talk to him as a joke he will laugh as they sitting as a group and they are many. He will laugh I think when he sitting alone isn’t there are phones and so on when he look and google so when he’s alone he will decide to go. And the other thing I can’t work with the community while I’m angry. I leave my place, greetings my name is so and so I’m doing this and that may I please do this can I give you pamphlets and condoms, what condoms do? And what is Medical Male circumcision does? And he end up taking it when you talk about Male Circumcision it means I have a foreskin? It means you started now you offended him but if you came with the smile even if he was angry then you say how are you my brother? I’m okay eish its hot today. From there I’m saw and saw I’m coming from this place, you find that him washing his takkies and you say I’m having something you will look and there are chocolate what kind of a chocolate I don’t eat chocolate but this one you will enjoy sure…sure . You see that person look at you and you say okay you will enjoy. So you are now straight. You find a way to talk to him and will be interested. And you also call other from the house you will end up saving so many lives and he will smile when you are joking

RA: When you came to a group of people and teach about HIV and AIDS and having condoms and again you met one person and teach him. Is there any bad influence to a group or its better when he’s alone?

P: According to me when they are in a group its easy cause when they are in group when you come they will ask do you are you using a condom? Let’s go and test with you. When they are in a group they will be laughing, when you come and say are you using it and you say yeah I’m using it when he start saying lets go, just say in the afternoon just borrow me you r time now it’s hot I will be back and you give that thing so that when you talk with others they will become involved, free to discuss. When you meet one even if you try to talk to him he will think you are wasting his time. Sometimes it’s difficult he say I’m having a wife and the children you are wasting my time I don’t do this. So it’s not easy but when they are in group in most cases , others if you fine one you can be able to approach him he will end up doing it and things becomes right. I believe when we work with the community it’s very important even if a person is rude I believe that you have to try you give up after you tried.

RA: So how did you do start at jokes at home, and that joke works for you as a person woke up and say I’m going?

P: Honestly speaking, the time I was saying I didn’t expect. Cause I didn’t know that how he feel by going there cause I’ve never had talking about that but when he says he’s going to do this it means I know her weak point it means he’s celebrate is it you know if you passed you got more points you celebrate that there is difference that I can do to a person especially that I’m not related with him

RA: How long did it take for you to take a decision to say I’m going now and this thing of tears that they always tease me about it here at home?

P: At the begging we were always had a good relationship but I didn’t know how to discuss but as soon as I had about tears it didn’t take me long cause when I spoke about I was already pregnant I was stressing to say tears. I said I’m going to my new mother and he said he’s going to {} (name of clinic) on the following day. So she delayed a bit but he never takes too much time. I don’t think even the six month period did he took as I was busy joking with him.

RA: Okay at the begging you said you haven’t have a problem whether he circumcise or not but after your pregnancy it came to your attention that he must remove those tears what motivated you to encourage him to remove those tears when you are pregnant.

P: It means I didn’t have a strategy to talk to him. When I got this thing to motivate him I said what are the circumstances of uncircumcised then I know about medical male circumcision by the I motive him I was aware. By the time I wasn’t aware I wouldn’t mind telling myself it’s the same thing it is one thing he got someone pregnant *w*hile he was having. By that time I wasn’t aware I didn’t mind saying I it’s the same thing and I was pregnant so I didn’t know the difference to say why he should go to circumcise, by that time was well informed then that’s why I said this thing is not right he must remove it.

RA: It means you got the information about medical male circumcision then you were able to talk to your partner?

P: Yes it means I got it. Even if at the beginning I did received it you see if you got something you say okay its fine. Like my mother they told her to say okay its fine there is no problem its fine. But by the time you heard especially when you read at the clinic, especially when you are pregnant, a person if he had a foreskin it’s simple to get an STI so I also think that my junior …suppose this picture, and say to this person, luckily when we were testing I’ve never get an STI but I think a person can do this, let me show the light go and do this thing for the sake of a child

RA: Maybe what are the things that you can avoid when you talk with your partner or a family member you motivate him to circumcise, what are the things that you can avoid when you talk to him about circumcision?

P: Firstly you have to do a research, you have to check what are they’re believes where it is. Check what kind of a person? What is their understanding? When I talk about believes I mean if you are in a relationship to you talk about your believes and a person how serious it is when he telling you what is the person saying when you are talking and if you talk about something you will be angry. Things that you can avoid are his believes .Another thing you don’t have to be rude. Show him like you have to share things and how do you see thing.

RA: When you say don’t be rude what do you mean the way you talk and the words that you are using?

P: When I say don’t be rude the words that you are saying and the way you are talking with him. That’s why I’m saying when you come maybe to me you want information maybe I eat my chappies or I’m writing can I ask sister where is so and so then you I feel down, look at 1,2,3 what will you do when I’m saying check there? But when I’m saying the person that you are looking for go straight, turn there when you get there you will find 3 people and ask them. So he will understand that he goes straight there. So in other times you feel that people from this house you will not say the person that I got there is rude people who are working there they are rude hats why I’m saying when you talk to him you are approached him with respect. Don’t be like your words okay even if you see this person is talking with you have check the facial expression how is it , you have to be relaxed before you talk to the person.

RA: The way in which you used before using a joke which made your partner to stood up and got circumcised, was it easy or it’s the way you talk to someone is very important, you must not be rude, or you must not criticize his belief, and the way in which you approach it. Are there other ways in which we can use again to try and encourage doing circumcision?

P: According to me, I believe that it’s not simple that a person you can approach in a way that you are too serious. That’s why they say… it’s just like school as a teacher , if you are a teacher you represent yourself by the way you dress up ,your neatness ,than the way in which you approach your students in class. That why, l say a person can say this, you know a person is like this, I like him because of they, you check their characteristics. I think we should have characteristics so that as people we can find a way to approach one another. But for my side, I believe from joking and humbling yourself. Because at home you could make a mistake you know that your wife loves tea right? Let’s say I didn’t sleep by my house, you know that she is angry, you won’t just come in and say hello. You know that a person like her loves tea, so you make tea and give it to her if she is about to shout at you, she will not shout to such an extent she will get to where you did not want her . She will eventually get over it. So that’s why I say it you will be humble when you talk to that person it will be easy and she can listen to you.

RA: Do think that getting circumcised is a good thing?

P: I think getting circumcised is a right thing. Because since I got information circumcision is a right thing, because we know what it prevents, what, so you know that when a person is circumcised, even though it is not 100% is less than not being circumcised. That’s why I say getting circumcised as number 1. Cause you understand that other men they tend to cheat and get to sleep with side girlfriends, maybe if he and you call him he jumps, when you get home you to call him first he does not answer his phone so it mean he is with other women, when you get home you don’t want him to jump in water you just need him to do it, so that why I that fore skin may get dirt or STI. So already if you come home with it. I will also get an STI.

RA: What are the benefits do a couple get if a male partner is circumcised?

P: The benefits we get. I believe that there’s a good hygiene between the two of them and it is not easy to get STI infection even if you bath is it there is that part of you open you find them cleaning inside so he is in a hurry he just wash on top.

RA: Are there any other benefits that you can get if your male partner is circumcised?

P: We tend to get many benefits, as much we are enemies, it receives right? So if he is circumcised it will not be easy for me to get STIs, fine we do get them but it not easy to get them, and cancer especially cervical cancer, it is not simple that I can get them as long as your partner I a circumcised.

RA: Let’s say in relationships who do you think should raise the topic of circumcision?

P: Is it the relationship is between the two of us, he better come and he should release and say mother tomorrow I will go to a certain place. sometimes you as a women because your health is involved you also you should raise this topic in a relationship, In a relationship there is no one better than the other one we share visions and we help one another.

RA: Let’s say if female partners in a relationship raise a topic about circumcision, maybe the male partner will not think that ooh my word! Where this circumcision topic comes from?

P: Sometime as I said it came as joke, sometimes I asked myself that did you get someone who does not coming tears you see ,but that’s why I say whatever happens in a relationship if you are talking to your partner about it you don’t give up but you fail while you have tried your best efforts.

RA: So can you support a man when he starts to take a decision about going for circumcision?

P: Yes I can support but to support him by asking questions what makes you to think is the right time? Then he will he replied. Do you is it painful? He will reply, just question you see, I will show him that I want but it won’t be like I’ve been waiting the question of…but you have foreskin you didn’t have a pain when you think? Ask him if he has information; ask him how long do you have to wait? Ask him if you are going to the clinic will you give me, when he says no how long do have to wait, and ask him where I will get it. Just discuss as a topic but in a manner that you encourage him to go. Oh he will say will I wait for that six weeks without fulfilling my sexual desires. But the fact is if a person makes up his mind he will go.

RA: From your own knowledge what do you think is the major reasons that make men not to want to get circumcised?

P: Other men, you tend to ask if it is painful, others they think yhoo the six weeks my partner will get another partner out there, the other one will think who will hold me when I’m there, other one will think to come when he saw a face of women and he will say yhoo maybe I will be circumcise by the one who was touching my penis and he will run away, you see. Others believe in culture as I said I’m the Zulu the Zulu men are fighting and say why I should circumcise? I was born like this why I should die incomplete. Sometimes others they say it’s the same whether you did or not, others they don’t have an information at all.

RA: As a Zulu person and you are saying they don’t like to circumcise was your partner not difficult to you?

P: Luckily my partner, when I discuss with him I was lucky when we talk that he was interested. And my partner is not a Zulu but when it comes to my brother it becomes difficulty. But the way they are so difficult I believe the reason why they don’t come here is that they don’t have information that’s why most of the time we are saying the information, information! It’s that’s why most of the time sit that’s why information, information it’s when people becomes informed. So if he doesn’t know well he will tell you about death, but since I have information I will persuade the person because he has decided to die complete and the death could be quicker because you are complete but maybe when you are half you can live longer and see your great grandchildren. That is why I’m saying if you talk to them you have to be polite and sharp. It means you must on the same level with the person and be with your serious joke. Now you find that you are too serious and he will end up saying irritating. But at the end of the day he will listen. That’s I’m saying you can tell a person something advising you end being friends.so…that’s why when I go to look for virgin…you find that there is person who is so and so other one will say but because I know the truth I will decide to say eish but this person was talking the truth I will not use the 15 minds of other people that I heard out there. I will use the mind that say I know this is like this that person told me the truth.

RA: You spoke about people who said they will die. Even now you speak about it. Is that what your brother spoke about it that if I go for circumcision I will also die?

P: Yeah most of them they spoke about especially those from rural areas. When you start a topic and discuss he will say I will not go there God love me this way if he wasn’t like the foreskin he was removed it during birth. When you ask a person was the cell phone there long ago? But now we are leaving in the modern way we are different. Then he will explain that the grandfather was having a foreskin he was sleeping around at the end of the day you never find grandmother with HIV. But now when you look at the reality people are dying. And when I go with my big school bag with condoms and put it they say men’s protection when you check in the bedroom you will find that they worked. That why I’m saying have an approach.

RA: You spoke about the importance of education that people should be given information about circumcision and other things like HIV/AIDS. And also the importance use of condoms. Maybe the way to go to the community to educate people about circumcision how can we do? What other easy ways that we can use to make people aware the importance of circumcision?

P: I can say when you go to the community go with patient people. In the community sometimes when you say something they can discourage, then from there they will discourage like that and you end up not having that, a person maybe can go a person like I will say like {} (name of comedian) comedian people. Person who’s doing comedian can be able to do comedy is it people they laugh and enjoy. But again you say something them they will be interested in that is it he will be standing and discuss they will be interested to make people laugh, but you will speak about life things that are happening in the community like they are able to do it, are they able to listen. You will find that you are going *to joburg* now you will find a guy wearing a skirt and dancing outside then you will laugh, come inside encouraging you at the end you will go in laughing. You will find that you got the money and get a shirt that you like and you go. So that’s why I’m saying when we go out to the community singa vrazweo our *dikeledi* at home you should be polite have a heart for people especially if you are working with people. You have to know how people do and how to treat them.

RA: Do you like working with people?

P: I like to work with people too much, I believe that I know people’s character I am able if a person even if you can speak something I can take it and throw it away it means you are giving a person a choice but you speak with him to say 1, 2, 3.its him who will decide to take it or not. Sometimes you can come to me and say something, abstain, take your pill, you cannot take me by hand and say let’s go for circumcision. It will be up to me, but him at the end of the day when there is no time he will think there was a time where a sister so and so and says this if I did listen it means she not working it means you give him a choice but not to say you are showing him you don’t say it’s his choice do it! (Shouting) he must decide. He might say I’m coming to this place and he doesn’t make it, it means he didn’t want to buy your face.

RA: So you said you got information about circumcision from {} (name of NGO), do you think {} (name of NGO) people did play a role in the community to educate about circumcision?

P: I believe that did play a role in the community. As much as in my relationship they play a major role, I said to my partner there is *dikeledi* and while I was speaking to him in the matter of time they came and knock and preach about it that’s why I’m saying they did, even many school kids, you will find a person, the other one is young and keen to age group, and even when you talk to the people in the community do you know {} (name of clinic)? My nephew like we spoke about it even yesterday we were busy about the same topic, I said tomorrow I’m going for an interview, why men they don’t want to go for circumcision and said I can’t tell you the reason behind that , personally I will go next year I’m waiting for my father to tell me what to do that’s all .That’s why I’m saying you give a person a choice, you give to pave a way ,like now they will ask you to go to school at home and you don’t go so you will find that you are old and you didn’t go in such place no you say I didn’t go there and now I reject. But they gave me a choice and I decided not to take.

RA: Thank you so much now we came to the end of the first part of an interview but is there anything that is important you want rap up or to say about it what?

P: The important thing that I want to say neh, I want to say please to our brother, our sister we are all together in this it’s not only our brother I ask our brother please lets circumcise, let’s keep on circumcising our things our lives can be better we will not burry every week end. Now we suppose to bury our mothers our grandmothers than our grandmothers bury us. So even our sisters must find a right away to talk to their partners and friends and say let them go to circumcise. They must not say they smell and say the kids are having pap.

RA: They are saying they have pap how?

P: They say in the foreskin when you are not circumcised you have pap I don’t know what that mean it mean there are white things they say are pap.

RA: So those are the female partners who tease the male partners to say they had pap

P: Yes and say this one had a chimney; this one had polo neck things that are not there according.

RA: How do they feel when men are told that they had a pap?

P: He feel, you see when a person says you got pap it means somewhere you ate something with no pap where it’s clean you see

RA: So this can it make him to go and circumcise or to be angry?

P: We are not the same, others can be angry others can say let me go to the clinic. But even if you say your pap but put it in a nice way, and say ei baby no the pap is ready.
