# Supplementary material for: “To speak or not to speak”: A qualitative analysis on the attitude and willingness of women to start conversations about voluntary medical male circumcision with their partners in a peri-urban area, South Africa
Source: PLoS One. 2019 Jan 25;14(1):e0210480. doi: 10.1371/journal.pone.0210480 (PMC6347244; doi:10.1371/journal.pone.0210480)
Supplement: S1 File — (ZIP) [file pone.0210480.s003.zip › QF015_QC2.docx]

PARTICIPANT ID (P) QF015

RA: ok, thank you my sister for agreeing to take part in our project I’m going to ask you for your permission err… to audio record our conversation

P: ok, I allow you to record

RA: so I can record ok, err… so sister where do you come from err… can you tell me a bit about yourself

P: about me

RA: yes, yes

P: ok, I stay at {} (participant address) I stay with my mom and my mom’s home is in {} (participant address) my mom is a mopedi (culture) and my dad is a tjhangan (culture)

RA: ok, so err… {} (participant address) is closer to {} (participant address)

P: yes at {} (participant address) you see when you are going to {} (participant address) the shacks that are close there

RA: ok, so you stay that side

P: mhm

RA: so like you didn’t struggle to come to the clinic

P: no I didn’t struggle to come

RA: you didn’t struggle ok, so what work do you do

P: ok, I work as a CSP field worker

RA: mhm

P: we go around doing door to door

RA: yes

P: about HIV/AIDS

RA: ok

P: we ask people and remind them that they always have to go to the clinic

RA: yes

P: to check all the diseases that are there and if maybe they find out that they have a disease they can see that they can treat it while it is still early

RA: ok,so you go house to house telling people about…

P: mhm we do door to door

RA: so here, here err… you tell them only that they have to go to the clinics an when they are sick they must go to the clinic

P: everything and we also tell them about this one for men

RA: mhm

P: that they must come to the clinic start from nine years is going up a boy child who hasn’t cut you come to the clinic to book a day for your child and then they give you a date you bring him and they cut him for free you don’t pay on the same day they gave you

RA: ok, so on this information you give to people you also encourage them to come and circumcise

P: mhm

RA: ok

P: we refer them we do have papers to refer if a person really wants to come we give then a referral letter to go to the clinic

RA: oh you give the referral letters to go to the clinic if they want to circumcise

P: mhm

RA: ok, so when we speak of circumcision what do understand what comes to your mind when they say circumcision?

P: it means that they cut they cut them on the foreskin and its them you mean I must explain that…

RA: yes when they say that circumcise what is it that they are doing

P: it is that they remove the foreskin

RA: yes

RA: mhm

RA: so maybe can you tell me what makes people to remove that foreskin

P: oh according to me and the way I learned since I grew up its that its right because of that if the foreskin is there like its closes so many people don’t use condoms and the dirt stays in there

RA: mhm

P: mhm it’s the way since I grew up they taught me that dirt causes diseases so in other cultures besides that the dirt stays there they say if you didn’t go like to circumcise you are not a

RA: in other cultures

P: ya you are not a man it means that for them to say you are a man and you are strong you have to go and cut this thing of diseases they don’t believe in it

RA: oh so the reason that makes these people to go for circumcision is that they want to be men

P: yes to be a real man so that people can respect him that he is a man no he is strong because if you didn’t go they say there is nothing that you can tell me because you are not a man a man is there one who went there who knows

RA: ok so can you tell me about the different ways of circumcision that you know

P: mhm I know this one that they come to the clinic or go to the doctor to cut is it

RA: yes

P: and there is this one that they like go to the mountain

RA: ok

P: they go there and stay they cut them that one I wouldn’t have more information about it because they tell they that they are not allowed to tell women about what is happening there

RA: oh

P: ya that they are not allowed it’s their secret they tell the men not to tell us about it you can only hear that one sharing a bit with you about what is happening there and then another one I don’t know if it’s done around I heard from people but I’m not sure about it its done by the tjhanganes that the tjhanganes when they say they are going for initiation they don’t go to cut they go like and stay in a house like they fast in a house like they stay maybe for the whole month not going out

RA: ok

P: doing nothing just sitting being locked up in there then after the days that they have set they say it’s an initiation you go out they grow you up and buy stuff for you what they is what I don’t understand I only heard that they do it that way

RA: oh so that one they also say its circumcision for, for…

P: for them it’s their believes just to stay in a house they do nothing to you they give you days maybe the whole month you don’t see people you don’t go out you don’t o anything then after the set date they buy you stuff and grow you up and do a ritual for you

RA: oh and then the other one

P: the other one I know that it’s like it’s the same as the Xhosa one that they just cut you for now

RA: oh so they just cut

P: mhm

RA: and then this one that you said from the doctor do you know what happens there

P: the one from the doctor

RA: yes how they work

P: like the way I see it works that they give them a date neh

RA: mhm

P: and they come on the same day I don’t know that maybe they give them an injection all that but they remove it

RA: ok

P: the foreskin and then on the same day you go it’s not the same as there they stay so that they can be healed and there has to be a male person there to take care of you who will be busy bathing you and everything until you become right

RA: oh so there at the initiation they there are people who take care of them

P: ya most of when we are going around door to door it’s like people don’t like this one

RA: ok they don’t like medical circumcision

P: ya because when they are done you just go home and then like other people say pain is it like it’s not the same like another person is weak and another one is that so when they are done they go home when they get there they sit and feel pains on that time you feel pains when they look at the private parts like they say there’s blood and it clots and then you have to have somebody that will help you sometimes one is not brave enough to bath himself and remove that blood and its now dry so in the initiations at the rural areas when you go to the initiation there is an older person or your brother that will take care of you always and he is the one who wakes up early and take care of you and help to see how you are doing and bath you, you see

RA: mhm

P: so this one is difficult because when you get home sometimes they stay with their mothers they are afraid to undress in front of them to show them what is happening

RA: so maybe what is it that makes them to be afraid

P: mhm

RA: hat is it that makes them afraid I’m mean it’s his mom

P: like maybe they tell themselves that that they are old if it’s a boy child it’s not easy like there is this one who came here he stays closer to my house when he came back he felt pains this and that he was screaming saying yoh mama what, what but he didn’t want to show her the mother ended up not knowing what to do and she ended up fetching us

RA: mhm

P: because she knew that we are working for ward two and we work with such things and it is us that explained to her so that he can go and what to expect

RA: oh

P: so when we got there she said yes he went today so since he’s been sleeping facing up saying it is painful and he regrets why he went there it is his mother that forced him to go maybe that one was going to be better

RA: mhm

P: you see ya he thought that maybe I don’t know they are afraid of that thing I don’t know because I don’t know what they do there

RA: mhm

P: you see what’s the difference there

RA: ok

P: mhm so when I asked him if they injected him like when you come back an injection for pains maybe because you can get healed for that time but where you are scarred

RA: mhm

P: it get painful it comes back later he said no they just said go and then they don’t call you for check up

RA: oh

P: you see another thing they complain about maybe they don’t say tomorrow you must come so that we see how you are

RA: ok so

P: they just leave it open like that and the blood clots there sometimes it clings there

RA: mhm

P: they say this one is worse they are afraid of it

RA: ok, so it’s what people say about the circumcision from the clinic

P: ya this one from the clinic

RA: that they don’t call them for follow up

P: ya

RA: and that …

P: that they come for follow up or sometimes he won’t be able to at least that they send people to this number to go and check and see that yesterday as we did this person how is he

RA: mhm

P: you see maybe some other time there should be a male person who will be able to see him and show him what to do so they say that one is better because by the time you come back you are right there is someone taking care of you from the start till the end

RA: oh so they say circumcision in the mountain is better because there is someone that takes care of you so here you think if we can send people to go and check them are to call them for check-ups then it would be better

P: you see like in a place they say like in a place when you are hurt you are the only one feeling the pain and you don’t have the guts to touch but another person will say I know that it’s painful but let me do it and then you will hold on even if you feel the pain mhm

RA: ok so have you thought of telling any family member or a male person about circumcision

P: mhm

RA: like I told him neh like I have a brother who comes after me he is nineteen now he hasn’t done it he hasn’t gone for it when I told him because of the one that o told you about he told him the things that I just mentioned so even for him it’s hard to come here

RA: ok but what is, is that made you encourage him to get circumcised

P: me

RA: yes

P: because of the things that they teach us always I know that he has to come and cut because of diseases one thing that I was talking about those diseases these other things of a real man what, what hai one think I like about it is the problem of diseases I heard that it holds dirt here and causes like drops

RA: ok

P: mhm I told him that you see that he is now old and he is going to start having sex sometimes when we tell them about condoms when they get there they don’t use them

RA: ok so you were talking about diseases

P: mhm

RA: ok, so I heard you saying he was told by his friend

P: mhm

RA: so he is fearing to come because he is afraid of the wound or he is afraid of the fact that there is no follow up or what

P: ya ,ya he fears that there is no and like sometimes it’s the other thing that I just said that when he is grown up as a boy he tells himself that he is old and he won’t be able to undress in front of me as his sister and his mother as he is the only boy he is like yoh what am I going to do

RA: mhm

P: yes and some other time he gets embarrassed by that you see mhm now with that one he said it’s better that he goes for initiation because he heard that you don’t go alone you are accompanied by someone

RA: ok

P: you see

RA: so err…

P: and then he fears that when they do that he is not sure he doesn’t know and I also don’t know because I am a woman

RA: yes

P: he also asked me and I said there I won’t be able to answer you I don’t know what I know is that you go they do it the same day or maybe when they cut you they inject you so that you don’t feel the pain

RA: mhm

P: so you find that I also don’t know when it comes to that part and I don’t know what happens I just say they will explain to you when you get there

RA: mhm

P: the doctors

RA: oh so he also doesn’t know what happens when you come here

P: mhm he just knows that they do what they do and after that they send you home at least you know when we go around the communities neh they say at least if there was a ward for them you see so that when the cut them they admit them maybe for seven days checking him and telling what to do

RA: mhm

P: not that they do same time on the same day they say go home you see that’s what they don’t want when you get home you feel pains and even die there

RA: ok so the other reason that makes people not to come is that they want to be admitted

P: yes

RA: oh

P: like so that they can be right and sure that even though they feel the pain at least there is a doctor that tells them that you are going to be alright you must do this and that than saying go home we are done with you and he doesn’t even know that when he gets there what is it that he is supposed to do he is just feeling the pain you see

RA: ok so err… like if a person wants like you maybe kike you want to tell somebody to go and get circumcised or you want to tell your err… younger brother

P: mhm

RA: about circumcision what are the things that you are not supposed to tell him or that you have to avoid when you are going to talk to him what do you think

P: that I’m not supposed to tell him

RA: ya like that you have to just avoid is it you want to encourage him to go get circumcised

P: what I saw is that when I go to him I explain that when you go there to circumcise neh they are going to help you for diseases like drop and explain those things the lines that I don’t want to mention like it’s the ones that are going to scare him like that when you get there they are going to cut you, you hear like most of them things I think I’m gonna scare him

RA: mhm

P: when I tell him like that he will have fear and say no you see with that one it gets differ-, difficult to them because of they even hear from people who come from here outside so you just go and a person says yoh

RA: ok so people who are encouraging other people to go for circumcision are not supposed to tell them that they are going to cut them or what they’ll do to them

P: isn’t it you see the circumcision at the mountain if a person didn’t go for initiation they don’t tell him that initiation is

RA: oh

P: they don’t scare them that’s why these people just run and go there only to come back dead because they don’t know what happened there

RA: oh

P: it’s your secret even if you can get a boy and ask he won’t tell you it’s the rule that they gave him because they know that they are going to scare others and they won’t go even if you beg him what, what so this one we just go around talking about it and then yes they hear from the others talking about it these who have already done it

RA: mhm

P: you see so that is what makes a person to have fear and say yoh imagine me going to get cut on the same day and feeling pains you see

RA: mhm

P: mhm

RA: ok so which one do you think is better the one that people go to the mountain or the one that come here to the clinic (voices on the background)

P: the circumcision from the clinic is right because I think before maybe the doctors check you first is it

RA: mhm

P: how you are before they do anything than that one because on that one when you have bad luck you just hear from the news that others won’t come back they have died we don’t know what is happening

RA: mhm

P: what was the problem so here when you come the doctors they check you first then they cut them then send them home

RA: mhm

P: it’s because they have already seen that this person has no problem he will be fine no problem you see

RA: ok so err do you thing male circumcision is a good idea

P: yes according to me I think it’s a right thing mainly because of diseases

RA: because of diseases

P: mhm

RA: ok so how does circumcision help in people who are lovers?

P: how does it help?

RA: yes people who are lovers a couple how does it help

P: mhm

RA: if a man went for circumcision how does it help?

P: that it helps with what

RA: or why is it important for people in a relationship for a man to go for circumcision

P: is it not because of the drops

RA: mhm

P: and then like if he didn’t cut even when he wipes off they say the dirt hides and stays there isn’t it

RA: oh

P: then same time he comes back and sleeps with you and then what happens next the dirt comes together with the other persons and creates a disease

RA: oh so it helps by removing diseases

P: mhm no it doesn’t remove diseases

RA: ok I’m saying it helps by stopping diseases

P: yes even if it won’t remove them but the drops at least

RA: o it reduces

P: mhm

RA: ok so you think for people in a relationship who is responsible for raising the topic for circumcision between a male and a female

P: I think both

RA: mhm

P: I think you must just start and talk both of you if you see that he is not talking about it and you can see that he is not circumcised you as his woman I think you have a right to start and explain just like I’m explaining

RA: mhm

P: so that he can go because of such things as drops I also have a right to start and tell him if he is not talking and I can see that he dint do it

RA: ok so in which ways would you start as you say you can start what are ways that you can use to start the topic

P: I can start like when I see him sitting and we both relaxing and he is just sitting on the bed I can start by asking him if he would like to go and circumcise

RA: mhm

P: mhm and tell him that I see that it is safe if he can go and do it and explain that if he is not circumcised there are many things that can happen you see

RA: mhm

P: ya

RA: ok so I heard you saying that you go around telling people about circumcision and so on so if or with people that you have told about circumcision what were your experiences like to tell like to tell maybe your partner or your brother how was it at first when you spoke to them about male circumcision

P: oh others just laughed at me , mhm like older men would tell young boys and end up looking like a fool according to other peoples beliefs is like if you talk to a woman about such it’s like , like to them these things are a secret

RA: mhm

P: things like these are not discussed with a woman you see others would laugh at you and say you are crazy and just leave you there and say no I won’t talk and another one would just say o go to the mountain I don’t do such things you see

RA: ok

P: another one would say I won’t tell you about…

RA: circumcision

P: ya he won’t tell you about it ok so others are interested they would be like which clinic do we pay no well go I have boys so on and so on others get happy

RA: mhm

P: with others hey it’s difficult

RA: so what do you think would make men not want to come for circumcision?

P: what would make

RA: or that makes men not want to come for circumcision

P; others it’s their cultures like the Zulu’s

RA: mhm

P: ya the Zulu men would say they don’t agree it’s their culture

RA: mhm ok

P: ya and then others its fear and what people say

RA: mhm

P: others its just what can I say to be stubborn maybe they need more information then the one that we are giving to them because of even the one that we are giving them its limited we just pass the message we don’t know anything about it we just refer them for more information

RA: oh

P: ya we just say go to the clinic for more information you see

RA: mhm

P: maybe like as we are like as we work with field workers if maybe we got trainings and all to teach us more about that so that when we get there we know everything to explain to them about what happens you see

RA: ok so you think that if they can train you more so that you can be able to give them more information everything

P: because again when we get there many things many things when we get there we don’t kno9w them and they get discouraged even if a person was exited and keen to go with you and then you say I don’t know and you give him a referral letter and say go to this place so that they can explain to you others would say I will go then just put it away and not go

RA: ok so now you as a woman you are able to go and tell oh like you tell people about circumcision err… if there are women who are not working with these things of circumcision do they share their experiences of trying to tell a male family member about circumcision or have you heard of such things

P: mhm it’s difficult to tell a man like that in other cultures because he knows and sometimes he will say you are disrespecting him or you have insulted him like what you have said to him was an insult

RA: mhm

P: ya but then with others

RA: mhm

P: they do teach them like they go to the clinics already with the knowledge of such things so it’s easy for them to understand some other time we help like when we are working door to door always we must carry condoms and pamphlets and make sure that on every pamphlet like maybe we give them three it’s for TB another one is for HIV/AIDS another one is for circumcision we leave it for them if maybe they are embarrassed or they have a brother or has a sibling

RA: mhm

P: that is there and hasn’t gone we say if they can’t talk to them they must give them the paper to read

RA: ok what are ways that you thing men can be encouraged to come and circumcise when you said you give them pamphlets and then go door to door what is another way that you think can be used to encourage men to come and circumcise

P: mmm I think that at least maybe if they do ward to ward and do like do campaigns and have workshops

RA: ok

P: ya for males maybe only teaching them more and explain more to them going ward to ward it would be easy like that

RA: ya

P: because with us shame sometimes they don’t even want to talk to us they say thank you we can’t the only think that we ask is that you leave the paper

RA: oh so they don’t want to hear anything

P: mhm

RA: so err… what do you think could be the reason that makes men if you say you want to talk to them they say they don’t want to hear anything what do you think is the reason

P: the way I see is that they think maybe we take them for granted because of its as other cultures they know that this thing you only talk to men about it you can talk to a woman

RA: mhm ok

P: yes as you see its their secret they don’t tell anyone what is initiation even another one maybe they talk amongst themselves and all but with a woman at all totally you don’t talk even there it’s their rule when you are done and going home they tell you it’s their rule you don’t tell your mother

RA: oh

P: what is initiation so that’s why it’s hard for them to talk to us women

RA: oh so if err… maybe if they can talk to men you think maybe it could be better

P: I think it could be better if they can send men or do those workshops or go ya

RA: mhm

P: it should be only for men only it’s easy in men for men to talk to men about these things

RA: ok but you think like if a female can tell like her partner about the circumcision do you think that could encourage him or discourage him

P: oh they are not the same other people it could discourage them another on can get angry and no longer free around you but the way is see it right it’s so much easy

RA: mhm

P: if you are his partner to tell your partner there are so many ways on how you can start him even if he doesn’t start and is not yet circumcised you tell him a story that there is this thing that it can be risky maybe he will also ask you questions about what happens then you start so you can start to explain to him and then you see and you tell him even you I think you can go and do this thing

RA: ok so err… if a man let’s say you are in a relationship a man just chose to say that now I want to go and circumcise

P: me according to me I can be happy because of I have more information about this thing and yes its right he has to do it you see

RA: ok so it won’t affect you in any way

P: no

RA: ok so err… it’s time for us to close this discussion

P: mhm

RA: so err… is there anything that we didn’t discuss here that you would like us to talk about or that you would like to add

P: with this one for men

RA: yes with circumcision

P: I think I spoke about everything that I think could be happening maybe if they can get a workshop only for them only maybe move around ward to ward men only talking about men

RA: ok

P: and that one that they fear is that they come here to cut same day he goes

RA: mhm

P: you see that is the thing that makes them have fear that there are no people who come and do follow up that yesterday’s person how was he and how he is doing you see

RA: ok so we are going to continue we are err… another part of the research that is called pile sort technique

P: ok
