# Supplementary material for: “To speak or not to speak”: A qualitative analysis on the attitude and willingness of women to start conversations about voluntary medical male circumcision with their partners in a peri-urban area, South Africa
Source: PLoS One. 2019 Jan 25;14(1):e0210480. doi: 10.1371/journal.pone.0210480 (PMC6347244; doi:10.1371/journal.pone.0210480)
Supplement: S1 File — (ZIP) [file pone.0210480.s003.zip › QF016_QC2.docx]

Participant ID (P): QF 016

RA: Do you agree that I can record with the audio recorder when we do our interview?

P: Yes I agree.

RA: Okay. Can you please talk about the knowledge you have and your thinking in regards to medical circumcision. What do you understand in regards to medical circumcision?

P: My understanding isn’t?

RA: Yes

P: I see it as something that is right cause you won’t get many sicknesses than going to the rural areas to circumcise there

RA: Okay. You say you won’t get many sicknesses ne?

P: Yes

RA: Which sicknesses do you get?

P: Like in the rural areas we won’t say can I please be separated ne? cause we do not see them that the razor which they cut them with do they sterilize them . Cause at the doctor when you enter already they have packed them, you also see that okay here these ones come from spirit. Cause there at the rural areas there are many people, how will you know that they used this one or a different one.

RA: SO now which types of circumcision do you know?

P: It is the one from hospital and the doctor

RA: Can you talk about the difference between the two, How is the one different from the other?

P: The difference I do not know. Don’t they do it the same way?

RA: I do not know I wanted to hear from you

P: Okay here at the clinic in my knowledge they check first that you do not have a sickness that is there. If you do not have they do it then you come back again for a check -up. You busy going to the doctor and stay at home for six weeks until you heal.

RA: Okay, by the rural areas you said it is which one?

P: The rural areas I said that they go and stay months or weeks I do not know

RA: Mhh…okay…mhh. Have you ever thought to tell a male maybe your partner or a family member in regards to medical circumcision?

P: Ehh. I have told my cousin

RA: Why did you tell him?

P: Because he asked me

RA: What did you say about that?

P: He said he has never done this to go circumcise. So I wrote a letter there where we worked with it, he did it here at the clinic and got help

RA: You work with what things?

P: Like teaching doing door to door.

RA: Teaching about what?

P: About AIDS, HIV, TB , diabetes, high blood pressure

RA: Okay. so you but have you ever thought about it when you sitting you find yourself thinking about medical circumcision?

P: Thinking about like?

RA: Do you just think about it?

P: That it is something right?

RA: What causes you to think about it?

P: Because the thing that makes me think about it is that a person who is uncircumcised is able to get sicknesses quickly than the one who is cut

RA: Ohh okay how?

P: The one who is not cut the dirt stays in front isn’t?

RA: Where does it stay?

P: On the penis yes it stays there the dirt yes then he goes and meets a girl then drops happen

RA: Mhh okay now what would it mean if a male talks about this issue of circumcision with a female? Would it make a difference maybe if this issue that a male must come medically circumcise, would it make a difference if a female said it or not?

P: Yes it would make a difference cause he would listen to me a male if I tell him to go and circumcise

RA: He would listen to you? Why would he listen?

P: Isn’t I am telling him that if he is not circumcised he will get many sicknesses; there are some sicknesses so it’s better for him to go and cut.

RA: So now if he would want to go circumcise what would you say to this issue?

P: Yes I would tell him that good idea

RA: Why would you tell him good idea?

P: Cause it is something right isn’t for him to do it, it is something that is there

RA: Why do you say it is something right which he must do? Why must he do it?

P: To avoid sicknesses

RA: So now you think that which ways a person like you can raise this issue of medical circumcision to males? Like how if you are a female how do you say to a male to go and medically circumcise?

P: Okay me I would just discuss, start to just discuss

RA: What kind of discussion?

P: Like just discuss just about talk about where I work, what kind of work and that we help people that a person who has never got treatment that where can you get treatment then we start to talk that even here there is circumcision whatever age it does not say anything, even if you are old he can come and do it as long as he will get help. Even if he says he is old he will not be able to do this this thing wants young people

RA: So how would you tell a male?

P: I would tell him that ehh to avoid everything like that is there like sickness just come and circumcise

RA: So if you think what other ways can be made for a male to get information that he must come circumcise?

P: Mhh…I would make flyers, I would advertise then I would go, I would go to VOT

RA: What is VOT?

P: It is FM to talk with them on radio and announce that males can go and circumcise that this thing how important it is

RA: Okay, why will you go to radio?

P: Cause the way I see it is the easy way in order to talk so that every person will be able to hear

RA: To be able to hear?

P: Yes cause this one of flyers is okay it is good but you will place them there and they will be taken by the wind again the person from the *spaza* will say they were taken by the wind, and go pick them up and others that push papers just throw them. They just say *ayi* this one is boring us

RA: They say that?

P: Yohh…

RA: So you have experience of that people take here and throw away over there and that they bore you?

P: Yes because we see them, when we finish giving them the papers they throw them away that we have just disappeared they throw them away just here. They don’t even wait for us to disappear they throw them away where we are standing; they just tell you that we already know about this thing. But the person if you see that, this one does not know anything.

RA: Now you think that what makes people do that? You do not know ne? At first the time you were talking you had said you would tell me that even if a person is old, why would you explain this issue that he is old? Is there a problem in being old or young in this issue of circumcision?

P: Isn’t some see that they are old they do not do this thing anymore

RA: So it is done by old people or young?

P: Anyone as long as you have not done it you can still do it

RA: So as long as you have not done it? So he must do it?

P: Not that you forcing him. Isn’t some say it does not go along with their culture this thing. Even if it is at the doctor

RA: What says it is culture?

P: The Zulu’s say. We once entered a house here in {} (reference to an address). At this house they are Zulu we found the father and his male son and he said here in his yard he does not want a person who will do this thing. He asked us to take our papers and leave with them. They say Zulu’s do not do it.

RA: So what did you say when you left there?

P: Ay we won’t say anything. We just departed because the father said so. It means it his law in the house.

RA: What was your answer to him?

P: We arrived and greeted him. He was made angry by the soul city books

RA: Why do you think that the soul city books made him angry?

P: When we departed they boys we found sitting under a tree said there we entered by mistake because the father there does not like these things, now we went there to teach them isn’t. We we sent by the department of health to teach them. We did not want to know that his law of the house does not go along how.

RA: Now you said this father maybe was made angry by the books

P: Yes many people these books they do not want them just like those who attend church at ZCC. Even condoms if we give them they do not want them. They say they do not use these things

RA: People if you give them? Okay now here at this home you arrived and said what?

P: We arrived and entered and greeted, we told them where we come from and who sent us. This father just said if you have come to tell us that there is circumcision that thing is not done here in my house. In fact the whole family from the rural areas it is not done. The Zulu’s don’t do it. We said okay. Many people say the Zulus do not do it. They do not go to the mountain, they do not go to the doctor.

RA: Many people say what are their reasons?

P: Ayy I do not know they say the way they grew up

RA: So what is your culture?

P: We are Ndebele’s

RA: Ndebele’s how is it there?

P: They go to the mountain

RA: They go to the mountain?

P: Mhh…and the girls go to *eQudeni*

RA: They go to *eQudeni*?

P: They go check if they are still virgins

RA: Okay. Alright do you think that a person just like you what things must they avoid or not do when trying to talk to a male trying to show him or encourage that they must circumcise

P: I would try that if I talk to him not to include too much the word to circumcise

RA: Not include the word circumcision too much. Why?

P: But tell him that the information he will get at the clinic. Cause if you start talking about this word circumcision other people get angry they see it as you are insulting them like you are disrespecting them. If you can go to {} (name of clinic) they do circumcision and take the child if ever you have also not went. Isn’t it is the way they taught us to talk to them.

RA: They said you must talk to them how?

P: They said we must tell them that at {} (name of clinic) there is male circumcision it starts at this age to this age. They test they do everything. What I see according to me ne I see that isn’t we tell me that they start by testing for disease. People are not afraid to come and cut they are afraid to be tested. Me that is what I see. A person to test they will be afraid especially a male.

RA: Why do you say that? That a person especially a male that they are scared what are they scared of?

P: I do not know I wont tell you what they are scared of. If you say they test if the mobile clinic are standing this side and you tell them there is testing this side they will say I am coming but will not come back.

RA: So what is it they are running from?

P: We don’t know what they are running from cause if they have HIV how long will it be sitting?

RA: So now if this thing this issue about circumcision you say it to your partner or a person who is family how are you supposed to say it?

P: Okay before I answer you, let us say they have done it my partner, they have done it in my family; it means there is no need to ask them isn’t?

RA: Okay then a male in the community how would you talk to him?

P: Cause I do not know that he has done it

RA: Isn’t because those in the community they do not know. If you think how can we do it because we talk with them?

P: Ehh the way I see it we can do what you call we call them like we have a meeting yes we place them like at the hall or a ground then we tell them

RA: How do we tell them? You are a female you standing in front of males how do you start to tell them?

P: I start by saying that ehh there is a clinic here obviously they will say they know it they want people who are not circumcised.

RA: They know that this clinic they medically circumcise?

P: Mhhh

RA: Okay. Why do you say they know it?

P: because isn’t the paper that are there are now all over and the pamphlets are posted everywhere, there is no one who does not know

RA: Alright you will arrive and say what to them?

P: I will arrive and tell them that the clinic wants people to come circumcise. If you know yourself that you are not circumcised must go there and get help, not that we are disrespecting them we are asking that to come to the clinic

RA: Why are you saying they must not say we are disrespecting them?

P: Other people do not like such talks, they do not like them they get angry. They ask you that if you look at me you see that I am not circumcised. How will you answer you?

RA: What do you say when you respond?

P: I will not respond to him

RA: So you have experience of this thing? Have you told a person to come and circumcise?

P: Yes there is someone I have told but last of last year

RA: How did it happen for you to tell him?

P: He was sick this boy so his family member my friend came to me and said cause I work with people like this you see, can you please come and talk with my family member. So I also went there and spoke with the family member to go to this place cause he said he has this thing he has drop this boy. He didn’t hide it to me and he told me that he went to the clinic at {} (clinic address) they said he has drop. So I told him that since you have drop go to this place at {} (name of clinic) clinic and circumcise. I asked him I said have you done it this thing are you circumcised and he said no I had not done it. I said go to {} (name of clinic).

RA: Why did you ask him whether he is circumcised?

P: Ay isn’t it is something that just came to my mind cause he said he has drop. I just asked whether he is circumcised and he said no

RA: What did he say when you told him to go to {} (name of clinic)?

P: He said he will come and indeed he came to {} (name of clinic)

RA: So how did you go about when telling him to come to {} (name of clinic)?

P: Isn’t his sister called me, I arrived and spoke to him and said if you can go to {} (name of clinic) you will get help that you need because the pills they gave you, you see them as not helping. Go to {} (name of clinic). And he came indeed here at {} (name of clinic).

RA: Okay how did he become when you told him that he must come to {} (name of clinic)?

P: Ay he became alright and asked me again and said will he get help at {} (name of clinic)? I said yes he will get it.

RA: Okay alright so you think that to circumcise is a good idea or not

P: Yes I see it as a good idea

RA: Why do you think that it is a good idea?

P: Cause it will help this generation that is growing up and the one coming

RA: How will it be helped this generation that is growing up and the one that is still coming?

P: In the sicknesses that is there

RA: Which sickness is there?

P: Just like STI and HIV

RA: How will they be helped?

P: If you are not circumcised the sickness stays there on this thing the penis then you go and sleep with a person, with this girl and she also goes to sleep with another.

RA: Okay so you think that which are the benefits of circumcision for two people who are involved or a couple?

P: Can you please explain to me in Zulu

RA: The benefit or the benefits of circumcision for people that are a couple that are in love. What do you think they are?

P: If they are circumcised at a doctor the sickness is less it won’t get them if ever they pay *isinaphu*

RA: What do you mean if they pay to each other?

P: Like if you stick to one partner. Not this one to say tomorrow you are here tomorrow you are there.

RA: So is that the benefit or benefits you think of? Now between two people that are in love who do you think which person must raise the issue of circumcision?

P: Like in the community or together?

RA: No to two people who are in love, a male and female that are in love. You think that a woman must raise the issue of medical circumcision or a male must raise this issue? Again why do you say that?

P: A male

RA: Who must raise the issue?

P: Yes

RA: Why do you say that?

P: Cause he is the man, he will be able to tell and talk with them others that want help

P: Okay but in a relationship?

P: Okay if we are together. Okay on that one it can be me.

RA: In a relationship why must it be a female?

P: Ehh when we are sitting talking and I am telling him that he must go and circumcise.

RA: Why must a female say it?

P: Because a man is lazy to think (Laughs). He does not think straight.

RA: What do you mean when you say he is lazy to think?

P: it must be the woman who thinks for him that if you have done this it will be alright

RA: Why must it be a woman? Why is a man lazy when he thinks? Can you explain to me the things that show that he is lazy when he thinks? How does a female think?

P: Cause everything is fixed by the female

RA: What kinds of things are fixed by a woman?

P: She puts the children first, even at school she is the one that goes. The man is always busy, he is not busy, and he is always busy.

RA: Why is it like that anyway?

P: Ay we grew up like that also

RA: Alright. So if a male takes a decision to circumcise what would a female say would she like it or not like it or she would be in the middle of the view of the male?

P: She would like it

RA: Why do you say she would like it?

P: Cause it is something right isn’t he wants to do it

RA: Okay what must a female do to show that she likes it?

P: She must make him happy. Tell him that it is the right decision that he is taking

RA: It is to make him happy that?

P: Ehh

RA: What else shows that she is making him happy that a female must do to show that a male took the right decision or the right decision?

P: To show trust in the house

RA: alright how does she show trust?

P: Isn’t she wants him to go do this thing to circumcise

RA: Okay so a male if he says this thing of medical circumcision how must females support him?

P: The female must conduct herself well.

RA: What does it mean to conduct yourself well? How?

P: Like she must not wander around. Isn’t he wants to go circumcise medically. She must come home and treat him well till he heals

RA: Okay if she wanders around what will happen?

P: *Yoh* me in my culture they say when a person is from circumcision or from a doctor in the rural areas they say you as a female you must conduct yourself well because if there are things you are doing and coming home this one will be sick and rot

RA: What does it mean to conduct yourself well to a female?

P: You must stay in the house. Isn’t this one is there at the house is sick

RA: Why do you say it is the same as he is sick?

P: Like isn’t they say a person who is from circumcision is like a small child. Isn’t a small child you stay for three months, this one who is from circumcision you stay for six week until they heal

RA: Okay why do you have to stay for him?

P: They say you are mourning for him

RA: Why must you mourn for him?

P: Like if you go to work you must go out and come straight back home, not this thing of going to see so and so. They say you will come back home hot.

RA: Hot?

P: Mmh

RA: Now if you are hot what will happen to him?

P: He will rot there in front

RA: He will rot?

P: Mmh. Cause isn’t if you enter let us say we renting in a room alone and I come back and enter there to him.

RA: Okay right. Now sister we are finishing now with this activity, we will enter the second activity which I told you about. But can I please ask you whether there is anything else that you think we are leaving out which is important that we should talk about?

P: Isn’t they say to cut it starts at children who are ten years?

RA: Mmh

P: Let us say the schools are not yet closed now if they want to cut, they won’t be able to go to school if the finished cutting?

RA: If they cut when the schools are not closed? They can continue at school but I think that the information you can get at the doctor when you ask but they can continue going to school. Except if there is something important that can cause him to not go to school maybe there is something that is a problem there. Even there if they find difficulties they must come back here to the doctor and get checked for what is happening and examine the situation. Is there something you want to say?

P: No I am alright

RA: Now can we please talk about your group one, what is happening about group one?

P: Group one here is a married man who is afraid to tell his wife about circumcision

RA: What else, what other thing can you say about this group? Why is he afraid to tell?

P: He is afraid to tell the wife that he is going to circumcise

RA: Why is he afraid?

P: That he is old. He sees that he is now old

RA: Okay what else can you say about this group?

P: The man says what will his family say when he comes back from circumcision so old

RA: What else again?

P: That is it
