# Supplementary material for: “To speak or not to speak”: A qualitative analysis on the attitude and willingness of women to start conversations about voluntary medical male circumcision with their partners in a peri-urban area, South Africa
Source: PLoS One. 2019 Jan 25;14(1):e0210480. doi: 10.1371/journal.pone.0210480 (PMC6347244; doi:10.1371/journal.pone.0210480)
Supplement: S1 File — (ZIP) [file pone.0210480.s003.zip › QF017_QC2.docx]

PARTICIPANT ID QF017

RA: Ok so as we discussed that we are going to record do you still agree that we can record as we discuss

P: yes I agree

RA: ok then urh… as I explained that we are going to do three things today so the first one, I’m going to ask you questions and then you going to answer according to your understanding. There is no right or wrong answer so urh… you can feel free as we discuss

P: ok

RA: but then before we start can you please tell me a bit about you, anything that you can tell me about you

P: (P laughs) about? Concerning circumcision?

RA: anything that you can tell me

P: (P laughs) yah ne… ok im… im a person who loves to know so when circumcision came to my ears I said to myself I want to know more about it

RA: ooh ok

P: mmm then and… it is important truly speaking compared to the one at the mountain that I grew up knowing it exists

RA: yah

P: so I see its going to make a lot of people live too much

RA: mmm

P: yah

RA: so the first time you heard about circumcision where did you hear about it?

P: I first heard about it at the clinic and FM but too much I saw it on this other taxi advert to Kempton but I don’t remember well, yah they showing some cartoons and… it’s a logo and then they showed their contacts and that you can sms, whatsapp what what, then that’s where I started seeing it.

RA: mmm

P: but I didn’t have… what can I say.. I didn’t have time to come here and know more but then a lot of time I go to the clinic I get some pamphlets, I take them and read them, that’s when I saw that this is important not only for somebody else but for me as well to know about it how it can protect me besides that a person can do it alone

RA: mmm

P: yah even to say… cause I have someone I’m involved with

RA: ok

P: so I had a lot of interest even on…

RA: ooh

P: that on me how can it fit if he does this thing of male circumcision

RA: ooh ok but what is it that you know about… like about circumcision

P: (P laughs) urhm… what I know about circumcision is what I read that they cut their foreskin

RA: mmm

P: which is something that we didn’t know, I didn’t know all this time because it was a secret during those times of the mountain

RA: yah

P: so now I knew that (RA clears throat) they cut that foreskin and when they have cut that foreskin it reduces the chances for a person to have… other sicknesses on the site

RA: mmm

P: especially sexually and those ones

RA: yah

P: urhm… I think that it helps especially when you do it at the clinic because… there is checkups that they do for you and even before you do it

RA: yah

P: you go having an understanding of what happens before and after

RA: mmm

P: yah

RA: mmm

P: and another thing

RA: yah

P: it’s that as like me I have a male figure the chances that I can get sick via the thing… what do they call it? Eish I don’t know how to put it cervical cancer

RA: ooh ok

P: yeah cause I heard that it helps with that too it reduces urhm… the chances that you can get that cervical cancer

RA: ooh

P: yah the womb something, the womb mouth or that something

RA: ooh ok so you saying urh… ok you talked about when you were growing up there was the mountain only

P: mmm

RA: ok and then now you know the clinic one

P: and the clinic one

RA: is there any other that you know except the two

P: aahi… I only know those two

RA: mmm

P: yah and I prefer the clinic one

RA: the clinic one

P: because its safe the one at the mountain a lot of people were dying

RA: mmm ooh ok

P: it has a high death rate so the clinic one since it was there I have never heard that someone, there is someone who died

RA: ooh

P: yah so I think it’s safe

RA: so when you say the mountain one was a secret, what was it that was made a (P laughs) secret?

P: it’s a secret because the first thing

RA: yah

P: it was not done at the village it was done at the mountain

RA: ooh

P: and it was not allowed to get near the mountain

RA: mmm

P: there was… like before they go to that mountain, they choose a place and then they come back to the village and tell the elders anyway then we will be told that hey don’t go to this place there’s an initiation

RA: ooh

P: so stay away from this place so it was a secret, you would see people going there then other people come back then the next thing one of them is not there then we know ooh it means he was eaten by that thing so it’s a secret because we didn’t know what they do and even if you ask they wouldn’t tell you

RA: ooh so even when you ask they don’t tell you

P: they won’t tell you, they will tell you it’s men’s thing that’s all

RA mmm

P: and again you should introduce… the female one

RA: the female one?

P: yes cause I see male male male so what about females it seems you have put them aside

RA: ooh you think there should be a female one?

P: yes the female one is there at the mountain, the female one is there. The female one they don’t do it at the mountain they do it at the village, we just know that at so and so place theres that initiation and we know that women go there to do what there, women I also don’t know what the do

RA: ooh there’s a female one?

P: yes there’s a female so you should also do it here (both laughs)

RA: so the female one you also don’t know what is done

P: mmm it’s a secret its only the ones that went there that know

RA: ooh so you think there should be a female one here at the clinic as well

P: a female one mmm

RA: ok (both laughs)

P: so we know what is done at that school

RA: mmm but just thinking the one done there whether its for females, males and the clinic one how do they differ? Or how are they similar?

P: urh… eei they are not… not the same just that the purpose is one to remove the skin but…

RA: yeah

P: a big difference is that urh.. the clinic one

RA: mmm

P: they consider things too much about your health before they do it they must know your status on where you stand and then even after and then the clinic one… the mountain one as long as a child says he wants to go, he goes no matter how he is or not that is why death is a lot on that side

RA: ooh

P: mmm so I think difference they differ there, a person’s health being, wellbeing they don’t consider it on the site of the mountain

RA: ok but when you say well being what are the things you talking about?

P: a person can go there, you find that he is sick

RA: ok

P: and when he is sick it’s the thing that haai because its winter maybe its flu without being checked, a person can go there while sick like now there is sicknesses like HIV what what, he goes there, even them they don’t know that this person is sick

RA: mmm

P: so it becomes a risk too much amongst all things and others go because they don’t know what is done there then through themselves there then the mountain one when they say… once you get there you don’t come back, if you get there and decide that you don’t want to do it anymore, you go there you gone, you have to do what is done there so you can come back

RA: ooh when you there…

P: when you get there, net, you get there you will come back with them when they come back (RA laughs) so that is the thing is not right there so here at least at the clinic you can … they counsel you, they give you counselling first

RA: yah

P: and then you feel if you agree with it or not then if you agree with it you continue and then if you don’t agree with it haai there is no problem so this one of the mountain no

RA: ooh the mountain one they don’t give you options

P: they don’t give you options and I remember my younger brother he went because his friend went and if he went while he didn’t, when he comes back he wouldn’t be his friend anymore so he saw that its better he go and when he went… him going he didn’t know what is done there

RA: mmm

P: mmm so they bribed bribed there that haai when you go there you will come back a man you see so he said I also want to be a man he then went, so I think that a lot of people go there not knowing what is done there

RA: mmm

P: mmm so he just see when he arrive there that its like this and I cant go back

RA: mmm ooh ok so ok when you say your younger brother went because his friend went knowing that when he has gone and he hasn’t they will never be friends again, how does it work that when one has not went they don’t become friends anymore?

P: it was like that, it was like that because when I look at this thing that women, I have a friend of mine after going there, when she came back we didn’t get along well anymore they said haai she can’t stay with someone who has not gone for initiation

RA: yah

P: so every time when you sitting with them they call you names that you are a leshoboro things like that mmm so haai its aah aah

RA: what is leshoboro?

P: people… a person who is not circumcised

RA: ooh so when you have not went they call you names

P: they call you names its because when we are kids we talk about everything so you will know that this one in their family they don’t go for initiation cause this this of circumcision my brother for him to run away to there it was because at home they didn’t believe in it because they said we don’t use medicine at home

RA: mmm

P: so they can’t do that thing so but then this modern one I can say a lot of my siblings… there’s another one it’s not so long ago he went he… they agreed because they use… clinic things they are safer than herbs from the mountain where by you don’t know what it is they use

RA: mmm

P: yes

RA: what kind of medicine are you talking about?

P: aah… herbs isn’t they are different those ones use their own herbs and they have… its just that they are different we like… our believe we didn’t believe in those traditional things too much that they will protect you and so on

RA: mmm

P: you understand that when you go there isn’t they say this thing will heal you, this medicine they put on you will heal you what what

RA: ooh

P: so it’s the thing we don’t know what they have mixed in there

RA: mmm

P: mmm and another danger of it its that when you come back, you come back there sick and when you sick you have to go to the doctor now because they have finished, isn’t when they are done they are done they don’t care about you

RA: mmm

P: you see so even me it once happened with my younger brother when he came back from circumcision after like maybe two days it wasn’t getting right and when he urinated he said it was painful what what so we ended up saying lets take him to the doctor and when they took him to the doctor I don’t know what they said they didn’t cut well because we didn’t know what was done

RA: mmm

P: its them who has a light to say ooh… they do this and those wounds you have to… to say when you home how you take care of them like it wasn’t there on them that thing it was just for them when they were at the mountain ok we do this we do this so but when they get at home its like they are on their own

RA: ooh

P: so at least here… here at the clinic thing if there’s a difference you not feeling well or whatever you can come back and ask that this is happening but then there when they are done they are done they break their things they all spread and go different directions

RA: mmm

P: you don’t know who to follow and ask that this

RA: mmm ok but this thing of urh… when one has went and the other has not they are no longer friends was it something with a person or…

P: it was a stigma in a way its like the time when like when we first got introduced to HIV that when a person has HIV you would not be near them things like that so it was also like that, that the things you learned there

RA: yah

P: what are you going to talk about with this one because you have to be friends with people that you come from the same place with so that you can agree on that thing

RA: mmm ooh it was not something within a person that I don’t want to be…

P: aah aah it was from… it was from people like it was a community thing that aai if you are not from eeh eeh we don’t sit with you if you are from we can accommodate you

RA: mmm ok but is there any other reason that you thing that maybe urh… people who come from one community but there are other families that don’t believe in circumcision and others believe, for a difference to be there in people from the same community

P: mmm there is a difference

RA: mmm

P: as I said that at home we didn’t believe that thing of…

RA: yah was it because of the herbs only?

P: it was a reason of believe only that they use medicine we don’t know so we cant do it there so better this clinic thing has helped

RA: yah

P: it has helped because the elders back home that I knew were able to go after

RA: yah

P: and then there was that thing to say when you are old like mostly it was boys until around 20

RA: mmm

P: when you go when you are old it was an embarrassment

RA: mmm

P: yes it was an embarrassment that you circumcising with kids so it was a force that every child… when a child is still young that when I grow up I will be right but they were going cause of pressure, it was peer pressure so to say that a person want to go when they are still young, its not their decision

RA: mmm

P: you going because when so and so goes I also can go or I have to go because my age is going to a level that I cannot but now they go when they are older because they know its privacy when they go they know that I go today then I’m done tomorrow I am ok… now… now I’m sure that when people come it’s because of information that a person got

RA: mmm

P: it’s not the same as the previous times that a person goes because he’s forced or something

RA: mmm so do you think that when it comes to age there’s a certain age that…

P: (P laughs) aah… the age thing aah… I think when it comes to age its determined by how a person feels

RA: yah

P: yah age I don’t think its important because it’s the same as like when you old, when I have a child and I see that yes I can take him there then I can take him then if you find that there is someone old like a teenage, teenagers love making their own choices

RA: mmm

P: so you tell him that so and so what is that, do you want to go or not he will make a choice, let it be that you tell him and you come with him to get counselling so he knows cause not that I started with something I heard its something I read

RA: mmm

P: so if he gets another idea from you, you who knows about all this things I think that he will make a choice that is right

RA: mmm

P: then he decides even if he doesn’t decide that same day, age I think that its from a person, when does he feel that he can go

RA: mmm

P: mmm yah age I don’t think it has an effect on that thing

RA: mmm when you… brother before he went did he talk to the family?

P: aai he didn’t talk to the family

RA: mmm he didn’t talk to the family

P: mhm… mmm it just appeared that he’s not there that aai that person said he’s going and cause we knew that in my family there is no one who is allowed to go we didn’t take it serious until that day he didn’t come back, we saw that his friend went it means he went so when they checked they found that he’s not around he left and when they got that side they don’t allow you to enter, an elder person for you to enter its only when you have a child that you brought just to leave him then go

RA: ooh others don’t enter

P: an elder person only enters when they have brought someone

RA: yah

P: aah aah you come to bring someone only and to say ok I sent kids I want to see if they are here only

RA: mmm

P: mmm so to say you coming to fetch someone there, if they have entered they have entered, there is no getting out, you get out when they say its done

RA: mmm

P: it’s their own boarding school

RA: so do you think your brother only went because of pressure that his friend went or do you think there was something else that…

P: he went because of his friend cause his friend they were close you would think that… have you ever seen as kids when you grow up as long as your gates are opposite so its his friend cause when he wakes up he goes that side and when that one wakes up he comes this site

RA yah yah

P: so they grew up together with that thing so mmm… *meskien* this one when he thought of losing his friend, I’m going to start another friendship who and at the street they will know that I didn’t go to circumcise, it’s what forced him to go

RA: yah

P: cause even at home he didn’t tell them cause he knew that its something wrong that he is doing but he wanted to satisfy his friend

RA: mmm so after you got information about circumcision is there any man that ever spoken to about it?

P: yah

RA: or you thought of telling about it

P: yah anyway I told a lot of people but I started with mine

RA: ooh ok

P: yah so he also got a bit shy then I told him that there is nothing to be shy about… he was shy about it cause thing thing is going to help him tomorrow with your life so he came, he came last year

RA: ooh ok

P: yah but then yah he’s right yah

RA: so he came… what was the reasons that made you think that you have to tell him about it

P: aah… isn’t I just saw it

RA: yah

P: they have written those things of yours so I thought haai because I see that home where we come from… so firstly I told him, I didn’t tell him that go I just told him that you know there is something in life we didn’t… we didn’t see that most things we are afraid of them because we don’t have knowledge

RA: mmm

P: so the reason you didn’t go for circumcision he told me that he ran away cause at his home there was… they did it at his home

RA: yah

P: so you would find that there are people dying and him being afraid of being cut its what he didn’t want so they forced him, when they forced him it cause him to run away from home to stay with his aunt somewhere else

RA: mmm

P: for him to be safe so he grew up with that thing to say he won’t go there so now when I got this thing I told him I said hey man why don’t you go to the clinic then, we had a lot of argument that maybe the clinic and the mountain they do the same thing

RA: mmm

P: mmm until we started getting information I came with pamphlets, there is a lot at home

RA: mmm

P: mmm I told him I said no man let’s try and see ok because at the clinic they give you counselling it’s the same as going for an HIV test they give you counselling before you do why don’t we go and see then

RA: yah

P: then I came with him then they counselled him but I didn’t get in cause I knew that (P laughs) he will be shy maybe when I’m there so he went for counselling alone, then when he came back he came back alright he said haai I think I’m going to go so I didn’t push him I just said when you ready you will tell me

RA: mmm

P: then he decided alone that he’s coming, he came and did it now he’s right

RA: mm ok can you please tell me about the experience of… the first day when you told him about circumcision (P laughs) how you told him and how he reacted

P: ok the first thing because I know what kind of a person he is, he’s very traditional so I just showed him those papers and said you know there’s some papers I came with from the clinic

RA: mmm

P: read them I saw they are written male circumcision what what so I think that because of it’s the clinic there can be something better that can happen there, he said haai, his reaction was the thing of saying its like maybe I undermine him or something cause he didn’t react the way I thought he would react cause he gave me that look of being surprised to say why must I do this thing so it’s when I told him that I’m not saying you should go

RA: yah

P: read and know and then you will decide if you don’t want, I also just took it to know what is happening so if you don’t go… you don’t agree with it you can leave it just…just read only

RA: mmm

P: then haai we read we read and plus they show pictures as well

RA: yah

P: yah you can see what is done so he said ok I will think about it then I will see if its ok so I just said ok if you are not ok with it you can leave it but then if you ok I will support you all the way

RA: mmm

P: mmm cause hes just… a shy person

RA: yah

P: yah this kind of things its not his things

RA: when you say hes traditional what do you mean?

P: he doesn’t want to be told

RA: ooh he doesn’t want to be told

P: mmm especially things on his body aah aah that is closed for discussion

RA: mmm

P: mmm even when he’s sick

RA: he doesn’t want to be told

P: yes unless he feels that he can not do anything its when he start to stand up then we go to the doctor, he doesn’t want to be told

RA: so ok and then when you say felt that he thought you undermine him what do you mean when you say undermine?

P: its like the way I tell him it made it look like hes a boy tat circumcision its for kids

RA: mmm

P: so I’m now an adult why must I do this thing

RA: yah yah

P: mmm cause he had that shyness at first when he came to ask me, when he was about to go there, what are they going to say? I was like stop saying they will say, what will they say? Go there and hear what he has to say before cause there when I read there was no age limit that they consider age and then cut from this age, they said its male circumcision if you want you can go

RA: mmm

P: then you will see there what happens so Im sure they will give you everything, counselling is there so it will be between you and that person on what you agree on

RA: what you agree on

P: yes so he took it as if it’s for small kids so it’s like I’m making him a baby now

RA: mmm ok but just thinking about it people who are in a relationship who do you think should start talking about circumcision?

P: (P sigh) mmm… here… this one… I don’t know I think it will depend on you as a couple like how open your relationship is because when you not open its not easy to tell someone and even him it will not be easy for him to tell yo, to start it especially if that person is old already

RA: mmm

P: its not the same as a small child because you just tell him that hey papa lets go to this place you see

RA: mmm

P: so when its an old person I think when you open both of you its simple

RA: mmm

P: yes cause even me I saw it, he didn’t start it, I saw it then decided that its better I tell him

RA: yah

P: I said it means if I didn’t tell him he was going to just sit, even now he would still be sitting cause there is some people they see something then after just read it then leave it

RA: yah

P: he doesn’t follow that this thing can also benefit me somewhere yes so I think just when a relationship is open everything is possible

RA: mmm so you saying when its open it can be any one of you

P: any one of us

RA: that can…

P: that can start because when you not open I will think that tjo how will I start

RA: mmm

P: then he will also ask himself that this woman, what is this person telling me now you see

RA: mmm

P: mmm it’s the thing that makes me say when you open and it depends how you tell the person

RA: mmm

P: you have to be careful even your tone don’t use that tone… it means a person you should tell him in a way that he can hear that this person is telling me this thing maybe she cares

RA: yah

P: not to maybe just tell him so that he feels small

RA: mmm

P: yes it’s not like there’s… isn’t that even us women there’s somewhere where we miss it a bit that because so and so has gone eeh eeh you should go too

RA: yah

P: it’s no longer support its just to say go

RA: ooh ok (RA laughs)

P: mmm so that goes with the relationship on how open you are

RA: ooh and then the tone

P: yah the tune, tone its important in a relationship, tone is important too much

RA: mmm

P: because tone is the one that can tell you if a person is angry or a person is just concerned… is the one that tells you a lot of things about a persons mood

RA: ok but what other ways that you feel that a woman should not use to tell a man about circumcision cause you mentioned that when you tell him about other men its out, what other ways do you feel a woman shouldn’t use to tell a man about circumcision?

P: mmm… yah others I’m not sure… I’m not sure just the one in my heart is that one that you shouldn’t tell him about other men

RA: ooh

P: yah just that you have to know your partner so you can talk to him cause yah when you are open you will know what he likes and what he doesn’t like

RA: mmm

P: yes cause you can’t do things in your house the way they are done next door

RA: yah

P: yah that’s what I see more important

RA: when you compare him with…

P: mmm cause he will feel that you comparing him with somebody else and which is not right

RA: ok but what ways do you feel a woman should tell her man using when telling him about circumcision?

P: urhm… when you got like information

RA: yah

P: the way you got it

RA: mmm

P: if you are shocked… you are shocked you give it to him the way it is not to give with… judging him

RA: ok

P: to say this is this because of I knew this thing I shout at him eeh eeh you tell him the way you also sure that you got it but you didn’t know so it surprised me also so I thought its important that I come and tell you because when you look at that thing you see that its useful

RA: yah

P: you know what its going to do to him so its better you tell him the way you feel then he can see that ok… because if like I also think that… if I didn’t tell him the way I saw it that say ooh there’s medical male circumcision and they give you counselling, or the checkups, or what what when I tell him, if I didn’t tell him that way I was going to just say hey you go to the clinic isn’t they have counselling and what what you will see when you get there so I started it from the bottom so when he came here he came with me, I gave him what I got from the books that talk about circumcision and what is done so I think it’s the way you should talk to him, just your tone

RA: mmm ooh so you feel that a woman should have a bit of information before talking to her man

P: a bit of information even if you can come and say I heard… isn’t a lot of things we know them by hearing them from others or I came to the clinic and then I got something, here is a paper it says this

RA: mmm

P: yes isn’t when you have all that information so it becomes ok why don’t we go together and do that 1, 2, 3 you see how much this can help us yes even that way I’m preparing that even tomorrow when he’s walking around and see something that he can see that it will help his woman he can bring it back home mmm and not only to say a woman or man even kids at home it’s important that thing to know how you going to talk to your child

RA: mmm

P: if you call him as if you sending him to the shops hey go buy me bread, you call him; sit down with him so he knows why he is supposed to do that thing

RA: mmm

P: mmm and then it can be his decision too to say ok I will do it or what

RA: ok but what other reasons can you give a partner when you tell him about…about circumcision to say why he should do it? What reasons do you think can be given?

P: to say the first thing its going to help him cause what i… what I heard is that that foreskin especially during intercourse its able to hold a lot of things inside so they end up making you sick, himself and that sickness obviously he’s going to bring it to me

RA: yah

P: let’s say we not using protection it comes to me you see and then again and… i… what do they call it… a something cancer for men this one pine whatever

RA: urh… prostate

P: yah that one also it will save him there

RA: mmm

P: you see so its that thing I think its right for when you want to tell him to do that thing

RA: mmm you can tell him about those things

P: yes

RA: ok but a child what do you tell him about?

P: urm… a child a lot of times I (P laughs)

RA: yah

P: its just that I have never experience it but I think to me it can be my choice to take him there for him to come cause I think a child most of the time a lot of things don’t … hes not concerned about them so it’s up to me as a parent to come register him, tell him that my child you doing this because there’s sicknesses out here, cancer is there it can enter you, here when you bath you can bath well without anything disturbing you cause that thing is like a wallet

RA: mmm

P: it’s a bag and then it holds things, you will tell yourself that you have bathed and you ok tomorrow you find yourself sick without knowing how the sickness came about

RA: mmm

P: yah

RA: ok…ok but urh... Ok in your relationship you started telling your partner right?

P: mmm

RA: if it him who started telling you that he is thinking of doing it, how would you take it (P laughs)? In which way

P: aah… it goes with his history when we I met him I wouldn’t… I would be happy I wouldn’t be surprised because… I told myself that ok at his age

RA: mmm

P: and he has not went to circumcise it’s what I was wondering how come cause a lot of people when I was growing up it was there… especially to men it was important too much that I want to be a man, each and every person was saying I want to be a man how did it come up in his case so when I started hearing his history then I said ooh ok but I think that I was going to support him one way if he came up with it

RA: ok but how would you take him? Like when he started (P laughs) talking

P: haai I was going to just take him right as much as I was able to ask him in the first place about how it came up

RA: yah

P: mmm I wouldn’t have a problem with it

RA: mmm

P: cause I had a problem with… when I started seeing that… ooh my God this person now what’s happening so I stayed with it but then when time went on I started to ask that’s why I said when a relationship its open it becomes simple to… that’s why I said some other times there’s no secret in a relationship

RA: mmm

P: so I held it to myself until I saw that aag man this thing is worrying me and this person let me tell him haai let me tell him cause I… where I come from I know that everyone what’s to feel that he is a man but to be a man he has to go to the mountain

RA: mmm

P: that ok when a person is from the mountain you have to show him that respect to say eeh… that person is from the mountain, circumcision otherwise you have to respect him and they change their names isn’t it’s what used to boost them a lot, a person leave as {} (name of person) when he comes back he’s no longer {} (name of person) it’s so and so urh… people why and they took it serious that thing cause if you call him {} (name of person)

RA: yah

P: he wouldn’t answer, if you younger than him you get beaten you see (RA laughs) to say you undermine me so he would beat you until you no longer call that name you will call him with the name they gave him there, you are a man now you no longer {} (name of person)you are so and so

RA: ooh they give him that name because he’s now a man

P: he’s a man he no longer use {} (name of person) that one it’s for boys so he’s a man he uses… so you call him by it not knowing you will feel a slap then when you ask and then whats the problem, because he changed his name now he’s a man so It’s what they used to feel so I also asked myself that, I told myself that maybe we are from different cultures, I found that we are different too much but why didn’t he do it cause in his belief its there so I realized after

RA: mmm

P: but I don’t think that I would have a problem

RA: what culture is he from?

P: he’s Ndebele

RA: ooh

P: mmm

RA: do they do it in his culture?

P: in his culture they do it and in Ndebele culture they do it while you still young

RA: mmm so but do you think culture has an influence in…in a man’s decision to circumcise or not?

P: yah but now I don’t see its importance that much anymore but those previous times it had a lot of influence it… cause when they did it, it was like Christmas

RA: mmm

P: especially when you are from the initiation it was like Christmas you would hear that there is this thing you feel at the village, there’s a huge thing and that thing was a…a… it killed them too much to say like they did it around June

RA: mmm

P: when kids went there they didn’t go to school

RA: yah

P: they miss a lot of things so at least the clinic one now you go today and make an appointment today then they do it maybe… it will depend whether you heal fast or not

RA: mmm

P: yah then its right than to say you miss class… with too much time cause when they go in June they come back in July

RA: ooh ok

P: so they stayed there for some time, he will stay there you would even forget that he exists you will just see that ooh there’s someone who was supposed to be here yah so it’s right cause they do it today when you done then you leave then if there’s other complications

RA: yah

P: that need attention you go to hospital to check whether you right or what then you come back

RA: mmm

P: so that one you stayed there until I think that was one of the things that increased death rate

RA: mmm

P: to say when a person is sick they don’t care that… they tell themselves that its…its flu or maybe because we cut him what what he will heal, he will heal

RA: mmm

P: so you will find that they know what is making him sick

RA: mmm

P: mmm

RA: ok so but when you say a man is from there the ceremony was like a Christmas who was responsible for organizing the ceremony? Was it the family or commu…

P: family… it was done by the family and because this thing is for the community it becomes like a whole

RA: mmm

P: yah it becomes a thing… of… for cause when they come back they come back in one day and each and every one will ooh my God people spend, a cow falls, they buy you a new blanket

RA: yah

P: and then if there is money at home they build you your own room, it means you are now a man you don’t enter… even when we enter your room we have to choose that ok you stop at the door and talk him at the door and then go out, its only his mother or grandmother who cleans, you don’t just enter

RA: (RA laughs)

P: isn’t that he is now a man

RA: ook…ok what did they mean by a man? When they said you are now a man

P: it means you have grown, its the way they say it that is why I said…

RA: yah

P: age was important those times to them that you cannot go when already at this age because of mostly you find it full of kids then you find that I am the only adult so I think even others that come like now it becomes a thing of pressure of age that forced them not to go its better I don’t go cause what am I going to do where there is kids you see

RA: mmm

P: cause I heard that like when they are at the mountain they are naked

RA: ooh ok

P: you don’t wear, you don’t this and you are together there sitting together so it’s what I think that it cause them to step back that I can’t do this

RA: mmm

P: yah but now I don’t think that age and culture noo I don’t think that they do anything now cause a child has his own choice so it’s up to a parent whether he/she agree or not when they talk

RA: ok but do you think are the benefits of… of circumcision to people who are partners

P: the benefits is that

RA: mmm

P: when sicknesses come

RA: yah

P: when they come cause is not only about a man

RA: yah

P: even a woman isn’t that thing has an effect that happens in-between so the moment he goes for circumcision it reduces the chances that a sickness can enter and that… sex becomes like that

RA: yah

P: The skin hurts especially when it comes to woman its not the same when there is no that foreskin

RA: mmm

P: yah

RA: ooh ok

P: so it’s one of the benefits that when the foreskin is removed it becomes simple

RA: mmm

P: so sex becomes simple whereby you don’t get hurt by the skin so where it ends isn’t it pushes you, when it pushes you it hurts…

RA: mmm

P: sometimes it causes some scratches around the vagina

RA: mmm

P: yah so I think that there it also… it helps there besides reducing…

RA: ok but do you think circumcision is a good idea or not just thinking?

P: (P laughs) circumcision ok you see if they ask me to choose between the mountain and the clinic one

RA: yah

P: I take the clinic cause it’s safe

RA: mmm

P: that one I’m sure of that its safe so considering that clinic circumcision I think it’s right just the mountain one i… I wish they can stop it like complete

RA: ooh the main thing is safety

P: mmm the main thing is safety, what’s the point in taking your child that side and then hope that I wonder if mine will come back, when they come back and yours is not there maybe he will come a bit later

RA: yah

P: you already afraid that it means he’s not coming back

RA: mmm

P: so it’s not ok so... and you see back then what they used to do

RA: yah

P: back then not back then even now they still do it cause the mountain is still there

RA: mmm

P: circumcision at the mountain urh… when you take your child there isn’t it they give them… you have to give them a blanket like when you go, he goes there wearing it then when you get that side then he undresses, he has to be naked

RA: mmm

P: then they give him a blanket, the blanket he is going to use it when he sleeps

RA: yah

P: and they treat him that side the way I heard, they don’t treat them like… cause like you have to work

RA: mmm

P: to show that you are a man

RA: ooh

P: and like when you sleep there is time that they have to wake you up just because you are there… you are going to become a man so they say they are treating you

RA: ooh

P: mmm so that is the thing that makes me think that the mountain one if they remove it then it will be better

RA: mmm

P: so who are you going to ask now that they have dispersed

RA: mmm

P: mmm so no the clinic one is the one that is safe so if they say I must choose there I will go for the clinic one

RA: mmm ok well but urh… I think… we are done with this part of… of this thing unless if there is something else that you feel we did not talk about

P: yoh I think we are (P laughs) done
