# Supplementary material for: “To speak or not to speak”: A qualitative analysis on the attitude and willingness of women to start conversations about voluntary medical male circumcision with their partners in a peri-urban area, South Africa
Source: PLoS One. 2019 Jan 25;14(1):e0210480. doi: 10.1371/journal.pone.0210480 (PMC6347244; doi:10.1371/journal.pone.0210480)
Supplement: S1 File — (ZIP) [file pone.0210480.s003.zip › QF018_QC2.docx]

**PARTICIPANT ID:** QF018

**FACILITATOR:** Eh, thank you for taking part in our project ma’am, will you please give me permission to record our conversation.

**PARTICIPANT**: Yes, there’s no problem. I give you the permission.

**FACILITATOR:** Ok. So, ma’am may you tell us a little about yourself. Where do you live ma’am?

**PARTICIPANT:** I live here in {} (participant address).

**FACILITATOR:** You live here in {} (participant address)?

**PARTICIPANT**: Yes. {} (participant address).

**FACILITATOR:** ok so Do you have any children ma’am? Who do you stay with?

**PARTICIPANT**: I live with my parents but I have two children, a boy and a girl.

**FACILITATOR:** A boy and a girl...so how old is the boy?

**PARTICIPANT:** He is nine.

**FACILITATOR:** He is nine years old? Okay. Eh ma’am would you like to explain your understanding of male circumcision? Could you perhaps explain what is it that you know about it?

**PARTICIPANT:** What I know about circumcision is...it’s when a male person...

**FACILITATOR:** Mhhm.

**PARTICIPANT**: ...he might be going the medical or traditional route...

**FACILITATOR:** Yes.

**PARTICIPANT:** ...he’ll be going there to get the skin of his private part removed, the one we call the foreskin.

**FACILITATOR:** Yes.

**PARTICIPANT:** Yes. From a traditional perspective we say a person is growing up...

**FACILITATOR:** Yes.

**PARTICIPANT: .**..that he has grown up, he is now a man, he will be responsible.

**FACILITATOR:** Yes.

**PARTICIPANT:** And then when it comes to the medical side, it’s there so that...it really helps with reducing...eh, chances of contracting diseases, especially those you contract sexually.

**FACILITATOR:** Oh! So, what you’re saying is when a person is...when they say they’re talking about circumcision, you are saying a person removes the skin...which is found on his private part?

**PARTICIPANT:** Mm.

**FACILITATOR:** Oh! Okay. I hear you telling me about eh the traditional route and another route, how many types of circumcision do you know?

**PARTICIPANT**: I know the traditional one and the modern one.

**FACILITATOR:** the traditional one and the modern one

**PARTICIPANT**: mmm

**FACILITATOR:** Yes. Then what happens in the modern one? What is done medically?

**PARTICIPANT:** What? Procedure?

**FACILITATOR:** Yes.

**PARTICIPANT:** Eh, maybe I’d be more knowledgeable if this child of mine had reached the age to go. But, according to books, when we read and hear, eh, it’s said that the procedure...you make an appointment right...

**FACILITATOR:** Yes.

**PARTICIPANT:** ...they give you a date, when you arrive there, they offer you counselling. They explain to you…

**FACILITATOR:** Mm.

**PARTICIPANT:** …about what is going to happen and ask you if you agree to get tested for HIV...

**FACILITATOR:** Yes.

**PARTICIPANT**: ...a blood test will be done, then you will enter into a room, I don’t know if it’s an observation room or what it is for.

**FACILITATOR:** Yes.

**PARTICIPANT:** That’s where you’ll be attended to by the doctors, they will inject you with injections...

**FACILITATOR:** Okay.

**PARTICIPANT**: ...for reducing pain, so that you don’t feel a lot pain. From there on, the procedure begins.

**FACILITATOR:** Okay.

**PARTICIPANT:** And then, from the traditional side I don’t really know, I just hear that they go to the mountain...the importance...you are attended to by old men, from what I hear...

**FACILITATOR:** Yes.

**PARTICIPANT: …**They’ll first tell you about what will happen...

**FACILITATOR:** Yes.

**PARTICIPANT**: ...how it will happen, but I don’t know how the procedure is done as it’s not being written down in books and we don’t read about it.

**FACILITATOR:** Mm, Mm. Oh! So, if you are to know about the procedure performed...

**PARTICIPANT:** Mm, perhaps if even in the traditional side if they’d also write it down in books and we’re able to read about it, we’d be able to understand about it.

**FACILITATOR:** Okay, so, I heard you say when a person goes to the mountain to get circumcised, it’s...eh it’s said that he takes, he grows up, in what way does he grow? Can you explain it to me?

**PARTICIPANT**: Ey, I’d don’t really know but it’s what we grow up hearing.

**FACILITATOR:** Mm.

**PARTICIPANT:** They say when you’re from there you are now grown, you’re a man, you are responsible.

**FACILITATOR:** Mm.

**PARTICIPANT**: When boys are hanging out by themselves and there’s a boy who is uncircumcised, they won’t regard him as a man...

**FACILITATOR:** Mm.

**PARTICIPANT:** ...they say he can’t tell them anything, he is uncircumcised, he has not went through circumcision.

**FACILITATOR:** Okay, so if a person is...if he hasn’t went through circumcision, the mountain one, and when he has went there, is there any difference from your point of view, between a person who hasn’t been there and when he is from there?

**PARTICIPANT**: Ah, I can say there’s no difference.

**FACILITATOR:** Mm.

**PARTICIPANT:** Because, when you arrive at the mountain, the old men will tell you, we don’t know what they tell them right...

**FACILITATOR:** Yes.

**PARTICIPANT:** ...it’s their secrets at the initiation schools...

**FACILITATOR:** Yes.

**PARTICIPANT:** They tell them whatever they tell them...a person comes back behaving in the same way when he was, when he hadn’t went there, still behaving like a boy. Perhaps they tell them that you’re a man and you should behave in this manner, you’ll be responsible, but when a person comes back he comes back as the same irresponsible person he was.

**FACILITATOR:** Mm.

**PARTICIPANT**: So, I don’t know how the traditional side, at the mountain...

**FACILITATOR:** Mm.

**PARTICIPANT:** ...is helpful or the...what do they call it? The advices they give to them...

**FACILITATOR:** Yes.

**PARTICIPANT:** …you see.

**FACILITATOR:** Okay.

**PARTICIPANT**: It’s like it’s difficult to talk about it, because you don’t know about it, you don’t read about it, you just hear when it’s being talked about.

**FACILITATOR:** Mm. So, I hear you saying that they say...you’re saying it’s a secret, why do you think they say it’s a secret?

**PARTICIPANT:** Ey, the fact that it’s a secret...according to me?

**FACILITATOR:** Mm.

**PARTICIPANT**: When we read, when we hear news stories a lot of the times about circumcision during winter time...

**FACILITATOR:** Yes.

**PARTICIPANT:** ...you find that there are many children who die at initiation schools...

**FACILITATOR:** Mm.

**PARTICIPANT**: ...I think the secret is...they made it a secret so that those who haven’t been there as yet shouldn’t know...

**FACILITATOR:** Mm.

**PARTICIPANT:** ...what happens, so that they always have a desire to also go there...

**FACILITATOR:** Okay.

**PARTICIPANT**: Do you get the point?

**FACILITATOR:** Mm.

**PARTICIPANT:** They’ll find out what happens while they are there, those who have been there shouldn’t tell them...because if those who have been there tell them that it’s like this, it’s hard, it’s like this, the next generation that should go there will be scared to go. So, they will find out for themselves when they get there.

**FACILITATOR:** Oh! So, you mean to say that it’s kept as a secret because they don’t want these ones to know that...other people to know that...to know what goes on there?

**PARTICIPANT**: Yes, you’ll know when you get there.

**FACILITATOR:** Okay, I hear you saying some...die there. Perhaps you can tell me what is it that might cause people to die?

**PARTICIPANT:** I think that...circumcision can be performed by any old man who deems himself fit to open an initiation school...

**FACILITATOR:** Mm.

**PARTICIPANT: .**..he isn’t a traditional healer...

**FACILITATOR:** Yes.

**PARTICIPANT:** ...he is just a person, he just opens that school of his, without any knowledge...

**FACILITATOR:** Mm.

**PARTICIPANT:** ...and when they are in the mountains, there’s nothing which they give people, or a pill or whatever that will help a person when he is in pains. I think some of them die from pains. (knock on the door, clicking sound)

**FACILITATOR:** Okay. Mh, ah. Here, I’ve also heard you say that in medical circumcision, people are tested, they are injected. Can you tell me the reason behind them getting tested, eh, what could it be?

**PARTICIPANT:** I think that, so that they might know the state of their...

**FACILITATOR :** Yes.

**PARTICIPANT:** ...health, their HIV status.

**FACILITATOR:** Yes, yes**.**

**PARTICIPANT:** As we know that when you’re HIV positive...

**FACILITATOR:** Yes.

**PARTICIPANT:** ...there’s a CD4 count that is expected of you to have so that you have enough strength to be able to do certain things.

**FACILITATOR:** Yes.

**PARTICIPANT:** Like when you are starting treatment, they have to take you right…

**FACILITATOR:** Yes.

**PARTICIPANT:** ...blood so that they see, eh, if your immune system will be able to receive that treatment. So, I think the reason that they should test you in here, eh, when you’ve come to do a medical circumcision...

**FACILITATOR:** Yes.

**PARTICIPANT:** It’s so that they may see what you CD4 count is. If it’s 350..

**FACILITATOR:** Yes.

**PARTICIPANT:** …will you be able to get circumcised? Because if your CD4 count is low...

**FACILITATOR:** Yes.

**PARTICIPANT:** …you’ll be injected...I mean you’ll be cut...

**FACILITATOR:** Mm.

**PARTICIPANT:** ...there’s a possibility that you might not heal faster.

**FACILITATOR:** Oh! Yes, yes.

**PARTICIPANT:** Or you might bleed heavily to the point of having complications.

**FACILITATOR:** Mm, mm. And I also heard you saying that they prick you with an injection. This injection they prick you with...what is it for?

**PARTICIPANT:** I think it’s an injection for when they are busy with the procedure that you might not feel a lot of pains.

**FACILITATOR:** Yes.

**PARTICIPANT:** ...in that way, it’s for minimising pains.

**FACILITATOR:** Oh, it’s for minimising pains, mm, okay. Have you ever thought of telling one of your family members or your partner about circumcision?

**PARTICIPANT:** With me, at home...

**FACILITATOR:** Yes.

**PARTICIPANT:** ...we’re Christian**.**

**FACILITATOR:** Mm.

**PARTICIPANT:** Since we’re Christian, my father, from what I see, my brother as well...

**FACILITATOR:** Mm.

**PARTICIPANT:** ...they’ve never been there**.** And then I have siblings boys, one was born in 1995 and the other in 1997.

**FACILITATOR:** Yes.

**PARTICIPANT:** The situation is, my father still has that mentality that from a church’s perspective we don’t do such and such. I sat him down and explained to him about HIV, I explained everything to him. He didn’t want….he didn’t understand at all.

**FACILITATOR:** Yes.

**PARTICIPANT:** I explained to him that getting circumcised, helps...how it was going to help the children.

**FACILITATOR:** Yes.

**PARTICIPANT:** Especially when we come to the issue of HIV because it’s something that we are living with, it’s everywhere...

**FACILITATOR:** Mm.

**PARTICIPANT:** ...to ensure that the children are safe…

**FACILITATOR:** Mm.

**PARTICIPANT:** ...let’s take them for medical circumcision since there’s one here in {} (clinic address). Let’s use of the services we have.

**FACILITATOR:** Mm.

**PARTICIPANT:** He was stubborn at first, but in the end he agreed, because I explained to him until he understood. I brought him pamphlets, he read until he understood. As I speak now, my siblings...these siblings of mine have done it, one did it this year in June, one did it in...eh in March.

**FACILITATOR:** Oh! So, when you went to talk with your father, how was it like for you?

**PARTICIPANT:** For me?

**FACILITATOR:** Yes, like was...was it just easy for you to talk to him?

**PARTICIPANT:** Yes. It was easy because this thing...especially since we have this medical male circumcision here in {} (clinic address)...

**FACILITATOR:** Yes.

**PARTICIPANT:** It’s something which he sees.

**FACILITATOR:** Mm.

**PARTICIPANT:** You see, at first we were unable to talk about it. There was nothing pushing us to talk about circumcision, especially medically because we knew that when you’re supposed to take a child to get circumcised medically or…you have to pay up a lot of money.

**FACILITATOR:** Mm.

**PARTICIPANT:** Some will tell you...we weren’t able to do our own research about how much it costs to go for circumcision at the doctor, people will tell you that it costs a lot of money. So, we did not go after that issue.

**FACILITATOR:** Mm, mm.

**PARTICIPANT:** But ever since the arrival of {} (clinic address) male circumcision...

**FACILITATOR:** Yes.

**PARTICIPANT:** They say it’s for free.

**FACILITATOR:** Mm.

**PARTICIPANT:** Then why not? Go and hear what is required…what is required.

**FACILITATOR:** Mm.

**PARTICIPANT:** Then when I went to talk to him it was easy because perhaps the first issue was the money factor but he did not want to say...

**FACILITATOR:** Yes.

**PARTICIPANT:** ...that “no, I won’t be able to take out any money.”

**FACILITATOR**: Oh!

**PARTICIPANT:** So, when I was talking to him it was easy.

**FACILITATOR:** Oh! So you think that reasons of...eh money is one of the things that make people to not to go get circumcised?

**PARTICIPANT:** Yes.

**FACILITATOR:** Okay. So, does he perhaps ever think about going to get circumcised himself?

**PARTICIPANT:** I don’t think that he thinks about doing so, perhaps he is considering his age because he is 70 years old now.

**FACILITATOR:** Yes.

**PARTICIPANT:** And then I talk to my mother about a lot of things.

**FACILITATOR:** Yes.

**PARTICIPANT:** When I talk to my mother...there are people who are born being partially...

**FACILITATOR:** Mm.

**PARTICIPANT:** ...circumcised naturally. You find that a person was born with a foreskin which isn’t big...

**FACILITATOR:** Yes, yes.

**PARCTICIPANT:** ...it’s as if it was cut but partially.

**FACILITATOR:** Yes, yes.

**PARTICIPANT:** So, when I talked to my mother, explaining to her about circumcision she told me that “you know, I am surprised, because your father...never did that thing...

**FACILITATOR:** Mm.

**PARTICIPANT:**  ...he was never circumcised the traditional way, he was never circumcised the medical way but he is like this”. That’s when I explained to her, also bringing her a pamphlet, there are pamphlet there at MMC [inaudible].

**FACILITATOR:** Mm.

**PATRTICIPANT:** And even books do say that there are people who were born being partially circumcised.

**FACILITATOR:** Yes.

**PARTICIPANT:** So, my father is like that and I don’t think that to him there comes a point in which...to an extent that it’s a push because he believes that he was born that way, he’s fine, he’s old, he’s not going anywhere, he’s focusing on his wife, you see?

**FACILITATOR:** Yes.

**PARTICIPANT:** He no longer has a problem. But then for his children, he felt that they may get circumcised.

**FACILITATOR:** So you think that his reasons are that he was born being that way, being partially circumcised? He also says his age is...he is also considering age?

**PARTICIPANT:** Yes. When I tell him about the issue of HIV he says “where will I get HIV from because I no longer do those things and I am staying with your mom I have children. So, it’s the same.”

**FACILITATOR:** So, those are his reasons for not getting circumcised?

**PARTICIPANT:** Mm.

**FACILITATOR:** Okay. So, talking to your siblings since you said you’re Christian…how was it? Was it something they wanted? Or eh did you first bring up the issue about why they should go?

**PARTICIPANT:** It was something they wanted, it’s just that maybe it was hard for them to talk to my father because they’re still small boys.

**FACILITATOR:** Yes.

**PARTICIPANT:** It didn’t happen that when I told them...they never gave me a problem, I didn’t waste any time, when I told them they said okay and they made bookings and came.

**FACILITATOR:** Did they already know about circumcision?

**PARTICIPANT:** They already knew. I think that when they are hanging out with their peer groups...

**FACILITATOR:** Mm.

**PARTICIPANT:** It’s things which they talk about. And then it puts...it puts pressure on them...

**FACILITATOR:** Mm.

**PARTICIPANT:** ...that so and so has done it and he is my age and I haven’t done it as yet.

**FACILITATOR:** Oh! So they were now being pushed by the fact that their friends have already underwent circumcision?

**PARTICIPANT:** Mm.

**FACILITATOR:** Okay. What is it that a person should not talk about…what a person should avoid when telling a male person about circumcision?

**PARTICIPANT:** I don’t understand.

**FACILITATOR**: I mean, when, when you want to talk to a male person about circumcision, who is old, what should you make sure you don’t touch or you don’t talk about? What is the way in which you should talk to male people about it?

**PARTICIPANT:** I think that when you’re talking with male people, eh, the manner you will use or approach...

**FACILITATOR:** Mm.

**PARTICIPANT:** ...it should be one of respect, you should respect them.

**FACILITATOR:** Mm.

**PARTICIPANT:** Whatever you’ll say, you should say it with respect and thinking...you should be very sensitive...

**FACILITATOR:** Mm, mm.

**PARTICIPANT:** Because you can’t just say “hey, go and get circumcised, your age mates have been circumcised”. You will give them reasons...

**FACILITATOR:** Yes.

**PARTICIPANT:** The advantages of circumcision. That, when you have done this...

**FACILITATOR:** Mm.

**PARTICIPANT:** ...this will happen. Then they won’t have a problem.

**FACILITATOR:** Mh, mm mm. So, you’re saying you shouldn’t...so, you should be mindful of what you are saying?

**PARTICIPANT:** Yes.

**FACILITATOR:** So, how should you begin the conversation with them perhaps? What’s the way in which you should start talking… [inaudible tell them?]

**PARTICIPANT:** I think that when you first talk to them, you should ask them how they feel about this issue.

**FACILITATOR:** Yes.

**PARTICIPANT:** How they understand it, you see?

**FACILITATOR:** Yes.

**PARTICIPANT:** How they perceive it.

**FACILITATOR:** Mm.

**PARTICIPANT:** Then, after you have asked them and they’ve responded you’ll be able to hear that these people still hold the belief that this thing shouldn’t be done...

**FACILITATOR:** Mm.

**PARTICIPANT:** ...or they believe in it. If they’re of the view that well this thing is not important...

**FACILITATOR:** Mm.

**PARTICIPANT:** ...that’s where you’ll show them its importance.

**FACILITATOR:** Okay. So, what are the ways which you might begin…apart from talking? What are the things you can do to bring the issue of circumcision at home? Or to a person who doesn’t want to get circumcised?

**PARTICIPANT:** I think the pamphlets...

**FACILITATOR:** Mm.

**PARTICIPANT:** ...if they’re there in the house, when you read them...you just strike a conversation about it, especially with the pamphlets...

**FACILITATOR:** Mm.

**PARTICIPANT:** ...if perhaps you see that it’s difficult to begin the talk orally, you can start with a bit of books, begin it by reading them, asking...

**FACILITATOR:** Okay. So, if it’s your partner or someone else who approaches you first and says “I want to get circumcised”, how will that make you feel?

**PARTICIPANT:** Good, it will make me happy.

**FACILITATOR:** Okay. Eh. What will make you happy about that?

**PARTICIPANT:** I’ll know that this won’t just benefit only him.

**FACILITATOR:** Yes.

**PARTICIPANT:** It’ll also benefit me.

**FACILITATOR:** Mm.

**PARTICIPANT:** I’ll know that he is also not in a higher risk of HIV infection.

**FACILITATOR:** Yes.

**PARTICIPANT:** And then I’ll also be protected.

**FACILITATOR:** Mm.

**PARTICIPANT:** When reading, we hear that that foreskin is able to cause me cancer as a woman.

**FACILITATOR:** Yes.

**PARTICIPANT:** Yes.

**FACILITATOR:** Oh! Okay. So, in what way would you say circumcision is of help to couples?

**PARTICIPANT:** Ey, it helps.

**FACILITATOR:** Mm.

**PARTICIPANT:** It’s the issue of [inaudible]...it helps health wise.

**FACILITATOR:** Yes.

**PARTICIPANT:** It’ll help me not to get cervical cancer.

**FACILITATOR:** Mmh.

**PARTICIPANT:** It’ll help him not to get...not to have a high risk of contracting HIV and when...when it’s like that...

**FACILITATOR:** Mm.

**PARTICIPANT:** …my risks are also minimised.

**FACILITATOR:** Mm, mm.

**PARTICIPANT:** As well as, as well as enjoying making love.

**FACILITATOR:** Yes, yes.

**PARTICIPANT:** Mm. Because truth be told, when you’ve, eh, experienced someone who is uncircumcised...

**FACILITATOR:** Yes.

**PARTICIPANT:** ...and one who has been circumcised...

**FACILITATOR:** Mm.

**PARTICIPANT:** There’s a difference there. When you’ve been with one who is uncircumcised, that foreskin will cut you.

**FACILITATOR:** Yes, yes.

**PARTICIPANT:** Yes. That’s something I know.

**FACILITATOR:** Mm.

**PARTICIPANT:** It’s not enjoyable.

**FACILITATOR:** Okay. So, it cuts, it cuts you as a woman?

**PARTICIPANT:** A female person.

**FACILITATOR:** Oh! Okay. So, with people who are in love, eh, who do you think should initiate the issue of circumcision between a man and a woman?

**PARTICIPANT:** I think you as a female person should initiate it if your partner is uncircumcised.

**FACILITATOR:** Mm.

**PARTICIPANT:** Because you’re talking about an old person, you’re both adults, why was he...what made him not to go all along? Maybe he doesn’t see it as important.

**FACILITATOR:** Mm.

**PARTICIPANT:** Do you understand?

**FACILITATOR:** Mm.

**PARTICIPANT:** So, I as a female person who knows the importance of circumcision…the advantages and the benefits, I will initiate it, he is my partner.

**FACILITATOR:** Mm. Oh! So, how do you think that will make him feel, if maybe it’s you who initiates it?

**PARTICIPANT:** If it’s me who initiates it?

**FACILITATOR:** Yes, do you think it’ll encourage him or will he be happy about it or what?

**PARTICIPANT:** I think the manner in which you’ll initiate it as his partner, your approach...

**FACILITATOR:** Mm.

**PARTICIPANT:** Mm. It’s the thing which matters...approach. The way in which you’ll approach a person…there’s a dialogue. You take him, sit him down and give him your reasons, and also understand how he feels like. It’s not a matter of you saying “go, go, if you won’t go I’m leaving you”.

**FACILITATOR:** Mm.

**PARTICIPANT:** ...you should understand him, when he’s also giving his reasons you should listen to him, you should reason together.

**FACILITATOR:** Mm.

**PARTICIPANT:** I think in the end he will hear you out.

**FACILITATOR:** Mm. Oh, okay. So, what do you think are the reasons for men not to want to get circumcised?

**PARTICIPANT:** Some say it’s painful.

**FACILITATOR:** Mm.

**PARTICIPANT:** They hear others.

**FACILITATOR:** Mm.

**PARTICIPANT:** Others don’t even hear from anyone, they just say maybe...it’s lack of knowledge really, not knowing.

**FACILITATOR:** Mm.

**PARTICIPANT:** He just tells himself that you just get there, they take a razor blade or scissors and cut you.

**FACILITATOR:** Mm, mm.

**PARTICIPANT:** Some, it’s pain. Some it’s religious beliefs.

**FACILITATOR:** Yes.

**PARTCIPANT:** You see. He’ll say, no, from a church’s perspective we don’t do such a thing.

**FACILITATOR:** Mm.

**PARTICIPANT:** Others are held back by the fact that they’re already old, he is ashamed that at such an old age, you see?...

**FACILITATOR:** Mm.

**PARTICIPANT:** ...“right now I’m here with small children.”

**FACILITATOR:** Mm.

**PARTICIPANT:** Another one…just as we know that men aren’t able to stay away from sex for too many days...

**FACILITATOR:** Mm.

**PARTCIPANT:** Another one thinks that this will...like, it’ll cause a disruption in my sex life.

**FACILITATOR:** Mm.

**PARTICIPANT:** i have to stay six weeks whereas we know that a man thinks about sex every 3 minutes

**FACILITATOR:** yah

**PARTICIPANT:** He feels that six weeks is a very long period...

**FACILITATOR:** Oh!

**PARTICIPANT: ...**“it’s better if I didn’t go”.

**FACILITATOR:** Okay. I also hear you saying it’s not knowing…it’s lack of knowledge. How do you think we can inform men?

**PARTICIPANT:** I think that through these very outreach programmes.

**FACILITATOR:** Yes.

**PARTCIPANT:** Campaigns.

**FACILITATOR:** Mm.

**PARTICIPANT:** These door to doors.

**FACILITATOR:** Mm.

**PARTICIPANT:** Because a lot of people are working, when we go into people’s homes you find that we only find women.

**FACILITATOR:** Yes.

**PARTICIPANT:** If it happens that in firms, companies, there are days in which awareness is made...

**FACILITATOR:** Yes.

**PARTCIPANT:** ...about circumcision.

**FACILITATOR:** Oh! Okay.

**PARTICIPANT:** Pamphlets should be taken there. Perhaps there should be help-lines, so that when a person things about such a thing...

**FACILITATOR:** Mm.

**PARTICIPANT:** ...he may find a counsellor online and talk to it.

**FACILITATOR:** Oh! So, where a person may call or get there and...

**PARTICIPANT:** Mm.

**FACILITATOR:** He...okay. So, when a male person, it might be your younger brother or your father, or your partner, if he says “I want to get circumcised”, how will you think about them? What is it that might cross you mind?

**PARTCIPANT:** I’ll...what I’ll think is that maybe this person already has information.

**FACILITATOR:** Mm.

**PARTICIPANT:** Because I don’t believe that you might want to do something you don’t understand.

**FACILITATOR:** Mm.

**PARTICIPANT:** Concerning what...concerning what.

**FACILITATOR:** Yes.

**PARTICIPANT:** Perhaps that person has already received information saying such a thing helps in such a way. Perhaps that’s the reason he now wants to get circumcised.

**FACILITATOR:** Mm. Oh! You mean he has already acquired some knowledge...

**PARTCIPANT:** Mm.

**FACILITATOR:** ...concerning circumcision.

**PARTICIPANT:** Mm.

**FACILITATOR:** According to you, is male circumcision a good idea or what?

**PARTICIPANT:** Yes, it’s a good idea.

**FACILITATOR:** What makes you think so?

**PARTICIPANT:** It’ll take us back to health issues.

**FACILITATOR:** Mm, mm.

**PARTCIPANT:** When we say…when they say research says it minimises...circumcision minimises HIV infections by 60%...

**FACILITATOR:** Yes.

**PARTICIPANT:** Then you are safer than an uncircumcised person. So, it’s important.

**FACILITATOR:** It’s important... Okay. So it’s time for us to end...

**PARTICIPANT:** Mm.

**FACILITATOR:** ...the conv...eh our conversation, but before we close, I’m going to ask that eh, that you tell me what else we may discuss which we didn’t touch upon, which you think is important.

**PARTICIPANT:** I think we discussed them all.

**FACILITATOR:** We discussed them all? Okay. So, I have a question here, eh, eh with women...eh do they ever discuss about circumcision as women only? Do you think they talk?

**PARTCIPANT:** Yes, a lot.

**FACILITATOR:** [Inaudible ] where does this normally take place? Do they just meet up as women to talk or is it just random conversations, what makes...

**PARTICIPANT:** In...in the workplace right.

**FACILITATOR:** Yes.

**PARTICIPANT:** Perhaps during lunch time.

**FACILITATOR:** Yes.

**PARTICIPANT:** People talk, another is talking about her partner or yet another has just met someone...

**FACILITATOR:** Mm.

**PARTICIPANT:** ...when they go to make love she finds out that this person has never been circumcised before.

**FACILITATOR:** Mm.

**PARTICIPANT:** Then a lot of times for us women, such a thing is an embarrassment, you see...

**FACILITATOR:** Yes, yes.

**PARTICIPANT:** ...when a man is uncircumcised.

**FACILITATOR:** Yes.

**PARTICIPANT:** Then you won’t willingly agree...

**FACILITATOR:** Mm.

**PARTICIPANT:** ...to continue with that person, and we sometimes get rude, we’ll say to him “no, you’re still like this?...

**FACILITATOR:** Mm.

**PARTICIPANT:** ...is there still a person like this in this day and age?”...

**FACILITATOR:** Mm mm.

**PARTICIPANT:** You see. “You are still uncircumcised?”

**FACILITATOR:**  Yes.

**PARTICIPANT:** You see?

**FACILITATOR:** Yes.

**PARTICIPANT:** So, we talk about such things, we laugh, you’ll hear another one telling you that “no ways friend, leave that guy”.

**FACILITATOR:** Mm.

**PARTICIPANT:** “What kind of a person is he?”

**FACILITATOR:** Yes. Okay. So women don’t want an uncircumcised man?

**PARTICIPANT:** Ay, 99.9% hey.

**FACILITATOR:** Mm.

**PARTICIPANT:** An uncircumcised man no ways!

**FACILITATOR:** Okay. So now if a man has not...when she tells him such things, do these encourage men or do they...do they encourage them or what?

**PARTICIPANT:** Ey, they will push them over the edge.

**FACILITATOR:** Mm.

**PARTICIPANT:** You meet me and I tell you that, right?

**FACILITATOR:** Mm.

**PARTICIPANT:** I leave you.

**FACILITATOR:** Mm.

**PARTICIPANT:** Next week you meet another one and she tells you the same thing, something should rill a bell in your mind that something isn’t right.

**FACILITATOR:** Mm. Okay. Alright. I...I...I forgot to ask you earlier on, at home, between a man and a woman, who do you think should talk to the children about the issue of circumcision?

**PARTICIPANT:** It’s the father.

**FACILITATOR:** It’s the father? Why should it be the father?

**PARTICIPANT:** Because we are talking about circumcision for boys...

**FACILITATOR:** Yes.

**PARTICIPANT:** ...and men.

**FACILITATOR:** Yes.

**PARTICIPANT:** Right?

**FACILITATOR:** Yes.

**PARTICIOANT:** A male person is supposed to talk to his male children about circumcision and explain to them...if he also hasn’t been circumcised...

**FACILITATOR:** Yes.

**PARTICIPANT:** ...but he hears the information, he knows it. This should be done by a male person, if he has issues then perhaps he may send a female person, but it’s a subject which should be initiated by a male person.

**FACILITATOR:** By a male person...so, do you think that, with an uncircumcised person, an uncircumcised man, will it be easy for him to discuss the issue of circumcision with his children?

**PARTICIPANT:** I don’t think it can be a problem because we have a lot of NGOs which go about educating people about HIV and AIDS.

**FACILITATOR:** Mm.

**PARTICIPANT:** A lot of people…you know…even if you’re uncircumcised what the benefits of getting circumcised are...

**FACILITATOR:** Yes, yes.

**PARTICIPANT:** ...the advantages, you might have not had the opportunity to undergo circumcision...

**FACILITATOR:** Mm.

**PARTICIPANT:** ...but I don’t believe that you might want what happened to you to happen to your children. It’s time for a change right?

**FACILITATOR:** Mm. Oh! so, you think it won’t be a problem for an uncircumcised man to tell his children...

**PARTICIPANT:** No, it won’t be a problem.

**FACILITATOR:** So, for a mother who tells her children to go and get circumcised, how do you think this will make them feel?

**PARTICIPANT:** The children?

**FACILITATOR:** Yes.

**PARTICIPANT:** Children don’t have issues, it’s what they like, there’s no child who, in this day and age…

**FACILITATOR:** Mmm...

**FACILITATOR:** ...doesn’t want to get circumcised.

**FACILITATOR:** Okay.

**PARTICIPANT:** Mm.

**FACILITATOR:** Mm

**PARTICIPANT:** So, even my nine year old according to him he should have done it by now, I explain to him and say “you have to be ten years old dear”.

**FACILITATOR:** Yes, yes.

**PARTICIPANT:** His dad says he’ll go when he is 16, he fights the idea, he says “next year I want to see myself there, having done it”. What does he know?

**FACILITATOR:** Mm.

**PARTICIPANT:** Do you see that it’s something which is already there in their minds?

**FACILITATOR:** Mm.

**PARTICIPANT:** They know that you should do such a thing.

**FACILITATOR:** Mm. So children know?

**PARTICIPANT:** Yes.
