# Supplementary material for: “To speak or not to speak”: A qualitative analysis on the attitude and willingness of women to start conversations about voluntary medical male circumcision with their partners in a peri-urban area, South Africa
Source: PLoS One. 2019 Jan 25;14(1):e0210480. doi: 10.1371/journal.pone.0210480 (PMC6347244; doi:10.1371/journal.pone.0210480)
Supplement: S1 File — (ZIP) [file pone.0210480.s003.zip › QF019_QC2.docx]

Participant ID (P): QF0019

RA: Can you allow me to audio record the interview?

P: Yes I allow you to audio record the interview.

RA: So can I ask you where do you come from?

P: I am from {} (participant address) and grew up in {} (participant address). I also went to study in the {} (participant address) in a boarding school but I came back.

RA: How long is the distance where you are staying in {} (participant address) to the Clinic?

P: It’s a walking distance about 15 minutes.

RA: Ok did you know that at {} (clinic address) there is a man’s Clinic?

P: Yes I knew.

RA: And did you know what is happening?

P: Yes I knew everything ever since it started.

RA: So in this Clinic you knew what was happening?

P: I know that before this Clinic Come here there is another one that was in the {} (clinic address). Like where I’m working we once visited it to see what they are doing, so they decided to open another {} (name of NGO) Clinic this side, and this Clinic is specializing on medical male circumcision. So when it started I knew exactly what was happening.

RA: I like it because you just talked about medical male circumcision, what is it that you understand about medical male circumcision?

P: Like the medical one?

RA: Just circumcision in general, what is it that you know about it?

P: It’s the removal of the foreskin; can I also speak in English?

RA: Yes.

P: Is to remove the foreskin, the front part of the penis. Some are doing it for the culture; some are doing it for their health so that they can be clean. so that they are able to clean them when they bath.

RA: I heard you talking about culture when you are saying that some are doing it culturally what do you mean?

P: I’m a Xhosa I grew up knowing that circumcision is in our culture. I grew up knowing that when a child is reaching a curtain age he must go and get circumcised. That’s where they are growing from being a boy to be a man.

RA: So what is happening in traditional circumcision how is their procedure do you have any knowledge of it?

P: There time he is still a boy there is a day where he is gonna prepare himself as he is gonna go to the mountains. In that week he is singing with other boys, ringing the bell that it’s time to go in the mountains for circumcision and he is wearing traditional outfit singing songs as he is saying bye bye stage of being a boy and saying hello to the manhood. Then as he was singing the whole week then on a Friday he gathers with other boys and girls of his age to announce that he is going to the mountains in the morning for circumcision and when he is coming back, he will come back as a man. In the morning the following day the elders will come and take him and they perform the culture, on the previous day the women built the house where he is gonna leave on and he is not allowed to see that house until he goes there. Then in the morning around 03:00 the elders take him so that they circumcise him. They must be sleep with him all of them. They take off all the clothes and they begin to wear blanket, they will buy them white blankets or white blanket with a black line and may be with a red line, that what they wear and they stay in the mountains for 7 days and they are not allowed to take water and they not allowed to eat salty food then after 7 days they slaughter a goat. They use to stay in the mountains for 3 months but now most people are studying its only one month now. We cannot know what is happening in the mountains because we are women. As women we don’t know what is happening in the mountain, they are not allowed to tell us what is happening even if he is your partner.

After another 7 days they slaughter another goat then they man come out and go home, they slaughter another goat and a cow and they celebrate with him that he is from the mountain and he is no longer a boy but a man now. When he is from the mountains he is not wearing like others he wears different from them, he wears khaki and he is putting something red in his face and he is not supposed to bath in the house he has to wake up around 4: 00 and go in the rivers to bath, then after 7 day he wears the normal clothes now. Even when he is greeting he has to shake hands and he must use the stick and you don’t have to touch them. He is not supposed to be with his partner not at all.

RA: Why is he has to wear the caught?

P: It means that you are a new person. You are new in the manhood. You are just like a new wife and they also put on something black on their head. Even a new wife she puts something black in her head because she is no longer a girl now but a wife , you are able to see that by the black clothes in the head. So they put the black clothes in the head to show that they are new in the manhood. Even a new wife has to put the black clothes in her head, and that is a symbol that he is a new wife in that family. So even a man who is from the mountains he is also new in the manhood we call him *isokana* in *Xhosa*.

RA: Ok what about greeting with a stick?

P: Even greeting with a stick, he is not supposed to touch people with his hands when greeting them he is respecting all the time. When he is walking in the streets he does not allowed to look back, even if there is someone calling him he is not allowed to look back. Those are the rules they give them in the mountains. On the day of celebration old men who attended they call him and teach him about health. They tell them what are they supposed to do and what are they not supposed to do. Learn from those that are also from the mountain and if they are behaving well. They give them examples of the guys that are circumcised and they are behaving well. And they say do you see so and so that he is behaving well, we want you to do things as he does. At this stage of your life you are expected to take a wife, and have a family and lot of things and things you do when you are growing, things that are expected of you when you grow.

RA: And then if we can compare the traditional one with the Clinic one how big is the difference what is the difference between the two?

P: At the Clinic they just cut the foreskin only, there is no teachings or education provided , and most of those from the Clinic they don’t change their behavior they still do the same habits that they were doing before they got circumcised they don’t change their behavior. They don’t think about changing. Otherwise these two people are the same because they are both circumcised. And another thing about traditional circumcision is that we don’t know how they cut the foreskin and at the Clinic we know that they remove the whole foreskin.

RA: so when you talk about the difference you are saying at the mountains they teach you and there are rules, what about the Clinic are they not teaching you at the Clinic , they just come in and go?, from your opinion how is it important for a man to be educated after they got circumcised?

P: It is very important especially in these days. Like some people they grow up without their fathers so they hardly get an advice from an old person who has an experience, of how is life and especially from a man. And the feeling of having a father, because there is no coaching there is only one father; there are so many elders there in tradition that they will give him guidance about life. And I think he is getting a chance to connect with the older man there. But if you have a father the it’s fine. Some they get more advise because there is also older man there who has a lot of experience. There are older men in the mountains passing knowledge from generation to generation.

RA: So I heard you saying as women you are not told what is happening in the mountains, why?

P: They are not telling us, I think it’s the way their culture because even us as women we don’t tell them how is happening when we are giving bath, we cannot talk about what is happening with man. I think it is a secret same applies with them. There are things that women must keep in private and there are things that man must keep private.

RA: So now in a situation where as a women you want to encourage a man to go and get circumcised, how would you do that?

P: As a woman you become concerned like the time my younger brother was about to go to the mountain to do circumcision my mother was concerned that he must go in the mountains and get circumcised. They don’t think of what is happening in the mountains, because in some arrears boys die in the mountains. So she was worried. But you don’t have to encourage them because in my culture they already know that once a boy reaches a certain age he has to get circumcised. So I’m saying my mother was worried because she does not have the access to see him when he is there, so that she chats with him and prepare food for him, she is not allowed to do that. Even if they cooked at home they are not allowed to give him that food, so she doesn’t have rights , even if she passed away they will not tell her, the only way she will know is when the child is not coming back the time he is supposed to come back home and she will not even pay respect.

RA: As now as women, what the role you think you can play in sending messages to the man so that they consider medical male circumcision?

P: You know I sometimes feel pain because I have two boys, I couldn’t take them through medical male circumcision because they must do it traditionally. I don’t know how can I encourage them, sometimes I talk to my brother and ask him what can I do so that my kids they go for circumcision as they young and then later on they do culture and he told me that it has nothing to do with me, and I don’t even have to talk about it. So I don’t have a right nothing, there is nothing I can do about them. And I cannot even talk to their biological father because he also did circumcision in the mountains as well, and their grandparents as well and it’s like they also have to go the same route and I don’t have a say at all.

RA: Have you tried to come with them here at the Clinic?

P: Yes I wanted to let them here because the older one is 15 years already and the younger one is 9 years. I want them to come here for circumcision. So the only hope I have is that when the time for them to go to the mountains , I will try and convince them to come at the Clinic and do medical male circumcision and then they go in the mountains and immediately so that they are safe.

RA: So what are your reasons for you saying you want them to come at the Clinic, you also talked about to be safe, what is another reason for you to say you want them to come at the Clinic? And why you don’t want them to go in the mountain?

P: I think in the mountains they are using one instrument to cut them that is my fear, and there is also bandages as well you understand, so I think they can also get infections as well. And sometimes they just cut them without knowing their status, if you diabetic or what they don’t know that. If they come at the Clinic they get tested of so many things so it’s safer. Some people they bleed too much and you find out that they are still cutting that person such that the person dies because they don’t know what to do now. So if they can check things like that, so we can minimize the risk of them dying in the mountains.

RA: So you grew up in an environment where men were going in the mountains for male circumcision and how did you feel about that?

P: Its scaring now and when he is still in the mountains you are not happy; you become happy when he is back. I never had of someone who passed on due to traditional circumcision where I’m staying but I heard of them to the places far from where I’m staying. So I feel like even where I’m staying we can experience the same thing it’s also possible that we lose someone due to traditional circumcision. So we are always worried about what is happening in the mountains and every time when those men come back from the mountain healthy and well you become happy because as he is going there you don’t know how the situation is.

RA: Another thing I wanted to know is that at what age are they supposed to go in the mountain for circumcision?

P: From the ages of 15, 16 to 20 and when you go at the age of 20 to 22 you are old already. The go to the mountains from the ages of 16 to 20 years. When they are there in the mountains they teach them some languages, and they teach you according to their culture, they teach you about man and staff like that.

RA: So if you are already at the age of 22 and 23 what happen?

P: You old already, some if they don’t have the money to do all the ceremonies they just go in the mountains to remove the foreskin and don’t do the ceremonies. And they go look for a job and then after 2 to 3 years he come back and do the ceremony. But he has to do the culture. You know when you grow up with your friends you will not feel good when they are going for circumcision and they leave you behind. So that’s why they encourage that they must do according to the age, of the same age must circumcise so that the is no one left behind alone.

RA: So if you are 22 to 23 do they encourage you or not?

P: The force you at that age. They are not encouraging you at this age they tell you what to do. Sometimes they will just come, maybe it’s in the morning and you are still sleeping then they take you by force to the mountains for male circumcision.

RA: Is there any age where they say once you reach that age you will not go for circumcision?

P: No there is no specific age even if you are an elder you can still do it but they encourage people to do is while they still young.

RA: From your culture a man who did not go through that process, how do you view him?

P: He is not a man he is still a boy, we differentiate them, the ones from the mountains we call them man. So those that are not circumcised they are still young and we call him a boy as he is still young. He is not even expected to have girlfriend, unless he can come in Gauteng, but if he is still at home he is not expected to have a girlfriend. If he is not circumcised there is no one who is gonna take him serious, and there is no one who will respect him. There is no one who will listen to him when he is talking, they will tell him that you are still a boy, when he goes in the process of traditional circumcision then they will respect him.

RA: From your opinion would you say circumcision is a good or bad idea?

P: According to my culture it is a good idea, and it can make a man matured. You become matured so I think it’s a god thing to do. A man who did it becomes clean and it becomes easy for them to protect themselves from sexual transmitted diseases so to me it is a good idea in both ways.

RA: So can you tell me what are the benefits of a couple if a man is circumcised?

P: Firstly if a man is circumcised weather traditionally or medically reduces chances of getting HIV infection by 50%. They say there is a possibility that the cell in the foreskin can result to a cervical cancer in a women, however I’m not sure about that so women can end up getting sick because of the foreskin. There is chance that the foreskin causes the cervical cancer in women, another thing is that it makes a man clean. They say the foreskin is dirty and it keeps the dirtiness. So health wise you become healthy.

RA: So just to go back, you said according to your culture a man not circumcised is a boy, is this opinion from other man or women or everyone says that?

P: Everyone, men and women. You will never get a respect and there is no one who will take you serious, even those younger than you who went to the mountains they will not respect you because you are not a man enough. For example if you are 30 years and there another man who is 18 years and he got circumcised and you are not, that man will not respect you they consider you as a boy. To them experiencing the pain you feel when you get circumcised then it means you are matured and that you managed it you become a man.

RA: So now tell me from your pinion if your partner is deciding to get circumcised how would you feel about that?

P: I will feel happy because in terms of our culture he will be respected and I will also respect him as well. And in terms of medical male circumcision we will be safe in terms of HIV and AIDS, cervical cancer and STI’s.

RA: How important it is for your partner to be respected in your cultural idea and you being safe?

P: It is very important to me and it is very important to me. I’m a woman and I respect my culture and I love my culture and I’m proud to be Xhosa. After I broke up with my partner I dated a guy who was not circumcised and I encouraged him to go and do medical male circumcision because in my culture I’m not allowed to date someone who is not circumcised (Laughs) and in terms of our time we are leaving in the days of HIV, STI’’s and I’m encouraged him to go and do medical male circumcision. And we ended up going to the Clinic to book, even there time he was at the Clinic for the operation I was with him, even when he was from the theater I was there, like all the steps I was there .

RA: So how did you receive it, how did you approach it and how did you go about it?

P: I am a very open somebody so I was talking openly, I use to tell him that you are so old and you are not circumcised? And I asked him his culture, and he told me that he grew up in the township so in the township they don’t care much about whether you are circumcised or not , if you want it you do it and if you don’t want it it’s still fine. I told him that I’m very serious about my health. I believe even if you can go home, you will never be respected because you are not circumcised may be you are sharing a room with my brother. My brother will ask me why I am dating someone who is not circumcised , you this thing it will also make me not to feel good , it will make me feel not respected, so it is very important that your partner is circumcised because the time we are leaving in. It is very important in both ways.

RA: So the time you were telling him how did he feel about it?

P: He was laughing at me but at the same time he understood me because I use to tell him now and again, maybe it took him a year to decide to do it, and I was telling him that you need to do this. He was very afraid of doing it and I could see that he was afraid and I understood. And there is a belief that when you are old you don’t heal properly. But I know that you will heal and as long as they take the HIV tests everything like sugar diabetes. I was telling him about things like that and he was also doing his research as well. And he ended up deciding to do it. He is also working in the department of health as well so this means that he cannot tell people to get circumcised while he is not. He has to do it before so that he will be able to encourage others. So that was another way I approached him with it , I told him that he cannot tell people to do circumcision while you are not circumcised it must start with you.

RA: So far you can say you we patient?

P: Yes to push him, and he ended up coming eventually.

RA: So you did not give up on him.

P: No I did not give up on him and even when he had to come for test he came on his own. And came with the results and he told me that he booked already. And he went to the private not in the public for circumcision, and he told me even the date , so I was happy to see him doing it and I knew that he wanted to do it.

RA: So we would like to know as you experienced this, like you suggested to your partner to go and do circumcision as you were patient as far…, as a women now who would like to encourage her partner to do circumcision what approach that they can use if talking with her partner about it did not work, what else she can say?

P: Like I said that in my culture they do circumcision this way, I think there is a pamphlet that you get at the Clinic, like in any department of health there is lot of pamphlets talking about medical male circumcision. Maybe she can give him those pamphlets, and explain a little bit of what is written there, and help him read about it. And she must tell him her feelings about it and she must also tell him about her culture as well before he forces him. And she must also understand how her partner feels about this whole thing because there are Zulu people and those people are very stubborn and they were not doing it. It’s only now that they are also doing circumcision. So somebody like that wants you to you to have patience and not to give up on him. If he cannot read, then read for him, and explain to him things he does not understand. And show the risk that he is exposed do as he has the foreskin. So man who takes time to understand don’t give up on the because you can tell them one thing even five tomes and don’t understand even if you stay with him in the house, you can tell him to do something on the first time and not do it, second time and not do it and third time, and he does it, so you need to be patient with them. You also need to tell them that man who got circumcised have 60% chances of not getting HIV but it doesn’t mean that you don’t have to use a condom, and she must tell him how is MMC gonna benefit him. Even if he can cheat, you know guy they can cheat and maybe not use a condom, I have a chance of not getting an HIV, and even if he is cheating you have a chance of not getting the sexual diseases. You show him things that will motivate him and make sure that he knows how MMC will benefit him.

RA: Now what approach a woman should avoid when trying to encourage a man to do circumcision?

P: I think they must not criticize them, like in my case I was very surprise that he is not yet circumcised, like things like that. You mustn’t show that you are surprised that he is not circumcised, avoid to describe him as someone dirty, someone who doesn’t have a respect, because maybe some man are not circumcised because it is not in their culture , so don’t judge them at all. Even if you see that he is not circumcised approach him nicely and talks with him nicely and help hip understand your beliefs and also be interested in his beliefs as well and then you meet each other half way. Don’t judge him because you don’t know why is he not circumcised.

RA: Now we are approaching the end of this part, but before we end this part is there anything you feel like it’s important and we did not talk about it and you would like us to talk about it before we go to the next step?

P: I think we talked about everything unless you feel like you need more to ask me about. So I think mine is to answer whatever you are asking me. And I think we talked a lot about cult here.
