# Supplementary material for: “To speak or not to speak”: A qualitative analysis on the attitude and willingness of women to start conversations about voluntary medical male circumcision with their partners in a peri-urban area, South Africa
Source: PLoS One. 2019 Jan 25;14(1):e0210480. doi: 10.1371/journal.pone.0210480 (PMC6347244; doi:10.1371/journal.pone.0210480)
Supplement: S1 File — (ZIP) [file pone.0210480.s003.zip › QF020_QC2.docx]

PARTICIPANT ID (P) QF020

RA: will you allow me to audio record the interview?

P: yes, I allow you to audio record the interview.

RA: ok, can you please try to… your voice, try to, your voice must be up so that I can hear you very well when you are talking *neh*.

P: ok

RA: mmm, what do you understand about the term medical male circumcision?

P: is the removal the, removal of the foreskin.

RA: ok

P: yah

RA: removal of the foreskin?

P: yes

RA: ok. How many types of mal-, male circumcision do you know?

P: two.

RA: two? Can we talk about those two types, can we first start with the other one and explain what’s going on there and you tell me the other one and you explain what’s going on there.

P: oh ok. I just only know there is a traditional one and the medical one.

RA: ok.

P: but I just know that they just remove the foreskin but I don’t know how.

RA: ok.

P: yah

RA: so which one do you know better, is it the medical one or the traditional one?

P: according to me is like the same thing.

RA: is the same thing?

P: yah, because they remove the skin..

RA: ok, why are you saying is the same thing?

P: ok, like when there is like, when they done removing the skin it looks the same.

RA: it looks the same?

P: yah

RA: what looks the same?

P: the penis

RA: the penis?

P: yah

RA: oh ok.

P: so I don’t know what is the different

RA: ok, why are you saying the penis looks the same with you, or maybe you have had an experience of that?

P: no I don’t have the experience, is just that because of that skin is removed there and then I see is like the same. I don’t know, is there any different or something there.

RA: oh ok, so in terms of removing the skin, what do you understand about the procedure? Is the procedure the same or is not the same? The way of removing the foreskin, how are they removing the foreskin in tradition and how are they removing the foreskin in, in the medical as you said you know two types?

P: ok

RA: What do you think is happening there?

P: eee, in tradition I really don’t know but is like is not safe in tradition but in the medical one as I heard they to screen you and do the tests

RA: yah. Ok.

P: that’s what I know.

RA: ok. So you are saying in the tradition is not safe?

P: yes

RA: Why are you saying that?

P: because they do screening and do the test.

RA: oh ok, how are they doing it?

P: eee, is like they using in a traditional way I don’t know how to explain it but they don’t do it like in the medical way.

RA: ok

P: yah

RA: oh right. So have thought about telling your partner or a family member about medical male circumcision? Have you thought about telling someone maybe you’re your partner or family member about eee, medical male circumcision?

P: eee, I think they know

RA: mmm?

P: they know they all coming from there

RA: they all coming?

P: yah

RA: ok, but have you thought about it, about telling someone?

P: no, I will never told them because, I know they know everything they coming from doing it.

RA: mmm, why are saying they are coming?

P: they did it

RA: is that your friend or your partner?

P: my partner and all my family.

RA: and all your family?

P: yah

RA: how did they come? Like your family members, how did they come?

P: how did they come?

RA: mmm

P: I don’t understand.

RA: how did they went for medical, did you say anything to them about medical male circumcision? Did you highlight or suggested it to them?

P: no

RA: did you talk about it to them?

P: no they just went there it was long time

RA: it was long time?

P: yah

RA: oh ok, what about your partner?

P: I can’t, can’t, like we met, when we met he was already circumcised

RA: oh, he was already circumcised?

P: yes

RA: oh ok, alright. And then what would it mean, if a man in a relationship has suggested that he wants to go for circumcision? What would it mean to you? Will it be any different if a woman brought up the topic? General from your personal point of view in a relationship, if a man is suggesting that, *eish* am thinking of going to do the medical male circumcision, what would it mean to you that suggestion or that idea?

P: it would be a good idea

RA: it will be a good idea?

P: mmm

RA: why are you saying that?

P: (cough) because eee, circumcision is reducing mmm, other disease or whatever {sentence not clear}

RA: so, would it be any difference if it’s a female who is bringing up the topic, that a man must go in relationship, that a man must go for circumcision, will it bring up any difference if it’s female who is saying that he must go for circumcision?

P: ok no, like eee, you cant force somebody if he doesn’t to do it but even it’s a female but it will depend on the man *huri naa* how does he take it, does he want to go or what.

RA: mmm

P: yah

RA: so someone who is forcing how is that person, saying it?

P: like telling the man that he must go there.

RA: mmm

P: mmm, like is like you forcing, not like eee, joking.

RA: so how to tell me a man to go for circumcision not in a forcing manner? Maybe how can you tell a man?

P: ok, according to me I can say mm, I don’t know what to say (laugh). Mm, ok, I can say… I can tell the man in like eee, you know is better to go and circumcise because you reducing many diseases they are a lot of diseases out there, so it’s better to go and circumcise but is not a force is up to you and then is your decision, yah.

RA: ok. Why is it his decision?

P: I can’t decide for him

RA: why a man has to decide on doing medical male circumcision?

P: maybe some people they don’t wanna be told by a woman (laugh).

RA: why? What is the problem?

P: I really don’t know what is the problem but they do, they don’t

RA: mmm

P: some of them they are afraid

RA: who is afraid?

P: like the man they are afraid to come and do circumcision, is like when you said eee, you will be testing for HIV and some of them will tell that *yoh* am afraid, I can’t go there, then I will tell a person that then I will take your time and think about it.

RA: mm

P: yah, like that.

RA: so, why are you going to suggest that the person must take his time and think about it? Why, why are you going to tell him that take your time and think about it, why? Why, why do you feel like telling him that he must take his time and think about it, why?

P: because is not easy for some people to just do something just like that.

RA: mm

P: some they, they have to maybe they want to go and do it in traditional way some they want to come to a medical one, so it’s a different way they have to think maybe talk to elders, it depends on them.

RA: mm

P: that’s why.

RA: so you are saying that maybe they must talk to elders why, do they have to talk to elders?

P: *yoh…*

RA: why do you feel like some they need to talk to elders, why?

P: yah because sometimes the elders they will eee, give them a good advice for them to decide *gori naa,* they, which way want to do that I either a tradition or a medical. Is not like they gonna tell him that, you must go there to the traditional or you must go to the medical, they gonna give them an advice in an understanding way, yah.

RA: mmm, what is that understanding way?

P: *yoh*, I don’t know how to explain it, I don’t know how am gonna explain it now.

RA: you, you are saying the elders will give them the good advice?

P: yah

RA: what kind of good advice to give? Like advice, like how? What advice? I want to know the good advice maybe what is the good advice.

P: ok, like if, for example maybe as am a parent I have a son, maybe my son she, his afraid to go to circumcision or maybe his confused he doesn’t know which one, he must go to the tradition, or to the medical, maybe his got friends which coming from the tradition he want to go there, other friends they went in medical he wants to go there, so his confused. I can te-,sit him down and say you know my son, I think is up to you to choose one thing, don’t be confused and don’t let others like somebody to play with your head or anything, yah. That’s how I can say is a good way to tell your son, he will sit down and think about it, or which way he want to do ee, circumcision

RA: mmm

P: yah

RA: why men do have to choose, the way that, why do they why do they have to choose? Like you are saying as a parent you will tell him that there is traditional and there is medical and then he will have to choose. Why they have to choose?

P: just because am a women I cannot choose for him

RA: mmm?

P: as am a women I cant choose for him, is like am forcing him maybe he doesn’t want to go to ee, traditional and then I said he you must go to the traditional, yah, you see. He want to go to medical or maybe he doesn’t want to go to medical and said no you must go to medical, is like am forcing him to do what he doesn’t want, yah.

RA: yah. Oh right. How can we know that the man wants to do medical or traditional how can know it? That he wants a medical or a traditional one.

P: eee, if, his gonna tell you.

RA: mmm, that maybe…

P: he wants to go to the traditional or what, cause even when you talk maybe you telling him, there is this other clinic which is doing medical circumcision, yah, maybe he will tell you know I want to go to the traditional, you see, yah that’s how I will know ok this man wants to go to the traditional.

RA: so if wants to go the traditional what are the going to do?

P: I cant do anything, I cant stop him, is his choice,.

RA: that’s his choice?

P: mmm

RA: you will let him go to the traditional?

P: yes

RA: what do you have to leave, to leave him because of his choice? You said you will have to leave him to go to for traditional one, why do you have to just leave him to go for the traditional?

P: is a difficult question

RA is difficult?

P: is so difficult

RA: so ok. Am… but from your personal point of view, which, which circumcision would you suggest? Would you suggest for a man, maybe your partner, a friend or your family member or any man in the community, would you suggest traditional circumcision or medical circumcision?

P: medical

RA: medical circumcision?

P: ee

RA: why?

P: from where I come from, my grandfather never allowed a kid to go to traditional because tradition is dangerous, people are dying there I mean they, they, most of kids they die’

RA: they die?

P: yah

RA: ok

P: yah

RA: so you would suggest medical because? Because?

P: is safe

RA: why are you saying medical is safe?

P: I have never heard eee, somebody said I circumcised in a medical clinic and then I feel sick or maybe I heard from or saw on the TV that somebody died, to circumcise in a clinic {statement unclear}.

RA: mm

P: or even in a hospital I never heard it

RA: ok. Why are those things not happening in the hospital? Like people saying they are sick or they pass, why are they not happening from those people who did the circumcision in the clinic?

P: *yoh* I don’t know what to say

RA: why are they happening in the traditional one, why are people dying in the traditional one as you said?

P: *yoh* I have no idea why are they dying

RA: mm

P: ok. What could be say way that a person like you could bring up the topic of male circumcision to a partner or a family member? How can you bring up the topic to your family member or to your partner?

P: like if I want to tell them about that?

RA: mmm, about the male circumcision

P: I can, can just show them th-e the, reason, I won’t even be afraid to talk about it because that thing it’s normal. Its there, no one, any one knows about it, so I just have to tell them that they there is a testing for circumcision as you know that a man has to circumcise, it would… I don’t know how to explain it

RA: you are saying as they know, is it known? Circumcision is known?

P: yah

RA: why, why are saying is known?

P: eee, because even a small boy they know that when they grow up they have to go and circumcise (laughing)

RA: even small boys?

P: yah they do know

RA: how are they knowing that?

P: because they heard from… from other people, they heard or maybe their parents tell them that when you grow you have to go and circumcise.

RA: mmm

P: yah

RA: ok, so also said that they heard it from other people like parents, who else? Who else is telling them?

P: like…

RA: who are those people? Like you said they heard from other people who are those people they hearing them from?

P: from their family it depends, even their uncles you see, yah

RA: maybe the uncle?

P: yes

RA: why the uncles?

P: they, they do the tell them, like you know as a, maybe aaa, boy, maybe uncle like to play like, for you to be a man you have to circumcise or yah. Mostly I heard those people who like to take kids to traditional one they, they do like that, they talking as if they making a joke but is not a joke is a serious thing.

RA: mmm

P: yah

RA: so ok. Why are they putting it in a joke manner?

P: I don’t know, I never asked…

RA: but what do you think? Why the uncle would put it in a joke manner to that boy?

P: *tjo* I have no idea

RA: you have no idea?

P: yes

RA: oh ok, mmm, ok then, what approach should a person like you avoid when suggesting your partner, to your partner or a family member to go for male circumcision? What is it you should avoid when you are suggesting that? When you are talking to them, what is it you mustn’t say, what is it you must avoid?

P: I don’t know what to say…

RA: you dint know what to say?

P: yah

RA: how to tell them that they must go for medical male circumcision, a man at the same year, how to, to, to put this to them? How can we start when we are telling them?

P: eee, I don’t know if I can put it the way you have, like maybe if am walking like when am walking like I will only say, maybe I will tell them that *ei*, we need people to go and check for any disease and so that they must know their status while it still early so they can get treatment early and then we need, again we need this young boys, I mean a man or boys from 10 years upwards to go and circumcise so that they can, it can reduce those, the sickness that’s what I will say.

RA: the sickness of what?

P: the disease, I can say like that.

RA: ok.

P: yah

RA: like diseases like what, they are so many, which diseases are you mentioning?

P: like HIV, and ST, STI.

RA: mmm. Tell me about your experience if you have tried, or when you were trying to tell your partner or family member, just a man in the community about going for medical male circumcision. What was your experience? When you were tell them, what happened? And especially like you told me that this is what you are doing, you also suggest, you also tell people, you also let them aware of the circumcision done here at {} (name of clinic) and what was your experiences when you were telling those man about the circumcision that is done here.

P: yoh

RA: what is it that they used to say to you when you are telling them about it?

P: some they, they will tell me that they, they go, they do it in traditional way, some they said they already did it, and some they ask me for the contact number, yah or those pamphlet which we have

RA: mmm

P: yah, some say no we will go yah, and that’s it.

RA: were you asking then why they are not going, when are they going to go those who said they will go?

P: no I don’t ask any questions if they say they will go. I don’t ask any questions if they say they will go.

RA: so in terms of follow up if they came or not, how, how are you checking that? Are you checking that those who said they will go, are they coming really, or they are not , are you checking that? Is there any way of checking if like they told you they will come up, are they coming?

P: yah cause I last, last, last year November most of them they, they stay around my way I stay so they did come.

RA: mmm

P: yah. All of them which I was talking to them they did come.

RA: oh ok.

P: yah

RA: oh right. They told you?

P: yah they told me and then like I even see it, like you can see how when somebody is after circumcision, you can see how they walk, maybe when he went to, he was going to buy a cigarette by the shop

RA: mm

P: maybe when you see him, he said, yoh I went there

RA: mmm

P: yah just like that you can

RA: mmm, so you are saying you can even see?

P: yah

RA: what else do you see that the person went for circumcision?

P: I only see the walk (laugh)

RA: the walk?

P: yah the walk and the way they walk

RA: mmm

P: that…

RA: how is their walk?

P: they don’t walk like normally (laugh), the way the person walks.

RA: ok.

P: They walk, like it wasn’t, the time they told me it wasn’t like, it was like maybe they came here today and then tomorrow they saw me

RA: mmm

P: yah

RA: mm

P: you see, they still like, I don’t know if they felt the pain or something, yah, the way they were walking, it was like they still felt the pain.

RA: oh ok.

P: yah

RA: oh right. Ok, so you are saying all of them that you talked to them that they must come for circumcision they all of them came? You saw them they came?

P: yes

RA: why, why, why, what is their main reason of them coming? What do you think, is the reason for them to come?

P: I didn’t ask them

RA: do you think maybe is the way you put it to them or not?

P: no I think because even before eee, this clinic, before they make it in the clinic, you were supposed to go to a doctor and pay maybe R500 or R700, so now it’s free, I think when you tell them no there at {} (name of clinic) is free, yah they just go.

RA: they just go, they just go…

P: whoever who is interested will come because is free, they don’t really need money to check, is just only a transport money.

RA: mmm

P: yah

RA: so if it was not, you’re saying they coming because is free?

P: yah, cause most of those guys which I saw, I talked with they are not working even now, they still, I saw them {unclear statement}

RA: ok

P: yah

RA: so, when you were informing them about it, you told them that is free?

P: yah

RA: ok, so do you think that they came because of that?

P: yah and they, they wanted to maybe long time and then they couldn’t because they still has to pay in money and then they don’t work, yah.

RA: ok, so to have money and not to have money is it delay mmm, (how can I put it), does the money have an effect for man to come or not coming for circumcision?

P: you know… circumcision it does have an effect on them not come because if you don’t have money and then like maybe for example before they used to be doctors who is doing that and they used to be traditional one, mostly they used to go to traditional and they paid just a small amount of money, yah, it wasn’t like going to the doctor cause at the doctor you were be paying I think R500 or so.

RA: oh ok, so how is the situation now, now that they are places like this one as it is free, are the people coming for medical one and not for traditional one?

P: yah, I see, I can say there is lot of traditional one cause I can see mmm, mostly, most people they come for medical cause is free even yah, most they come here.

RA: ok, oh right. Why are you saying most people are coming for traditi-, for this one?

P: cause now even if am watching the news I don’t see many people they used to show before, that in traditional eee, they are people who died or who is sick, it shows that maybe around everywhere there is eee, the medical circumcision even maybe there to the rural areas, yah. So they don’t go there for their own safety.

RA: oh ok.

P: yah

RA: oh right. Do you think that medical male circumcision or do you think that males circumcision is a good idea?

P: yes it is

RA: why? Why are you saying that?

P: mmm, ok.

RA: how or why?

P: I don’t know how to say it like (laughing), I just say it is every time is a good idea to circumcise {couldn’t hear she was speaking very low}… cause when the penis has the foreskin, they are people, for the other man sometimes who doesn’t even bath and then that thing it doesn’t reduce the sickness or the diseases, let me say it’s a disease, yah.

RA: mmm

P: yah

RA: why are you saying…

P: it makes it worse.

RA: it makes it worse?

P: mmm

RA: why do you feel like that? Why are saying it makes it worse? Or how?

P: (laughing), ok.

RA: what do you think are the benefits of male circumcision for a couple? (Coughing). For a couple? What are the benefits?

P: *yoh*, I don’t wanna lie,

RA: in a relationship who do you think should be responsible for raising the topic of male circumcision in a relationship? Is it a male or female who has to come up with this topic?

P: eee… I can say, *yoh, eish*, is so difficult

RA: why are you saying is difficult? In a relationship who has to talk about this topic *ya* male circumcision? Is it a man or a female?

P: so if I say both?

RA: still even that one is fine. You are saying both?

P: yah

RA: why are you saying that both?

P: to support each other I think

RA: to support each other?

P: yah…

RA: how?

P: like eee, if we are in a relationship we talking about that, is like I as a female am supporting him to go and do the circumcision. I have to be supportive.

RA: you have to be supportive?

P: mmm

RA: why are saying, you have to be supportive? Or how can you be supportive?

P: aaa,

RA: you were saying you have to be supportive to him right?

P: yes

RA: how you be, how to be supportive to a man who wants to do circumcision?

P: by, ok, who wants to be it?

RA: yah

P: ok maybe I didn’t understand the question, I cant answer that is so difficult.

RA: how to be supportive to someone who wants to do circumcision, how can we support man? Who wants to do circumcision.

P: mmm, that is hard.

RA: because you said we must be supportive, I want to understand how can you be supportive. A female who is supporting a man who wants to do circumcision, how is she doing that? Mmm? What do you think?

P: yoh, am trying but I don’t know how to, I don’t…

RA: can you put it in your language maybe?

P: even if is in my language…

RA: you don’t know how?

P: mmm

RA: oh ok. How would a man’s decision to be circumcise affect your opinion of him as a person, would it be, would your opinion be more favorable or unfavorable or neutral? If a man has to do circumcision, would you support, your opinion about him to do circumcision, would your opinion be neutral, or favourable or unfavourable? Would you favour a person who wants to do circumcision or not or you would just be neutral to him?

P: I will favour

RA: you would favour?

P: mmm

RA: why are saying you would favour?

P: because…

RA: mmm

P: because, I don’t know what to say

RA: can you try and put it…

P: aaa,

RA: I will understand, try and put, I will understand, what do you think?

P: *yoh*, some of the question are too hard.

RA: mmm, why are you saying you going to favour him? Mmm?

P: because his saving other people like, like by reducing the disease if I may say it like that, yah.

RA: ok, that’s fine, that’s important to me, I understand you very well *neh.* Ok now we are done now for this activity like I explained to you that we’ve got 3 activities, this one and the card and the picture messaging. And then we are done for this one but before we close this one is there something important that you feel like telling me now before we close this one? Is there anything you feel is important and you want to share with me? Maybe from your ‘personal experience, from your understanding, from, that is in relation to the, this topic before we go to the next activity?

P: no

RA: you are fine

P: yah

RA: ok.
