# Supplementary material for: “To speak or not to speak”: A qualitative analysis on the attitude and willingness of women to start conversations about voluntary medical male circumcision with their partners in a peri-urban area, South Africa
Source: PLoS One. 2019 Jan 25;14(1):e0210480. doi: 10.1371/journal.pone.0210480 (PMC6347244; doi:10.1371/journal.pone.0210480)
Supplement: S1 File — (ZIP) [file pone.0210480.s003.zip › QF021_QC2.docx]

PARTICIPANT ID (P) QF021

RA: ok, thank you my sister neh for agreeing to take part in or project I’m going to as you err… to allow me to audio record this interview

P: ok I allow you to audio record the interview

RA: ok, so err… you my sister can you briefly tell me about yourself where do you come from err… where do you stay

P: ok, I stay at {} (participant address)

RA: yes

P: mhm

RA: oh you stay here at {} (participant address) who do you stay with

P: I stay with my family but I don’t have parents I stay with my brother and younger brother it’s the three of us at home

RA: oh and then do you have kids

P: I have one boy child

RA: a boy how old is he

P: he is five years

RA: so you say you live at {} (participant address) do you know this place that you came to

P: to know it I just know it as from the time they launched it

RA: mhm

P: yes but I never entered to see what is happening and then all the information

RA: but you knew what was happening here

P: what is happening I know?

RA: can you explain to me maybe what is happening

P: ok what I know is that they help men who are not circumcised who are not initiated

RA: mhm

P: ya they help them by circumcising them

RA: so to you when they talk about err… circumcision what do you think what is it that you understand about circumcision

P: ok what I understand about circumcision

RA: yes

P: it’s this thing there are diseases as we live

RA: mhm

P: diseases that come with ethnic groups or whatever

RA: yes

P: so they help men that are not circumcised to say that it is them that catch diseases quickly

RA: ok

P: that is what I understand that’s why they made this thing of circumcision so that men don’t catch diseases quickly

RA: oh

P: then they spread them

RA: mhm so err… when they say a person is going for circumcision what is it that they do exactly

P: what does he do?

RA: yes the thing that is done

P: ok what I understand is that what is done on the man’s private part

RA: yes

P: there is this thing that is called foreskin

RA: yes

P: the other one is big so they cut it that’s what I know that’s what I know

RA: ok

P: yes they cut it

RA: mhm

P: ya they, they stich after stitching something like that then they give you pills then you take them then you get a little bit sharp for those times

RA: oh so the foreskin from a man’s private part they…

P: ya the foreskin from a man’s private part

RA: they cut it

P: ya

RA: oh ok and then err… what are different types of circumcision that you know

P: types

RA: yes

P: I know circumcision and this one that they go to the mountain for initiation but I don’t know what happens in the mountain

RA: ok so can you I heard you telling me that err… circum-, when they say circumcision a man goes to cut so on all the circumcision places they do that

P: ya they do that

RA: and then they stitch

P: they stitch but on the mountain I don’t know if they do stitch

RA: oh ok

P: ya it’s where I don’t know what I know is that at the male clinic they stitch

RA: oh you are saying at the clinics

P: yes

RA: you know that they stitch but you’ve never heard that at err…

P: the mountains if they stitch or not

RA: yes

P: I just know that they cut but what they use after I dint know

RA: oh ok now err… (clearing throat) at… at the clinic how do people end up going there what is it that bring people to this circumcision do you know how they get to choose this or that maybe they…

P: which side

RA: yes

P: ok what I see is sharp neh

RA: mhm

P: cause clinic is easy and it’s fast

RA: ok

P: neh

RA: mhm

P: and then it’s simple so this one of the mountains you are going to take the whole three weeks at the field you understand

RA: oh ok

P: ya so I see that the one from the clinic is sharp you go today and you come back and you sleep at you place being sharp

RA: oh so you are saying the circumcision in the clinic is sharp

P: ya and safe

RA: how is it safe what is it that you see that is safe

P: what I see is safe

RA: mhm

P: when we like check neh when I see like when you see like the rates of men who went for circumcision at the mountains almost every year in June or July

RA: mhm

P: when they come back there is this thing that so many kids died hee so many kid were injured they are not yet right these thing end up making them to go the clinics

RA: mhm

P: so at the clinic there has never been even a single person that I heard they were not cut sharp so now they are hurt so now they have to go back there

RA: mhm

P: you understand

RA: mhm

P: so I think it reduces death and many diseases

RA: and diseases

P: ya

RA: ok and then err… what do you think causes the deaths at the mountain

P: what is it

RA: what do you think causes that?

P: I don’t know maybe it’s what they are using on the man’s private part I think that there is something that they are using on the man’s private part that is what causes trouble for these boys trouble for these men

RA: ok so it is what you…

P: I don’t know but I think maybe it’s what they are using on the men or maybe I don’t know but I think so

RA: oh so the people that go for circumcision ion the mountain are which age or how do they go

P: ok the circumcision at the mountain doesn’t go with age as long as you feel that ok I’m a man enough

RA: mhm

P: you can go to the mountain they don’t choose

RA: ok what about circumcision at the clinic

P: ok for circumcision at the clinic I heard that its ten years

RA: mhm

P: ten years upwards

RA: ok ten years ok so have you thought maybe of telling err… your partner or a family member about err… circumcision

P: ya I did so I have a boyfriend

RA: mhm

P: so my boyfriend so my boyfriend hasn’t circumcised yet

RA: yes, yes

P: you understand

RA: yes

P: ok I knew well by the time we were going to have sex that ok my boyfriend hasn’t circumcised

RA: mhm

P: and then this thing is giving us problems because it grips

RA: mhm

P: you understand I tried showing him

RA: mhm

P: that ok you have to go and do circum you have to go get help

RA: mhm

P: and he said I’ll go so I said if you ever you can’t I’m here ill help you I’ll take you and well go to the clinic

RA: mhm

P: as its close

RA: ok so he said he will go then what did he end up doing

P: he said he will go so now just because he is always at work what I have to do is to go there and get information on the operating hours

RA: mhm

P: cause he is off he is only available on weekends

RA: so do you think the time you told him that he has to go and get circumcised err… did that encourage him or what

P: ha it didn’t encourage him cause when I tell him he tells me that like this thing of circumcision is not there in their culture

RA: which culture is that?

P: it’s the Zulu culture

RA: ok, ok

P: he said it’s not there in their culture there is no Zulu man that went for circum and I told him its fine but this is the modern days

RA: mhm

P: you understand there are many diseases it’s not the same as the olden days

RA: mhm

P: now it’s the new generation everything has to go on the same stage

RA: mhm

P: you understand for the sake of me not to get diseases from you

RA: ok

P: you see

RA: so now err… you say he said he will go what is it that made him say he will go

P: to say he will go

RA: mhm

P: there is this thing that peo-, is it that we as people talk

RA: mhm

P: you find that maybe we are sitting maybe they are talking we just talk hee an uncircumcised man this hee an uncircumcised man that so when we are saying that those things hit him

RA: mhm

P: as he knows he is uncircumcised

RA: mhm

P: you see if I can say a man that is uncircumcised is not sharp hee what , what, what hai that thing does not sit well with him it sits him off side so that is what made him to say we will go to the clinic to do that thing

RA: so now what are the reasons that make him not to come

P: the reasons he does not come now is that he is working

RA: ok

P: and he knocks off late

RA: ok

P: yes

RA: so which way is best that he can that can be done so that he comes

P: the way that I see is that err… he is going on leave soon

RA: ok

P: ya then when he is on leave

RA: mhm

P: we are going to choose one day on hi leave to come and do to come and enquire first about what is happening and what not then if we have to book we book

RA: ok his reason is time

P: its time he doesn’t have time

RA: ok so I hear you saying that err… the foreskin grips how does it grip

P: you know the foreskin grips in this way

RA: yes

P: I am using condoms

RA: yes

P: cause hai prevention is not good for me so I use condoms so it grips on the condom it grips it at the front there is a possibility that the condom blasts

RA: mhm

P: you understand it grips so if ever he just put it on it shows that it hasn’t gripped but the mean time we get busy that skin starts to come back and it grips the condom’s opening

RA: mhm

P: it covers it

RA: oh

P: ya you see when that thing has gripped this thing is plastic

RA: mhm

P: so it means anytime when he uses power so anytime it can blast

RA: oh ya

P: ya

RA: ok so how did you start him

P: how I approached him

RA: how did you start talking to him about this thing of circumcision?

P: ok we were sitting like he had a girlfriend before and they had a child with that girlfriend

RA: mhm

P: so that girlfriend knows he didn’t go for circum

RA: ok

P: so we were at a party so when we were there it was fun unfortunately that lady was there

RA: oh ok

P: when that lady was there she found us sitting and enjoying she did this she attacked him and shouted at him hee you don’t support you child things like that

RA: mhm

P: you see hai and she swore at him about that

RA: oh

P: hee you are stupid things like that you are not circumcised you are like your child

RA: mhm

P: you see that’s where I managed to get a way that as long as they told him in public I will tell him in private

RA: ok

P: just the two of us that this thing let’s do this

RA: and go for circum its doesn’t sit well with him because he was toll in public

RA: yes

P: you see that’s where I managed to get through to him

RA: so but when you started what did you say to him

P: when I started

RA: yes

P: he is the one who started it

RA: oh ok

P: it was started by him we were sitting and he told me that like the past the way that girl was telling him

RA: mhm

P: and he said you know that thing does not sit well with me imagine a person telling me that I didn’t go for circum

RA: mhm

P: and my son hasn’t gone for circum

RA: mhm

P: and then I say if you look at it do you think its sharp for you not to go for circum

RA: mhm

P: then so he said no this thing is culture at home there is no one that comes from there even my older brothers no one has gone for circumcision

RA: mhm

P: I said it’s the Zulu culture and he said yes

RA: mhm

P: and I said but we are no longer living that old life

RA: mhm

P: now we live as like it’s the modern days we have to be on the same procedure that diseases are coming as like day by day comes a new disease

RA: mhm

P: that is different that we don’t know where it comes from what if you are going to be the first person to catch diseases

RA: so this thing that they insulted him didn’t sit well with him

P: yes they insulted him

RA: yes

P: it’s what makes me enforce that and to have power that I can talk to him because he was insulted in public

RA: ok so but if he wasn’t insulted in public did you know that he hasn’t circumcised

P: I didn’t know

RA: ok

P: you see

RA: oh but you already had sex with him

P: no

RA: oh

P: we were never we… I only knew after I had sex with him and already they had insulted him before I had sex with him

RA: oh ok

P: yes after they insulted him then I saw it and I saw that there is no need to leave him and say no because he is not circumcised I can also help him to go for circum so that they stop insulting him

RA: mhm

P: you see

RA: so if they didn’t insult him err… how were you going to talk to him

P: if he wasn’t insulted I was going to talk to him as you know if a man is yours you can there are many ways that you can use to communicate with him

RA: mhm

P: he is my man I know him and im his woman he knows me

RA: mhm

P: I think it was going to be simple cause I was going to start when we are relaxed in a good mood I was going to start the topic

RA: ok so what is it that a person doesn’t need to mention when trying to encourage someone a man to go circumcise

P: not to mention

RA: mhm like things they need to avoid talking about

P: like insults

RA: mhm

P: you understand like telling a person that men that are not circumcised

RA: mhm

P: are like this they are weak

RA: mhm

P: things like these you are not supposed to mention words that mean nothing

RA: mhm

P: you have to tell them facts about why they have to circumcise

RA: mhm

P: is it and that how it will help him

RA: ok

P: you see

RA: so I hear you saying we have to avoid insults and not tell them about men who went for circumcision so do you think that a man that is not circumcised is weak or what and uncircumcised man

P: mhm ya I think that a man that is not circumcised is weak as I have explained at the beginning that diseases

RA: mhm

P: he catches diseases quickly

RA: ok

P: ya

RA: oh he is weak because he catches diseases quickly

P: yes diseases quickly as they explain to us on the TV programs that a person that didn’t go for circumcision catches diseases quickly

RA: ok so you are saying that the one who went for circumcision doesn’t catch diseases quickly

P: no

RA: ok so it means that one is strong

P: I think the one that is circumcised is strong

RA: oh against diseases

P: against diseases

RA: ok so do you think err… your partner if it was him who came to you and said I want to circumcise err… how were you going to feel

P: yoh I don’t know you know if ever he came to me

RA: mhm

P: how I was going to feel

RA: yes

P: I was going to be happy

RA: mhm

P: I was going to be happy too much

RA: mhm

P: I was going to be happy that at least there is something that he is thinking and he is going to save me

RA: mhm

P: cause you know these days a person will tell you hee a condom is not good for me what, what, what hee this and that

RA: mhm

P: you understand that if a man says hey baby I want to circumcise I wouldn’t even have stress I was going to be happy ye happy

RA: so I hear you saying he was going to save you how was he going to save you

P: he is going to help me not to get diseases isn’t it diseases if ever he has diseases its simple for me to get them

RA: yes

P: cause I have sex with him it’s going to be easy for me to get them

RA: ok err… (coughing) for people in a relationship who do you thing is responsible for raising the circumcision topic

P: for people in a relationship

RA: yes

P: I have to according to me as a woman because a woman grabs a bull by its horns

RA: ok

P: you understand if maybe you are going around maybe doing research

RA: mhm

P: as a woman if you stop a man he won’t refuse

RA: ok

P: ya so that’s why I’m saying a woman has to tell her man that papa let’s do this lets do this

RA: mhm

P: then wait for his feed back

RA: ok so you can support him when he comes and tell you so

P: yes I can support him

RA: ok so you can support him

P: mhm

RA: so why do you think it’s supposed to be a woman

P: why do I think

RA: mhm

P: it’s because a woman eish I don’t know how to explain

RA: mhm

P: a woman can approach a man

RA: mhm

P: it’s even if they say a man is not easy to approach but a woman will be able to get to him

RA: mhm

P: and end up talking to him

RA: mhm

P: and end up putting him a position that she wanted

RA: oh so you think women have to start the topic of circumcision

P: ya women have to get the strength you know what our men have to go and get circumcised

RA: so as you ok maybe for you it was easy as err… you man was insulted so as he was a person who didn’t want to hear anything about circumcision do you think it was going to be easy to talk to him about this thing as e didn’t want to get circumcised

P: as I think it was not going to be easy but there is this thing called anointment if a person doesn’t want a thing you keep on nagging as I know he doesn’t want it I would try different ways I would talk about it almost every time I’m having a conversation with him I would say darling what do you think about this thing you see

RA: so besides talking what are other ways that you think a person can try to encourage a man to go and circumcise

P: besides talking

RA: mhm

P: err…

RA: besides talking to him

P: besides talking to him

RA: let’s say maybe if you want to talk to s person and he says no and don’t want to hear anything what are different ways that you can try to use to get to him

P: I think a mouth conversation is better cause its whereby we share feelings

RA: mhm

P: you see so if ever he doesn’t want to talk I don’t know what I would do

RA: so now ok I hear you say it’s easy to talk to your partner about circumcision if maybe it is your brother or your father do you think it would be easy how do you think it would be like to say there is thing called male circumcision

P: my brother yes I can approach yes cause I come after him

RA: mhm

P: we share a lot of things

RA: mhm

P: if ever they say you go to and tell your brother I can

RA: ok

P: I can go and say hey brother there is this thing so and so (clearing throat) I’m doing such and such so as I was reading they told me about this ting of circumcision have you gone for it

RA: oh

P: if he says no and then I’ll ask him are you not interested on this thing you see it means that you can get help if you can do this you see eish with my dad it’s going to be difficult for me

RA: how is it going to be difficult

P: eh

RA: yes what comes to your mind when you think of dad

P: what comes to my mind is that he is an old person

RA: mhm

P: you understand it’s my dad unless to get through to him using my mom I’m scared of confronting him straight I will go through my old lady and say mama it’s like this as you know has papa done such a thing

RA: mhm so what are the reasons that you cannot go to papa straight and talk to him

P: the reason is that he is old and he is my dad

RA: mhm

P: it’s my real father

RA: mhm so your brother isn’t old

P: he is old but between me and him we share a lot of things so brother is better that dad papa eish and maybe he can even kick me out cause it will be like I’m disrespecting him he is going to think this one went to school just a few days ago and she is starting to tell me about circum whereas I’m the one who brought her here to life

RA: ok

P: I brought her to life without even doing those things so why now why is she telling me about such things

RA: ok so if it’s just another person who is old

P: mhm

RA: can you go to them

P: yes hundred percent

RA: yes… why is it err… you can go to another err… but with daddy

P: like let me put it like this if it’s just another person

RA: yes

P: just another person you can approach as I am simple

RA: ok

P: I can approach I would come up with different ways I know that if I just spoke to them and there is something that didn’t sit well with them they will come back and tell me hey sister there is something that I don’t understand or I’m not fine with this

RA: mhm

P: so my dad eish I cant

RA: mhm ok so err… (clearing throat) so how does male circumcision help on people in a relationship

P: how does it help

RA: yes why is it important for people in a relationship for a man to go and get circumcised

P: I think that for people in a relationship that a man to go and get circumcised as I explained at the beginning that we have diseases that’s the core of the topic that these days people get sick for god’s sake and we are using condoms but we can’t trust them anytime it blasts so if it blasts you see it’s a mess

R: mhm

P: you see you will find that now we are all sick and in a mess

RA: mhm

P: so for two people I think it’s important to avoid diseases

RA: so which diseases are you talking about

P: STIs there is this disease I forgot it they say if ever it’s an STI they say if ever a man hasn’t gone for circumcision its simple for that man to contract that diseases but I don’t know how you get it

RA: mhm

P: so if ever you haven’t gone for circum you will get that disease

RA: oh ok

P: yes

RA: so err… do you think male circumcision is a good idea or a bad one

P: I think it’s a good idea

RA: what is it that makes you think so

P: err… if I am looking I see the circumcision at the clinic as good that the one in the mountain because if you have done it at the clinic people don’t die and don’t get sick

RA: mhm

P: you see than the one at the mountain I see clinic is good

RA: mhm

P: more than the mountain

RA: mhm according to the things that you have seen

P: that we have already seen and then ones we hear from the TV

RA: mhm

P: cause each and everything that is happening that happens that thing spreads that in this place so many kids died from the mountain they are sick hee in this place there are kids at the hospital form the mountain what, what

RA: mhm

P: yes

RA: ok so err… I’d like to take you back a little neh

P: mhm

RA: so I heard you telling me that err… telling me about your partner I heard you telling me about your partner that he can’t come to circumcise because he is always busy so what do you think are other reasons that make men not to come and circumcise

P: reasons that make men not to come and circumcise

RA: yes that could make them not to come

P: ok like there are men that are like as we speak

RA: mhm

P: they will tell you words that are simple hee I won’t be seen naked by another man and another man touching my private part hey I’m not going to be able to walk and I won’t be able to be with my partner

RA: mhm

P: yes they talk about things that are simple where as people agree a agree on it that hee my love let’s do this so that we can be right I’ll take care of you I’m here

RA: mhm

P: I won’t leave because you are in pain

RA: oh so they are worried that these men will

P: they will see their stuff

RA: so they prefer to be seen by whom

P: I don’t know but I prefer they be seen by men because they are also men

RA: oh

P: rather than to be seen by women

RA: and then I hear you saying that they are worried about their women

P: I’m going to spend the whole week not being able to walk feeling pain

RA: oh so they fear losing their women or…

P: they fear pain

RA: yes

P: and that a person is going to spend the whole week not being able to be with a woman

RA: not being able to be with a woman how not being able to be with a woman how

P: not being able to sleep with his woman

RA: oh without sex

P: yes without sex

RA: oh so it’s that men don’t want men to see them they are worried that err… (clearing throat)

P: they will have pains

RA: they will have pains

P: and that err… do this he has to sleep with his woman

RA: oh so they are worried about that

P: that thing

RA: so what do you think has to be done or changed to make sure that men don’t think of such things

P: cause you know what I see could be done is that there has to be people like you

RA: yes

P: there should be people like where I’m staying there is no electricity

RA: ok

P: so the TVs are scares

RA: I think there has to be a group

RA: mhm

P: that goes around where we stay talking to people

RA: mhm

P: just approach them in a good way and talk to them and share the information with them

RA: mhm

P: all of it in full you understand

RA: mhm

P: I think that would help rather than TVs because in many places there is no electricity so we see the TV after a long time

RA: mhm

P: so if there are people giving them information a person will always know this is what is happening and I heard from person that knows not hearsays

RA: mhm

P: you see that can encourage them

RA: ok so you think that these people err… it because they lack information

P: its lack of information yes

RA: mhm

P: that such a thing works like this so because if a person you tell him now when I look at it those who got the information came in numbers

RA: ok

P: to do it isn’t it

RA: those who knew who have the information and then now they have a clue that this thing works like this not they say

RA: so those who came where did they get the information when you look at it

P: most of those who came got it here

RA: ok

P: ya

RA: how did they know or…

P: like when you are sick maybe you have flu or something

RA: yes

P: you come to the clinic like as a man

RA: mhm

P: there are those nurses who ok they can as you, you are sick you have flu how many times do you get flu in a month or in a year

RA: mhm

P: when she sees that you get many times she will ask you if you went for circum

RA: mhm

P: isn’t it that there are questions that they ask you while they fill up and then she will end up talking to you

RA: mhm

P: and give you to see that hai man the way I see it its like this is caused by this and this so why don’t you go that side and they will help you with circum

RA: mhm

P: maybe you can be right

RA: ok so those who don’t know is it because they have never been to the clinic or what

P: ya there are those people whereby that person even if he is sick he doesn’t go to the clinic

RA: ok

P: when he is sick he doesn’t come he would rather if he has flu it will be healed while he is at home he doesn’t go to the clinic he will tell you that the clinic what, what he won’t go to the clinic

RA: so how do you think we can reach those people

P: my thoughts

RA: mhm

P: they must group people here who work with this circum thing

RA: mhm

P: and give them this information so that they take this and take it to people not that people must come here to get it because as people we are lazy straight we are lazy to go get information if they can take people maybe ten of them and say go two go there two go there its not everyone that will not listen

RA: ok

P: people lets say out of ten five percent will listen

RA: mhm

P: you understand others will be stubborn I’m not saying that but one will listen and fend up helping other where you are going

RA: mhm ok (clearing throat) thank you my sister neh

P: ok

RA: so its time for us to close this conversation

P: mhm

RA: but before we proceed err…is there anything that you would like us to talk about that I didn’t touch

P: no there is nothing

RA: mhm

P: yes

RA: ok

P: I think I have spoken im satisfied
