# Supplementary material for: “To speak or not to speak”: A qualitative analysis on the attitude and willingness of women to start conversations about voluntary medical male circumcision with their partners in a peri-urban area, South Africa
Source: PLoS One. 2019 Jan 25;14(1):e0210480. doi: 10.1371/journal.pone.0210480 (PMC6347244; doi:10.1371/journal.pone.0210480)
Supplement: S1 File — (ZIP) [file pone.0210480.s003.zip › QF022_QC2.docx]

PARTICIPANT ID (P): QF022

RA: okay so now that we have spoken that we are going to record this interview when we are talking, so do you agree that I record as we speak

P: mm. I agree

RA: ok. And then eh, I said we are going to do three things today

P: mm

RA: so the first thing I’m just going to ask you questions around this topic of circumcision and then you are going to answer me according to you knowledge neh

P: mm

RA: so the is no answer that is correct, eh that is correct or wrong, okay. So, but before we proceed can you just tell me about yourself

P: about me how

RA: anything that you can tell me about yourself

P: like, I want to know I’m telling you about me concerning…

RA: yaah

P: mm mhm

RA: anything that you can tell me

P: I am a woman who lives here at {} (participant address)…

RA: mm, okay

P: and I’m always around

RA: around

P: mm

RA: mm, so when did you start living here at {} (participant address)

P: I started in 2010

RA: oh

P: mm

RA: so where were you living before

P: I was living in {} (participant address)

RA: oh. Okay

P: mm

RA: where did you grow up?

P: I grew up there in {} (participant address)

RA: mm, mm

P: at {} (participant address) [inaudible]

RA: oh .okay

P: mm

RA: so is {} (participant address) and {} (participant address) different, how do they differ?

P: they differ too much

RA: yah

P: because the lifestyle

RA: mm, mm

P: …at home and here

RA: yha

P: they have a hug distance

RA: okay

P: the life in Gauteng is fast

RA: it’s fast

P: it’s fast

RA: okay. So why do you think it is fast? What is fast about it?

P: firstly I can talk concerning things like marketing like people who came here

RA: mm

P: to Gauteng looking for jobs, like if a person is going job hunting they just wake up and go, maybe to Olifants (place in Gauteng), its close you can even walk

RA: mm, mm

P: or walk to the station and catch a train with just a few rands

RA: mm, mm

P: but at home it is difficult

RA: mm

P: cause even where you are job hunting you find plaza’s and then…

RA: mm

P: that means you will work in the shop, whereby we know that in the shops the money is not enough

RA: mm

P: mm

RA: okay, but how was growing up in {} (participant address)

P: I can say it was sharp

RA: mm

P: cause what can I say? I was taught well, when I look at the kids here in Gauteng

RA: oh, okay

P: the way the life here is lived

RA: mm

P: cause here in Gauteng before a child reaches 10…

RA: mm

P: no if a person is a boy or a girl they she already a woman, she is no longer a girl, he is no longer a boy like

RA: yes

P: he already knows a lot of things that …

RA: mm

P: … we knew that it’s only for adults

RA: like? [laughs]

P: just like bow

RA: mm

P: you find that a child is still in grade 8…

RA: mm

P: …he already knows about sex, he knows vibes…

RA: Mm, mm.

P: things like that, off which at home those things are only done by older people

RA: Yaa

P: mm

RA: oho. Children are not supposed to know about these things

P: they do know about them they just hide what they know

RA: yaa

P: here in Gauteng it’s like everything is just done in public

RA: oh its done in public

P: mm, there is no such a thing as, plus there is this thing of…

RA: mm

P: …if you are a child and you come back from where you were in the morning

RA: yes

P: maybe they are walking her, the lady from…they don’t have stress if the lady from next door sees her and what she will say …

RA: mmm

P: no everyone just does as they please, life goes on

RA: okay, so, okay, but did you know about circumcision

P: mm, circumcision

RA: do you think it is also done in public

P: circumcision at home

RA: yaa

P: it was not done public

RA: ok, okay

P: mm, it was done in the mountain

RA: mm, mm

P: only in winter

RA: in winter

P: mm

RA: okay

P: we didn’t know what they are doing, we just knew that they went for initiation

RA: oh

P: yes

RA: okay. And then here

P: at {} (participant address) …

RA: mmm

P: … that is whereby even a child is aware of what is done in circumcision

RA: mm

P: unlike the way I grew up, at home we just knew that for a month or a month and two weeks men went for initiation, and then they come back on the day they do, even if you ask a person from there that “what are they doing”, they won’t tell you , he won’t tell you it’s their secret

RA: oh okay, so at home they go to the mountain

P: mm

RA: okay, but what is it that you understand about circumcision? Or the term circumcision, when you hear of it what do you understand

P: the term circumcision, I understand that is whereby…

RA: yah

P: …a man is going to get cut

RA: mm

P: his private part, the foreskin

RA: ya

P: of the man

RA: mm okay

P: and that is where they say we reduce the risks of…

RA: mm, mm

P: sexual infections [inaudible mostly]

RA: mm, mm

P: mm

RA: do you know how it reduces

P: mm, I hear that it reduces by, like if you have sex having… like

RA: yah

P: …that foreskin

RA: that thing they say it holds dirt

P: mm, mm

RA: and then if it’s not there that’s where they say like

P: the dirt will be there but it will be better

RA: mm, mm. Okay. So, you said here it is done in the clinic and then there it is done at…eh…

P: the mountain

RA: at the mountain

P: mm, mm

RA: are there other… another circumcisions different from these two

P: it is there because here it is done…

RA: yes

P: … it’s like all year through when I look at it

RA: oh

P: and then at home it is just winter

RA: okay

P: mm

RA: mm. okay. What differences are there between them or what similarities are there between them

P: I can say it’s the same because they are doing the same thing

RA: okay

P: … on the male part

RA: okay

P: and then, what is different I can say the one from here…

RA: mm, mm

P: …it’s done today and then that person goes home, at home

RA: mm

P: he will be still there, and we won’t even know what they are doing

RA: mm

P: and then he will come back here being sharp, but…

RA: mmm

P: … here you are able so see that this brother is from circumcision

RA: [laughs] how do you see

P: [laughs] you will find that they have hands in their pockets, it’s like they are trying to hold the trouser, you just see that, that one is from circumcision

RA: he is holding the trouser

P: mm

RA: why is he holding the trouser [laughs?]

P: I understand that they feel pains, that’s what I understand

RA: mm. so there are pains in circumcision

P: yes. That is what I understand that there are pains

RA: okay

P: maybe that is the reason at home they are keeping them there so that we don’t see them

RA: oh

P: they come back being sharp

RA: [laughs]

P: and then walk fine

RA: oh. Okay

P: [inaudible]

RA: oh so you think they stay there because of pains

P: yes, so that we don’t see those things

RA: mm

P: because they …

RA: mm

P: … told themselves that this thing is a man’s secret, it is don’t there and ends there. When they come back they are sharp

RA: yaa

P: mm

RA: mm, mm. Okay, so the… are there other ways that they differ

P: other ways?

RA: yaa

P: maybe the other ways are, if I look at it, it like…

RA: mm

P: … it is done in a way of using traditional herbs maybe … like …

RA: mm

P: … what can I say?... medical things. Maybe at home, I’m not sure, maybe they just do it themselves

RA: mm, mm

P: … with their herbs that they dig from the soil or what…

RA: yaa

P: … cause like it like it is being used…it’s that like…

RA: mm, mm

P: … what can I say?

RA: ay, the way that you can just put it…

P: eish, I don’t know how to put it, but the way I see is like maybe

RA: yes

P: e… they are different [laughs] …

RA: [laughs]

P: … even if I don’t know how to put this word

RA: of the herbs

P: yes, maybe there …and here it’s like they do it medically…

RA: mm

P: let me put it this way, I can say it is used medically

RA: mmm

P: at home in the mountain they are sure that they use traditional things

RA: mm, mm

P: and it’s like that

RA: mm, mm

P: mm

RA: okay. And then, at home you say it is done only in winter

P: mm

RA: do you think there is a reason for it to be done in winter maybe?

P: I think the reason for doing it in winter is because we have this belief that…

RA: hmm

P: … like things, we say in summer it doesn’t heal…

RA: yaa

P: … that is what I think is the reason for doing it in winter, we just think it doesn’t heal in winter

RA: yaa

P: you can get hurt in summer

RA: mm

P: it gets hot, and it oozes, and they are thinking that in winter it’s going to get dry

RA: mmm

P: ‘cause it is cold in winter

RA: hmm

P: that is what I think

RA: okay

P: mm

RA: so here… in Sepedi, this thing of circumcision, is it a law or it is only done by anyone who wants

P: in Sepedi this thing of circumcision started a long time ago it is a law, we know that we have to go…

RA: yah

P: … and circumcise but these days things have changed, others just…

RA: mm

P: … they just tell themselves that “I don’t believe in such things, so I’m not going there”

RA: mm, mm

P: but before we all knew that, no, it winter obviously its circumcision time…

RA: yah

P: … and then we are looking at oh, this is the size, it has to go for circumcision, these ones have to go

RA: by size what do you mean

P: isn’t it they don’t just take a child that is eight years or what

RA: oh

P: they look at around 12 years or what, those are the ones that go for circumcision

RA: okay

P: yes

RA: what do you think about the age? if a person… at what age do you think a person should circumcise?

P: according to me

RA: mm

P: … a person… actually a child… a man should be circumcised as a child

RA: okay

P: maybe…

RA: yes

P: … from the hospital after birth, maybe after five days or what…

RA: yes

P: …still a baby, for the sake of…

RA: mm

P: … them to not feel these pains, I understand that each and every part of the baby’s body…

RA: mmm

P: … is still too much soft, even if you turn the hand too much…

RA: mmm

P: … I don’t think they will feel the pain, if they can remove while still babies…

RA: ya

P: … I think it will be something that is better. That is what I think

RA: Oh. Okay. Okay

P: and then now if they are not doing so, before a person starts engaging in sex, maybe around 12…

RA: mm

P: … that is when they can take him for circumcision

RA: okay

P: mm

RA: if they are

P: yhooo

RA: mm

P: ay that one, that is something else, ‘cause if that person is already 40 this thing…

RA: mm

P: … if it is really preventing the risks of

RA: mm

P: sexually transmitted infections…

RA: mm

P: … he would have contracted a lot by now

RA: oh. Because…

P: … because they had sex many times

RA: mmm

P: mm

RA: okay. But what is it that you think… like are you saying in Sepedi it was a law that…

P: mm

RA: everyone has to go, so now, everyone gets to choose …

P: … they choose

RA: … so what do you think makes people who are from one area, under one law, but their thoughts are not the same about this circumcision issue?

P: I think it is because, like now…

RA: mm

P: … ey, a lot of people we have adopted the modern lifestyle, it’s that…

RA: ya

P: … if a person goes out maybe goes to school, going somewhere

RA: mmm

P: … and then he meets different people… and then adopt…

RA: ya

P: … other people’s lifestyles. So when he gets back home he changes, “no I no longer do this, I do that …

RA: mm

P: … I’m not doing this I’m doing that “

RA: mmm

P: mm

RA: oh, when he is with…

P: people who are

RA: … different people

P: … different

RA: oh, okay but have you ever thought of err… telling another person a male about circumcision? It can be your partner, it can be your sibling or y our child

P: I know that where we come fro if a child is around 12 years it can be a boy or a girl they have to go for initiation

RA: a boy or a girl

P: all of them have to go for initiation

RA: oh, even girls do…

P: mhm

RA: … go for initiation? Okay so what is the difference between the male one and the female one?

P: I can say the one for females is different from the male one ‘cause I can say the male one they cut what they are cutting and then…

RA: ya

P: … for female there is nothing serious that I can say is being done that is reducing

RA: mmm

P: … something ‘cause you will find that they differ you find that thy do elevens

RA: elevens

P: levels or elevens something like that you see

RA: yah

P: so they will just mark you with something on the thigh just to show that you went for…

RA: mmm

P: initiation

RA: mmm

P: so it doesn’t have like I can say

RA: o yah

RA: it doesn’t reduce or increase…

RA: oh

P: maybe ‘cause we have these thoughts that is a person is from initiation…

RA: mmm

P: …follows the law…

RA: oh

P: … mm. they become a real woman or a real man [inaudible]

RA: the law in what way

P: many people think like at home…

RA: mm

P: …firstly, it’s that a man, if you are not from initiation, maybe you are from circum…

RA: mm mmm

P: … not from initiation, there are things that men do like when they do like if maybe there is a ceremony at home …

RA: yes

P: … or somewhere, you will do what they tell you to do and others they will tell you go and hide first

RA: oh

P: and as a woman you find that I’m not from the initiation…

RA: mmm

P: … but you find that here are things that there is a…

RA: mm

P: … ceremony at home [inaudible merula?] they will just tell you, you cannot brew the traditional beer because you are not initiated

RA: reason?

P: yho, I can say I don’t know the reasons

RA: okay

P: I just found things done like that

RA: oh

P: I can tell you what the reasons are. I think they just tell themselves that

RA: mm,mm

P: she is not a real woman

RA: mm, so the women are just marked elevens

P: yes

RA: that’s it

P: yes

RA: oh okay [clears throat] so when you say a child around 12 years already knows, who tells him

P: like… you see as we are living in this community

RA: mm

P: it’s like at home many people still go for initiation

RA: mm

P: like each and every year we know that people go for initiation

RA: Mmmm

P: and then a child sees that so and so went there and then I will go on this time, they don’t know what is happening there

RA: mmm

P: … so and so went for initiation, and then when he gets to the age that he can see that… and we can also see that…

RA: yah

P: he is now getting clever and we tell him that next year he is going for initiation

RA: oh

P: mm [inaudible] he is going for initiation

RA: okay I want to know who is responsible for telling him in the family

P: everyone, you can even me or his mother or his sister…

RA: yah

P: …or whoever, I just say “you

RA: yah

P: no the things that you are doing you now have to go for initiation”

RA: [Laughs] what he is doing?

P: mm

RA: [laughs], okay so is there anyone you have told, a male

P: yes

RA: who was it was it a child or a partner

P: it was my aunt’s child

RA: oh

P: mm

RA: okay, okay I want you to tell me a bit about what happened when you told him, on the day you told him. How did you tell him and how did he react [inaudible]?

P: ai its like as I have told you that at home they have to go for initiation, you find that…

RA: yah

P: … like the time im going to tell him when I went out to the street…

RA: mmm

P: … at home there is a kraal …

RA: yes

P: … I found him with some girl and then I sai “lets go home” and we left and I asked him what he was doing with that girl and he say no sister

RA: mm

P: I was just talking to her and then I asked him what he was talking about and he said ‘no I want her to be…

RA: mm

P: …you sister in law” and then I said you see what you are doing you have to go for initiation

RA: okay

P: mm

RA: [laughs]

P: mm [inaudible 17:59] you are starting to behave like you want to be a man

RA: and then what did he say

P: no he didn’t have a problem, he just [inaudible] that yes, and it was that last year he wanted to go there then they told him that now there is not enough money to take you there

RA: ok, okay

P: yes, and now you find that when I told him that he say I also wanted to go others are already from there

RA: mm

P: he said he also wanted to go

RA: so there they require money

P: mm

RA: like you pay

P: mm, they do pay

RA: oh, okay. Mmm

P: and then when they come back there has to be like something showing at home maybe…

RA: mmm

P: a ceremony they rejoice and buy him a blanket…

RA: mmm

P:… and buy him a chicken they just buy what they can afford…

RA: yes

P: … and then

RA: oh there is a ceremony

P: yes a celebration that he is back

RA: oh

P: mm

RA: okay. Okay. But have you ever told a partner about circumcision

P: mm. I have never met a partner that has not yet circumcised

RA: oh

P: but I am sure that if I can meet someone that is not circumcised from…

RA: oh

P: now I can tell him straight there is something like this that is being done there

RA: [laughs]

P: … I think you need to go and ask about it

RA: yah

P: how are thing going

RA: mmmm

P: mm

RA: so what are the reasons that you gave that child about the importance of circumcision? What are the things that you told him about

P: like I did say that…

RA: mm

P: … they say it reduces sexually transmitted infections…

RA: yes

P: … obviously they will end up having sex

RA: mmm

P: I told him that I want him to go for initiation before he starts engaging is such things because he will get sick

RA: mmm. Oh you told him about the diseases

P: mm

RA: is that all

P: mm

RA: m. okay when you just think, lets say for a couple, who do you think is responsible for raising the topic of circumcision

P: partners

RA: yah. Between a man and a woman

P: yes

RA: okay, so do you think it is a woman that has to start

P: they can all start but…

RA: yah

P: … sometimes when im sitting with a man if he knows that…

RA: mm

P: …he is circumcised or not circumcised he wouldn’t start such things and then because I am

RA: yah

P: a person who always wanted to know, I can start asking him if he is circumcised

RA: [laughs[ ya

P: yes

RA: and then when he says no

P: when he says no I will ask hi about why he is not circumcised

RA: mmm

P: and when he gives me the reasons, I’m going to try showing him that I know that circumcision does this and that it reduces…

RA: mmm

P: … diseases, so how about it /?

RA: oh you will still tell him about diseases [inaudible again?]

P: yes

RA: oh okay so but do you think that a woman has to start the topic of…

P: yes she has to start because you find that she is staying with a person…

RA: mmm

P: he knows that he is circumcised and it’s not something that comes to his mind…

RA: mmm

P: … he is just thinking of other things

RA: mmmm

P: … and then you won’t know

RA: mmm

P: and then you will find out late [inaudible here?] you have to have sex, what will you do?

RA: mm

P: mm. you have to start as a woman and ask…

RA: mm

P: …’cause especially these days its no longer a secret

RA: mmm

P: previously I understand that it was difficult that you can start asking a person if they are circumcised

RA: mmmm

P: … and hen you wouldn’t know

RA: mm

P: mm

RA: and then, but what are other ways that a woman can try telling a man about circumcision? Like the things that she is not supposed to say if she suggests this thing to a man?

P: things that she is not supposed to mention

RA: mm. you said you told the boy about diseases that if he is circumcised it reduces the risks…

P: mm

RA: of diseases

P: mm

RA: what is it that you think a woman is not supposed to say? To tell a man that…

P: I understand that…

RA: mm

P: … if you come and tell him that it reduces diseases he will think that you are saying he has got diseases

RA: oh, if you tell him about diseases he will think that…

P: yes that maybe you think he has diseases

RA: [laughs]

P: “maybe you want me to”…for him to

RA: yes

P: … to reduce those diseases

RA: oh, okay

P: yes, maybe you can tell him the way it…

RA: mm

P: …a person will say” ey eish an uncircumcised man, if I am with him I don’t enjoy compared to the circumcised man.

RA: to enjoy what

P: like sex

RA: oh, okay

P: like, you don’t enjoy as…

RA: yes

P: …the way it is…

RA: mm

P: …you are with a person that is circumcised, I understand that maybe he will understand better, that for me to tell him about diseases

RA: okay, you have to tell him about sex issues

P: yes, so that you can make him understand

RA: oh

P: mm

RA: so you will think he will understand about sex [inaudible 23:46 better?]

P: yes

RA: oh, okay, and then, but when you just thin k, do you think circumcision is a good or a bad idea

P: I think it is a good idea

RA: Mm mm mm

P: like for me I did mention that I have never met a person that has never been circumcised, like when we are sitting as women we talk

RA: yes

P: a person would tell you “no a person that is not circumcised no”

RA: mm

P: … he is not doing the right thing

RA: not doing the right thing how? [laughs]

P: he is not doing the right thing in the blankets, that person would just go for one round, and then he goes to sleep

RA: oh, as women you talk of such things

P: yes

P: oh. Okay. [laughs] what kind of women that you talk to? Is it people you are close to or?

P: yes its like…

RA: yes

P: my friends or…

RA: mmm

P: … just maybe my friends we are used to like maybe if we are done with our chores…

RA: mm

P: … we just sit together maybe during the day…

RA: mm

P: …one day you find that we start a topic…

RA: mmm

P: … and explain about your life experiences towards the people that you lived with…

RA: yes

P: … and the ones that you live with

RA: mm

P: and then you find that there others have met with people who were not circumcised…“no that person no”

RA: okay, so, okay so when you are talking what is it that they say they like or dislike about a circumcised man

P: about a man that is circumcised

RA: mm

P: most of the people say a circumcised man…

RA: mm

P: …if you are with him in a room, in a house…

RA: yes

P: … it can happen that, maybe you are sleeping, maybe he…

RA: mm

P: he can do about three to four rounds upwards

RA: okay

P: but a man that is not circumcised, no they will tell you he won’t even get up to three rounds

RA: oh. Okay

P: what I’m asking myself is that what is causing that in a man that is not circumcised…

RA: mm

P: … not to get to where circumcised get to

RA: so do you think its true what they are saying or do you agree with it or not

P: I cannot say ‘cause I don’t haven’t experienced it

RA: [laughs] ikay

P: mm

RA: oh. So is there anything else that they say they don’t like

P: mmh

RA: there is nothing else

P: yes

RA: okay. And then you say this thing of circumcision is right because of what

P: like I said it reduces diseases

RA: mm

P: and they say the man can…

RA: ya

P: …like you enjoy when you are with him

RA: mm

P: you understand that yaa he is a real man

RA: mm mm

P: mm

RA okay, but for people who are in a relationship as you say a woman enjoys if a man circumcised…what are other benefits that are there for partners if a man is circumcised?

P: others that can be there

RA: ya, for the two of them

P: ah, besides that it reduces diseases…

RA: mm

P: … and they they enjoy, I don’t think of anything else

RA: those are the only two that you think…

P: mm. those are the ones I know

RA: oh, okay. But if ever, lets say a man is not circumcised and then he is the one who starts the topic of circumcision , as a woman how would you feel about that

P: I would feel good, I will understand that this guy didn’t understand all along, maybe he is from a family that is not doing that

RA: yes

P: I will think that he is now informed…

RA: mm

P: … he is now able to see something different than what he was thinking before

RA: m mm

P: okay, you will be… would that affect your opinion about him

P: I don’t think I will be affected

RA: m mm

P: it can change but it won’t change like …

RA: mm

P: … to take him in a bad way like he would be a good person that is like…

RA: mm

P: … maybe we will have a future together because a man that is not circumcised no… I don’t think I will ever be involved with him

RA: how would you know?

P: I won’t know until I ask him

RA: oh, so you will ask him…

P: I will ask him when we are sitting that …

RA: yes

P: “my love you and I are in love, have you been there”

RA: okay

P: mm

RA: oh, before anything you will just ask that…

P: mmm

RA: mm

P: mm. it is something that I have to know, if he is from there

RA: mmmm

P: mm

RA: so if he is not from there then it just ends there

P: haa, if he is never been there I will try find ways that I can use for him to end up agreeing with me, and if we don’t agree that is where it will end, but if we talk and agree, no we will continue

RA: mmm okay. Okay. So circumcision reduces risks on diseases, like what

P: mm, I understand that the diseases would be STI’s

RSA: mm

P: and then AIDS

RA: yes mm

P: I understand is those diseases

RA: okay

P: mm

RA: mm, alright so is there anything else that you feel we didn’t talk about regarding circumcision

P: I wan to ask you a question now

RA: [laughs]

P: ‘cause like I did mention that mm…

RA: yes

P: … circumcision reduces diseases

RA: mm

P: … it even makes a man and a woman to enjoy together…

RA: mm mm okay

P: …what is it that you know to add to what I have said? Are there others that you know that I don’t know with regards to circumcision

RA: well, today it’s about you…

P: it’s about me…

RA: … what you know

P: oh

RA: like I did say,there is no answer that is..

P: good or…

RA: ya the ones you know

P: okay

RA: okay. But like that is why I ask you if you believe that or not

P: the circumcision?

RA: that if a man is circumcised, sex is enjoyable for partners

P: mm, I did mention that I don’t have proof on that, I wouldn’t say I believe it or not

RA: mmm

P: mm

RA: okay. But, but are there things that you think of or that you are sure of according to your knowledge that you think you can do or you don’t know

P: about circumcision

RA: mm

P: no… I

RA: that is what .. the question that you have asked me where does it come from? I want to know where it comes from

P: where it comes from

RA: yes

P: no, like the question im asking you…

RA: mm

P: its from the time you were asking me…

RA: yes

P: … about the things that circumcision… the advantages of circumcision…

RA: mmm

P: … so I think that maybe they are many, not that im thinking of others

RA: oh, you think I can have more

P: yes

RA: no today im learning from you…

P: oh

RA: … so just take it as if I know nothing

P: yes

RA: so just take it as if I know nothing

P: mm

RA: what do you think I know

P: mm… im not thinking of anything else

RA: the one that you think I know and you don’t

P: ah

RA: its not there

P: its not there I just wanted to hear if maybe there can be another one that you know that I don’t

RA: oh. But then eh, which means we have come to the end of the first part, the one I said we will do today neh

P: mm
