# Supplementary material for: “To speak or not to speak”: A qualitative analysis on the attitude and willingness of women to start conversations about voluntary medical male circumcision with their partners in a peri-urban area, South Africa
Source: PLoS One. 2019 Jan 25;14(1):e0210480. doi: 10.1371/journal.pone.0210480 (PMC6347244; doi:10.1371/journal.pone.0210480)
Supplement: S1 File — (ZIP) [file pone.0210480.s003.zip › QF023_QC2.docx]

**PARTICIPANT ID:** QF023

**FACILITATOR:** Okay so, now, just as I...we said that we’re going to record our conversation, so do you still give me permission to record our conversation?

**PARTICIPANT**: Mm.

**FACILITATOR:** Okay. I want you to say it out loud.

**PARTICIPANT:** Yes, I agree.

**FACILITATOR:** Oh! Okay, okay. And then eh, I said we’ll be doing three things today, right? So, in the first part I’m just going to ask you questions and then you’ll just respond to the questions, eh, there’s no answer which is…eh, right or wrong, it all depends on your understanding, okay. So, and then before we proceed, what can you tell me about yourself generally?

**PARTICIPANT**: I love…I’m a person who loves people.

**FACILITATOR:** Okay. Mm.

**PARTICIPANT:** And I also love the truth.

**FACILITATOR:** Okay. Mm

**PARTICIPANT:** And if a person has wronged me I tell them immediately…

**FACILITATOR:** Oh. Okay.

**PARTICIPANT:** ...if the person has made me angry or whatever…I can’t be angry at a person.

**FACILITATOR:** Oh, you’re a person who talks now and imm…?

**PARTICIPANT**: Yes, immediately.

**FACILITATOR:** Oh.

**PARTICIPANT:** So that I may be at peace. When someone and I are angry at each other I’m unable to…

**FACILITATOR:** Mm. Mm.

**PARTICIPANT:** ...go through the day being well.

**FACILITATOR:** Oh. Okay. So, when you say you love people, what do you mean? What is it that you do that shows you love people?

**PARTICIPANT:** I don’t like seeing a person suffering.

**FACILITATOR:** Oh.

**PARTICIPANT:** Like when someone doesn’t have anything to eat…

**FACILITATOR:** Mmm.

**PARTICIPANT:** I won’t eat, and leave them hungry.

**FACILITATOR:** Oh.

**PARTICIPANT**: I make sure that I share whatever I have with them.

**FACILITATOR:** Mmm. Mm. Okay. So you get along with people.

**PARTICIPANT:** Yes.

**FACILITATOR:** ...in a way.

**PARTICIPANT:** Yes.

**FACILITATOR:** Okay. So, where do you live?

**PARTICIPANT:** {} (name of area)

**FACILITATOR:** You live here in {} (name of area)? Did you grow up in {} (name of area)?

**PARTICIPANT:** M mm, I grew up in the rural areas.

**FACILITATOR:** Where in the rural areas?

**PARTICIPANT**: {} (name of area).

**FACILITATOR:** Is {} (name of area) in {} (name of area)?

**PARTICIPANT:** Mm.

**FACILITATOR:** Oho. Okay. So, how was it like growing up in {} (name of area)?

**PARTICIPANT**: It was great, I enjoyed...

**FACILITATOR:** Mm Mm.

**PARTICIPANT:** ...because that’s where I grew up.

**FACILITATOR:** Mmmm.

**PARTICIPANT:** Mm.

**FACILITATOR:** Okay. But, what kind of things did you generally enjoy?

**PARTICIPANT**: It’s like I liked...

**FACILITATOR:** Mm.

**PARTICIPANT:** ...things we did in the rural areas, we liked going…to practice…

**FACILITATOR:** Mm mm.

**PARTICIPANT**: ...tradition.

**FACILITATOR:** Oh, okay.

**PARTICIPANT:** Mm.

**FACILITATOR:** What do you mean by tradition?

**PARTICIPANT**: Leboa, there’s this thing we call lebowa, we used to do the lebowa dance.

**FACILITATOR:** Oh! Oh, you danced?

**PARTICIPANT:** Yes.

**FACILITATOR:** Oh. Okay.

**PARTICIPANT**: We’d do traditional dances.

**FACILITATOR:** Mm mm, mmmm. Oh, so you eh you spent a lot of time around people in a way?

**PARTICIPANT:** Yes.

**FACILITATOR:** Okay. So, how is life in {} (name of area) different to life in {} (name of area)? From your point of view?

**PARTICIPANT**: It differs a lot…

**FACILITATOR:** Mm.

**PARTICIPANT:** ...because here...

**FACILITATOR:** Mm.

**PARTICIPANT**: ...if you can look at the youth in Gauteng…

**FACILITATOR:** Yes.

**PARTICIPANT:** ...they don’t have respect like those from rural areas…

**FACILITATOR:** Mm mm.

**PARTICIPANT:** You can differentiate between a person who is from the rural areas and one who originates from here in Gauteng…

**FACILITATOR:** Okay.

**PARTICIPANT**: Mm.

**FACILITATOR:** Through respect?

**PARTICIPANT:** Yes.

**FACILITATOR:** Oh, okay. What do you think is it that causes this difference?

**PARTICIPANT**: It’s because they grow up being…like here in Gauteng…there are a lot of things…

**FACILITATOR:** Mm.

**PARTICIPANT:** It’s because the youth here...they grow up in the streets, so there in the rural areas, no...

**FACILITATOR:** Mm mmm.

**PARTICIPANT**: ...you know nothing, you might find that there’s not even a tavern in a particular village, you might find there’s nothing there…

**FACILITATOR:** Mmmm. Okay. So, did you know about this clinic here in {} (name of area)?

**PARTICIPANT:** Yes.

**FACILITATOR:** Did you know that there was a circumcision clinic here in {} (name of area)?

**PARTICIPANT**: I started knowing last year.

**FACILITATOR:** Oh! You started knowing last year?

**PARTICIPANT:** Mm.

**FACILITATOR:** Okay. What is it that you generally understand by circumcision?

**PARTICIPANT**: Eh...I think it makes a difference because, a person who has already been circumcised…

**FACILITATOR:** Yes.

**PARTICIPANT:** ...is not like one who hasn’t been circumcised…

**FACILITATOR:** Mm mm.

**PARTICIPANT: .**..and when we consider the issue of infections…

**FACILITATOR:** Yes.

**PARTICIPANT:** ...it’s not likely that a person who has been circumcised will…

**FACILITATOR:** Mm.

**PARTICIPANT:** ...get sick.

**FACILITATOR:** Mm. Oh, it’s not likely that he’ll get sick?

**PARTICIPANT:** Yes.

**FACILITATOR:** Okay. And then...what about one who hasn’t been circumcised?

**PARTICIPANT:** One who hasn’t been circumcised...

**FACILITATOR :** Mm.

**PARTICIPANT:** ...it’s more likely that he can get sick and…

**FACILITATOR :** Mm.

**PARTICIPANT:** ...the person is prone to...he’s prone to getting sick.

**FACILITATOR:** What do you mean when you say the person is prone to sickness?

**PARTICIPANT:** Because he’d not have went there…every single thing which goes into him won’t easily get out of him because he is still…

**FACILITATOR:** Yah. Mmmm.

**PARTICIPANT:** Mm.

FACILITATOR: Like what kind of infections are you referring to?

**PARTICIPANT:** Infections such as...such as AIDS.

**FACILITATOR:** M mm.

**PARTICIPANT:** Such as...STIs.

**FACILITATOR:** Yaa. Mmmm. But, what is it eh...eh, what’s another thing you see as being important about circumcision?

**PARTICIPANT:** Eish...

**FACILITATOR:** You talked about the one regarding infections, right, that eh you don’t get infected easily? What other things do you think would benefit a person with…when he has been circumcised?

**PARTICIPANT:** That...some of them show that…

**FACILITATOR:** Mm mmm.

**PARTICIPANT:** …you’re a man.

**FACILITATOR:** You’re a man?

**PARTICIPANT:** Mm.

**FACILITATOR:** Okay. When you say you’re a man can you expl…

**PARTICIPANT:** Because if you haven’t been circumcised a lot of people don’t regard you …as a man.

**FACILITATOR:** Oh! People.

**PARTICIPANT:** Mm.

**FACILITATOR:** Okay, why don’t they regard you as a man?

**PARTICIPANT:** You’d not have been circumcised…those who’ve been circumcised know…

**FACILITATOR:** Mm.

**PARTICIPANT:** ...that…well a person who has undergone circumcision…

**FACILITATOR:** Mm.

**PARTICIPANT:** ...is in such and such a way…

**FACILITATOR:** Okay. Can you explain the difference between these two people to me? How do they differ?

**PARTICIPANT:** How they differ?

**FACILITATOR:** Mm.

**PARTICIPANT:** Aa, mm, how should I put it?

**FACILITATOR:** Whichever way you’re able to, in a simple way.

**PARTICIPANT:** Oh! Often times, the one who has undergone circumcision…has respect.

**FACILITATOR:** Mm mm.

**PARTICIPANT:** ...and he’s like scared of a lot of things.

**FACILITATOR:** Okay.

**PARTICIPANT:** Mm.

**FACILITATOR:** Like what is he scared of?

**PARTICIPANT:** It’s like sometimes...

**FACILITATOR:** Mm.

**PARTICIPANT:** ...when he thinks of doing something, like...

**FACILITATOR:** Mm mm.

**PARTICIPANT:** ...even when he thinks of having sex with person, he starts thinking that “by the way…

**FACILITATOR:** Mm.

**PARTICIPANT:** ...[inaudible] encountered a problem like this…”

**FACILITATOR:** Mm.

**PARTICIPANT:** ...he encountered…

**FACILITATOR:** Yah.

**PARTICIPANT:** ...when he’s already undergone circumcision he saw what they did to him…

**FACILITATOR:** M mm.

**PARTICIPANT:** So, he should...he knows that he should always be safe.

**FACILITATOR:** Oh! A person who has undergone circumcision has knowledge about…about being safe?

**PARTICIPANT:** Mm.

**FACILITATOR:** Oh, so one who hasn’t undergone it doesn’t have it?

**PARTICIPANT:** No. He doesn’t have the same knowledge as the one who has undergone circumcision.

**FACILITATOR:** Mm.

**PARTICIPANT:** Mm.

**FACILITATOR:** Mmm. So, this respect, when you say respect…you mean respect to…? Who does he respect?

**PARTICIPANT:** He can be respectful to his own parents…

**FACILITATOR:** Mm.

**PARTICIPANT:** ...or to people even...

**FACILITATOR:** Mm.

**PARTICIPANT:** ...it’s not likely that such a person can do…

**FACILITATOR:** Mm.

**PARTICIPANT:** things that are out of the line...

**FACILITATOR:** Mmmm.

**PARTICIPANT:** Mm.

**FACILITATOR:** So, you...the reason for him not to do things that are out of line is just…is it just because he is circumcised?

**PARTICIPANT:** Mm.

**FACILITATOR:** Oh, okay. So, when you say people are the ones who...see the difference, or that there’s a difference between a circumcised person and one who is not circumcised, is it something which comes from people or from a person who has been circumcised or one who hasn’t been circumcised?

**PARTICIPANT:** It’s something that comes from a circumcised person.

**FACILITATOR:** Just him personally?

**PARTICIPANT:** Yes. Him personally.

**FACILITATOR**: Mm mm. Okay. But what is it that you understand by the word circumcision? When we say circumcision, it’s…what do you think circumcision is?

**PARTICIPANT:** I think it’s something that helps men.

**FACILITATOR:** In what way does it help them?

**PARTICIPANT:** It helps them with a lot of things really, it’s like when they start…when you’re told that you’re a man, they’d have seen that you underwent circumcision.

**FACILITATOR:** Oh okay, oh okay, so if you haven’t went through it you’re not a man?

**PARTICIPANT:** No. You’re not a man, you’re half a man.

**FACILITATOR:** Half? [laughs].

**PARTICIPANT:** Yes.

**FACILITATOR:** Half in what way?

**PARTICIPANT:** You haven’t been to where other men have been to.

**FACILITATOR:** Oh, so what makes you to become a man is that you go where other men have went to [laughs]?

**PARTICIPANT:** Mm.

**FACILITATOR:** Oh, okay. So...do you mean to say that even if a 40 year old man hasn’t underwent circumcision then it means he’s not a man?

**PARTICIPANT:** No, according to me he’s not a man.

**FACILITATOR:** Okay. What do you think they get from undergoing circumcision…eh that makes them to be men?

**PARCTICIPANT:** Mmm

**FACILITATOR:** Mm.

**PARCTICIPANT:** ...they get, let me say the thing they do to them…

**FACILITATOR:** M mm.

**PARCTICIPANT:** ...is what makes them to have respect, for a person to respect himself.

**FACILITATOR:** Oh, yah..

**PARTICIPANT:** Mm, himself.

**FACILITATOR:** M mm okay, do you...if you know, can you explain to me what they do where circumcision takes place?

**PARTICIPANT:**  [Clears throat], eh I know that they remove…men have…

**FACILITATOR:** Yes.

**PATRTICIPANT:** ...like when he hasn’t been circumcised...

**FACILITATOR:** Mm.

**PATRTICIPANT:** ...his thing will have a somewhat long skin.

**FACILITATOR:** Oh okay.

**PATRTICIPANT:** Mm.

**FACILITATOR:** Yeah.

**PARTICIPANT:** And then when he comes back from being circumcised you will see that this one comes from being circumcised, you’ll be seeing his skin…

**FACILITATOR:** Mm.

**PARTICIPANT:** ...no longer being long.

**FACILITATOR:** Oh! So, what do they do so that the skin is no longer long?

**PARTICIPANT:** They cut it off.

**FACILITATOR:** Oh! They cut it off?

**PATRTICIPANT:** Mm.

**FACILITATOR:** Oh! Okay. Okay. But, how many types of circumcision do you know of?

**PARTICIPANT:** How many types?

**FACILITATOR:** Mm.

**PARTICIPANT:** I know two.

**FACILITATOR:** Okay.

**PARTICIPANT:** Mm.

**FACILITATOR:** Which one and which one?

**PARTICIPANT:** I know the one of…like this one of yours here at the clinic, and the…

**FACILITATOR:** Mm.

**PARTICIPANT:** ...traditional, the traditional one.

**FACILITATOR:** The traditional one?

**PARTICIPANT:** Mm.

**FACILITATOR:** So, how do you think these two types of circumcision differ or in what ways are they similar?

**PARTICIPANT:** I think they are different, the traditional one and the...

**FACILITATOR:** Mm.

**PARTICIPANT:** …this one, I think they are different ’cause...

**FACILITATOR:** Yah.

**PARTICIPANT:** ...the traditional one…here...the people there haven’t been taught about it…

**FACILITATOR:** M mmm.

**PARTICIPANT:** ...mm, then this one...

**FACILITATOR:** Yah...

**PARTICIPANT:** ...the people have been taught…and when they are done they give you a medical prescription of what you should do…what you should do.

**FACILITATOR:** mm, mm.

**PARTICIPANT:** Yes, with the traditional one they just do it and then smear you with whatever they smear you with, then they are done [inaudible]...

**FACILITATOR:** Yaa.

**PARTICIPANT:** And, if you don’t recover fully when you get to…

**FACILITATOR:** Mm.

**PARTICIPANT:** …when you had went to the mountain and you now get home…

**FACILITATOR:** Mm.

**PARTICIPANT:** ...you go to the clinic, so it would have been better if you had just went to the clinic at the first place…

**FACILITATOR:** Mm mmm. Okay, so, from your understanding what would you say they teach them here at the clinic? Do you know?

**PARTICIPANT:** It’s like before a person attends to a person, he’ll be knowledgeable…

**FACILITATOR:** Yah.

**PARTICIPANT:** Yes.

**FACILITATOR:** Like what kind of knowledge?

**PARTICIPANT:** That he should start...

**FACILITATOR:** Mm.

**PARTICIPANT:** …the things he should touch so that he can cut off that person’s thing…

**FACILITATOR:** Oh, yah.

**PARTICIPANT:** Mm. It’s unlike with a person who does it the traditional way, where they just say well this person is able to stay pure or he knows…

**FACILITATOR:** Okay, what do you mean by staying pure? …yes…

**PARTICIPANT:** Like with the traditional one you’ll be told that very old men will carry out the procedure…

**FACILITATOR:** Yes.

**PARTICIPANT:** ...this means the old women won’t be…they won’t be having any wives, or not having whatever…

**FACILITATOR:** Okay.

**PARTICIPANT:** ...they are the ones who do this thing…they are the ones who carry out the procedure…in the bushes.

**FACILITATOR:** Mmm.

**PARTICIPANT:** ...hence you’ll hear that they know how to stay pure, they mean that they are old people, they are the ones who are able to stay pure.

**FACILITATOR:** Oh! So with the traditional one is it just old people who carry out the procedure?

**PARTICIPANT:** Mm.

**FACILITATOR:** Oh, okay. So, with the one here at the clinic what is it that you say the person will be knowledgeable about?

**PARTICIPANT:** The person who’ll be doing it would have went…like let me say the person would have done a Course on that…he was taught.

**FACILITATOR:** Oh!

**PARTICIPANT:** Yes...on what you should do to a person.

**FACILITATOR:** Mm, mmm. Oh, the one, the one...don’t they get taught with the one at the mountain?

**PARTICIPANT:** No, they just...they don’t get taught, they just know how to treat their people, what they should do to them…

**FACILITATOR:** Mm.

**PARTICIPANT: .**..hence often times you’ll hear that people died in such and such a place…

**FACILITATOR:** Mm mmm.

**PARTICIPANT: .**..they died, or you’ll find a person not being able to do…

**FACILITATOR:** Yes, yes.

**PARTICIPANT:** …anything...but just telling himself that he is able to.

**FACILITATOR:** Mmm. Okay, so are there any other ways in which you think they differ or don’t you think there are some ways in which they are similar? The one here at the clinic and the one at the mountain?

**PARTICIPANT:** I see them as being different ‘cause, here at the clinic...

**FACILITATOR:** Mm.

**PARTICIPANT:** ...you do it only in a day and leave for home…

**FACILITATOR:** Yeah.

**PARTICIPANT: ...**eh [inaudible] you get medicine and recover from your own home, but the traditional one…

**FACILITATOR:** Mm mmm.

**PARTICIPANT:** ...burdens a lot of people ’cause you find them staying there two months, three months...

**FACILITATOR:** M mm.

**PARTICIPANT:** ...in the mountains...

**FACILITATOR:** M mm.

**PARTICIPANT:** ...and they get cold...

**FACILITATOR:** Yes.

**PARTICIPANT:** ...and hungry. Sometimes you find that the food isn’t enough…

**FACILITATOR:** Mm.

**PARTICIPANT:** ...but when you’re at home…the clinic one is great in that you do it today and go home…

**FACILITATOR:** Mmm, okay. So, do you think there’s a reason for them to stay there at the mountain for a long time?

**PARTICIPANT:** The reason for that, is that they seek to see if you’re recovering…

**FACILITATOR:** Oh, okay.

**PARTICIPANT:** …if you’ve recovered...

**FACILITATOR:** Oh, is the reason to see if you’ve recovered or what?

**PARTICIPANT:** Yes.

**FACILITATOR:** Mm. Okay. But you...have you ever thought about telling a male person about circumcision?

**PARTICIPANT:** Yah I sometimes go and ask.

**PARTICIPANT:** Mm.

**FACILITATOR:** Is there a person you once told about it, a male person?

**PARTICIPANT:** Yes.

**FACILITATOR:** It could be a partner, it could be a child at home, it could be your own child.

**PARTICIPANT:** Aa, I told my friend’s child…

**FACILITATOR:** Yah.

**PARTICIPANT:** ...but then he was with my friend, I asked that: “have you taken your child to get circumcised?” She said “I want to take him when he’s 16 years old”.

**FACILITATOR:** Mm.

**PARTICIPANT:** ...I said to her “16 years...

**FACILITATOR:** Mm.

**PARTICIPANT:** ...he’ll be too old”

**FACILITATOR:** Okay.

**PARTICIPANT:** Mm.

**FACILITATOR:** Mm mm.

**PARTICIPANT:** I told her it was better to take him now**,** while he is still young and naïve, because she won’t be certain of the fact that at 16 he’ll still be naïve. Because if he can starts dating…well…

**FACILITATOR:** Mm.

**PARTICIPANT: ..**.he won’t ever get right. When you take him, he’ll take longer to recover.

**FACILITATOR:** Oh, when he’s already dating he takes time to recover?

**PARTICIPANT:** Mm.

**FACILITATOR:** Oh, okay. So at what age do you think a person should get circumcised? From your point of view?

**PARTICIPANT:** Mmm, from my point of view, I think when he is ten years old.

**FACILITATOR:** Oh, at ten?

**PARTICIPANT:** Mm.

**FACILITATOR:** Okay…what about when he is already old?

**PARTICIPANT:** When he is already old he’ll still go [inaudible] because he is already old and his time has passed by, has passed by.

**FACILITATOR:** Yeah.

**PARTICIPANT:** But, according to me…

**FACILITATOR:** Mm.

**PARTICIPANT:** When he reaches ten he should already be circumcised.

**FACILITATOR:** Okay. Reason being he’ll recover quicker?

**PARTICIPANT:** Yes.

**FACILITATOR:** M mm. So is your friend a female or male person?

**PARTICIPANT:** It’s a female person.

**FACILITATOR:** Oh. Okay, so, okay, it’s...okay I’d like you to tell me a little about the particular day in which you told her child, about how you told him, and then how your friend reacted…how did you start…how did you start it.

**PARTICIPANT:** A**,** I just started, I said to her “it’s winter, and...

**FACILITATOR:** Yes.

**PARTICIPANT:**  …other children… come from…

**FACILITATOR:** Mm.

**PARTICIPANT:**  ...at the thing there, did you take your children?”

**FACILITATOR:** Yeah.

**PARTICIPANT:**  She said “No, I haven’t taken them yet”, I said to her “what are you waiting for?” and she said “I am waiting for them to get a little older”...

**FACILITATOR:** M mm.

**PARTICIPANT:** I told it to her like this, I said to her: “you’ll have...they’re going to experience some problems when they’re already old…it’s better that you take them now”

**FACILITATOR:** Yaa.

**PARTICIPANT:** ...she said: “shuuu, my children will suffer. They are still too young.” I said: “when he is still young, he’ll recover quicker”.

**FACILITATOR:** Mm, mm.

**PARTICIPANT:**  She said she’ll see if she’ll take them or what.

**FACILITATOR:** Mm, mm.

**PARTICIPANT:** But last year...

**FACILITATOR:** Mm.

**PARTICIPANT:** ...I told her last year, she never took them…

**FACILITATOR:** Mm mm.

**PARTICIPANT:**  ...it’s only this year that she took one of them…

**FACILITATOR:** Mm mm. Did she have any reasons for wanting to only take them when they are a little grown up?

**PARTICIPANT:** Ah, she had said, her reason was that she was going to take them to the rural areas. She said her cult…in their culture they still go to…to the mountain.

**FACILITATOR:** Mm. Yes, yes.

**PARTICIPANT:** I told her that: “there’s no problem with wanting to do as your culture says…

**FACILITATOR:** Mm.

**PARTICIPANT: ...**it’s better that you take them to the thing before…

**FACILITATOR:** Mm mm.

**PARTICIPANT:** ...to the clinic before, then they’ll get circumcised, afterwards when you have a thing you’ll go to…

**FACILITATOR:** Mm mm.

**PARTICIPANT:** ...to the mountain, take them to the mountain.

**FACILITATOR:** Oh! What tribe is she?

**PARTICIPANT:** Eh she…she is Zulu.

**FACILITATOR:** Is she Zulu?

**PARTICIPANT:** But her children fall under Bush, they fall under the Shangaan people.

**FACILITATOR:** Oh! In the Shangaan culture they go to the mountain?

**PARTICIPANT:** Yes, they go to the mountain.

**FACILITATOR:** Don’t they want...

**PARTICIPANT:** Every child...they want him to have his own name…the thing is when you go to the mountain they give you a name, when the person comes back he’ll render a poem, he’ll be praising himself.

**FACILITATOR:** Yes.

**PARTICIPANT:** Mm.

**FACILITATOR:** Oh, when you go to the mountain they give you…another name?

**PARTICIPANT:** Yes, when you go to the mountain they give you a name…

**FACILITATOR:** Mm mm.

**PARTICIPANT:** ...like when you get there they can give you…they can say you’re Ngwato, or they might say you’re so and so...

**FACILITATOR:** Mm mm.

**PARTICIPANT:** ...then when you start praising…you start with…

**FACILITATOR:** Okay.

**PARTICIPANT:** ...with that name of yours.

**FACILITATOR:** Okay, so when you come back you no longer use your old name…do you use the new one or?

**PARTICIPANT:** You still use the old one, but…

**FACILITATOR:** Mm mm.

**PARTICIPANT:** ...when they praise you, they’ll praise you with…

**FACILITATOR:** Mm.

**PARTICIPANT:** ...with the name of, with the name you came back with from the mountain.

**FACILITATOR:** The new one, okay, so, but under which circumstances do they praise you? Do they merely praise you or do they praise you under certain circumstances?

**PARTICIPANT:** Often times they praise you like...

**FACILITATOR:** Mm.

**PARTICIPANT:** ...when there’s something at home, when they call you…

**FACILITATOR:** Yeah.

**PARTICIPANT:** ...they aren’t supposed to call you using your ordinary name, they’re supposed to call you by…

**FACILITATOR:** Mm. Mm

**PARTICIPANT:** ...by your praise name.

**FACILITATOR:** Mm. Okay. So, but is there a reason for you to…for telling your friend to take her children because it’s winter? I heard you talk about having told her about winter?

**PARTICIPANT:** ...ah, I know that usually when it’s like cold…

**FACILITATOR:** Yah. .

**PARTICIPANT:** ...the person recovers much quicker.

**FACILITATOR:** Oh, when it’s cold a person recovers much quicker?

**PARTICIPANT:** Mm.

**FACILITATOR:** Oh, okay. Okay. But, the child...your friend’s child, did you tell him directly that the issue of…that there’s such and such an issue?

**PARTICIPANT:** No, I didn’t tell it to him directly. .

**FACILITATOR:** Oh, you only told your friend?

**PARTICIPANT:** Mm.

**FACILITATOR:** Oh. So, there’s no man you’ve ever told about circumcision in your life?

**PARTICIPANT:** Ah, sometimes at work, I just ask...

**FACILITATOR:** Yah.

**PARTICIPANT**: ...but they end up not telling me the truth, they say: “you want to trap us, you want to…” and I’ll say “there’s no use, I know what is it that you do there”.

**FACILITATOR:** Yes.

**PARTICIPANT:** ...yes they end up not telling me, they say: “we won’t tell you”.

**FACILITATOR:** Oh! You ask your colleagues?

**PARTICIPANT:** Yes.

**FACILITATOR:** Okay. What’s your reason for asking them?

**PARTCIPANT:** [Laughs] sometimes you just see, like some person doing crazy things…

**FACILITATOR:** Yah.

**PARTICIPANT:** ...it’s like the person talks about things which are out of the line…

**FACILITATOR:** Mm mm.

**PARTICIPANT:** ...so sometimes you’ll find me asking: “do you want to tell me that you have been circumcised though?...

**FACILITATOR:** [Laughs].

**PARTICIPANT:** ...because I don’t believe that a person who has been circumcised can say the things you’re saying.”

**FACILITATOR:** [Laughs] what things does he say ?

**PARTICIPANT:** It’s like…

**FACILITATOR:** Yah.

**PARTICIPANT: ...**[laughs] I don’t know what example to give. It’s like when you’re talking the person will just get off topic, it’s like…

**FACILITATOR:** Yah.

**PARTICIPANT:** ...eish I don’t know how I might explain this.

**FACILITATOR:** It’s when you...okay, when you talk about what kind of things?

**PARTCIPANT:** It’s like you find that...

**FACILITATOR:** Mm mmm.

**PARTCIPANT:** ...like we’re sitting around as women…

**FACILITATOR:** Yah.

**PARTCIPANT:** ...then a man just comes up and lies flat down in our presence, and starts rolling up and down in our presence…

**FACILITATOR:** Yah.

**PARTCIPANT: ...**you see?...

**FACILITATOR:** Mmmm.

**PARTCIPANT:** ...it’s such a person that you’d think that this one, he probably hasn’t been circumcised.

**FACILITATOR:** [Laughs] oh! Okay, so a man who has underwent circumcision doesn’t do such things as those [laughs]?

**PARTICIPANT:** No. It’s rare that you’ll see the person…I’m not disputing that, you might have underwent circumcision but still like hanging out with women, but…

**FACILITATOR:** Yah.

**PARTCIPANT:** ...you won’t do those things, you find laughs]...

**FACILITATOR:** [Laughs].

**PARTICIPANT:** ...a person just rolling about just here in front of you, not doing a single thing which really makes sense.

**FACILITATOR:** Mm [laughs] okay, but okay, the reason for you to know if they’ve been circumcised…the reason that made you to ask your friend about whether his child had been circumcised, what was it…what was the reason for you to think that the child should go and get circumcised?

**PARTICIPANT:** Oh, when wintertime approaches…

**FACILITATOR:** Mm.

**PARTCIPANT:** ...it’s like I was just thinking about when I was growing up, that…

**FACILITATOR:** Yah.

**PARTICIPANT:** ...when it’s May, May...June, people go to get circumcised…

**FACILITATOR:** M mmm.

**PARTICIPANT:** ...it’s like those who go to the mountain go to the mountain…

**FACILITATOR:** M mmm.

**PARTICIPANT: ...**those who go to the doctor go to the doctor...

**FACILITATOR:** Yah.

**PARTICIPANT:** ...so a thought crossed my mind that since she lived with…

**FACILITATOR:** M mmm.

**PARTICIPANT:** ...she has boy children and she doesn’t live with their dad, had they went to get circumcised or not.

**FACILITATOR:** Mmmmm. Mm, okay. So didn’t she take it badly That another woman is…is asking me about…about my children?

**PARTICIPANT:** No, she didn’t take it badly.

**FACILITATOR:** Mmh! Okay.

**PARTICIPANT:** Because, she said she’s taking the other one this year. She told me that, well…

**FACILITATOR:** Mmh.

**PARTICIPANT: ..**.she did…

**FACILITATOR:** Mmmm. Okay, but, what are your thoughts regarding couples, who do you think should be the first to talk about this issue of circumcision between a man and woman?

**PARTICIPANT:** Mmm, it’s a man.

**FACILITATOR:** Is it a man?

**PARTICIPANT:** Mm.

**FACILITATOR:**  Okay, what’s your reason for saying it should be a man?

**PARTCIPANT:** Because a man…obviously a man should pass there, he should go through circumcision…

**FACILITATOR:** Yah.

**PARTCIPANT:** ...it’s not a lot of men who don’t pass through circumcision…

**FACILITATOR:** Mmh

**PARTCIPANT: ...**and women, don’t....

**FACILITATOR:** M mm.

**PARTCIPANT:** ...only those women in the olden days used to go, now, no.

**FACILITATOR:** Oh! Women also used to go?

**PARTICIPANT:** Yes, in the olden days women used to go through circumcision.

**FACILITATOR:** Okay, oh, like how does it differ to male circumcision, the female one?

**PARTICIPANT:** How does it differ?...ay I don’t know how to explain it. They do differ. [Inaudible] they’re almost similar, actually they don’t differ because…

**FACILITATOR:** Mm.

**PARTICIPANT: ...**the one of...the female one, they differ in that the female one, here…it’s not there here at clinic…

**FACILITATOR:** Mm oh, okay. Yah.

**PARTICIPANT: ...**then the traditional one...

**FACILITATOR:** M mm. Yah.

**PARTICIPANT:** ...the women’s thing.

**FACILITATOR:** Oh! Okay. So, the traditional one, the female one and the male traditional one…are they the same or are there ways in which they differ?

**PARTICIPANT:** Yes, they’re the same.

**FACILITATOR:** Are they the same? Oh...do the women also go to the mountain, stay there for two months…that period of three months?

**PARTCIPANT:** Mm.

**FACILITATOR:** Oh, and since men are cut, the women…are?

**PARTICIPANT:** Ay, I don’t...know if [laughs], I don’t know what they do to them.

**FACILITATOR:** Oh! You don’t know about that aspect.

**PARTICIPANT:** No.

**FACILITATOR**: Oh, okay, and then…since you say you think that a man should be the one to start the discussion about circumcision with his partner…do you think that to a woman, if a man is the one who comes forth and says I’m thinking of doing such a thing, to a woman, how will that be received by the woman?

**PARTICIPANT:** Ay, it becomes okay.

**FACILITATOR:** Mm.

**PARTCIPANT:** Mm. Because this thing…you’d be wanting to go where other men have went.

**FACILITATOR:** Yah ah.

**PARTICIPANT:** Mm. It means he envies the men who’ve underwent circumcision. He also wants to be like them.

**FACILITATOR:** Mm, so how will you take a man who comes to you and says he wants to do such a thing?

**PARTICIPANT:** I won’t take him light heartedly, I’m going to encourage him to go…

**FACILITATOR:** Yaa.

**PARTICIPANT:** ...so that he becomes like other men.

**FACILITATOR:** Mm, mm. But do you think they might be a different if it’s a woman who starts the discussion about circumcision?

**PARTCIPANT:** ...Mmm, it’s there sometimes, often when a woman asks a man…about circumcision…a man tends to think she is disrespectful of him or…

**FACILITATOR:** Yaa.

**PARTICIPANT: ...**whatever.

**FACILITATOR:** So, there’s a difference if it’s the woman who started?

**PARTICIPANT:** Mm.

**FACILITATOR:** Okay. But, okay [laughs]. So, but, do you think if the woman is the one who starts to talk about this thing…in what way do you think she should put it across to her partner, or to a child or…?

**PARTICIPANT:** Mmm, you can start when he’s in a happy mood.

**FACILITATOR:** Ya a.

**PARTCIPANT:** Ask him if...if he has undergone circumcision yet…

**FACILITATOR:** M mm.

**PARTCIPANT:** ...ask him when is it that he’ll go and get circumcised.

**FACILITATOR:** Yah.

**PARTCIPANT:** ...and you should approach him in an engaging manner… so. You just say “yho”...

**FACILITATOR:** Mm.

**PARTCIPANT: ...**um...like if it’s a child...

**FACILITATOR:** Eyah.

**PARTCIPANT:**…you tell him that on the day which your father went through circumcision this is what happened…this is what happened. It’s like you just…

**FACILITATOR:** Yah, yes, yes.

**PARTCIPANT:**…in an engaging manner so that he doesn’t become discouraged.

**FACILITATOR:** Oh, you tell him a little story?

**PARTCIPANT:** Yes.

**FACILITATOR:** ...about when his father went...

**PARTICIPANT:** Yes.

**FACILITATOR:** Okay. And then if it’s a partner?

**PARTICIPANT:** Even with him, you can make an illustration that will bring up why he hasn’t yet been circumcised…

**FACILITATOR:** Mm.

**PARTICIPANT:** So that he’s able to be like other men.

**FACILITATOR:** Okay. You tell him that, eh, so that he’s able to be like other men?

**PARTICIPANT:** Mm.

**FACILITATOR:** Okay. But, what do you think are some of the things a woman shouldn’t talk about when discussing circumcision?

**PARTICIPANT:** Often...

**FACILITATOR:** Mm.

**PARTICIPANT:**...men will...will tell you that women know nothing about circumcision.

**FACILITATOR:** Okay, Mm.

**PARTICIPANT:** This is why they often don’t like it when you tell them about such things, you’ll hear him saying “why are you telling me because you haven’t undergone circumcision?”

**FACILITATOR:** Oh, he refuses since you haven’t been there?

**PARTICIPANT:** Yes.

**FACILITATOR:** You haven’t undergone circumcision…oh the female one?

**PARTICIPANT:** Yes.

**FACILITATOR:** [Laughs] oh, okay. So, men refuse to hear women out because women haven’t undergone circumcision, so they know nothing and can’t tell him anything?

**PARTICIPANT:** Mm.

**FACILITATOR:** Oh. Okay, but what I mean is, when telling a partner how would you suggest circumcision. You said when you tell a child you can do so through a story about what happened when his father went…when suggesting it to a partner…what is the thing which you think you shouldn’t touch or talk about? That go there because of this and that…

**PARTICIPANT:** Like...

**FACILITATOR:** Mm.

**PARTICIPANT:** ...when you tell him that...you shouldn’t tell him that he should go there so that they may cut his thing or what. You just say “go and you’ll see when you get there…

**FACILITATOR:** Mm.

**PARTICIOANT:**...you’ll see what they’ll do to you there.”

**FACILITATOR:** Oh! You shouldn’t give him the information that they’re going to cut him?

**PARTICIPANT:** Mm.

**FACILITATOR:** Okay, why? What do you think he’ll get scared of if you tell him that?

**PARTICIPANT:** He might not get scared sometimes, he’ll just tell himself that you’re being disrespectful towards him or…

**FACILITATOR:** Mm.

**PARTICIPANT:** [Laughs] or what…

**FACILITATOR:** Okay. When you say being disrespectful, what do you mean by being disrespectful?

**PARTICIPANT:** Like he’ll see that you, like...you take it as if he’s not a man.

**FACILITATOR:** Mm mmm.

**PARTICIPANT:** Mm.

**FACILITATOR:** Mm mm. Oh! Okay. Okay. But, from your own point of view do you think that circumcision is a good thing?

**PARTICIPANT:** Mm. Yes I think it’s a good thing.

**FACILITATOR:** Mm.

**PARTICIPANT:** Because circumcision prevents infections.

**FACILITATOR:** Yaa...

**PARTICIPANT:** And it also prevents the person...a person who has undergone circumcision and one who hasn’t, if you can observe them they don’t grow up the same.

**FACILITATOR:** They don’t…?

**PARTICIPANT:** They don’t grow up the same.

**FACILITATOR:** Growing up?

**PARTICIPANT:** Yes...it’s like they don’t live life in the same way…

**FACILITATOR:** Oh, okay.

**PARTICIPANT:** Yes.

**FACILITATOR:** Oh! Okay, what do you mean by that?

**PARTICIPANT:** Like, they are different...you’re able to see the person, that yeah this one has underwent circumcision.

**FACILITATOR:** How are you able to see him?

**PARTICIPANT:** You can see him through his actions, that this one...he really has underwent circumcision.

**FACILITATOR:** Okay. What do men who have underwent circumcision do really? Explain to me.

**PARTICIPANT:** Men who’ve underwent circumcision respect themselves.

**FACILITATOR:** Mm. What do you mean by that? I want you to put it simply, when you say a person respects himself, it’s when he does what?

**PARTICIPANT:** Like, it’s unlikely that he may bathe in front of children or people…

**FACILITATOR:** Yah. Oh, okay.

**PARTICIPANT:** Mm.

**FACILITATOR:** Oh, that’s respecting yourself?

**PARTICIPANT:** Mm.

**FACILITATOR:** Oh, Okay. But considering couples, how does circumcision benefit them? Both of them.

**PARTICIPANT:** It benefits them when they are going to have sex…

**FACILITATOR:** Yah.

**PARTICIPANT:** They don’t struggle before they [laughs]...

**FACILITATOR:** [Laughs] with what?

**PARTICIPANT:** [Laughs] if the man hasn’t underwent circumcision, he works first.

**FACILITATOR:** [Laughs] oh, eh yaa.

**PARTICIPANT:** He should work that skin of his first.

**FACILITATOR:** [Laughs] works it, [laughs] works it in which way? Heeeee?

**PARTICIPANT**: ...you’ll find that he has to fold it up or do whatever to it…

**FACILITATOR:** [Laughs]. Fold it? Okay. Oh, when he’s been circumcised since they remove that skin there’s no way that he’ll fix it up first or fold it or do whatever?

**PARTICIPANT:** Mm.

**FACILITATOR:** Okay, and then another benefit? How do you see that...mm.

**PARTICIPANT:** Another one is preventing...

**FACILITATOR:** Mm, mm.

**PARTICIPANT:** ...infections.

**FACILITATOR**: Okay, yah...Okay and then for a woman, how do you think she benefits from her man having been circumcised?

**PARTICIPANT:** Mmmm...she benefits in that, even infections won’t...

**FACILITATOR:** Mm mm mm.

**PARTICIPANT:** ...it won’t be easy for her to contract infections.

**FACILITATOR:** Oh, the issue of infections.

**PARTICIPANT:** Mm.

**FACILITATOR**: Oh. Okay, excuse me...Okay, but previously on you spoke about your friend, that she wanted to take her child to the mountain because it’s her culture, right…So, what do you think makes people from different cultures to think differently about circumcision issues?

**PARTICIPANT:** Mmmm...some take it that it’s in ancient times…others will tell you that they still believe in taboos…

**FACILITATOR:** What?

**PARTICIPANT:** ...a taboo, that a child should go to the mountain so that they might give him rules on how to live.

**FACILITATOR:** Mm, mm, mm.

**PARTICIPANT:** Mm. Others say if a child hasn’t been to the mountain then he won’t be principled.

**FACILITATOR**: Mm, mm, mm.

**PARTICIPANT:** ...that he won’t know what initiation is all about, often times they say circumcising is initiation…

**FACILITATOR:** Is initiation to circumcise?

**PARTICIPANT:** ...yes.

**FACILITATOR:** Like is it when they cut?

**PARTICIPANT:** Mm.

**FACILITATOR**: Okay, so it should be the mountain one only?

**PARTICIPANT:** Yes, when they get to the mountain, there’s something they teach them.

**FACILITATOR:** Mm okay, they do it because there’s something they get taught?

**PARTICIPANT:** Mm**.**

**FACILITATOR:** Oh, but you don’t know what it is.

**PARTICIPANT:** Mmm m, they won’t tell you what it is.

**FACILITATOR**: They won’t tell you [laughs].

**PARTICIPANT:** Mmmm.

**FACILITATOR:** Why?

**PARTICIPANT:** I don’t know, perhaps they told them not to tell people. You’ll never hear a person telling you.

**FACILITATOR:** Mmm.

**PARTICIPANT:** ...not even when...even the praise name of his, he will only recite it to you when he’s back from the mountain.

**FACILITATOR**: Yah.

**PARTICIPANT:** Mm.

**FACILITATOR:** Only that? Oh, he doesn’t talk about what happens there?

**PARTICIPANT:** Mm m.

**FACILITATOR:** Reason being?

**PARTICIPANT:** I don’t know…

**FACILITATOR**: What do you think might be the reason?

**PARTICIPANT:** I don’t know what is it that they might have seen which they

**FACILITATOR:** Mh mh.

**PARTICIPANT:** [Laughs] which they aren’t supposed to tell people of.

**FACILITATOR:** Mm. Mmmmm. Okay. Okay. Well okay, but then I think we are done with the first part. Eh, is there anything you think we haven’t talked about? As we were talking?

**PARTICIPANT:** No.

**FACILITATOR:** Is it not there?

**PARTICIPANT:** Yes.
