# Supplementary material for: “To speak or not to speak”: A qualitative analysis on the attitude and willingness of women to start conversations about voluntary medical male circumcision with their partners in a peri-urban area, South Africa
Source: PLoS One. 2019 Jan 25;14(1):e0210480. doi: 10.1371/journal.pone.0210480 (PMC6347244; doi:10.1371/journal.pone.0210480)
Supplement: S1 File — (ZIP) [file pone.0210480.s003.zip › QF024_QC2.docx]

PARTICIPANT ID (P) QF024

RA: will you allow me to audio record this interview

P: Yes

RA: Okay, so I just like to know err… a little bit about you where are you from where are you currently residing

P: err… me I was born in err… the {} (participant address) I grew up {} (participant address) I went to school there attend the University of the North North West University of the North West I got B com Accounting Degree I currently work as a senior book keeper for an organization called look and I live in {} (participant address).

RA: Ok so where you currently resending at {} (participant address) how far is it as far as travelling distance from here to the clinic.

P: err…is about twenty, twenty to thirty minutes’ drive from where I am to here.

RA: ok, and did you that there was Male sexual health clinic here in {} (clinic name)

P: no, no I did not know till my husband came here I don’t know I just knew that they are available I Just don’t know where exactly they are

RA: ok so now from where you, you grew up and now err… can you tell me what, what, what is it your understanding when we speak of the term male circumcision.

P: err… I think my understating is the same as everybody’s err… the removal of the foreskin err… some do it for traditional reasons err... in my family it has always been done for err…hygienic reason so our boys we don’t even take them to the mountain they just go to the hospital get it sorted out for hygienic reasons

RA: ok, ok so, so I just wanna now focus on that err…growing up has your family been one that always believed in err… circumcision and sending people to, to hospital to get circumcised.

P: I wouldn’t say the whole family I would say certain individuals err… within the family err…especial err...my aunties because they were nurses so for them they understood the medical err…reason and benefits of male circumcision so they would always encourage that the boys have go and get circumcised err...but other believed that they will sort themselves out once they are growing up if they want to go they can go If they don’t wanna go they do not need to go so it was always depends on which family member were talking about.

RA: Okay so, so the one that said they will sort themselves out any reason particularly reason why okay let them decide for themselves.

P: err... Because they had to sort it themselves out once they are grown err... think the environment in which they will grow up in issues of sex and sexual health where not discussed especially with parents so they also perpetuate that same err… behavior or say to say or attitude towards sex and sexual health so it was an issue of once they grow they will sort out themselves I don’t have to do deal with it

RA: mhm

P: ya err…those who are in medical profession however were like they need to know about it and I’m gonna tell them about it and am gonna make sure they do it if I have to do it when they two years old I will do it I guess that the different between two groups

RA: mhm

P: In my family

RA: So where at what age were they actually encouraged to go and circumcised.

P: err… I would say most my cousins were circumcised before they were two err… my brothers on the other hand they had to do themselves when they were already in their twenties because my dad never bother with that issues it was never a problem for him err…when they were teenagers I think my mother to broach the subject then he said when are grown they will do it by themselves I’m not getting involved with that so yeah that’s the age different between those have parents that would wanted done as soon as possible and those people like my parents whatever like that.

RA: mhm so, so with you, have you had experience where you probably try to broach the subject of circumcision with maybe your brothers or err...your partner maybe

P: I think with my brothers no with my partner yes err... where err...I could see the consent struggle especially when come to hygiene part of how he has to struggle on daily basis to keep clean and err...not getting Infections and things like that and that when I go to why don’t you and circumcised easier no more worrying about where what is hiding and what is hiding there so that when we brought the subject we actually I actually encouraged to say him go get circumcised you know I know it has not been in the practice with especially with his family but do it for yourself is better for you hygienically and that’s I think that the position where I was approaching it from the for hygienic perspective to say look here it saves lot of headache to say if, if am in the area there is no water I don’t have to worry about the fact that I haven’t taken a bath for two days I have any infections I have to get antibiotics and things like that for your health is just easier to do that so how it we broached the subject of male circumcision I think that was all about and also discussing the fact of saying what are we gonna do without children err… if we have a boy in the family how are we going to tackle the issue if their circumcision because for me hygienically clean its, its adamant that they do it and I rather do still young than wait when they are older.

RA: mhm

P: err…because is more painful when you are older rather do it when you are still on your nappies it became part of you, you do not have to think about it err... its something that you go up it was done and when you grow up and it was done so it was okay so that how the subject of male circumcision in my family was discussed.

RA: mhm and when you broached the subject with your husband what was his reaction at first

P: You know he, he was accepting of it err… I guess from he in a way he understood the benefits because I don’t just say err…you have to go get circumcised we discussed about it what are the benefits to him as an individual err…the fact that he doesn’t to worry about err…there is no water for two days he if he may err... so because it was not me forcing my opinion of him it was a discussion he was more accepting of it and understood the benefits of it better than if I have just said because I want it that way

RA: mhm

P: So he was very accepting of it and hence why he ended up err...actually do it.

RA: ok so now, now that you, you shared experience of the approach that you taken that you took to encourage your husband err… what advice you would give other females as far as them trying to err… their partners encourage or encouraging male males their families to circumcise what would be your advice

P: I would say don’t, don’t make sex and sexual health a taboo don’t make it a no go area because then there are lot of things that you are not going to discuss and should anything adverse happen you don’t even know how to broach the subject to say how do you now we have a situation how do we deal with the how do we talk about it how do we encourage each other how do we counsel each other about it so don’t make issues of sex and sexual health a taboo don’t make it this a no go area because then it makes it difficult to broach the subject when the time comes even with your children don’t make sex the enemy it’s a natural process talk about it even if you have to joke about it but make it part of the normal conversation as much as we speak about flu and,and,and pregnancy and things like that let’s not make se-, issues of sex and sexual health a taboo let it be an open book when you talk about it not just for the males even for females there is a lot of things with regards to my own err… sexual health that my parents never spoke to me about I discovered them somewhere along the way as I proceed it was not adverse for me but for other people it turned out very adverse and it affected negatively and just because issues of sex and sexual health In a family a no go are a taboo we don’t talk about it it’s not a black issue especially in black families we do not talk about issues of sex and sexual health we don’t talk about relationships you would not even broach the subject of dating with your mother or father or else you will get a clap from you know but that’s what makes it so difficult to speak about it err…and to actually get the message across because we build walls around sexual health or reproductive health err… health issues we don’t talk about them

RA: so now let’s, lets speak about a woman now who is err… in a difficult situation where broaching the subject is not easy an probably the husband does not wanna hear anything what approach would she take in just trying to err… get the message across and just trying to encourage the husband

P: I would say that find somebody that your husband trusts respects and speak to them so that they can broach the subject with him err… usually for husbands who are very unaccepting of it, it is better if a male speaks to them than a woman where if they have an uncle that they respect speak to the husband and say you know uncle I’m trying to broach this with my husband but I can see that its becoming difficult for him can you maybe speak to him about it that way it does not seem like you are trying to disrespect him because sometimes that’s what they think you trying to disrespect me you tell me what to do this is my house but when somebody that they respects speaks to them about it and say you know what err… even if they say that your wife is approaching me about this subject and I think she’s got a point look at it from this perspective they tend to be more accepting of it err… because then it is coming from somebody that they respect somebody that they look up to so for women who are in that situation find somebody that husband or that individual respects and trusts and speak to them about it

RA: ok and then in a case now where you’d advise on an approach that they should not use what approach should a women never use in trying to encourage their husband

P: never belittle him never ever, ever belittle him don’t compare him to other guys do not because that makes that guy to resist the minute you start *saying awufani no sbaniban* or why don’t you behave like u *sbanibani* that makes them rigid you can be coming up with a good idea but the fact that you are making them feel like they are inadequate or they not good enough or what they are doing is incorrect err… the only thing thats correct *yile eyenzwa u sbanibani* then you not going to get the message across so never try to belittle him don’t compare him to anybody …….and don’t speak down to them broach the subject with respect treat the man as if you are approaching the king make it a suggestion don’t make it a command such things your tone of your voice has to be revering not *ukuthi* *uyamesaba* but that there is respect in your tone because you must understand that you are broaching the subject of his personal health and a subject that is very sensitive for most men to speak about so that is what I think one approach that women should never us

RA: so earlier you, you spoke about traditional err…circumcision and versus your medical circumcision going to the hospital err… can you just briefly tell me what is you understanding about the traditional circumcision and what is the difference between traditional circumcision and medical circumcision

P: I have absolutely no knowledge of the traditional circumcision I don’t know anything about it except for what I seen in the news where people have lost body parts and died err… for me there hasn’t really been a strong advocate for the traditional method it worked in the past I’m not saying it didn’t work it worked in the past we are in a different station but right now there is a lot of diseases there is cross infections there is just too much at risk for us to continue with the traditional method err… whereas the medical circumcision for me it’s a fairly easy procedure err… in a sterile environment err… where your health is taken care of err… you are not being exposed to harsh environment its done in a control environment whereas the traditional one you are in the mountain in winter with very little to go on and that for me is what makes me recommend the medical procedure other than the other one and err… in fact the other one is a long process this one is quick and easy you carry on with you normal life you came whereas taking out six month of my life to go spend with a bunch of guys somewhere in the mountain and there is a risk of getting infections actually losing my penis in the worst case scenario dying is not worth it so that where I find the problem or the distinction between the two

RA: ok so can you tell me do you think medical circumcision is a good idea and why

P: I think it’s a good idea as I said previously for hygienic reasons specifically I wouldn’t I wouldn’t know for sexual reasons but for hygienic reasons for me its adamant if I’m in a place where there is no there is nothing for me that I can use to keep me clean I don’t have to worry about it for at least for a period of two days then I can get some water somewhere and start getting clean but if you are not circumcised chances of infections are numerous because throughout the day You are touching your penis you know your hands are not clean and it goes inside you know so for me that’s why it’s a good idea for hygienic reasons

RA: ok so for partners who what do you think are the benefits for a couple for a husband to circumcise

P: shoo I wouldn’t know really I wouldn’t know really what the benefits are for the couple err… maybe because I never really thought about it except for what is now bring encouraged in the media err… I wouldn’t know if sex is better with a circumcised partner or an uncircumcised partner so I wouldn’t really know the benefits for the couple I can only vouch to the benefits for the individual

RA: and now err… in a relation who, who do you think should take the responsibility of broaching the subject of circumcision

P: both

RA: ok

P: I think both partners should not just the subject of circumcision both parties should take the responsibility of speaking about their sexual err… relationship or, or their I wouldn’t just say relationship the whole issue about their sexuality it should both responsibility to talk about it because if it’s just one party it seems like you are nagging constantly you are talking about this thing you can see I’m not interested so why keep talking about it so both parties should take the responsibility to do so

RA: ok and err… what is your view of a man who decides to take a decision to get circumcised do you view him favorably or how was it when your partner decided I’m gonna take this decision and circumcised how did you feel how was your view

P; err… I wouldn’t say he took the decision it was the discussion and we decided when it should happen it was a process that we went through together it was ever a one person’s decision err… but I wouldn’t have viewed him less favorably because he made the decision to go because for me it was all about his personal health because if he is healthy I’m healthy my approach the same as with me if I’m healthy that means that he is going he is healthy so when I go and take care of my own err… women sexual health even though I do discuss it with him but it’s for me as well as for him so it’s for the benefit of both if I’m healthy that means he is going to be healthy so I wouldn’t view a person that took that decision in a bad light I would actually congratulate them for actually taking a stand to ensure that err… not only their own health but their partners health

RA: ok so now we are about to close this part of the interview err… but before we close I don’t know if there is anything else that you feel maybe you left out that you would like to discuss on this subject before we move on to the next section

P: err… not really I don’t think there is anything just maybe wish that err…such programs of, of promoting male circumcision and talking about men health had been there before now cause I think a lot of men would have made better decisions would have made much more informed decision about their sexual health that what they had at that time so I would say programs like these are needed especially for the older generation to have a more informed err…shall I say more informed approach to their own sexual health because you find that err… some men who had the procedure done in the hospital are being told that they are not men because they didn’t go to the mountain and the fact that you went to the mountain doesn’t make you a man so I think such programs where men are encouraged to speak about their sexual health and they are being told about the benefits of doing a and not b or doing b not c are needed so that nobody feels like they are not man enough because they didn’t go err… do the traditional methods or they went to the hospital or they are circumcised or uncircumcised I think just the whole informing people of the whole circumcision it should be something that carries on it should be just for this period of research

RA: and what advice would you give to us as researchers as far as we can go about err… going out there and informing the older generation

P: how do we use all platforms err… you know err… clubs the football clubs err… sports clubs err… churches anywhere where you can find men gathered use those platforms use social media err… to promote messages about health let’s not even make it about circumcision let’s just say as a man how do you take care of your health you know how just make it the health issue so that it’s not an issue because sometimes if you make it about a certain subject some people exclude themselves from so use all platforms that are available speak about men’s health and not just a specific topic within men’s health
