# Supplementary material for: “To speak or not to speak”: A qualitative analysis on the attitude and willingness of women to start conversations about voluntary medical male circumcision with their partners in a peri-urban area, South Africa
Source: PLoS One. 2019 Jan 25;14(1):e0210480. doi: 10.1371/journal.pone.0210480 (PMC6347244; doi:10.1371/journal.pone.0210480)
Supplement: S1 File — (ZIP) [file pone.0210480.s003.zip › QF025_QC2.docx]

PARTICIPANT ID (P): QF 025

RA: Will you allow me to audio - record the interview?

P: Yes…

RA: It’s ok. Now let’s talk about your understanding and you’re thought about medical male circumcision what do you understand by the term medical male circumcision?

P: It’s when a male has to remove his foreskin for him not spread mmm… for him not to get STI’s and HIV and for him to protect his partner, for him to show that he is independent and strong man.

RA: Ok, A strong man?

P: Mmm

RA: Ok what do you mean by a strong man?

P: *Angithi indoda* if like he is strong *angithi mawukhuluma naye* man becomes independent and strong through a woman *andithi?*

RA: Ja ok I understand you but can you try speak English as you preferred English it must be like that neh but feel free about that and don’t get disturbed by that, but try a little bit of English hence we had a preference of language if you preferred Zulu we were gona do it in Zulu throughout if you preferred Isipedi were we gonna do it in Isipedi throughout even in English it’s the same.

So ammm how many types or can you tell me about different types of medical male circumcision you know?

P: I know traditionally and western.

RA: Traditional and western? Can we talk about those two types what is happening in traditional and what is happening in western one?

P: The traditionally it’s when a man goes to the mountains and I don’t know much about full information about that one but the one western it’s when there is a counseller who gives before you start advise you how things operate for example I went with my partner they gave us steps to follow before he can be healed and the person needs be comfortable so that they must not be scared and you must not aaaa think whatever you do you ganna regret you must feel free as a person so for my opinion since I was the one suggested that he must come and circumcise he took a long time but he said he is gonna think about it but at the end of the day he come and did it.

RA: So you were saying the person was making you comfortable, how did she do that?

P: She was giving us that you know you can get tempted as a woman you have to be strong and whatever you do you are doing it for your own benefit not for somebody else and for your future, like so she was she was like her facial expression the way she was talking she had that thing that you have to believe it what you are doing is right and it’s not wrong, that’s how my partner became strong and think ok this woman is motivating us in a right way.

RA: What is the right way of motivating?

P: First of all is the facial expression like how the wards came out from her mouth and how like her smile towards the way she is talking to you guys.

RA: Ok so does a person ok, you also talked about the smile, is the person has to smile?

P: Not necessarily it depends on how you as person.

RA: Ok…

P: like for example before we had counseling it’s how we give you research and how you do it. It’s not necessarily about the smile but it’s the way you speak your words to them or to that person to do whatever they do like you keep your words like are you strong enough for that person to be motivated?

RA: How are the strong words for a person to be motivated, can give me an example of a strong words to motivate someone, can you just give me an example of a strong wards for motivating some one? Can you give me a clear picture of what you talking about?

P: A strong words?

RA: Yes when you are motivating remember you are saying when you are motivating you must have strong words like words like what?

P: Strong words are like we came here for a reason not just because you heard from somebody else or anyhow but strong words are like values you understand and beliefs those are strong words from my understanding.

RA: Can you give me those beliefs beliefs like what?

P: Beliefs is for example when we were growing up we could see ok there people who are going there that’s how our parents form home they taught us how we should believe what is male circumcision so from my own opinion as I was growing up we were doing it in a traditional way but as we were going to school and seeing it in aa… you know you understand the knowledge it was different from how we understand it ok this thing is a right way as we experience through growing around community and people talking about it for me it was like ok this thing I right. Not necessarily that you have to hear it from peer pressure or from what but I believe that I’m also benefiting for my own family because I’m not getting any younger and I’m not getting old. So from my own understanding as I was growing up I think it will be the right way to build my partner and to protect my partner and myself.

RA: Ok so ok you talked about from your experiences do you have some experiences in relation to circumcision?

P: *Ja* I have friends who like their partners don’t agree to do circumcision because they think it’s a wrong way it’s because of their culture they think it’s a wrong way a man does not have to listen to a female who has to suggest those kinds of things but as a women it’s how you approach your partner. You are the women you have to make a woman to be what you want him to be you have to have communication first of all he has to understand and he has to know you. So what if he is talking to you he has to understand that oo ok this person is saying it in a right way or a wrong way. From my experience I had friends that are saying their partners don’t agree to male circumcision.

RA: Ok what was their problem?

P: Ok most of them from where they were growing up they don’t believe in those things. So for a woman to come and tell them to do that it’s a wrong way they don’t listen to a woman first of all as long as he is a male everything goes his way he doesn’t have to listen to his partner.

RA: Mmm, ok but have thought about medical male circumcision have you thought about it?

P: Have I thought about it?

RA: Mmm,

P: Yes I thought about it.

RA: What was your influence? Why were you thinking about it?

P: It’s a right way because I have to fix him like I have to build a future because sometimes he can cheat with somebody else there and that person can spread that disease or whatever but not circumcised a man is man you goes to another person who is not circumcised a man is a man he can get tempted mmmm it depends on CD4 count or viral loud what goes to another person it might be in a dangerous way compared to the person who is giving the infection. From my opinion I’m advising some people already they mustn’t spread it

RA: Mmm ok so you are saying they might they might spread, how?

P: Aaaaa the bacteria’s that’s why I’m saying a man can get tempted, he might go to somebody else and cheat so without out knowing that the person is infected or whatever because he has a foreskin the foreskin traps the bacteria’s so whenever it goes to that person it depends on how the immune system that the other one has, so it can get worse compared to him.

RA: Ok I got you there. What would it mean in a relationship a man suggested that he wants to go for circumcision, what would it meant that a man In a relationship wants to go for circumcision , would it be any different if the topic is brought up by a women or not?

P: It depends most if it’s ok because most of all if you are afraid of your partner or is there communication it won’t be a problem a man can also suggest that he wan t to go for circumcision.

RA: So if it’s said by a female is there any difference if it’s suggested by a female?

P: It’s no difference it’s the same.

RA: Why are you saying that?

P: Because most of them … ok it’s how you for example may be you give reasons why you suggested it and he doesn’t want to talk about it and maybe he had peer pressure from friends who said that you are not a man enough when you are with your partner or woman but it’s from a women, woman understands and has a different knowledge compared to a man, if he suggest it’s a peer pressure from friends..

RA: If a man suggests its peer pressure why are you saying that, how do you know that its peer pressure?

P: Whenever men go they talk about those things for if he talk and I’m not circumcised they say you are a coward or you would say at the end of the day you want to prove a point.

RA: So you said women are more knowledgeable than man what kind of knowledge that a women have compared to man?

P: Man are… that’s what I was saying that ok a woman is the one that is making a man to be who he is.

RA: To be?

P: Who he is.

RA: Ok

P: To build the family most of the time a man comes to a woman and suggests curtain things and say am I right or wrong, if he an understandable male some will say ok since I because we have different background some will say ok since I grew up in this method a woman should listen to a male but if it’s an understandable male he would say ok let’s see what is his suggestion, that’s what I’m trying to say is most of the time a woman is the one who has a lot of knowledge compared to a man because a man can be knowledgeable but outside things change compared to a woman , a woman don’t get pressure most of the time.

RA: Ok you saying woman don’t get pressure most of the time, who are getting peer pressure?

P: Man …

RA: Why are you saying man get peer pressure?

P: Mmmm by trying to prove a point to other man…

RA: Ok you are also saying that woman don’t get pressure why woman are not getting pressure?

P: Ok if you are person you need to know where you going and most of the time you don’t like you have to know yourself and where you going but since you told yourself ok I don’t want friends and this kind of things so most of the times maybe a male will think whenever you don’t have friends your women made you like in a traditional way like bewitched you. He doesn’t want his friend to talk bad things about him.

RA: What are those bad things that guys friends talk about, can you give me an as example of those bad things that guys talk about.

P: For example they want you to have different partners mmmm alcohol.

RA: Ok its fine then, what could be some ways that a person like yourself could bring up the topic of medical male circumcision to your partner of a family member who is not circumcised how can you start?

P: How can I start ok I will give them the disadvantages and advantages. So I have to tell him what is he going to benefit, first of all child gets discipline from his parents so he has he doesn’t have to get it from bill bards or somewhere else he has to get it from his parents if this thing is right or wrong. So from my own opinion I think I will wait until he is a little bit old maybe he is 12 years so ok this person understand what I’m talking about so whenever he goes to his friends he will give it in a right way not when he is a little bit younger because it’s gonna be like I’m forcing him or something if he doesn’t want it’s his own choice but one thing I know I will be giving him in a right way the way I understand it and from my experience.

RA: Ok what are your experiences concerning circumcision?

P: My partner I found him and he was not circumcised so since I have people outside that male circumcision you have to do it in this kind of way, so I would tell him that before I meet your father I had those kind of reason but he didn’t like it at first but at the end of the day he did because he also wanted to be a better person.

RA: Ok so you told him to come and circumcised?

P: I suggested.

RA: You suggested that, why were you suggesting that?

P: I suggested so that he doesn’t get … ok he doesn’t get spread STI’s and HIV those kinds of things.

RA: How did you start, how did you put it to him as you are saying you suggested it to him, How did you tell him?

P: (The voice not clear) at first it was kind of I was joking to him then at first he was saying to me are you joking or serious? And I said I’m serious at first he said I will think about it I will give you my answers just don’t rush me about it. And then I said I will support you also.

RA: Ok so how did you feel about that?

P: It was ok.

RA: Why are you saying it was ok, what is it that he said or what was his response to that?

P: To make it more I was saying ok we will make a second baby and then he said ok I will go.

RA: That’s how you put it?

P: Yes...

RA: What is it that you say?

P: I said I will give you a second baby and then he said ok.

RA: Why were you why were you approaching it that way?

P: Like I know him I have skills so if I suggested it in a wrong way he was not gonna go so I put it in that way that we will make a second then he said its ok I will go.

RA: So you saying if you put it in a wrong way he was not gonna come, what is the wrong way of putting it?

P: Ok since I know my partner how he is if I would put it in a wrong way for example say be a man and do what what he was gonna get hurt obviously.

RA: He was gonna?

P: If I put in a wrong way he was gonna hurt and think ok this person she thinks I’m not a man enough for her then I put it in a right way and said ok I will give you the second baby.

RA: Ok if someone is putting it in a wrong way how is that person putting it? How to put it in a wrong way?

P: like in a wrong ok if I didn’t joke about the suggestion I suggested for example I was serious like you need to be joking not to be serious.

RA: Why do you have to be joking about it?

P: Ok like it depends on how you know your partner if it’s the person who is serious it depends on how he takes things, the conversations but since I know my partner is this kind of person when you talk things in a joking way then he knows that you are serious it’s the opposite for him.

RA: Ok that sounds interesting, so what approach of a person like you should avoid when suggesting that your partner or a family member must go for circumcision? What is it that as a female you should avoid saying when suggesting that your partner or a family member must go for circumcision? What is it that you mustn’t say?

P: Mmm I understand I must my choose words.

RA: You must choose your words, what do you mean by choosing words, like how?

P: It means like I can’t just say ok since I thought about it I just have to think how am I gonna start that person like my son, I have to think how am I gonna put it, will he agree or disagree it will depends from m y suggestion it’s how to approach you I have to choose words.

RA: What type of words to choose when you are suggesting this to man may be how can you say it?

P: Mmmm…

RA: Why why you must choose words when you are saying it?

P: Because may be if I say things in a wrong way he might get angry

RA: Why?

P: He would feel why women would tell me this kind of things, why would she suggest me to go male circumcised, why she would think all of those kinds of things, why would she think these kinds of things. So first of all I need to think why he go and circumcised, that’s what I’m trying to say I have to think and choose words.

RA: What is the main… why do you have to think when you are about to tell man this is it difficult of what?

P: It’s not necessary difficult it’s how the person she or he is. First of all I can’t tell my cousin or my uncle I won’t tell them because I don’t know them well I just know them because we meeting during weddings or those kind of things it’s not the same way as going to tell those kind of things because I had some friends I had friends at school whose different from my culture I once told him that my partner is what what those kind of things he told me he won’t go because isiXhosa and his parents told him never do that. So I respected his suggestion I didn’t tell him ok why because you want to get married to your girlfriend why don’t you do this because he is not my girlfriend but he is my friend it’s how you a approach that person don’t force everyone has a choice.

RA: Mmmm, Ok what is to force a person?

P: Ok for example it’s like it’s your own way not his way that’s the way of forcing.

RA: Ok you are forcing if it’s your way?

P: It’s your way…

RA: Ok can you give me an example of a person who is forcing

P: He wants that thing to go his way, for example since I spoke it it has to end that I don’t have to listen to your suggestion or whatever so it’s a way of forcing so you don’t want to listen to other person suggestion it’s the end since you said it it’s the end.

RA: How do you listen to other person’s suggestion?

P: To other person suggestion?

RA: How to listen to other persons suggestions?

P: It’s when they are responding to what you were saying.

RA: Ok alright, if not already mentioned can you tell me your experiences when you were trying to suggest the male to your family or partner, how did it go and how it happen?

P: He said he is gonna think about he will give me an answer I mustn’t rush him.

RA: Was that your partner or a family member?

P: My partner.

RA: He said he is gonna?

P: He said he is gonna think about it he said he is gonna give me an answer of what he thinks, I mustn’t rush…

RA: He said you mustn’t rush him, what do you think, why he said you mustn’t rush him what do you think?

P: The man I think he went to his friends to ask for an advice the way I know my partner.

RA: Did you rush him?

P: I didn’t rush him.

RA: Why you didn’t right him?

P: No it’s his own choice.

RA: Ok is it because it’s his own choice?

P: Yes

RA: What else that your reason of not rushing him?

P: I respect his I was gonna respect whatever answer you he was gonna give me.

RA: Why were you gonna respect whatever answers?

P: That’s how I am, I don’t like whatever goes whatever and whoever saying it from my understanding I will respect it and there is no other way.

RA: Ok alright, do you think that medical male circumcision is a good idea or a bad?

P: It’s a good idea.

RA: Why do you think it’s a good idea?

P: Mmmm from my opinion for male to reduce STI’s and HIV.

RA: What do you think are the benefits of male circumcision for couple?

P: Mmmm the the penis get a little bit larger because of the skin was covering it up… what are the benefit? Ok the sex is different.

RA: The sex is different; ok can we talk about your experiences when it comes to sex different, how?

P: Mmmm before before he was he was he didn’t have aaaa he was not that energetic after being circumcised he was more energetic and it was a bit larger than before.

RA: It was larger than before?

P: Yes.

RA: Ok what else are the benefits of male circumcision in a couple?

P: Benefits mmmm benefits we enjoy each other…

RA: You enjoy each other, why are you saying that?

P: (Laughing)…

RA: Ok mmmm in a relationship who do you think should be responsible to raise up the topic of circumcision, who do you think has to raise up the topic of male circumcision, is it a male or a female?

P: It has to be both…

RA: It has to be both, why?

P: Mmmmm first of all… I can’t say it’s a male of a female that’s why I say it has to be both because you will see the benefits of what your relationship for example do you think that partner is the one who think about the future or just the person you playing around with.

RA: Mmm ok so how would it mean if a man’s decision to be circumcised or what are your opinion of him as person would your opinion be more favorable to him or unfavorable to him or neutral?

P: Neutral…

RA: Why? Can you tell me more about you being neutral?

P: For example since my partner is different compared to my X or whoever when we got into a relationship we always said communication is everything, whatever we talk about he would say do you think my suggestion is right and whatever I suggest I would go to him and ask do you think my suggestion is right? Even if it’s not right it’s ok but one thing is that we have to agree in one thing.

RA: Why will you be asking him those questions?

P: Because he is my partner.

RA: He is your partner?

P: I respect his opinions and he respects my opinions.

RA: Do you have to respect his opinions, why do you have to respect his opinions?

P: To respect his opinions sometimes I might suggest something thinking its right and with my partner its wrong, that’s why I’m saying I respect his opinions.

RA: What kind of the opinion you respect?

P: Mmmmm money wise financial wise.

RA: What else?

P: Most I respect Financial wise.

RA: Why financial wise?

P: He is the breadwinner of the family. We have to draft budget before I can’t just say I want money for earrings with out when we doing budget you have to inform him before, he doesn’t disagree he will buy them but you have to draft everything and inform him before.

RA: Ok alright now it’s time to close up the first activity ,like I said to you that we have three activities and then we gonna go to the second activity but before we go there is there anything that you feel it’s important and you want to say it before we close this activity.

P: So far I’m covered…

RA: So far you covered, ok thank you very much about that lets go to activity two then I will explain to you how are we gonna do it. Ok can you explain your group one under barriers what is group one all about these cards?
