# Supplementary material for: “To speak or not to speak”: A qualitative analysis on the attitude and willingness of women to start conversations about voluntary medical male circumcision with their partners in a peri-urban area, South Africa
Source: PLoS One. 2019 Jan 25;14(1):e0210480. doi: 10.1371/journal.pone.0210480 (PMC6347244; doi:10.1371/journal.pone.0210480)
Supplement: S1 File — (ZIP) [file pone.0210480.s003.zip › QF026_QC2.docx]

Participant ID (P): QF026

RA: Will you allow me to audio record the interview?

P: Yes sister I agree to be recorded our interview today

RA: Okay thank you

P: Okay can you please tell me what are doing currently, maybe are working or is there anything that you doing?

RA: Sister I am a house wife I’m just sitting I am not working I was working before then I lost the job I am a mother who’s sitting at home now, I am looking after my house and the children.

RA: Were you aware of the {} (name of clinic) the one for us?

P: No it’s the first time for me today to come at {} (name of clinic) I don’t know.

RA: You never had about it?

P: I used to hear when they talk about it like HIV people when they come from {} (name of clinic) even those for circumcision they say they come from {} (name of clinic) but today it was my first time to come at the clinic

RA: Those who spoke about circumcision where did you met them, or they went to your place or you met them somewhere?

P: Those for circumcision when I first met them I was at SARS that is where they were talking and they said if you want to circumcise, to circumcise your partner or your child and come to {} (name of clinic) for circumcision it is for free.

RA: To circumcise.

P: Circumcision its better in Zulu. I heard them again at the clinic in {} (name of clinic) there was a lady who was speaking about circumcision and teaches about circumcision it means it is important that men should circumcise.

RA: What’s makes you think that the circumcision is important?

P: Eh the sister who was explaining there at {} (name of clinic) she explained very well she was not in a hurry and say it is important for men to circumcise and especially to circumcise in our living times circumcise us as young people who are leaving where there is too many diseases, so that is why you have to be clean actually circumcision makes a man to be clean when there is no foreskin.

RA: Okay do you have information about male circumcision?

P: No I don’t have information I am just learning what’s the important of getting circumcised and what is circumcision all about you see

RA: Okay when you say you are learning where exactly do you learn?

P: I am learning that it is very important for a man or whoever as long as you are a man or even if it’s someone close to you or it’s your own son he has to be circumcised.

RA: Why do you say it is important for your partner or your son, it’s important for them to be circumcised?

P: Supposed to be, I can say like the removal of the fore skin it’s the main thing that protects from getting diseases, even if is your male partner or even your child, let’s say your child is still young so he is just clueless about the diseases so at that time you don’t know what your child might be doing during that time you should protect him by getting him circumcised.

RA: Maybe if you are talking about diseases, what type of diseases are you talking about?

P: Those diseases that are sexually transmitted especially men, mostly them they do it to prevent themselves from getting sexual diseases.

RA: Maybe could you tell me more about these sexual diseases?

P: You protect from… I don’t know how they say it in Zulu for STI’s and HIV virus, yeah.

RA: Okay what are those things you saw that are important by the time people were teaching at {} (name of place) and SARS that made you realize the importance of circumcision?

P: The First things that makes the circumcision the important the person has to be…man during the time when a man gets circumcised he protects his female partner from the diseases because men usually have odd diseases like you get them there…on his thing where he get circumcised these are the things that affect us women like getting cervical cancer, lots of diseases even the discharge we get it from men we don’t have discharge, we usually get it from men, I just know it that way.

RA: Maybe you as women how do you get these things from men?

P: By having sexual intercourse with men.

RA: Then how do you get it?

P: By the time when the man is not circumcised there is…what can I say , a man is ejaculating , during the time he ejaculates there is something that remain in the foreskin and when it remain in the fore skin it stays there they say these things creates diseases to us females.

RA: So in other words if eeeh men are circumcised it’s not easy for women to get these diseases?

P: It’s not easy if your partner is circumcised, it is not easy for you to get the diseases ,because when your partner is done doing what he is doing he is clean like you become clean if he is circumcised he is hygienically clean he does not have dirt in his thing that’s in front.

RA: Maybe what kinds of circumcision do you know?

P: I just know the one where they remove the foreskin that is the only one that I know.

RA: You just only know the type where they cut the foreskin, where do they do the type of removing the foreskin the one you know?

P: It’s happening to men in the front.

RA: Then where it is happening and where is it done?

P: The one where they remove the foreskin it happens in the clinic.

RA: Where else? Is there any other place besides the clinic?

P: There is the one that is done in the rural areas, what did they call it? I forgot but there is the one that is done in the rural areas.

RA: Okay let’s talk about these two types the clinic ones and the one that is done in the rural areas, let’s first start with the one that is done at clinics how can you differentiate the clinic type and the rural one?

P: I can differentiate by saying the clinic process is much safer than the rural one. How safe it is? If you are at the clinic you first check if you are healthier enough to get circumcised like they first check your blood then they injects you on your thing. The one done in the rural areas I have no clue how it’s done I just heard that they are circumcised but I really don’t know how and I have never seen a doctor or a nurse checking them up if they right those who went for circumcision is their blood alright for circumcision you see.

RA: Maybe the one for rural areas where does it take place?

P: It takes place in the mountain, in the mountains it is cold , In the mountain its very cold and they also sleep there and no one is taking care of them and to check if they don’t have certain diseases as they are about to get circumcised and if it’s alright to get circumcised , at the clinic that how it’s done to the man and to check if your body stable enough because they check you and inject you and to see if are you good enough and in the rural areas they don’t inject you they just cut you.

RA: At the clinic as you say they check if you are good enough to get circumcised. What is exactly they look for in order to check or to see that your body is good enough to get circumcised at the clinic , what exactly are the things that they check in men if they are about to get circumcised , as you are saying at clinic you get checked if you are ready to get circumcised?

P: They check you at your penis if you don’t have any disease, like in males in front they have these white things they have to be removed and they also check your blood if it will manage the removal of the foreskin.

RA: In the blood what is exactly they check if you will be able to manage the removal of your fore skin?

P: They check if do you have an HIV maybe especially you blood have to have the energy in a person and if the blood able to do the job.

RA: There in mountain do you think that where all these things are done?

P: I don’t think in the mountain they can do those things and not that I don’t think it’s not done it’s just not done at all! Because in their own knowledge they know that we know that we only get cut if the blood has to be checked for HIV, your front part doesn’t have an STI’s they don’t check that they just did it.

RA: As a person who said you heard clearly the teachings at the {} (name of clinic) and you also heard people talking at SARS, have you ever thought about telling your friend about circumcision or a family member or maybe your son and thought that maybe one day you have to send him for circumcision?

P: Yes, I’ve been thinking as my partner he also came for circumcision, I was the one who told him about it.

RA: How did you started how was it, are there any challenges that you came across by the time you were telling him about circumcision or it was easy for him when you tell him to circumcise how did you tell him?

P: I can say my partner as a person that we are in love we are like friends, like there is no secret, we don’t hide anything whatever we are discussing we spoke about it so that it can help us in the future it doesn’t end here you see, “my husband what do you think about circumcision” he stood up and went for circumcision you see. Okay when I motivated him in the beginning I told him awu you know Zizo’father if you can go and circumcise we started as if we are playing if you can go and circumcise, “he said you are in a hurry” because he’s not a person who always in a hurry and “I said Ha! I am growing up now what if this thing is sick and I do not see and I don’t know and it is better when we remove what we called your polo neck it’s boring now” and he said “where did you get it?” Then I said “no I see polo neck is caring diseases at the clinic they said it must be removed because when we are doing there the polo neck carries the diseases it must be removed because it carries diseases and it’s been a long time that it carries to my womb and I become dirty, I will get like especially the cancer and you the cancer is killing”, then he said he will see. Then I ask him for the second time I said” there was those people who said circumcision is for free then he was laughing “then he said “no I will go beginning of winter they say in winter the healing process is quicker”, and then” I said I don’t know real then he came when he was right”.

RA: Eh when you are joking you call it polo neck, where does that name come from?

P: (Laugh) we use to… like I can say this foreskin normally close I don’t know if I can call that name it covers the man’s things so it covers the man’s thing it look like a neck and the polo neck and it shift backwards and do its job because it cannot do with the polo neck.

RA: Ooooh okay that is why you call it polo neck it is a closed polo neck so what does it close feel free to say all the words?

P: (Laughing) I am scared

RA: What makes you scared?

P: No (giggles) that’s why we joke about it we call it polo neck because it’s closed.

RA: As a person who spoke to your partner and you are the one who motivates him to go for circumcision what works for you to motivates him?

P: For me to protected from diseases yeah

RA: I want to know did you talk to him because you heard at {} (name of clinic) clinic when they were teaching and also when they were teaching at SARS so I want to know from you which was the easy way to encourage your partner to circumcise which was the easy way to motivate him to stood up and go for circumcision?

P: The polo neck to carry the diseases it will transmit you see it means if partner is sick obvious I will be sick because this polo neck carries the diseases. I will be transmitted even if I don’t have but I will be infected even if I don’t have because my partner carries his polo neck, actually this polo neck contain diseases

RA: Ooh it means you gave him reason that makes him to remove this polo neck.

P: Yes I gave him the reason to remove his polo neck because this polo neck carries STIs and dirt of all men even I if I am dirt that also goes to that polo neck actually this polo neck is a carrier of diseases

RA: Alright you said your partner was not interested in circumcision maybe what was the reason for him not to be interested in circumcision?

P: I can say the reason behind that it is because it is not his culture you see. Is it to other people it’s all about culture so him it’s not his culture. That is why I encourage him to circumcise because it is not his culture because everybody is affected with diseases it is not about the culture and when the diseases is there to the person it is no longer about culture .

RA: How do you do your culture?

P: The Swazi’s they don’t cut I will be lying.

RA: They don’t go for Traditional Circumcision or Medical Male Circumcision

P: Yes they don’t go for Traditional Circumcision or Medical Male Circumcision

RA: If maybe as it is; not his culture to circumcise if he’s the one who likes to go for circumcision firstly how will you encouraged him?

P: Personally I do not have a problem for circumcision because it something that was there long ago so for me it won’t be a problem

RA: Even if it was him who started to say he wants to go for circumcision?

P: I won’t have a problem with that

RA: If you encourage a man to circumcise what are things that you avoid when you encourage him about circumcision?

P: If you motivate I can say do not say he must go, let me say he must circumcise so that he’s able to be protected from diseases, and you know that most people they don’t want to use a condom, so if they don’t want to use condom and they want to be protected but they want to use condom, so that is why you have to tell him if you do not want to use condom go and circumcise so that he can be able to circumcise, you see.

RA: What is the reason for them not use a condom?

P: Most men they do not believe in condoms. I do not know the reason they do not believe in the condom use

RA: Maybe you never spoke to your partner to hear what might be the reason why they do not like use a condom?

P: My partner used to say when he uses the condom he doesn’t feel it; if he is using a condom if he is doing it without a condom he feels it. If he is using the condom no he refuse he says he doesn’t feel it.

RA: Why do you mean when you say he doesn’t feel it?

P: He actually means he doesn’t get satisfied with the woman (giggles)

RA: If he uses a condom?

P: Yes when he uses condom but when he’s not using a condom he gets the satisfactory.

RA: That’s what your partner says so?

P: Yes that’s my partner

RA: Others have you ever heard what their challenges are when they use condoms?

P: I don’t know about the others.

RA: But let’s say you are motivating a man to circumcise what are the things that you can avoid when you talk to him things that can make him to be scared to go for circumcision when you talk to him, what are the things that you can avoid when you talk to a man?

P: Firstly I can say he must go to circumcise so that we can get thee satisfactory firstly. Secondly he must go so that we can be protected from diseases .No one can agree that he got diseases but most of the time you have to encourage him and tell him that there are diseases even if he can say he can’t use a condom because I do not have diseases.

RA: For you as a man the way he’s talking to you as partner a person that you are in love a friend when he says if he puts on condom he doesn’t get satisfactory for you as women do you feel the same way when you are using the condom?

P: Yes there is that thing. You see when he puts on the condom there is a difference that okay today there is a condom still you can fell when there is no condom.

RA: From you as a woman?

P: Yes as woman , like you do not come quickly, you don’t come quick , like I can say you do not ejaculate quickly I can say so it takes time to ejaculates even if, let me say if you start doing it takes time when you put a condom to ejaculates when you don’t put the condom you quickly ejaculates.

RA: So when you say it takes time when you put a condom to ejaculates, when you didn’t put condom it doesn’t take time, what’s makes you happy as woman on that when you say its quick to ejaculates or as you are saying there is a difference can you explain?

P: I can say that’s what makes the two to be satisfied you see if one of you will not be satisfied and you left behind and the other one is quickly done the man, when you as woman you do not ejaculate there is …you fight because you do not go along and you have to be together you see.

RA: Do you also have an experience on that when you use a condom or not?

P: Yes

RA: You said that when you speak to your partner you motivate him that the foreskin keeps the dirty neh, those are the things that make him to be aware that it is important to circumcise for him to more encourage to take a decision to circumcise can you tell me what are the things that you can avoid things that are not right if I talk to a man if you want to encourage to go and circumcise things that will make him not to be interested in circumcision what are those things?

P: Things like circumcision is painful, there is also death you see there during circumcision when they circumcise there is a vein in the pelvic sometimes they do cut that vein and that will result in weak erection so those are the things that you should avoid to a man that they do cut that vein sometimes and that vein sometimes is not working you can lose your partner and to say there is death at the clinic of which is not true.

RA: Those things that you say you must avoid when you encourage a man like talking of pain, talking of death and the cutting of the vein during circumcision where does this happening?

P: It is happening in the mountain in rural areas that is happening.

RA: If you can motivate to medical male circumcision maybe are there any things that you can avoid that you cannot say it things that will make him not to be interested to be circumcised, those you just counted they are happening more in the mountain. Okay if you can motivate him for medical are there any things that you can avoid to talk about that can make him not be interested to be circumcised medically.

P: What can I say, on the doctor’s side it won’t be like the mountain on the doctor it is only one day it will not be like in the mountain and on the doctor they give you injection in your private part so that you will not feel the pain by the time they cut you can see. That is the difference this side I can say they just cut you but at doctors they gives you an injection so that you will not feel when they do the cut.

RA: Maybe if a person says I am scared of the injection maybe to a person like that, maybe you are talking to your brother or maybe you are talking to your partner they never had some questions about what is happening in medical male circumcision that will make them to doubt.

P: I can say, like he must go he will see there because I don’t think that an injection can kill a person. The injection I can say is one of the that is healing the person because if you can just cut without an injection it will be too painful and he will not tell other people to go for circumcision, but if you can say they are giving you an injection like this and that a person can be motivated to do it.

RA: As a person who got the information and you were able to use it to motivate your partner to circumcise, now in a couple or in a family who do you think can raise the topic about circumcision is it a female or a male?

P: I think it should be a man because a man knows better about being a man I can say so you see, woman they don’t circumcise people who circumcise are men so in a family a person who should promote circumcision is a male because is the one who knows the procedure you see.

RA: Maybe when you say supposed to be a man who knows about circumcision; if this person doesn’t have any information about circumcision so how can it easy for him to start the topic about circumcision?

P: It can be easy if is the one who went for circumcision first and heard how it feels like if he tells other people about something that he knows “I know circumcision I know because I did”

RA: But you were able to promote circumcision as a woman and you don’t circumcise and you said it was not that difficult even though your partner was a person who believes in tradition circumcision but you were able to motivate him as a woman.

P: Yes I did

RA: So why you are saying a man can be the one who promote circumcision but you were able to do it as a woman and you did it?

P: What helps me is because I used to listen you see, if I was not listening or I was a person who is ignorant when people are talking like to listen when people are talking, so I give myself a time and listen okay circumcision is not something that is difficult and it helps here and there not just circumcision only it also help you as a man.

RA: You spoke about the ways that helps a woman when a man is circumcised, we will talk about it, maybe other women what can they do as you did to encourage men or your partner, your child or any family member what are the ways that you can use to promote circumcision so that women can use to promote male circumcision?

P: People should talk about different things you see. You do not need to pretend as if you know while you don’t t know you ask when you don’t know after that you do it. Even myself I heard when they were talking about circumcision then I was able to talk to my partner and say circumcision is like this and that and he was able to feel and have an interest that I am interested to circumcise next year I will be able to go for circumcision and next year it’s like is too long it’s like you can go now you see, suppose we know that circumcision protects us from diseases.

RA: In other words it is very important that women should be informed about circumcision

P: It is very important that women should have information about circumcision. And also it is important to talk about it and say “my husband you are not circumcised go and circumcised,” because you will date a person who’s uncircumcised so it is important to tell him that my husband circumcision is very important because of this and this.

RA: You as person who spoke to your partner and explain that circumcision is very important because it prevents sexually transmitted diseases, other person maybe is talking to her partner and explain that circumcision is important because it helps to prevent sexual disease maybe it becomes difficult to this person to understand, maybe what can you advice, maybe what are ways that you can use to talk to a person so that he can be able to listen and understand the importance of circumcision, the person you explain to him that when you are circumcise it prevents you from sexually diseases but you find that the person is difficult to understand the importance, what are the things that you can do?

P: I can say the polo neck is smelling he must remove it, secondly when I look at the polo neck I am not happy me as person the polo neck doesn’t appetize me at all you see. You know when a person talks obviously he will say this polo neck is boring real it better to go and removed this polo neck because it means it bothers you.

RA**:** Do you think that can work?

P: It can work plus when you say it does smell not a single person will want something that smells

RA: So you like circumcision a lot because it helps you to prevent sexually transmitted diseases

P: Yes

RA: What else did it helps and you think it is very important?

P: Personally it helps me in sexually transmitted diseases other things I don’t see, mostly in sexually transmitted diseases you have to go and it

RA: If you are in a relationship or in love what are the things that you both benefit if the man is circumcised, as man or you as woman?

P: That’s what I and my partner make us to do sexual activity. Sex is the only thing that makes me and my partner to have a good relationship he doesn’t put the condom the fact remain what’s makes us to love one another is that we get satisfactory if we are not satisfied no I just leave (laughs). Because I do not reach where I want it is very important if you are in love with a person you have to get satisfactory you see.

RA: You said that when a man is circumcised you do not get the cervical cancer as a woman it reduces the chance to get it?

P: Yes

RA: When you talk about sexual transmitted diseases, for man is there any diseases that he is protected from when he is circumcised

P: There is, like HIV mostly because you get it through sexual intercourse that is the major diseases the others I don’t know

RA: So you said a woman can be saved because….

P: Even man can have a cancer a man can have a cancer, yes

RA: You think if he is circumcised…

P: It reduces the chance to have HIV and the cancer

RA: And then the woman?

P: Also it reduces the chances to get Cancer and HIV

RA: Alright maybe as you are saying that you and your partner you are like friends, a person like your brother do you think it was going to be easy for you to encourage him for circumcision as you were able to do it to your partner or it was going to be difficult?

P: It was not going to be easy to my brother because he had a partner who supposed to encourage him, my partner is my friend is it. Actually it is not easy to an outsider to motivate him for circumcision it is easy to a person that you had a relationship I can say my friend can you go and circumcise.

RA: What might be the reasons for you to have difficulties to encourage your brother for circumcision because he’s your blood, what can be difficult for you to encourage your brother for circumcision?

P: My brother will ask me if I am circumcised how I knew because it’s a man’s thing because they take it as a secret you see, and circumcision is not a secret because you can say my brother go and circumcise. But when you tell your brother strait in the eyes he will not agree on that he will say how you saw that I am not circumcised and you you didn’t see him, you don’t bath with him so it is easy to your partner because you sleep together and bath together you see.

RA: Why men think circumcision is a secret?

P: I don’t know because if you are circumcised it’s a good thing because if you are not circumcised they will tease you and say you are not circumcised you are a boy. Actually circumcision we were not supposed to hide is something that we should talk about it that circumcision is very important to all men, and that there won’t be able to differentiate that so and so circumcised and so and so is not circumcised and so and so is circumcised that is to create division amongst people and friends, let say my friend had boyfriend and her friends, my friend I am circumcised you are not circumcised even today, they supposed to motivate one another to say my friend go and circumcise because I already did this like this and this..

RA: If a man comes to you neh and speak about circumcision you see, how would you show him that this is beautiful and very interesting topic

P: I can say it is very important because at the end we both get helped, both you are free we don’t have any diseases.

RA: Maybe is there anything that you want to say that you feel it is very important about circumcision or about the whole interview?

P: About circumcision?

RA: Yes before we go to the next act…

P: It is very important to us all as woman and men that your partner should circumcise. Because you are protected, your partner won’t have a polo neck that thing remain clean all the time but when he’s uncircumcised he’s thing becomes dirt and he’s not free he supposed to be free when he’s with other people and he becomes man amongst people by just doing circumcision you become free like any other man.

RA: You said it is very important because you will not have the polo neck how men feel when you use the word polo neck?

P: They feel bad not a single one who likes the polo neck when it’s hot you wear a polo neck even if it is cold you wear a polo neck , you wear the polo neck only once when it’s cold only. So when it’s hot you don’t need a polo neck you wear a vest. When you remove the foreskin it’s like you wear a vest in winter and summer there is no month to wear a polo neck next month you wear a vest it’s the same thing the whole of your life

RA: Don’t you think you undermine him when you say he’s having a polo neck?

P: You do undermine him definitely he can you can also see that you do undermine him when you says he’s got a polo neck like it doesn’t end to the two of you he will think you told other people that he’s got a polo neck and he will say don’t say this thing that I have a polo neck it should remain to the two of us then you ask him when are you going to remove it, because you have to remove it so that you can free without polo neck amongst people whether its summer or winter you have to wear a vest like other people and be free

RA: You said they can see that you are a man when you remove the polo neck so if the polo neck is still there it means you are not a man?

P: You become embarrassed if you don’t remove the polo neck as a man you become embarrassed like in work in environment they undress and take the shower and bath you see so them they cover their front when a person is having a polo neck you can see by that by covering their front men and when they are together they laugh one another why can’t you remove the foreskin you are very old you see it is very important that you remove whether you are young or old it’s very important to remove it

RA: So if you can motivate a person for circumcision between rural circumcision as you said the mountain one or the clinic which one would you prefer?

P: I will encourage to the clinic one yeah. I think the clinic is very safe because at the clinic you can get the pills and the injection; they clean you there at the clinic

RA: Okay thank you very much we are going to the next activity maybe before we go to the next activity is there anything that you want to say?

P: It is very important that men should circumcise so that they can be able to protect us women. It’s us as women who have a big challenge when the men are not circumcised we get diseases like cancer, sexually transmitted diseases those are there killer diseases to women
