# Supplementary material for: “To speak or not to speak”: A qualitative analysis on the attitude and willingness of women to start conversations about voluntary medical male circumcision with their partners in a peri-urban area, South Africa
Source: PLoS One. 2019 Jan 25;14(1):e0210480. doi: 10.1371/journal.pone.0210480 (PMC6347244; doi:10.1371/journal.pone.0210480)
Supplement: S1 File — (ZIP) [file pone.0210480.s003.zip › QF027_QC2.docx]

PARTICIPANT ID (P): QF027

RA: Will you allow me to audio record this interview?

P: yes I allow you to audio record the interview.

RA: ok. So, where, where are you from?

P: Am from {} (participant address).

RA: {} (participant address). So you, you, how far is that from the clinic?

P: mmm, less than… how far, 5, 5 minutes like…

RA: 5 minutes’ drive?

P: yah

RA: ok. Oh ok, and then eee, did you know that there was a male clinic here at {} (clinic address)?

P: yes I knew.

RA: ahahah, so what is it that you knew about this clinic?

P: I only knew that the man circumcision clinic

RA: mmm

P: yah, I found out when a brother of mine came to do the circumcision and it was this year

RA: mmm

P: yah

RA: oh ok, speaking of circumcision, what is it that you knew about circumcision? What is circumcision?

P: what is circumcision?

RA: if you were tell me who didn’t know what circumcision is, what would you describe it as?

P: of the cutting of the foreskin the male eee, (laughing) the male penis if I can say it.

RA: mmm. Oh ok. Ok. And eee, what are the types of circumcision that you know?

P: I know the one that they do at the clinic and the one that they do in the mountain.

RA: mmm. Can you kindly share for me the difference between the two, the clinics and the mountain, what is your understanding of the two?

P: my understanding is that the clinic one is good and is healthy but for the one that they do in the mountain eee, I think is not healthy because I have heard several number of people eee, dying from the circumcision.

RA: mmm

P: mmm

RA: Do you perhaps have any knowledge of what happens here at the clinic and what happens at the mountain?

P: nope, I have got no knowledge

RA: as far as both is concerned?

P: no

RA: have you ever asked your brothers concerning, when he came here?

P: I have tried but most of the people don’t like sharing.

RA: mmm

P: mmm

RA: so what has been your experience with talking to males about circumcision? What has been your experience?

P: they were quit shy talking about it, being open, and they feel it’s one thing, they won’t tell you exactly what happens.

RA: mmm

P: yah. They shy.

RA: do you perhaps know or have any thoughts maybe why they shy to share?

P: no I don’t know.

RA: but in your thinking, why do you think eee, they will be you know to share, and talk about it?

P: I think is a private matter. Because is a private, is personal.

RA: mmm

P: yah

RA: ok. Ok. So, as far as you are concerned, have you ever tried to bring up the issue of circumcision with probably a male figure in your live probably a brother or partner?

P: yes I have.

RA: mmm. And how was that experience for you?

P: it was ok, it was ok I think I approached him well, he understood, eee, though he didn’t agree at first but then after a while he just went to do the circumcision.

RA: so only you talked to him regarding circumcision?

P: my partner?

RA: mmm.

P: yah

RA: so how was his reaction at first what, what, what did he say and how was the reception that he gave you?

P: he was ok to me, the only think he was scared was that he had to go do the HIV test, that’s what scared him but after doing it the test he was ok. He went for the circumcision.

RA: mmm

P: yah

RA: and so, what approached do you use as far as bringing the subject, what, what did you do like, how was the preparations like, how did you plan?

P: not many things, I didn’t plan it I just told him you should go for circumcision, to avoid, to protect yourself and me of course, to avoid our STI’s, yah.

RA: so what gave you the idea to actually suggest this thing to your partner?

P: because I didn’t like the men with the foreskin

RA: mmm

P: that’s why I suggested it.

RA: so when you brought it up did you mention that part, or how did you go about it

P: no I didn’t mention it, I just told him the, the, the risk of having the foreskin

RA: mmm. So, as a female who have experience of bringing up the subject of circumcision, eee, what do you think is the best approach women that should use as far as talking to their partners in regards to circumcision?

P: people are different so there isn’t any best eee, approach because people are different , it depend on who you are approaching and how you the person, yah, people are different so there is no better, there is no best approach is about how, who the person is. Because they can’t resume the approach in the same way whether is the best approach or not, so the isn’t a best approach

RA: ok, but you are good with your tactic, what was your tactic as far as your approach?

P: aaa, you don’t have to make them feel less of a man, not respected eee, you need talk to them in a calm way, you need to make them understand.

RA: mmm. Ok. So now what is the approach that a woman should never use in trying to now bring a subject of circumcision to a man?

P: making them feel less of a man, and they not man enough, yah, just don’t make them feel they less of a man if they are not circumcised.

RA: so which, which, how would one make a man feel like less as a man like an example?

P: by insulting them, shouting him, or maybe reminding him every day that his not circumcised or whenever you having an argument, you need to talk to him when you both calm, not when you are fight.

RA: ok. And then do you think circumcision is a good idea?

P: yah I think so

RA: and why do think circumcision is a good idea?

P: eee, it prevents male and their partners from transmitting STI’s; it lowers the risks of contracting HIV, yah.

RA: ok. And now as far as being a couple, what are the benefits of a couple now that the partner is circumcised? Any benefits that you can think of?

P: any benefits, aaa, no.

RA: sure? So they are no benefits as far as eee, where in a relationship when a man is circumcised? In your experience?

P: aaa, I don’t know I mean.

RA: ok. So now, in, in, let’s think about it in relationship, in a relationship who should take the responsibility of eee, bringing up the subject of circumcision?

P: who should take the responsibility?

RA: yah

P: aaa, anyone who feels uncomfortable with the male not being circumcised, if it’s a women then she should take the responsibility, if it’s a man then he should take the responsibility of bringing help.

RA: mmm.

P: so is any of the partners who feels uncomfortable and is their responsibility of bringing help.

RA: Ok. And, and, what do you think would make a partner be eee, feel like ok no for me *haa* no, no is not right for me, vice versa what would make them uncomfortable about it?

P: I don’t know.

RA: as far as your experiences with this concerned what made you uncomfortable now having to address this issue with my partner?

P: what made me uncomfortable?

RA: mmm

P: (laughing). I don’t know

RA: ok. So now if your partner were to approach you and had said to you ok, I have taken a decision to circumcise, how would your opinion of him change?

P: his, his taking responsibility for himself and my health, yah that’s what I would think.

RA: would you view it in a favourable manner or in less favourable manner?

P: would I?

RA: would you view him more favourable or less favourable?

P: more favourable.

RA: mmm

P: more favourable

RA: because? Why would you view him more favourable?

P: because he has the courage to, to, to go and get circumcised so that he can protect me and himself, yah.

RA: ok. So and then eee, and then for, for, for have you ever tried to tell anybody else besides your partner as far as encouraging them get circumcised?

P: nope

RA: there is nobody else?

P: no

RA: ok. Is there anything that you feel you would like to tell me that I haven’t asked or you feel like look I just want to let you guys know as far as the topic of circumcision is concerned?

P: not anymore am good.
