# Supplementary material for: “To speak or not to speak”: A qualitative analysis on the attitude and willingness of women to start conversations about voluntary medical male circumcision with their partners in a peri-urban area, South Africa
Source: PLoS One. 2019 Jan 25;14(1):e0210480. doi: 10.1371/journal.pone.0210480 (PMC6347244; doi:10.1371/journal.pone.0210480)
Supplement: S1 File — (ZIP) [file pone.0210480.s003.zip › QF028_QC2.docx]

PARTICIPANT ID (P) QF 028

R: so as I, I, we discussed that we gonna request you to, to audio record the whole session, do you still agree that we can audio record the whole session?

P: I agree

RA: ok, and then we gonna start off by me asking you some question around circumcision and we will move on to the cards and the marketing messages ok.

P: ok.

RA: so, whatever answer there is no right or wrong answer, is just whatever you believe in, ok. But can you tell me a bit about you?

P: ok, am out going

RA: mmm

P: one person who doesn’t give up easily

RA: ok

P: eee, always push for something that I want

RA: mmm

P: yah and hard worker.

RA: oh ok, ok, ok. Can you tell me, oh ok, what do you mean by outgoing?

P: outgoing I mean like I don’t spend the whole month around {} (participant address).

RA: oh you move to….

P: yah, I love visiting places

RA: oho

P: and see what they have for me

RA: (laughing). What they have for you?

P: yah

RA: (laughing)

P: maybe I can relocate to that place if possible

RA: mmm. Oh ok, you still looking for a place where you can…

P: no, just to see if it’s nice why not?

RA: oh ok.

P: mmm

RA: and you don’t give up easily?

P: no. I push until I get what I want

RA: mmm

P: mmm

RA: mmm. Ok. Currently where are you staying?

P: {} (participant address).

RA: oh in {} (participant address)?

P: mmm

RA: ok, did you grew up there?

P: mmm, I grew up here

RA: mmm. How was it growing up in {} (participant address)?

P: it was fun

RA: mmm

P: nice, challenging…

RA: challenging?

P: mmm

RA: ok, how so?

P: you know when you grew up in {} (participant address) you need to know what is right what is wrong

RA: mmm

P: especially when you go to school, you come across challenges like smoking, drinking…

RA: oh ok

P: but I decided not to join that group of people

RA: mmm

P: mmm

RA: so you have come across those kinds of challenges?

P: yes

RA: mmm. Ok. But didn’t you know about the clinic here in {} (clinic name)?

P: yah, I knew about it

RA: have you ever used it before?

P: no

RA: oh you have never?

P: you mean the clinic as a whole or this site?

RA: which site?

P: the, the, the, arm or {} (NGO name) site?

RA: oh ok, any site.

P: no the clinic yah ones…

RA: yah or the {} (NGO name) site?

P: no

RA: you didn’t know about it?

P: I knew about it

RA: mmm

P: but I was thinking is for male…

RA: (laughing)

P: …actually because its written male clinic

RA: oho ok

P: mmm and then underneath that’s where they have written HIV…

RA: mmm

P: eee, TB and whatever but the heading it says male clinic

RA: Oh ok.

P: mmm

RA: oh ok, ok. But, from that did you gather that it’s for circumcision as well?

P: mmm

RA: oh you that as well?

P: yah

RA: mmm. But what do you understand by circumcision? Just hearing the word circumcision what comes to your mind, what do you understand about it?

P: what I understand is, according to my understanding…

RA: yah

P: each and every man has to be circumcised

RA: mmm

P: cause when we grew up young boys used to go for circumcision but nowadays you come across this man who didn’t circumcise, I don’t know how but it happens.

RA: (laughing),ok.

P: mmm

RA: back in the days you saw young boys?

P: mmm

RA: and you saying…

P: … we knew that maybe at the age of seven or eight…

RA: yah

P: they have to go

RA: mmm. Ok.

P: yah. Even though they didn’t explain what was happening there but we knew that they will have to go there.

RA: mmm

P: mmm

RA: ok. When you say they, who do, who are you referring too? When you say they didn’t explain.

P: the adults

RA: oh the…

P: …actually the parents

RA: oh the parents?

P: mmm

RA: oh ok, so, ok, so you saying every man should go through circumcision?

P: yah

RA: Ok and then you saying back in the days you saw young boys doing it?

P: mmm. Almost each and every boy.

RA: oh ok, so does age play a role in circumcision according to you?

P: yah I think so.

RA: yah

P: if I had a boy child I would take that child to, for circumcision as early as three or two years

RA: ok, ok, ok its, its sounds like you believe so much that it has to be done in an early age, is it any different if aaa, is done in later age?

P: yah

RA: yah

P: if the boy is still young

RA: mmm

P: I think the pain is bearable according to my understanding

RA: ok

P: but when they grow up *eish* I think is bad

RA: you think is bed?

P: mmm

RA: oh Ok. So there is pain in circumcision?

P: yah there is.

RA: (laughing). Ok, how did you know this, because you saying you don’t have a baby boy?

P: yah my partner went for circumcision not so long

RA: oh ok

P: so I have noticed that his in pain

RA: oh ok.

P: mmm

RA: so your partner went not so long?

P: mmm

RA: mmm, ok. Did you ever talk to him about it before he went or circumcision?

P: yah, actually am the one who pushed for this to happen.

RA: you the one who pushed?

P: yah

RA: ok

P: otherwise he wouldn’t came for circumcision

RA: ok, ok. What do you say pushed, what do you mean pushed?

P: ok

RA: yah

P: I forced him

RA: (laughing)

P: to do that

RA: how so?

P: but it took me like four years to convince him

RA: mmm

P: mmm

RA: ok, can you tell me a bit more about that just the experience of telling him about circumcision, how he reacted, what changed for him to come eee, can you tell me a bit about that?

P: ok, I spoke about it, why did… ok at first when we met I asked why were you not circumcised and then he said in their culture the zulu culture actually they don’t do that.

RA: yah

P: yah

RA: mmm

P: so ok, culture aside now you grew up, we have been dating,

RA: mmm

P: so it means your ladies were not telling you that this is wrong

RA: (laughing)

P: and then he said didn’t have a problem with it and then I told him I have got a problem with it.

RA: ok.

P: yah

RA: mmm

P: so it went for like three, four years

RA: mmm

P: but the problem started when he gets the cracks or something after having sex

RA: oh ok.

P: he said it was painful and whatsoever and then I came back and said if circumcise, you cut that thing off

RA: yah

P: those cracks

RA: mmm

P: won’t be there again

RA: oh ok.

P: I even promised I can even pay you the doctor if you don’t want to the clinic

RA: ok

P: because he thought it was like everyone would know what it is.

RA: mmm

P: I offered to pay but he refused until dis year 2014

RA: mmm

P: he decided to come

RA: ok, what changed? Was it only the cuts that made him to start thinking?

P: yah I think is the cuts

RA: oho

P: mmm. Cause if it’s, if the cuts appears it means his gonna be like over seven days or more before having sex again.

RA: oh ok

P: yah

RA: it took…

P: he has to heal first. So (laughing)

RA: (laughing), oh ok. So that was the problem?

P: yah

RA: mmm. But why did you feel a need that he real needs to circumcise, like *yoh* is so important to you that your man gets circumcised.

P: no we watch TV everyday

RA: yah

P: and they say it can eliminate the STD’s

RA: oh ok

P: yah

RA: mmm. How so, do you know, have any idea how so?

P: yah

RA: yah?

P: that foreskin can put, I don’t know, they can accumulate dirt

RA: mmm

P: whatever soup that you use to bath with

RA: yah

P: you not sure if you going to clean it all

RA: mmm

P: it might stay there and cause an infection and might infect me as well

RA: mmm

P: mmm

RA: mmm. Oh ok. So he said in his culture they don’t circumcise at all?

P: yes

RA: but he still chose to come and circumcise?

P: mmm

RA: ok, what changed for him to go from, going against his culture and deciding that am gonna go except the cuts is there anything else that you feel maybe also made him to decide?

P: yes. I, I, I, asked him to choose between his culture of his

RA: yah

P: and making me happy

RA: oh ok.

P: if am not happy nothing is gonna be fine

RA: ok, what were you not happy about?

P: the, the sexual relationship

RA: mmm

P: it was not so good

RA: ok

P: yah

RA: when you say not so good, what do you mean?

P: (laugh), yoh when I say not so good because like we will only sex after few days

RA: mmm

P: am not saying we should have sex everyday

RA: oh ok I understand because of the cuts…

P: … the cuts he has to heal

RA: oho

P: mmm

RA: oh ok. Ok. So but ok, according to you who do you feel eee, should be the first person to raise the topic about circumcision in a relationship? Since in your relationship you are the one who, who started talking about it but generally who do you think should be the one who is supposed to start?

P: yah women should start this

RA: mmm

P: because they can’t start the topic they grew up knowing that they didn’t do this thing, so if you don’t raise it or question it

RA: yah

P: nothing is gonna happen they will think everything is fine and is normal, of which I feel is not normal at all.

RA: yah

P: or if it was possible a new born baby…

RA: mmm

P: must be circumcised before coming back home

RA: mmm, ok. So if women should raise this because man just think *aah* is ok even if I, I, have the foreskin.

P: mmm

RA: mmm. Ok, but would it be any different if a man was the one who raised the topic?

P: ok, if he raised the topic now at the age of 30, why didn’t he do it long time ago or he didn’t see it necessary?

RA: ok, so would it be any different if, let say it was him who came to you and said am thinking of doing this, would it have been different?

P: yah, very.

RA: yah

P: because I will be surprised why now, what happened.

RA: mmm

P: it means maybe he went somewhere, somewhere I mean cheating somewhere, and they told him about that thing and it was embarrassing for him so he decided to take it off.

RA: ok, so to you it would mean that he cheated on you and somebody told him

P: yah, that is not on

RA: oh ok. But how would you take him as a man if he was to say aaa, mentioned to you that am thinking of going for circumcision? Would that change your view of him as a man?

P: no it wouldn’t, it will be a good thing actually a good start

RA: mmm

P: mmm

RA: oh ok. But do you know of any kinds of circumcisions?

P: kinds of circumcision?

RA: mmm

P: I don’t get your question

RA: how many kinds of circumcisions do you know?

P: apart from clinic and thee… whatever they do in the villages? Or you mean how they cut it cause I have seen somewhere in the pamphlet that they are different kinds and I didn’t know, so I was surprised why is not the same.

RA: ok either one of those, whatever you feel am asking you which one do you choose or do you wanna talk about them both of them? Ok let’s start with, let’s start, you said the one at the clinic and back home…

P: where they do it somewhere we don’t know

RA: somewhere you don’t know?

P: yah, somewhere in the veld (laughing).

RA: oh ok

P: yah

RA: they do it somewhere in the veld

P: yah, of which is risky

RA: yah

P: mmm

RA: how so? How is it risky?

P: first thing first

RA: yah

P: you don’t know if they use one blade

RA: mmm

P: mmm

RA: mmm

P: I don’t think they have got like surgical spirit or something to wash the wound with

RA: mmm, oh ok.

P: yah

RA: mmm

P: and those people they have to bear that pain until…

RA: mmm

P: yah

RA: but are they any similarities between the one at the clinic and the one at the villages?

P: I don’t know what you mean

RA: are the any similarities, where you feel ok they do this the same way or?

P: no they don’t look the same (laughing)

RA: they don’t look the same?

P: no. you mean the, after taking off the foreskin or what?

RA: no I mean the whole, it can be the whole process, it can be after taking the…

P: no I won’t encourage young boys to go there, I can encourage them to go to the clinic

RA: yah

P: get injected if they need an injection, take medication, clean the wound and then yah

RA: ok, when you said they look different what were you talking about?

P: the appearance

RA: the appearance of eee…

P: the appearance of the penis (laughing), it doesn’t look the same, I don’t know how they cut it

RA: yah

P: yah oh ok. Or are they centimeters or I don’t know, they just cut

RA: (laughing). Ok, they don’t look the same, that’s the difference that you have noticed?

P: mmm

RA: ok. In the actual procedure of the two types of circumcision are they any difference?

P: I haven’t noticed

RA: you haven’t noticed any differences? Mmm ok. Ok but then how do you think eee, a woman should encourage a man to go and do circumcision? What ways do you feel a woman should encourage a man inn?

P: first you need to if, if his your partner you need to down with him

RA: yah

P: show him why you want him to get circumcise

RA: mmm

P: first you tell him about STD risks

RA: ok

P: the eee, satisfaction

RA: (laughing) satisfaction of what?

P: (laughing) is not the same the person who circumcise and the one who is not

RA: ok

P: yah. It doesn’t feel the same

RA: ok

P: especially if you have been with a partner who has circumcise and then now this one

RA: mmm, oh ok.

P: mmm

RA: ok, it doesn’t feel the same?

P: mmm

RA: ok, what else would you tell him about it?

P: and then you encourage him to go and do this because all man should circumcise

RA: ok

P: so, you don’t have to (how can I put it)….

RA: mmm

P: you don’t have to make him feel that his not a man enough because he didn’t circumcise, no I love you but you need to do this

RA: yah

P: for the sake of our relationship actually the sexual relationship

RA: mmm

P: mmm

RA: oh ok

P: and you don’t have to threaten to leave him because of that no.

RA: ok, that some of the ways a women should avoid when telling a man about circumcision?

P: yah never threaten to leave

RA: mmm. When you say a real man what do you mean? When you say don’t have to make him feel like his not a real man…

P: yah like his not a man enough, you tell him yes can do this but if you can circumcise I think the sex will be more enjoyable than before

RA: ok

P: and if he asks me, how do you know that this is better than the other one? Obviously he knows I have been with other man…

RA: yah

P: mmm

RA: ok

P: and we don’t have to push it that far

RA: mmm (laughing) ok. But is he, is your partner the only man that you ever spoke to about circumcision?

P: *heeeea* (no)

RA: yah

P: I speak to my younger brothers they came here also, I had to push them to come cause I knew that they didn’t circumcise

RA: (laughing)

P: I grew up with them

RA: mmm. Yah.

P: mmm

RA: so how was the experience different from aaa, telling your partner and telling your younger brothers?

P: with the younger brothers it’s, to me it’s easier cause I talk to the about everything and everything

RA: mmm

P: yah

RA: it was easier for you?

P: for me, but to them to wasn’t easy because they never thought I can raise that question

RA: mmm ok. So *bona* how did you tell them? Was it any different from how you talked to your partner about circumcision?

P: no, with your partner is sensitive cause it will be like oho it means now she found somebody who is circumcise

RA: mmm

P: so with the brother, you just ask them did you circumcise or what? And then it’s either yes or no

RA: mmm

P: and then when they say yes I will ask them when,

RA: (laughing)

P: because I don’t remember even one day

RA: yah

P: I have been with them my whole life.

RA: mmm

P: mmm

RA: ok, ok. So but what do you think are the benefits of circumcision to a couple, people who are in a relationship?

P: relationships?

RA: yah. How does circumcision benefit the relationship?

P: yah actually at the end of the day after having sex we have to satisfied both of us not him

RA: yah (laughing)

P: me else well

RA: mmm

P: so if am not satisfied is a problem, cause I can go out there and look for somebody who can give me what he cant.

RA: oho

P: mmm

RA: ok

P: but that’s not the aim

RA: mmm

P: mmm

RA: oh ok. So the satisfaction part you feel is the most important benefit in a relationship?

P: mmm

RA: ok. So but generally do you think circumcision is a good idea?

P: is a very good idea

RA: mmm

P: mmm

RA: what do you say so?

P: like I said before…

RA: yah

P: if you not circumcised you have a high risk of infecting other people with STD’s especially if you have got multiple partners.

RA: mmm, oh ok

P: you need to wear a condom regardless of whether you trust that person or whatever, if you don’t know the status of that person is a risk, even if you know…

RA: yah

P: anything is possible

RA: ok. So mostly the, the STI reduction, risk reduction?

P: mmm

RA: ok. But do you think culture plays a role in a man deciding to come for circumcision?

P: back there it used to play a role but nowadays is no longer about the culture

RA: ok

P: because back then we used to listen to eee, what can I say, whatever the adults were telling us, don’t do this, don’t do this but nowadays because they don’t listen to what the parents are saying …

RA: mmm

P: they just do things on their own

RA: mmm

P: that’s why according to my understanding, not everyone, according to my understanding that’s why the, the HIV infected or affected people…

RA: yah

P: is way too much compared to back in the days, am not saying HIV was not there back then, it was there…

RA: mmm

P: but because they respected the, I don’t know whether I can say the culture or…

RA: yah

P: nowadays we just go there, we have sex with whomever without even knowing what’s happening with that person.

RA: mmm

P: so

RA: ok.

P: if you circumcised and you wear a condom, life goes on

RA: ok. So you still need to wear a condom even if you circumcised?

P: yah, you still have to.

RA: ok. But what do you think makes it easier for other cultures to accept circumcision so easily and other is a bit difficult?

P: ok, in my culture is up to you actually, in my culture where I come from, it’s up to you whether you go for circumcision or not.

RA: mmm

P: but mostly when they grow up they will, they will go for circumcision at the hospital

RA: mmm

P: but with this zulu culture is like you don’t go there at all

RA: mmm

P: and there is no valid reason why you shouldn’t do this circumcision.

RA: so he never gave you reasons why he didn’t?

P: no he said culture and then I said now we put the culture aside

RA: oh ok. And your culture is?

P: Tsonga.

RA: oh ok, in Tsonga culture is your choice?

P: actually if your parents don’t want you to go at the younger age, you can go when you are older

RA: mmm

P: is up to you

RA: mmm

P: mmm

RA: so ok, so you also feel that parents also play a role in deciding whether somebody should go and circumcised or not?

P: mmm, cause if the parents say no, you can’t go on your own

RA: ok

P: mmm

RA: (laughing), reason being?

P: what if they disown you, you know this old people

RA: (laughing)

P: and is not something that you can just go and do it, they will see that something is wrong with you

RA: mmm

P: mmm. Cause you will be sick, like you will feel very sick. You won’t just walk around as a normal person

RA: oh ok

P: cause is painful (laughing)

RA: oh that’s the experience that you have?

P: yah, I have seen that the men is in pain.

RA: mmm

P: mmm

RA: ok, was it that severe? Like how you say it is like it was so painful

P: it was painful but the thing is men are, I don’t know if I can say they are week or what, we women can bear the pain but they can’t.

RA: oh ok, you think maybe is because he couldn’t bear the pain?

P: yah, but only for few days not like the whole month

RA: it was only for few days?

P: mmm

RA: so how was that experience for you seeing your partner in pain cause of something you suggested, forced him to do it?

P: (laughing), I wasn’t happy, I shouldn’t have pushed him

RA: oh ok

P: but then I said no he will be fine

RA: mmm

P: mmm

RA: mmm ok. Well eee… but then I think we have come to the end of first eee discussion, unless is there anything else that you feel we didn’t talk about?

P: no we did
