# Supplementary material for: “To speak or not to speak”: A qualitative analysis on the attitude and willingness of women to start conversations about voluntary medical male circumcision with their partners in a peri-urban area, South Africa
Source: PLoS One. 2019 Jan 25;14(1):e0210480. doi: 10.1371/journal.pone.0210480 (PMC6347244; doi:10.1371/journal.pone.0210480)
Supplement: S1 File — (ZIP) [file pone.0210480.s003.zip › QF030_QC2.docx]

**PARTICIPANT ID:** QF30

**FACILITATOR:** As indicated we are going to record the conversation as we talk, are you agreeing that we record the conversation?

**FACILITATOR:** Okay. I said that today we are going to do three things. Firstly, I am going to ask you some questions about circumcision. There are no wrong or right answers. You answer according to your understanding. But then before we start, what can you tell me about you?

**PARTICIPANT:** About me?

**FACILITATOR:** Yes, what can you tell me about you? Just briefly about you.

**PARTICIPANT:** You want me to just tell you about me?

**FACILITATOR:** Yes.

**PARTICIPANT:** Okay. First, my desire is to have a good family. I wish to at least get a job; and see myself driving a second-hand car. Have the right family. I wish God can help me live a better life; so that I could assist people.

**FACILITATOR:** Assist other people?

**PARTICIPANT:** Assist other people? I am not such kind of people who go to taverns. Since I was born, I have never tasted beer. I was raised by both parents. That’s why I grew up with respect. I had a child at the age of 26, because my dream was to achieve other things prior to falling pregnant and having a child. I didn't like roaming the streets not knowing what to do. I wasn't such kind of people who like going up and down. I preferred staying in one place at a time. And being a straight-forward person. And also relating well with other people.

I like respecting older people so that I could be showered with rewards in abundance, stroke of luck and blessings in return.

**FACILITATOR:** When you say the right family what do you mean?

**PARTICIPANT:** Just like the way I grew up. The manner in which my parents raised me. I didn't grow up the way today's youth are being raised. When I say I need the right family, I mean having my own household; having two or three children of my own; and that the man I live with should be a man amongst men. Today’s men are men by title only. You live together with a man and assist him with everything; when realising that all his problems are sorted out, he abandons you, because he is now seeing other pretty women with nice-looking bodies with bright colours, although their judgment is far less than yours.

**FACILITATOR:** What is a man amongst men?

**PARTICIPANT:** He is someone who knows that he has a wife, he has children and one who knows that every day when he goes to work he always remember that he has left behind a wife and children. Let’s say when both of us are employed, and when we receive our salaries, we put our salaries together and agree on how to spend it.

**FACILITATOR:** As we speak now, is there such a man in your life?

**PARTICIPANT:** There is no such a man these days as I have already mentioned. Today’s men are dangerous. They are running after hair-styled women, and show no interest in those of us who cover our heads with something. Besides running after hair-styled women, they also look for somebody with a nice shape and a bright colour. Those of us who are black in complexion are in trouble. There are women out there who are bright in complexion, with curves, and look very nice. Although they look very nice, their reasoning capacity is far less than mine and we do not think the same.

**FACILITATOR:** When you say reasoning capacity what do you mean?

**PARTICIPANT:** Most girls particularly those who are frequent guests at the taverns, just tell themselves that they are going to wear in a particular fashion, and like it or not they are going to get a beer even if they don’t have money. When Friday comes they get excited, but on Mondays to Thursdays, it is the opposite. When it is Friday, they get excited because they are going to trick poor men to buy them beer, I am said men sometimes are useless. Out of 100% men, maybe only 20% of them are wise enough these nowadays. Even married men who have rings on their fingers are still going up and down. No one knows what are they looking for. It is something that we always see. And we girls don’t think the same. There are those who only think about taverns and men, instead of thinking about preparing for their own future such as: looking for a job, have her own home, buy herself a car and assist her parents.

**FACILITATOR:** You said that you were raised by two parents, neh?

**PARTICIPANT:** Yes.

**FACILITATOR:** How many were you in the family?

**PARTICIPANT:** We are eight and are staying together. And it was nice.

**FACILITATOR:** How many were you? How many boys ... [interjection]

**PARTICIPANT:** We are five girls and three boys. At the moment, only one girl has passed on, the first born. Only seven are left, and all of us, with the exception of the last two boys, are having our own homes. They are still staying with my mother as well as the daughter of my deceased sister. They are staying with my parents.

**FACILITATOR:** But do you talk to these two boys about circumcision?

**PARTICIPANT:** Yes, they talk to them, they had been to the initiation school. At home we go to initiation schools. Women go to initiation schools and so are the men.

**FACILITATOR:** Even women go to initiation schools?

**PARTICIPANT:** Yes. I went to the initiation school; and spent the whole eight weeks there. It was in 2002. These boys went to the initiation school the year before last in 2012.

**FACILITATOR:** Okay. Is there an initiation for women, and an initiation for men?

**PARTICIPANT:** Yes.

**FACILITATOR:** How many types of initiations do you know about? Isn’t it that you have just spoken about female initiation and male initiation; are there other types of initiations you know about?

**PARTICIPANT:** No, I don’t know of any; the only initiations I heard briefly about them are that of the Vendas. It is said that the Vendas are initiated thrice. I am not sure whether they first one is called domba, then follows dombelela, and the third one I don’t know what it is called. I forgot it. I don’t know. We, the Pedis are initiated once; we don’t get initiated twice. And the Pedis are not different. We, the Pedis from Jane Furse, something is done on our backs to signal circumcision. With others (Pedi people) it is done on their faces. They make a round thing on their faces. I don’t know how other cultures are doing it.

**FACILITATOR:** What do they do on them?

**PARTICIPANT:** I mean teaching them codes of behaviour. They teach us codes of behaviour and also apply an animal skin on our shoulders to make a cross.

**FACILITATOR:** Oh, they make a cross?

**PARTICIPANT:** Yes.

**FACILITATOR:** What do they do?

**PARTICIPANT:** I don’t wanna lie. I don't know what they have used. But we are told that they are using the animal skin. Other Pedi people; you might have heard other Pedi people proclaiming that they are initiated the Sekone way and others also proclaiming their own practices. This Bakone clan are the ones who make a round on the faces of initiates. I don't know what they use. With us, something is applied on our shoulders. Men, it is said, are circumcised by being cut.

**FACILITATOR:** Oh, men are being cut?

**PARTICIPANT:** Yes, men are being cut. We, women, are not being cut.

**FACILITATOR:** What do they do on you … [interjection]

**PARTICIPANT:** They just do what I mentioned earlier. In actual fact, I wasn't aware. When I went to the initiation school, I realised the importance of undergoing such initiation, but at a later stage I then discovered that there is no need for women to be initiated. Why should we women be initiated, if nothing is done on us? Men are better off, because they get help.

**FACILITATOR:** You said earlier that the Venda are initiated three times and that the men are being cut … [interjection]

**PARTICIPANT:** Seemingly men don't get initiated three times. It is only women get initiated three times. It appears that women are the ones who get initiated three times. That’s why I said I am not sure. I overheard them talking.

**FACILITATOR:** But what do you think makes these initiation rituals differ according to cultures instead of being practised the same. Why is it practised differently according to cultures?

**PARTICIPANT:** It is because we are also different. Our cultures are different. It is the same as surnames. Can you see how different are our surnames? The same applies to the initiation rituals and cultures thereof. Cultures are not the same. As indicated earlier, even the Pedi people don't speak the same Sepedi language. Some refer to porridge as 'bogobe' and others refer the same as 'booswa'. Have you taken note of the dialect spoken by the Balobedu people? They refer to porridge as 'booswa'. That's the difference. We, the Pedi people also, there are those who go to the initiation school for two months or eight weeks. There are those who go for three weeks; and others go for six weeks.

**FACILITATOR:** Oh, the duration is not the same?

**PARTICIPANT:** Yes, the duration is not the same?

**FACILITATOR:** So, your younger brothers also went to the initiation school?

**PARTICIPANT:** Yes, they went to the initiation school. There is only one person at home who did not go to the initiation school. It is my deceased sister's daughter, whose mother died when she was only seven years old. She is the only one who did not go to the initiation school. But all of us have been to the initiation school.

**FACILITATOR:** Before they went, who spoke to them about initiation matters?

**PARTICIPANT:** It is my father who spoke to them. And we girls, it is my grandmother and my mother who spoke to us about going to the initiation school. It was my grandfather, my father and my uncle who spoke to my brothers. They are the ones who advised them about what happens there. It is not so long that we learnt that men are being cut at the initiation school. We learnt about it now when we were grown-ups that men are being cut/circumcised at the initiation school. We did not know. Remember, nothing is done on women. We just go there to learn codes of behaviour in life.

**FACILITATOR:** They teach you how to behave in life?

**PARTICIPANT:** Yes, they teach us such codes. They teach us how to behave just like when you get into a marriage. When you get into a marriage, elderly women would sit with you and teach you proper behaviour towards your husband.

**FACILITATOR:** Are these the codes they teach you?

**PARTICIPANT:** Yes, these are the codes they teach us? But at the initiation school, they don't teach you how the husband is supposed to be treated. They only prepare us to behave ethically when reaching a particular age; this include telling us how to speak and conduct ourselves properly, else we would not have families. If not, we would find ourselves facing divorce and ultimately starting to drink heavily. Just like now, we were raised under very strict conditions by our parents. My sister imparted some wisdom on me at a very young age. I then told myself that education comes first. Without education there is no employment; without education there is no life. She is the one who made me realise that 1+1 is 2 and not 11. When you attend school, this is what you are supposed to be doing, said my deceased sister. Our parents also used to guide us. Her second child is the one who has gone astray. My mother no longer knows what to do. She quit school. When we tell her about school, she does not listen. Today is Friday and she is going to the tavern. She is the only one who went astray. We all go to church. We are members of the ZCC church. I have not wearing my badge because my child is going through some traditional healing rituals; which is against Christian beliefs.

**FACILITATOR:** What do you mean by traditional rituals?

**PARTICIPANT:** I mean customary rituals, the Apostolic beliefs and practices. My child is going through apostolic rituals because the father follows apostolic beliefs.

**FACILITATOR:** How is the church related to issues of circumcision? Do you think there is a link between these two practices? When you say in your family all of you have been to the initiation school, was it something that was influenced by the church?

**PARTICIPANT:** The church is strongly opposed to initiation rituals. According to health regulations, they want to fuse them into one thing. This is done in order to avoid someone doing this and others doing completely different things altogether. Let’s say you believe in the existence of ancestral spirits, and I am a Christian who believes that ancestors do exist; you find that although we are born of the same parents, we have never done the same thing. It is possible that if the deceased person whose name you have assumed has been initiated and you have not, this deceased person can make your life miserable. Even at church they would tell you that your angel does not want you to do this and that. You could see that although people pretend not to believe in ancestors, in practice they follow them. A person would ask about which angel are you talking about? When they speak about angels, they actually refer to the ancestors.

**FACILITATOR:** Who are you referring to when you say the person whose name you assume after graduating from initiation? Are you given a new name that belongs to someone else?

**PARTICIPANT:** Yes. It might be our grandmothers' names. For instance, when my mother falls pregnant. She then confides to my grandmother. After my father has impregnated my mother, my grandmother proposes that when the child is born, it be named after her.

**FACILITATOR:** Oh, is that so?

**PARTICIPANT:** Yes, that is how it is. That being the case and having assumed the grandmother's name. The grandmother dies without the child knowing her or where she was buried. It is possible that when she needs something she would come to me. When she comes to me, let’s say maybe she went to church. When they prophesise, they would tell her that her angel wants one, two and three. It is that very person who suggested that the child be named after her.

**FACILITATOR:** How did you know about what is being done at the men's initiation school? Isn't it that you said that you did not know what is being done there. You only became aware now. How did you know?

**PARTICIPANT:** We knew about these when we started participating in this research study at clinics. We accompany people to the clinics. My first visit at the clinic, was when I accompanied my deceased sister, who is the first born at home. I was charged with the responsibility of accompanying her to the clinic. I was taking here there and bringing her back home, because she was unable to do anything on her own. I listened attentively while people were talking about circumcision issues – some people don't take this matter seriously. People can't think the same. There are those who only think about going to the taverns. I resolved that I would remain in the yard … other girls can't think the same as I do; they don't have a future. She would go to the tavern; when she gets there a man lures her with beer, and thereafter have sex with her. He brings her back. He takes another girl and have sex with her. What nonsense is this? You'll become infected with the disease and not know how you contracted it, because you were drunk.

**FACILITATOR:** Oh, you first heard about circumcision at the clinic?

**PARTICIPANT:** Yes, we first heard about it while at the clinic. And often people mentioned that when men go to the initiation school they are being cut. But we did not understand what was meant when they say they are being cut. As time went by, we started understanding that when they say they are being cut, what is it that they cut them.

**FACILITATOR:** Okay, what is that you heard it is being cut?

**PARTICIPANT:** The front part of the foreskin which conceals unhygienic discharges.

**FACILITATOR:** What foreskin?

**PARTICIPANT:** What would I say? The front part of the foreskin of a penis. If I could put it in that way. It is that part of the penis which swara the diseases.

**FACILITATOR:** It conceals the diseases?

**PARTICIPANT:** And it is not long that we discovered about this; we were told what circumcision is and how it is done. Then we started realising that when people get sick it is mainly because of the diseases transmitted during sexual intercourse from the male partner. Particularly from those who drink beer. They have sex with men without a condom. And the man is not circumcised. Or he has never been to the circumcision school. Are they not going to infect each other? Surely, they are.

**FACILITATOR:** This foreskin conceals unhygienic discharges and diseases?

**PARTICIPANT:** Yes. Because when two different sperms come into contact, they result into a disease.

**FACILITATOR:** When you say two, who are you referring to?

**PARTICIPANT:** Like two men. For instance, I have sex with you, and then suck ditšhila from you. I then go and have sex with somebody else. Can you see what those sperms are going to do?

**FACILITATOR:** So when you came to the clinic, they told you about traditional initiation school?

**PARTICIPANT:** They are almost the same. In actual fact they are the same. It’s just that we don't know what instrument is being used at the clinic to cut/circumcise, at the initiation school they use one segment of a pair of scissors. At the clinic they use an injection. At the initiation school they don't use any injection. They use some traditional medicine.

**FACILITATOR:** What do they use it for?

**PARTICIPANT:** After removing the foreskin, they smear the medicine over the cut area. At the clinic they use the injection. After the injection you use low salty water.

**FACILITATOR:** Is that how they differ?

**PARTICIPANT:** Yes, that is how they differ?

**FACILITATOR:** Is there anything else that differentiates them?

**PARTICIPANT:** There is nothing. The other difference is that stitches are used at the clinics, and at the initiation school they don't do stitches, but apply the indigenous medicine over the cut area. At the clinic they make use of stitches and you wait until they come out on their own while continuing with the salty water treatment.

**FACILITATOR:** Have you ever spoken to a man about this issue of circumcision? When I say a man, it could mean your partner, it could be a family member, and it could be a friend. Anyone who is a man.

**PARTICIPANT:** I told my friend about it; I also told my partner, I currently have an affair with, who is also the father of my child.

**FACILITATOR:** Can you briefly explain to me about this father of your child as to how did you approach him and what was his reaction, what happened when you told him.

**PARTICIPANT:** Before I could tell him, I was at a clinic at Thafene. Then came some nurses from {} (area mentioned). On arrival they asked to speak to us. They explained to us about the benefits of circumcision these nowadays – isn't it that circumcision was not practised previously. Before people became aware of this circumcision, they went through initiation schools. These nurses told us that the reason for the introduction of circumcision is because there are many diseases. They explained to us what circumcision is. They explained to us that they remove the foreskin. Circumcision protects the family and many other people. They protect them by removing the foreskin of the man which conceals unhygienic discharges, because you can have sex with someone without using a condom; and this someone in turn has unprotected sex with another one who has an STI without knowledge. After that when he comes and have sex with you, you become infected. Because I have said that the reason for circumcision is because the foreskin conceals unhygienic discharges which cause ailments on people. After realising that my partner is a Zulu speaking person and the Zulus do not go to initiation schools at all. I then resolved that I need to speak to him and ask him to think about circumcising in order to protect himself. I said to myself that I would talk to him and ask him to be circumcised. I would for him when he comes back from work. I went to the clinic and when I came back, I waited for him to come back from work. I sat down with him. I said to him: There are diseases. Aids is one of them, although Aids is no longer a killer disease. It is possible to contract diseases such as 'drop' or get sick after having sex with someone who aborted her pregnancy. I explained to him about such kind of diseases and then suggested to him about removing the foreskin, though I knew that the Zulus don't get circumcised. The reason is that I don't know whether he is having other affairs out there or not, because that's the culture of men. And I can't be his guard. I asked him to do himself a favour by doing what I was asking. Then he said, okay, what you are telling me is true. I would answer you at a later stage if I am going to do it or not. I then went to {} (area) for one week. While in {} (area), he phoned and said to me that he went to do what I had asked him to do. He realised that what I told him, is a better life. Like others, I would be able to see if my foreskin has unhygienic discharge. Before circumcision it was not possible for him to notice any unhygienic discharges, because after having sex with a woman and you wipe off your private parts, there are some unhygienic discharges that remains on the foreskin. When these unhygienic discharges gather on the foreskin, they cause diseases. He went and did it and it was a good thing to do.

**FACILITATOR:** Didn't he react in a strange way?

**PARTICIPANT:** He didn't react in a strange way. He also invited his friend, who also did it.

**FACILITATOR:** Between a couple who are in a relationship, who do you think is supposed to initiate the topic of circumcision?

**PARTICIPANT:** It is the wife because the husband thinks about himself only. He doesn't realise that if I do this, it would advantage or disadvantage me. He thinks about that moment only, and about himself only.

**FACILITATOR:** Does he take time to do self-introspection?

**PARTICIPANT:** Yes, he takes time to do self-introspection. If I was not there, he was not going to do it. He was going to say: I am a Zulu, I can't do such a thing. I can't be circumcised, because even my uncles have not been circumcised. I am a Zulu, I am not going there. At least a woman can think and then talk to you, and does not care whether you are going to react strangely or somehow or whether you think what she is going to tell you would save your life or not.

**FACILITATOR:** What do you think has changed that convinced him to ultimately go for circumcision as you have said that Zulus do not go for circumcision at all? He is a Zulu as well, but he ultimately went for it. What do you think has changed that made him do it?

**PARTICIPANT:** After talking to him, I think what made him realise that this is a good thing is because he realised that the foreskin was indeed concealing unhygienic discharges. And besides the fact that it conceals unhygienic discharges when you sleep around, the next you realise that you are infected with Aids. Aids is caused by the blood which is not clean.

**FACILITATOR:** Okay.

**PARTICIPANT:** I think he realised that after having had sex with someone else, after that when he wipes himself, some unhygienic discharges gather on the foreskin.

**FACILITATOR:** That is why you spoke to him about this issue of unhygienic discharges and diseases?

**PARTICIPANT:** Yes.

**FACILITATOR:** Are there other alternative ways through which you think a woman should talk to her husband regarding circumcision? Things which you think a woman should tell the husband when talking to him about circumcision?

**PARTICIPANT:** I think the main reason why the husband must consider circumcision is diseases.

**FACILITATOR:** It is because of diseases?

**PARTICIPANT:** Because I don't dispute the fact that he can protect himself with a condom, but as those unhygienic discharges gradually gather on the foreskin, as time goes on, he would start feeling some pain on his private parts. When he feels the pain, he would need some injection.

**FACILITATOR:** Which aspects do you think the wife should avoid when talking to the husband about circumcision? I mean the things which she is not supposed to mention?

**PARTICIPANT:** Do you mean those things which she is not supposed to talk about them?

**FACILITATOR:** Yes, when talking to the husband about circumcision?

**PARTICIPANT:** She is not supposed to tell the husband that he must be circumcised because he has sexual relationships with other women. I am not supposed to mention such things to him. I am supposed to tell him about sexually-transmitted diseases.

**FACILITATOR:** You are not supposed to tell him that he is having relationships with other women?

**PARTICIPANT:** Don't tell him that he is having other relationships, and as a result must be circumcised in order to protect himself when having sex with those women. He would go out there and sleep around with different girls. You must never mention that to him. This would result in a fight and the main reason being that you are accusing him of sleeping around with other girls. It means you are saying that your husband is ‘jollying’.

**FACILITATOR:** You are telling him of ‘jollying’ without any proof to that effect?

**PARTICIPANT:** Yes, you don't have proof that he is ‘jollying’.

**FACILITATOR:** But if he is the one who initiated this discussion of considering undertaking circumcision, do you think it would have been different for you to tell him?

**PARTICIPANT:** It was not going to be different; it is just that I was going to be surprised that this person thought about doing a nice thing. I was going to ask myself as to who advised him to do such a thing? Because Zulus are very stubborn people. They would tell you that they don't know such a thing.

**FACILITATOR:** Will you be surprised because he talks about it as a Zulu speaking person or you will be surprised because he is thinking about doing such a thing?

**PARTICIPANT:** I would be surprised as to how did this person think about this thing, because they don't get circumcised. And they would tell you that they don't do these things. I was going to ask myself how it came about because these people would tell you that I am a Zulu and I don't do this thing. I am saying so because after the father of my child did it, we told his younger brother to consider doing it as well. The younger brother replied by saying that he is not crazy and he is not going to do such a thing. He said he is not going to do it, because most people from his area don't do such a thing. When we tried to explain to him the benefits of doing such a thing, he replied by saying, no, I am living my own life. I have already said earlier that people don't think the same.

**FACILITATOR:** Are you saying it is different when it is initiated by the man than when it is initiated by the woman?

**PARTICIPANT:** Yes, it is different. A woman thinks. I say a woman thinks because women would consider a whole lot of things first. Let’s say a man is living in a rented room; he thinks about buying a bed, a stove and one or two pots and that is all. A woman, on the contrary, would also think about displaying a flower for decoration; buying a micro-oven and putting it there; at least buying a fridge; put the bed and the wardrobe in a particular style. But as long as the husband has bought two cooking pots, and has a bed; that's all.

**FACILITATOR:** What do you think are the benefits of a couple with regard to circumcision, when the husband decides to get circumcised?

**PARTICIPANT:** I beg your pardon?

**FACILITATOR:** The benefits of circumcision in a relationship? How do you think it would benefit the couple in a relationship?

**PARTICIPANT:** What would I say? Please repeat the question.

**FACILITATOR:** Where people are in a relationship, what do would you see as benefits when the husband gets circumcised?

**PARTICIPANT:** The benefit in the family for the couple when the husband goes to the initiation school is the same as when he gets circumcised, because a man is a man; you may be living with him as your husband, as I have already indicated earlier, even married husbands who have rings on their fingers go out and have sex with other women outside. If he went outside and had sex with someone else, and he was drunk and maybe the woman was also drunk, they can't remember whether they used a condom or not. I also don't know whether the woman has terminated pregnancy or not? If he is circumcised, this would save the situation.

**FACILITATOR:** Oh.

**PARTICIPANT:** It saves because if the man has slept with a woman who has aborted her pregnancy, he gets ill very fast. And let’s say maybe after getting ill you go and visit a priest. When you arrive there, you make a confession that you slept with a woman who has just done an abortion, he then prescribes you tea or something to drink to cleanse you.

**FACILITATOR:** He would be prescribed tea to drink for what?

**PARTICIPANT:** He would be prescribed tea to drink in order to cleanse himself. It is just the same as infection when you have sex with a woman who has just terminated her pregnancy. She is unhygienic, her blood is unclean. And unclean blood makes you ill. It is just like using contraceptives. Let’s say maybe you had been using contraceptives for three years and were not seeing your menstruation, that blood can make you sick, because it didn't come out. For instance, if a person, let’s say has spent six or seven years without having had sex with a woman; if you look at him, you'll realise that he appears insane. Serious, such a person would appear like someone who is insane.

**FACILITATOR:** Why?

**PARTICIPANT:** His blood is no longer circulating in his body. It is not circulating. I have seen a certain gentleman where we stay, who appears insane. He is no longer thinking properly. He is not normal. When he is supposed to go somewhere, he would take the long way instead of just passing here and going straight there. He would go and turn far away, turn at this corner, until he reaches there. Do you think this person is in a good frame of mind? No, he is insane. Instead of collecting water from a nearby tap, he would walk to the one far away in the street. He opts to walk a distance to fetch water from the tap in the street. When you ask him, he would tell you that water from the nearby tap is bewitched. Can you imagine that? The next thing he no longer makes use of the pedestrian road, but uses the road earmarked for cars. He would tell you that the pedestrian road has been bewitched. Can you imagine that? Can you see that this person is insane?

**FACILITATOR:** What do you think is the cause?

**PARTICIPANT:** I think that it is because of the blood which is not circulating, and religion beliefs also add to the causes, because you would see him go to church, the next thing he is drinking liquor while wearing the badge and the two don't go hand in glove. He has not been dating a woman or sleeping with a woman for a very long time. He would drink liquor, if not he would then go to church. How would you drink liquor with your badge on? It is unheard of.

**FACILITATOR:** You said earlier that if it was the husband who initiated the discussion about circumcision, you would be surprised?

**PARTICIPANT:** Yes, I was going to be surprised indeed.

**FACILITATOR:** Were you not going to support him and encouraged him to go?

**PARTICIPANT:** I was first going to be surprised, but I was going to tell him that what he is thinking about is a good idea. What you have thought about is a nice thing. Please go ahead and do it. If you have thought about this and have concluded about it, it is a nice thing indeed.

**FACILITATOR:** But generally do you think circumcision is a good thing? Generally?

**PARTICIPANT:** I think it is a good idea. Initiation or circumcision, as I speak now at least for now has saved many people. Some people would just indulge in liquor and get drunk; and when they are drunk they sleep around with strangers; and not knowing who is carrying what disease.

**FACILITATOR:** For how long have you been living with your partner?

**PARTICIPANT:** Three years.

**FACILITATOR:** When did you tell him about circumcision?

**PARTICIPANT:** This year. I told him this year after having a child with him.

**FACILITATOR:** Oh, you already have a child with him?

**PARTICIPANT:** Yes.

**FACILITATOR:** What made you tell him in the third year of your three-year relationship that you were suggesting that he should be circumcised?

**PARTICIPANT:** I told him in the third year, because I was still researching him to establish what kind of a person he is. I wanted to establish first if he is that kind of a person we can live together? I wanted to find out if he is that kind of a person that I can tell him such things and also to find out if I could confide to him my family problems. I realised that at least he meets some of my expectations, but out of 80%, I wouldn't say he meets 50%, but at least 30% he tries. Zulus are very different people. I then had a child, plus I had a child with him and he also had never had a child. I also did not have a child; I had my first child at the age of 26 last year.

**FACILITATOR:** Hm, first you wanted to see what type of a person he is?

**PARTICIPANT:** I first wanted to see what type of a person he is. You can research a person and find out he is sharp for three years, in the fourth year then he changes. We have had four-year relationships and then broke up; we then had three-year relationships and broke up again. If I had told him to do this, maybe even if we broke up one day when he sees me he would say that I had saved him.

**FACILITATOR:** I think we have come to the end of part one. Unless if you think there is something we have not spoken about.

**PARTICIPANT:** We have spoken about them all.
