# Supplementary material for: “To speak or not to speak”: A qualitative analysis on the attitude and willingness of women to start conversations about voluntary medical male circumcision with their partners in a peri-urban area, South Africa
Source: PLoS One. 2019 Jan 25;14(1):e0210480. doi: 10.1371/journal.pone.0210480 (PMC6347244; doi:10.1371/journal.pone.0210480)
Supplement: S1 File — (ZIP) [file pone.0210480.s003.zip › QF011_QC2.doc]

Participant ID **:** QF011

**FACILITATOR:** QF011. Do you give me permission to record this interview?

**PARTICIPANT**: Yes I agree.

**FACILITATOR:** Okay. So I would like to know more about you, where do you live and where were you originally born?

**PARTICIPANT:** I live here in {} (participant address), I grew up here.

**FACILITATOR:** You grew up here in {} (participant address)?

**PARTICIPANT**: Yes.

**FACILITATOR:** How many are you at home?

**PARTICIPANT:** From my mother we are 5.

**FACILITATOR:**	You are 5?

**PARTICIPANT:**	I am the first born.

**FACILITATOR:** Oh, you are the oldest?

**PARTICIPANT:** Yes.

**FACILITATOR:**	Okay, how many boys are there at home?

**PARTICIPANT**:	1

**FACILITATOR:** Only 1? So you are 4 girls at home? Oh okay, so how old is the boy?

**PARTICIPANT:** He is 15.

**FACILITATOR:** Okay, he is 15? Okay, alright. So is the clinic not far from where you live to here?

**PARTICIPANT:** From here...?

**FACILITATOR:** Where you live?

**PARTICIPANT:** To come here?

**FACILITATOR:**	Hm.

**PARTICIPANT:**	Aah.

**FACILITATOR:** Okay.

**PARTICIPANT:** Because I come with a taxi but it doesn't take even an hour.

**FACILITATOR:** Oh okay so did you know that here at {} (clinic address) there's a men's clinic?

**PARTICIPANT**:	Yes I knew that they do circumcision.

**FACILITATOR:** Oh okay so you knew they do circumcision here?

**PARTICIPANT:** Yes because my sibling once came here.

**FACILITATOR:** Oh your sibling was brought here?

**PARTICIPANT:** Yes.

**FACILITATOR:**	Oh okay. So would you tell me what you know or understand this thing when we talk about circumcision?

**PARTICIPANT:** I personally don't know why it's done, why boys should.... but from what I hear when you go to the clinic they explain that, it's done so that yo don't germs fast or HIV, and not to have... you like drop.... not to get it fast from the information I got.

**FACILITATOR:** Okay so what types of circumcision do you know of?

**PARTICIPANT:** I don't know, when I was growing up I knew that they go to the mountain, I didn't know that it's done at the clinic I just found out recently. When I was growing up they would go to the mountain.

**FACILITATOR:** Okay and if you could tell me what do you know about the one at the mountain?

**PARTICIPANT**:	Nothing.

**FACILITATOR:**	You know about going to the mountain only?

**PARTICIPANT:** Hm.

**FACILITATOR:**	What happens?

**PARTICIPANT**:	I don't know

**FACILITATOR:** But do you know of men who went to the mountain?

**PARTICIPANT:** Yes.

**FACILITATOR:** So when they talk...

**PARTICIPANT:** You know boys don't like to talk and more especially if it's a..... person you not used to. Yes you will talk but it doesn't cross my mind to ask what happens at the mountain.

**FACILITATOR:** So you've never heard them talking about what happens at the mountain? Nothing?

**PARTICIPANT**:	Hm Hm

**FACILITATOR:**	Oh, okay. But what do you think about circumcision, what's your view about circumcision?

**PARTICIPANT:** I've since known that if... I think it's a right thing because they say when you are circumcised it's not easy to get sick fast. So I think it's a right thing because boys these days, we as the youth of today we fast in a lot of things. So we don't think that eish, that if I do this, this will happen so at least for a boy, if it's like that to me it's a right thing.

**FACILITATOR:**	A right thing?

**PARTICIPANT**:	Yes.

**FACILITATOR:**	And then women in general, you girls, what do they say about circumcision?

**PARTICIPANT:** Hey we as girls, okay let me refer to myself and not other people. To me it's like a boy lives his own life. The thing is I don't know I've never stayed with a boy from outside and speak to him, that you boys live this life so so and so. I see them but I've never sat down with them and speak to him, why you boys live this life, you see?

**FACILITATOR:**	So eh... some you will be sitting with your friends you've never talked about circumcision and discuss it as girls alone?

**PARTICIPANT**:	No I've never.

**FACILITATOR:**	Okay. What do you think of women who talk about circumcision issues do you think it's good idea according to you?

**PARTICIPANT:** For me hey... I don't know about other, for me I don't know whether I am shy or I don't know for me hey...

**FACILITATOR:** So why do you think it's not a good idea for women to talk about circumcision issues?

**PARTICIPANT**:	I think boys staff should be discussed by them, that's how I see it.

**FACILITATOR:**	So you don't think it's an idea you would have?

**PARTICIPANT:** Huh-huh.

**FACILITATOR:** So let's say you were encouraging someone to circumcise. Which method would you use to encourage a boy?

**PARTICIPANT**:	Hey I don't know.

**FACILITATOR:**	Okay.

**PARTICIPANT:** But I would tell him to do it. If he asks me why? I would tell him you have to, because now there are lot of diseases.

**FACILITATOR:**	And then as a woman which way do you think you shouldn't use when encouraging a boy about circumcision issues? What do you think a boy wouldn't be happy about when encouraging him about circumcision issues? Maybe which approach will he not be happy about?

**PARTICIPANT**:	He will ask me why I want him to do it because I will go on my own.

**FACILITATOR:** And then you as a woman in that situation when he says hey I will go on my own, how would you handle it?

**PARTICIPANT:** If he doesn't agree I will leave him.

**FACILITATOR:** Hm

**PARTICIPANT:** Because I won't force him.

**FACILITATOR:**	So what do you think women who are trying to encourage men to circumcise should do? What must they do? What mustn't they do?

**PARTICIPANT**:	Yoh. You know I don't know.

**FACILITATOR:** Alright. Who encourage your brother at home?

**PARTICIPANT:** My mother.

**FACILITATOR:**	Your mother. What does your mother think about circumcision issues?

**PARTICIPANT**:	I don't know what to say she thinks.

**FACILITATOR:** Hm

**PARTICIPANT:** Because he is growing now and he will be busy with girls and I don't know because me and my mother don't sit down and talk about such things.

**FACILITATOR:**	Hm

**PARTICIPANT**:	But my mother spoke to him. I don't know how come he agreed because he was a child who didn't want to hear anything. But eventually he agreed.

**FACILITATOR:** Why didn't want to hear?

**PARTICIPANT:** Because he was afraid that he is going for circumcision and when I come back my friends will laugh at me. Because boys at the township laugh at each other; wow you from that place, you see things like that? So he also had that, even after he came back from the clinic he stayed until he recovered. He would just go outside and not go anywhere until he was better.

**FACILITATOR:**	Oh, okay. Do you think circumcision is a good or a bad thing according to you?

**PARTICIPANT**:	I think it's a good thing. From what I see it's a good thing.

**FACILITATOR:** Okay. Why do you say it's a good thing?

**PARTICIPANT:** Hey what can I say? Because it's something that should be done. It's something that all boys should do.

**FACILITATOR:** Why do you think it's something that all boys should do?

**PARTICIPANT**: Hey I don't know what to say, but I think it's a good thing for all of them to do.

**FACILITATOR:** But from what you think, remember there's no wrong or right answer. But from your own view, what do you say is a good thing about this thing, circumcision?

**PARTICIPANT:** I think it's a good thing because these days there are lot of things that are happening so if you have done it, like you know.... so what I can say? I think it's a good thing because these days we go at night, anything can happen, you don't know what will happen where you are going.

**FACILITATOR:** So to you it's a good thing because you go at night...anything can happen; something like what? Just if you can explain to me something like what?

**PARTICIPANT:** Like boys these days, there is what we call rape.

**FACILITATOR:**	Hm...

**PARTICIPANT:** So, a boy doesn't know that he's sick or what's happening he meets you in the streets and rapes you. So when you.... at least it's better you can be protected isn't?

**FACILITATOR:** So according to you circumcision is a good thing because you are protected?

**PARTICIPANT:** Yes.

**FACILITATOR:** Okay. Okay. So what is a man protecting against when circumcised?

**PARTICIPANT:** He's protecting himself against diseases that are available these days.

**FACILITATOR:** Okay. So if you can tell me, between two people who are in a relationship who do you think should talk about circumcision issues?

**PARTICIPANT:** I think it's mostly boys.

**FACILITATOR:** Why do you say a boy should be the one who deals with it?

**PARTICIPANT:** Eh...

**FACILITATOR:** Why should a boy be the one who talks about it?

**PARTICIPANT:** I don't know. I don't know but from what I see boys normally talk a lot about sex, that this and that should be done.

**FACILITATOR:** Okay. And then according to you what are the benefits of a circumcised man between two people who are in a relationship, benefits two people who are in a relationship get when a man is circumcised?

**PARTICIPANT:** Hm...

**FACILITATOR:** You've never heard of....okay, okay. And then let's say a person you dating says I have made a decision to go circumcise, what would you think of him?

**PARTICIPANT:** Hey I would ask him why he wants to circumcise after such a long time. To me it will be clear that he is with another person. That's why he is doing like this.

**FACILITATOR:** Hm Why do you think that?

**PARTICIPANT:** Obviously I would think that but I don't know why.

**FACILITATOR:** Okay so you would think that if a man tells you that he wants to circumcise that hey, maybe there is someone else?

**PARTICIPANT:** Hmm…hey…

**FACILITATOR:** When will it show?

**PARTICIPANT:** It will show...

**FACILITATOR:** Hey maybe it came from somewhere.

**PARTICIPANT:** Hey...

**FACILITATOR:** So you will ask yourself why all along. What is that time. Why would you think that way why all along?

**PARTICIPANT:** Why was he sitting and not doing it all along, and only thinking about it now? So other boys will tell him that you are now a man and you shouldn't be with one person, things like that.

**FACILITATOR:** So according to you what is a problem with a man who does circumcision later in life?

**PARTICIPANT:** I don't know.

**FACILITATOR:** Like you said all this time... what worries you that hey... why you only thinking now?

**PARTICIPANT:** Why wasn't he not thinking about it all this time, I would ask him that we've been together for 11 years so.... if he would tell me now I would ask him that why you were not thinking about it all along.

**FACILITATOR:** Hm

**PARTICIPANT:** You should have thought of it long ago.

**FACILITATOR:** So they way you look at him will change?

**PARTICIPANT:** Yes it will change.

**FACILITATOR:** What will you think?

**PARTICIPANT:** A lot of things.

**FACILITATOR:** Like what?

**PARTICIPANT:** He will be dating a lot of girls, because boys can influence each other more than us as girls. So they will influence each other.

**FACILITATOR:** Influence each other about what?

**PARTICIPANT:** You are now a man and so on.... there are lot of things.

**FACILITATOR:** I want to know from you what they are, like you say there are lot of things, I want to know those things maybe what you know that boys influence each other about.

**PARTICIPANT:** The thing is these days boys say you are now a man, you have to have lot of girls and not just stick to one person, so he can see himself as a man, that's what I normally hear from them when talking.

**FACILITATOR:** So usually men after circumcision have this thing that...

**PARTICIPANT:** Yes, that when you are a man you shouldn't stick to one person.

**FACILITATOR:** Oh, so that is why your view of him will change when he decides to go for circumcision?

**PARTICIPANT:** Hm

**FACILITATOR:** Okay. Okay. So according to you a man who decides later in life to go for circumcision, is there something wrong with that?

**PARTICIPANT:** It means there is something wrong with him to decided to do this thing this late.

**FACILITATOR:** Like what?

**PARTICIPANT:** Hey, I don't know.

**FACILITATOR:** Okay, okay, alright. So what I would like to ask you is, is there something else you would like to say about circumcision, maybe that you haven't mentioned or that you would like to say before we continue.

**PARTICIPANT:** No, nothing.
