# Supplementary material for: “To speak or not to speak”: A qualitative analysis on the attitude and willingness of women to start conversations about voluntary medical male circumcision with their partners in a peri-urban area, South Africa
Source: PLoS One. 2019 Jan 25;14(1):e0210480. doi: 10.1371/journal.pone.0210480 (PMC6347244; doi:10.1371/journal.pone.0210480)
Supplement: S1 File — (ZIP) [file pone.0210480.s003.zip › QF013_QC2.doc]

**PARTICIPANT ID:** QF013**.** Thank you for being part of this study. I would like your permission to record this interview with a recorder.

**PARTICIPANT**: Okay, I agree.

**FACILITATOR:** Okay, thank you. So please tell me where do you come from?

**PARTICIPANT:** Okay, I am from {} (participant address). I do door to door work at the clinic, where we educate and refer people to NGO's. Yes.

**FACILITATOR:** Oh. So, you go...

**PARTICIPANT:** Door to door, house to house.

**FACILITATOR:** You go house to house looking for what kind of people?

**PARTICIPANT**: No like we educate about HIV and AIDS.

**FACILITATOR:** Oh, okay.

**PARTICIPANT:** Yes, so no exactly to educate people they already know about HIV but we just remind them to go to the clinic and get tested so they know their status, also about their health for things like high blood, cancer you know.

**FACILITATOR:** Oh, so you tell them about high blood?

**PARTICIPANT:** Yes social development, others want to go to Home Affairs, so we send them there. Others to SASA and others to NGO's.

**FACILITATOR:** Okay.

**PARTICIPANT:** Things like that.

**FACILITATOR:** Oh alright. Okay so since you've mentioned that you go and tell people about those things and explain to them, can you tell me if you've ever come across something called Medical Circumcision?

**PARTICIPANT**: Yes I have and we even give them pamphlets. Because we once went for training, in fact it was a workshop…[Inaudible]. So he told us more about circumcision. So we tell people about circumcision as well.

**FACILITATOR:** Oh okay so you tell them about circumcision?

**PARTICIPANT:** Yes.

**FACILITATOR:** So if we talk about circumcision, what comes to your mind? If person says "circumcision" what comes to your mind?

**PARTICIPANT:** No, you know at first it was something that wasn't spoken about, it was only for men. But now if a person talks about it I understand that he wants to be healthy and also to reduce other diseases when he's circumcised. So I understand that he wants to have a good life. Because at first as a woman you couldn't talk to a man about it.

**FACILITATOR:** Okay. So now when they talk about circumcision, what exactly does it mean, what is done?

**PARTICIPANT:** It's something that is scary, because you think how can a person be cut while he's still alive and still be alive after that.

**FACILITATOR:** Okay. So when they talk about circumcision, they talking about being cut?

**PARTICIPANT**: Yes.

**FACILITATOR:** What do they cut?

**PARTICIPANT:** The foreskin. But we at first? We didn't understand how you can be cut alive. And that you can survive after you've been cut. Yes that's what I used to ask myself that how can you be cut in that sensitive place. How does it recover, we knew that it's done in winter and not in summer because it's hot and it will get infected but in winter it's cool there's no sun and it's not hot so you recover nicely. But now, it’s forever.

**FACILITATOR:** So you when they talk about circumcision they talking about removing the foreskin?

**PARTICIPANT**: Exactly.

**FACILITATOR:** Okay so what types of circumcision do you know of?

**PARTICIPANT:** That I know?

**FACILITATOR:** Yes.

**PARTICIPANT:** Please explain to me.

**FACILITATOR:** Okay. Where do they go for circumcision?

**PARTICIPANT**: Okay some go to the mountains.

**FACILITATOR:** Okay.

**PARTICIPANT:** Some go to the hospitals, here at the clinic like {} (name of clinic) and some go to private doctors, yes.

**FACILITATOR:** Alright, so do you know what is happens or what is done when you go for circumcision at the mountains?

**PARTICIPANT:** Eish, I think from what I see on TV going to the mountains is a very bad thing because your child goes there alive and comes back dead. And that is not right because there are no check-ups there, there are infections because the place is not clean it's just in the wild. And I am not sure what they use to cut them and I am not even sure if they sterilize it for other disease like HIV. So from my side I think that is very dangerous.

**FACILITATOR:** Okay. Okay and what about circumcision that is done at the clinic like you mentioned?

**PARTICIPANT**: From what I've learnt at the workshop which we went to, it's much better than at the mountains because you get counselling before and you also tested for diseases that you might have, like high blood and HIV and they also they you CD4 count to see whether it's fine for you to be circumcised or not, after circumcision you go for check-up to see how you are recovering, whether you recovering nicely or if there are infections.

**FACILITATOR:** Oh, okay. So like you mentioned earlier that before when you talk about circumcision people would be scared they didn't want to talk about it or they were scared to talk about it. Do you think you would tell a man and encourage him to go for circumcision, whether at home or your husband?

**PARTICIPANT:** Yes from the information I have now I can talk to people and tell them that at {} (clinic address) there's a Men's Clinic. So you can go there to find help. Like now there are people that when you talk to them they would tell you that as a woman you mustn't talk to them about such things, so if you don't want to talk I just leave you a pamphlet then you will read it later and understand it but it's a good thing to talk to them, so you can give him a pamphlet or give the person next to him if they sitting together. When you leave he will ask you to give him the pamphlet and read it when he's alone. And call that number if he's scared to come to me. Because they normally say you are not a man when you are not circumcised, so they might be scared that I will know his situation, so he can call the number on the paper.

**FACILITATOR:** Okay so like you say that you go talk to them but if a person doesn't want to talk to you, you give him the pamphlet, what other way do you think would be better for women to talk to men or tell them about circumcision?

**PARTICIPANT**: I think if, let's say you are sitting with your male sibling or cousin it would be better if you talk to him or if he's sitting with his friends you can start the topic and tell them that there's a men's clinic, which means you start with circumcision and tell them that there's a men's clinic if you are sick or have a problem with your sexual organ you can go there to find help. And even if you want to circumcise they can do it as well. In that way you started the conversation indirectly.

**FACILITATOR:** Oh, okay.

**PARTICIPANT:** Yes maybe he can understand because sometimes if you talk to him and tell him straight to go circumcise, he will feel like you undermining him. And you don't know whether he's circumcised or what but you were just talking to him. But at least if you start the conversation about telling him that there's a men's clinic and you can get help so, so, so. If you have a problem with your sexual organ you can go talk to them.

**FACILITATOR:** Okay. So what else do you think makes men not want to be told by women about circumcision?

**PARTICIPANT**: That he will think you being disrespectful, you don't see him as a man. Which means there are others you see as real men instead of him. So he will start defending himself and say you being disrespectful.

**FACILITATOR:** Okay.

**PARTICIPANT:** Which means you don't see him as a man, but he is a man. Some are just ashamed, they don't want to admit to you but deep inside he knows he wants to do it but he doesn't know how to ask you if he can do it. So that is why I say a pamphlet is an easy way of talking to people, because he won't agree to talk to you but if you leave him a pamphlet he will take it, read it and then think about it at the end he will go for circumcision.

**FACILITATOR:** Oh so you think a pamphlet is a way we can use to talk to men?

**PARTICIPANT**: Yes. And that they should get used to the fact that women are available everywhere these days. He should accept that these days you will find a woman where ever you go. And you should get permission from township councillors to host events on certain days to talk to men. And then come to the parks and halls, because some are afraid to talk in front of people so we also have people who go door to door. Maybe they can counsel and explain to him how it's done. Maybe they can go outside because there are other men who haven't been circumcised.

**FACILITATOR:** A lot.

**PARTICIPANT:** And there are a lot of diseases.

**FACILITATOR:** Yes. Okay so I understand that you mostly talking about circumcision done here at the clinic. What about the ones done at the mountains, how can people be encouraged to go there?

**PARTICIPANT:** I personally wouldn't encourage them to go to the mountains.

**FACILITATOR:** Okay.

**PARTICIPANT**: Because a lot of things have happened, in fact children have lost their lives and parents have lots their children. So I don't encourage anyone to go there. The government, community and people they all felt the pain hence they introduced such ways to save the lives and for parents to still have their children. Yes.

**FACILITATOR:** Okay so what things should you prevent or not talk about, like you say that when you go talk, what things should you avoid when talking to men about circumcision?

**PARTICIPANT:** To be honest you should be straight and not beat around the bush because that's what you came for, like I said earlier that some want you to be indirect but while you still getting to the point he will just leave you there but if you get straight to the point he might leave but after hearing what you there for.

**FACILITATOR:** Okay.

**PARTICIPANT**: So straight talk is the way to go, nothing should be hidden these days.

**FACILITATOR:** Let's says it's an older man maybe who is around his 50's do you think it should differ on how you approach them depending on their age. Which one would you use when talking to an older person?

**PARTICIPANT:** Because I am scared of talking to older men about circumcision due to respect, if there are children I just tell them that there's a {} (name of clinic) where they can take the children for circumcision. Here is pamphlet and these are the numbers you can send a call back to they will then call you to arrange when and how you can take the child. And then he will ask: Until how many years? Then I say: No mister there's no age limit as long as you haven't been circumcised you still have a chance to go there. That's how I start the conversation.

**FACILITATOR:** Okay, so what challenges do you come across when telling people about circumcision?

**PARTICIPANT**: Eish. Being beaten. Yoh. Some Zulu men tell us that: "We don't do that." And then we went to others: "That is none of your business, you shouldn't be telling me about circumcision. You are not a man you know nothing about circumcision! So there's nothing I will say to you!' So others are difficult, some wants the information, you give them, go to the clinic and they come back and say to you: We went to the clinic thank you I am fine now." Another one wouldn't even look at you when passing by he thinks: "Eish, there she is, she's going to tell me about circumcision." So he wouldn't want to even look at you or to hear anything. Another one will get the information from the one I gave the pamphlet to, then he goes to the clinic. You see?

**FACILITATOR:** So do people talk or they hear from the ones are from there?

**PARTICIPANT:** Who are from there. Yes.

**FACILITATOR:** Okay. I heard you talking about being beaten, beaten...?

**PARTICIPANT**: He would just promise to beat you and say "I will beat you my girl if you going to talk about this thing because you don't even know how it's done, you haven't even done or seen it." It's for men not women.

**FACILITATOR:** So they say it's for men?

**PARTICIPANT:** Yes.

**FACILITATOR:** Okay. So since you were talking mostly to Zulu men, how do you think other cultures would react? If maybe you were to go to a Xhosa or Sotho man, do you think it would be different?

**PARTICIPANT**: You see most people we go to are who didn't grow up in the rural areas. But grew up here in the township. But if you were to find people from the rural areas who still believe that circumcision is for men, then it becomes difficult. So others will tell you that we don't do it, in our culture we don't do that thing. But if you explain to them about diseases and the difference between life in the olden days and now, some take the decision to go for circumcision. But others still believe that the foreskin shouldn't be removed, I was born like this and will die like this, there's no disease and I won't get it. So others do understand and go to the clinic even if they don't come to the clinic they go to private doctors. Because some are ashamed because the clinic is always full so whoever will see them. But if they go to a private doctor no one will know what they there for. So they think if you all go to the clinic then it means you there for one thing even if that's not the case. Yes.

**FACILITATOR:** So what do you think makes men not want to do circumcision?

**PARTICIPANT:** Men have pride. And they don't want to listen to someone else. They want to decide for themselves even if it's not right for them. Their problem is pride, and the worst part is being ashamed and not wanting to expose themselves.

**FACILITATOR:** Oh so pride and shame is what prevents them from being circumcised?

**PARTICIPANT:** Yes.

**FACILITATOR:** Okay. So what do you think can be done for them to stop being shy?

**PARTICIPANT:** Doing campaigns once in a while, and not to have a campaign this year and again the following year only. Like in my section where I live it's in the squatter camp called Phumasilwe. I talk about it a lot and most boys are not circumcised and they are sick. So I sometimes try to talk to some of them. So I think if there can be a campaign in our area where only men can attend and encourage them to go for circumcision but if they see women or if I can go to that campaign I don't think some of them will come. But if only men can attend that campaign it would be better.

**FACILITATOR:** It would be better?

**PARTICIPANT:** Hm Now and then, meaning there should always be active campaigns. Because there are a lot of people and they are sick.

**FACILITATOR:** They are sick. I can hear that when you talk about circumcision you also mention diseases, what does circumcision prevent diseases?

**PARTICIPANT:** From what I got from the workshops it reduces them, not to say that you won't get sick at all but it minimizes the risks of getting diseases. Because the foreskin holds a lot of dirt and some people don't even bath so that dirt can turn to sores or something else and then you end up being sick. You see? But at least when you have removed it, it's better yes you can get sick but not that much. Like there are percentages.

**FACILITATOR:** Okay. So please tell me between two people who are in a relationship, who should start the topic about circumcision?

**PARTICIPANT:** People who are in a relationship?

**FACILITATOR:** Yes. A couple.

**PARTICIPANT:** It should mostly be a woman because you will get sick and you keep quite he will also not say anything because he will think he's right. Yes. So if you are a woman and your partner hasn't been circumcised you should tell him "My love you can go to the clinic they do circumcision and it doesn't take long you will be better in six weeks, you can even go for check-ups if you have a problem they check you if you have any pains and to see how the circumcision is". He wouldn't think about circumcision on his own but as a woman you should protect yourself from any diseases he might get. So you should encourage him and if he doesn't want to go maybe you can punish him and say there will be no sex until you decide to go circumcise or you will use a condom until you decide to go for circumcision.

**FACILITATOR:** So you say a female should start the topic about circumcision? How do you think he will feel if his partner is the one who starts the topic about circumcision?

**PARTICIPANT:** You see. Once you are done doing what you were doing and just relaxing, you start the topic while he's happy. It doesn't matter how he feels as long as you have told him and he heard you. Because if you don't say anything he will just continue. Can I say something?

**FACILITATOR:** Yes it's fine.

**PARTICIPANT:** I was in a relationship with another men who wasn't circumcised, and the first thing I told him was I won't be able to continue with this thing if you are not circumcised because that's what I always tell people to go circumcise. So why should I stay with you while you are not circumcised? But he wouldn't get my point, so whenever I leave the house I would leave pamphlets about male circumcision on the bed.

**FACILITATOR:** Okay.

**PARTICIPANT:** Every time when I come back from door to door I would talk about it; and tell him how many men I met and even if I didn't meet any of them I would say they agreed to go for circumcision and I helped some of them, they even call me or come to me to say thank you. Even if I didn't get anyone that day, I would make sure that everyday I talk about circumcision. So at the end we separated. I said okay thanks you don't want to cured, you will make me sick. So that's how we broke up because I used to do that all the time.

**FACILITATOR:** Okay. But through everything that you tried like telling him everyday, do you think it encouraged him or what?

**PARTICIPANT:** Him? To be honest I don't know. But you can see if a person has a guilty conscious, he felt guilty so he wanted to avoid me because I was always talking about circumcision. He hears what I am saying but he doesn't want to do it. So he thought he wouldn't be able to live with me so it's better to move on to other ones. So can you see that we broke up just because of a simple thing? It doesn't even take 2 months, not even months but only a month and a week only, but he decided to leave me because I was a nuisance. And always when I do door to door with my pamphlets I meet him because we don't leave far from each other. If he sees me coming he would go away because I would go to him and to people he's standing with and tell them that I do door to door and this is what I am talking about, which is especially for men so obviously he would move away. But you find other people who like to talk about circumcision, they ask how it's done, is it painful and I would tell them that it's not painful and they take care of you while you there, they give you breakfast, counselling, they tell you how you should check yourself and when to come for check-up and all this is for free. And if maybe you are a lot in that same area they transport you back home, so some enjoy that and they come. Some say I can't be told by a woman or people will see that i am not circumcised, so he's not thinking about his health but rather what people will say. While other people are continuing with their lives, they do it.

**FACILITATOR:** Okay, I hear you saying you tried talking to him and even bring pamphlets at home. So a woman who is not as active as you and who wants to tell her partner, what ways do you think she can use?

**PARTICIPANT:** Pamphlets, there's no other way. You just put a pamphlet in an obvious place where you partner can see it. Without saying anything, if he asks about it you just deny it and say maybe it's the kids who put it. If you don't have kids, you will say some people gave it to you and you took it without even noticing it and you put it there, you didn't even read it because you were busy. But the point is he will see it, and will realise that this person is trying to tell me something. If she doesn't want her partner to know that she wants him to do it, there are numbers to call, there is everything on the pamphlet.

**FACILITATOR:** But do you think it's easy for woman, maybe the ones you've met, do you think it's easy for them to talk to their partners abut circumcision?

**PARTICIPANT:** Talking about circumcision is not easy. For me as well it wasn't easy because I was thinking that eish... where would I start, what will I say. It's really not easy because people are different. Another one will just beat you up the moment you start talking about it and ask you where you saw it meaning you cheating on him. Go to the one who is circumcised because now you thing I am not a man since I am not circumcised. Things like that, so it's not easy.

**FACILITATOR:** Oh when women start talking about this men think that they cheating on them with men who are circumcised?

**PARTICIPANT:** Yes.

**FACILITATOR:** Okay. Do you think circumcision is beneficial to couples?

**PARTICIPANT:** Yes it's beneficial, oh they say there is what they call foreplay..[inaudible]. If he tells you to touch him there eish, it's that thing is disgusting, you touch it first before touching the penis. You see? So there are people who like sucking it, so you busy pushing the foreskin first, eish it's a real turn off, you see? So it doesn't turn you on sometimes when he has a condom on, because the condom doesn't fit properly it keeps coming out and you don't understand whether it's still there or not because it keeps moving and since there are lot of diseases I am really scared of those diseases to be honest.

**FACILITATOR:** You scared of diseases. Okay. So it helps, it helps that when you doing foreplay it's better?

**PARTICIPANT:** At least it's much better because even if you don't like doing that thing but if there's no foreskin you get that energy to say hey let me do this.

**FACILITATOR:** Okay.

**PARTICIPANT:** Whereas if there's a foreskin, eish, you see...

**FACILITATOR:** It's disgusting?

**PARTICIPANT:** Yes.

**FACILITATOR:** Okay. So if it's a man who decides that I want to go for circumcision, how would that make you feel as a woman?

**PARTICIPANT:** It would make me happy but I will a bit worried and ask myself what's happening but I wont show him. And I would also encourage him to go, that's good news. If he wants me to go with him I would go, just to give him that support so he can see that I am with him and if he wants to go alone that's also fine I will pray that all goes well for him.

**FACILITATOR:** So when you say you will ask yourself what's happening, what would be happening, what would you be thinking?

**PARTICIPANT:** I would be thinking where he go that information from, who helped him because I didn't say anything to him I was scared to tell him. I would ask myself I wonder where he got that information from, what made him take the step on his own, all along he wasn't doing anything and never talked about it, at the end of the day he wants to go circumcise.

**FACILITATOR:** So it would make you, it would make you happy or how would you feel?

**PARTICIPANT:** I would be happy if it was me.

**FACILITATOR:** You would be happy? But you would think...

**PARTICIPANT:** Where did he get it from, who helped him, I wish I could see that person and thank him, but if it was just from him then that's a good thing if he decided on his own.

**FACILITATOR:** So the question I asked you that what would make men scared to go to the clinic, I now want to know what makes men scared to go to the clinic?

**PARTICIPANT:** Usually know that it's mostly women who works at the clinic, and some think that it's painful, it's a painful thing. Some will ask you if they inject you to relieve the pain. Some say uh-hmm, they don't inject you they just cut you like that. You see? So I think that's what makes them scared. But if like your people...what do you call them?

**FACILITATOR:** Recruiters.

**PARTICIPANT:** Yes. If your recruiters can explain to them how it's done because they know more then us, isn't? That it's not painful they inject you before, you don't feel any pain, if you once had your tooth removed you would understand, they inject you before removing your tooth and then it becomes numbs, then they remove your tooth, no pain no what. And then afterwards they give you pills then you go home. So they patient with them and they explain. We try to explain to them even though we not more but we say something that will make them come to the clinic, and the information we get about circumcision. Some come and tell you that hey thank you we went to the clinic, and another one will tell you that it was full at the clinic so I was scared and went back. And then you will tell him not to be scared because they all there with their own problems. To some it's their first time like you, some are there for check-up, some are there with different problems, you all men, everyone has his own problems. So you wouldn't be scared to go to the clinic, you don't know what's wrong with them some are there due to high blood, cancer, flu and so on. So you should know that everyone has his own problems at the clinic. So you must be there for your own side and help yourself and not look at another person because you scared of a person whereas he's going on with his life.

**FACILITATOR:** So when they say they are scared of pain, what pain are they scared of, do they specify which pain they are scared of?

**PARTICIPANT:** Yes, they say it's the pain on their penis, because they say they don't inject them, they just cut them like that.

**FACILITATOR:** Oh so they scared of being cut?

**PARTICIPANT:** Yes of being cut, they scared of it.

**FACILITATOR:** Oh and then what do they say after that, do they say anything after the surgery once they've been circumcised?

**PARTICIPANT:** No.

**FACILITATOR:** They don't say anything after that, oh what they scared of is to be circumcised?

**PARTICIPANT:** Yes, they think they don't inject you instead they just cut you and put you on the bed that's what they think because they also say there's no doctor at the mountains they just cut you so how come there are pain killers at the clinic. If you explain to him that the clinic and the mountains is not the same because at the mountains you don't go back for check-up once you out you go home but at the clinic you go back for check-up, so there has to be a way for you not to feel pain at the clinic because they also know that if they cut you it's painful. And you won't sit still obviously you will jumping if they do some mistakes they do something before cutting you, they inject and then cut you, no pain.

**FACILITATOR:** So in their questions do they know about the whole procedure?

**PARTICIPANT:** No they don't know, there are those who don't know and never heard of it, you know like when you talk about bad news, it travels faster then good news. So they hear that first and then discourage each other and those who have been there tell them of what goes down. And they would say who are you to tell us. The thing is they concentrate on the bad things and they don't want to hear about the right ones, you see? That's the problem with men.

**FACILITATOR:** So when they say they are scared of women here at the clinic, what do they mean?

**PARTICIPANT:** They say women are the ones who cut them so they will disrespect them.

**FACILITATOR:** Oh, okay. So they choose not to go then...

**PARTICIPANT:** Then to circumcise, they say women can be there just for cleaning, administration but not inside at the surgery to cut them.

**FACILITATOR:** Oh so they prefer them to be there but not where the procedure is done?

**PARTICIPANT:** Yes because they say it's not right for a woman to remove a man's foreskin. I mean they still have that old mentality that if you are a woman who sleeps with a man you shouldn't touch him, you shouldn't get close to him so I am asking because the man himself if he comes back from the woman and he's still hot also, at the hospital you are assisted to give birth by a woman but they say the baby shouldn't be in a presence of a child so can you see that if you consider these things you will end up not getting any help. But if you yourself make a decision that now I am going to the clinic to circumcise, you will do this thing and finish.

**FACILITATOR:** And also say they saw too many men so they went back, what makes them go back if there are a lot of men?

**PARTICIPANT:** So what I am saying is pride, men have an empty pride that's the problem. He says if he goes there he will find other men who will see him; that wow at this age I am still like this then he goes back, the next time he goes again same thing it's a men's clinic obviously it will always be full of men who needs help like him but he runs away again.

**FACILITATOR:** Okay. It's time for us to close this part of our conversation, but before we do that is there something else you would like us to talk about that we haven't discussed, maybe something you would like clarity on?

**PARTICIPANT**: No we talked about a lot of things the only thing I am asking is that this happens now and then what I said earlier about campaigns.

**FACILITATOR:** Campaigns...

**PARTICIPANT:** That campaigns are always done, maybe for two months and so on and they should keep on repeating in the sections especially in the squatter camps because that's where things are happening, a child leaves home in the township and go to the squatter camps because there s/he has freedom, he has everything people who lives in the squatter camps stay there because they don't have places to stay so they spoil the name of where they live, because in the squatter camps that's where things are happening, these kids run away from their homes and go stay there, tomorrow they become pregnant, just because of squatter camps.

**FACILITATOR:** Because of squatter camps...

**PARTICIPANT**: Yes so they should be targeted, such issues about campaigns.

**FACILITATOR:** Okay.

**PARTICIPANT:** And there should always be a lot of pamphlets, which they should always hand out, even the recruiters when they leave they should hand out pamphlets. Pamphlets are the only way of talking to someone if he doesn't want to listen.

**FACILITATOR:** Okay. Thank you my sister. We will continue to part two.
