# Supplementary material for: “To speak or not to speak”: A qualitative analysis on the attitude and willingness of women to start conversations about voluntary medical male circumcision with their partners in a peri-urban area, South Africa
Source: PLoS One. 2019 Jan 25;14(1):e0210480. doi: 10.1371/journal.pone.0210480 (PMC6347244; doi:10.1371/journal.pone.0210480)
Supplement: S1 File — (ZIP) [file pone.0210480.s003.zip › QF029_QC2.doc]

**PARTICIPANT ID:** 029

**FACILITATOR:** Do you give me permission to record our interview with a recorder?

**PARTICIPANT**: Yes I agree that we can record our interview we a recorder.

**FACILITATOR:**	Okay, thank you. I would like to know how far do you stay from {} (name of clinic)?

**PARTICIPANT:** I don't stay far I stay here at {} (participant address).

**FACILITATOR:** Okay. So, how long does it take you to come here at {} (clinic name) from {} (participant address)?

**PARTICIPANT**: If you are using a taxi, maybe it takes about..... it depends what time you left. If you left at.... but it doesn't take more than 30 minutes, less than 30 minutes.

**FACILITATOR:** Okay. Alright. So, what do you do at the moment? Are you studying? Are you working? Or you are self employed?

**PARTICIPANT:** For now I am still looking for a job. I was working, but then the contract ended.

**FACILITATOR:** Okay.

**PARTICIPANT:** So, now I am looking for a job.

**FACILITATOR:** Okay, alright. Did you know about {} (name of clinic)?

**PARTICIPANT:** I knew about it, I first heard of it at work, because a doctor came to test us at work, and they asked me if the father of my kids is circumcised or not? And I said no he's not circumcised. They gave me another paper to give him so he can go for circumcision.

**FACILITATOR:** Okay.

**PARTICIPANT**: So when I got home I gave it to him then he came for circumcision.

**FACILITATOR:** Okay, so how did he feel when you gave it to him?

**PARTICIPANT:** The thing is me and him, I understood it because I would normally hear about it from TV, that it's easy to get viruses when you are not circumcised, so all men should go circumcise, only to find that he also heard about it. He didn't have a problem.

**FACILITATOR:** Okay. What do you know about male circumcision?

**PARTICIPANT:** Male circumcision, the way I understand it because I've heard that when you are a man who is not circumcised it's easy to get a virus if you sleep with a person who has or if you don't use a condom, so all men should take the step and go circumcise. Okay, I grew up in {} (participant address)? In {} (participant address) when I grew up they would say boys, I am not sure from what age; have to go to the mountains.

**FACILITATOR:** Okay.

**PARTICIPANT**:	So, when I came here in Johannesburg I met a Zulu man. Okay, so Zulu people didn't go to the mountains, but Xhosa people went. So this Zulu man that I met didn't understand why you go to the mountains, okay so, he would disagree with me, because in the rural boys and so on, okay so according to me if you didn't go to the mountains because that's what they talk about these days on TV that you should remove the foreskin. Then he would ask me why he should remove his foreskin, don't you understand me or what's wrong with you. Then I would say to him not that I don't understand you but that's how we grew up that it should be removed, I am not sure what happens but the way I understand it should be removed, then he said he won’t remove it because when he grew this thing never used to be removed.

**FACILITATOR:** Hm

**PARTICIPANT:** We then left it there until they spoke about it on TV and on radio that men should circumcise because it's easy for them to get a virus when they are not circumcised, that's when he had interest to take the step and be circumcised.

**FACILITATOR:** So, like you say that at the end he took the step to be circumcised, you tried encouraging him so do you think this was because he saw it on TV or was it because of how you explained it to him about the importance of circumcision?

**PARTICIPANT**:	I think it's because of the way they were talking about it on TV, because it's been long since I've telling him to go circumcise, maybe he thought I didn't feel him or .... I don't know what he was thinking.

**FACILITATOR:** When you say he thought you didn't understand him what do you mean by that?

**PARTICIPANT:** Like...

**FACILITATOR:** You were fee and not scared, am I right? Free.

**PARTICIPANT:** Okay. He thought I didn't feel him when we having sex, the thing is he has the foreskin, he didn't remove it, so I don't know what he was thinking when I told him to go circumcise, maybe he thought I am cheating on him and found that it's nicer with other men, I don't know what he was thinking because when I tell him to go circumcised he would say oh so it means you don't feel me.

**FACILITATOR:** So, when you tried explaining to him that it's not that you don't feel him, what reasons did you give him to go circumcise so that he doesn't think that you don't feel him, was there a way you explained to him so he can understand why he should go for circumcision?

**PARTICIPANT**:	I would tell him that I grew up in {} (participant address), I also didn't know why they go for circumcision, but I heard from the clinics and hospitals that they should circumcise, so I would tell him that I grew up knowing that boys have to circumcise. Okay, we have a boy alright... So it means this whole thing started with my boy that I want to take him to the clinic for circumcision and he said according to their tradition they don't do that because this child is Zulu so he will follow their tradition. Then I told him that at home boys are circumcised, and you should also go for circumcision because all men do it. Then he said to me; oh so does it mean I should go circumcise because you don't feel me? Or you slept with other men and felt the difference.

**FACILITATOR:** Okay. Explain to me about circumcision that is done in Eastern Cape and then you also encouraged him to go circumcise at the clinic, what types of circumcision do you know of?

**PARTICIPANT:** In Eastern Cape they go to the mountains, I left {} (participant address) when I was still young, maybe around 19 or 20 years then I came this side.

**FACILITATOR:** Okay. So between this type of circumcision that is done in {} (participant address) that you mentioned and encouraging him to come here at the clinic, what's the difference between those two types of circumcision.

**PARTICIPANT**: In Eastern Cape they go to the mountains…

**FACILITATOR:** Uh-hmm.

**PARTICIPANT:** Here in Johannesburg it's easy because you go to the clinic, you make a booking and they give you the date when you can come. In Eastern Cape it's difficult because it involves a lot of risks, hence I don't like Eastern Cape, I wish my child can come here at the clinic or the hospital.

**FACILITATOR:** Okay, what risks are you referring to that you say are in Eastern Cape?

**PARTICIPANT**:	I am not sure really what happens in Eastern Cape because some people don't come back from the mountains. I don't know why they don't come back. That is why I say it's risky because I don't know what causes that or it's because there are no doctors there, I don't know who cut them, that's what I don't understand.

**FACILITATOR:** Okay, and then did your partner come to do circumcision at the end?

**PARTICIPANT:** Hm

**FACILITATOR:** Okay, since he eventually came for circumcision and you were trying to encourage him to circumcise and he will ask you if you don't feel him or what or if you were cheating, how did you feel when he said that?

**PARTICIPANT**: Yoh. I would be disappointed. That's why I ended up not saying anything, until he was the one to take the step and come here. I remember the first day, he didn't tell me when he was going for booking, that he has done it, he only told me when he was from the clinic, he called me while I was at work that he went for circumcision and they told him not to have sex with me for six weeks. I was very happy that finally he realised how important this thing is.

**FACILITATOR:** Do you think that maybe when you encouraged him to go for circumcision while he didn't want to do it, because he thought you heard something from other people, do you think it was because of the way you were telling him, what would you say to him, when encouraging him to go for circumcision?

**PARTICIPANT:** I think maybe or I didn't give him the right reasons because I also didn't have much information as to why they circumcise, and how you get this virus? How it stays there? Where?

**FACILITATOR:** So, what would you say to encourage him, when motivating him to circumcise?

**PARTICIPANT**: I would encourage him to go for circumcision because I saw from how I grew up in Eastern Cape that men circumcise, like I had a brother but he died last year.

**FACILITATOR:** Hm

**PARTICIPANT:** My brother used to say sill things like ‘you are dating a Zulu man', he's not a man, so I didn't want him to be classified as a boy.

**FACILITATOR:** So, like he would explain to you that it's not my tradition to circumcise, we as Zulu men we don't circumcise, and then what would you say to him?

**PARTICIPANT**: I would be disappointed and end up not saying anything and see that it's better for me to keep quiet, because I didn't want him to feel otherwise.

**FACILITATOR:** Since you have child which is a boy and you would like for him to circumcise, and your partner came for circumcision, do you think it will still be difficult to bring your child for circumcision or do you think one day he will agree for him to circumcise?

**PARTICIPANT:** No,I don't think so because he also wants him to be circumcised, but when I told him that my child has to go for circumcision he said there must be someone to take care of him, since he knows how it was, pains and staff, so there should be someone who will be with him full time.

**FACILITATOR:** So now you think he doesn't any problem for the child to be circumcised?

**PARTICIPANT:** No, he doesn't have a problem.

**FACILITATOR:** Okay, how old is your child?

**PARTICIPANT**: He is 5 years old.

**FACILITATOR:** Oh, okay. Alright. So, if maybe your partner was the one who firstly told you that he wishes to be circumcised, what would you have said?

**PARTICIPANT:** I didn't have a problem at first.

**FACILITATOR:** You wouldn't have felt the way he felt with you, that maybe you heard it from other people that is why you telling me about circumcision because according to your culture you don't circumcise, wouldn't have thought otherwise if he was the one who started the topic?

**PARTICIPANT**: No, I wouldn't have.

**FACILITATOR:** Okay. So when you started to encourage him about circumcision, by explaining to him that while growing up in Eastern Cape people used to circumcised but he responded by saying that doesn't matter, don't you think there was another way you could have used to encourage him to go circumcise without telling him about how you do things in Eastern Cape, maybe another way that would have made him understand better the importance of circumcision?

**PARTICIPANT:** There was another way but I don't know what happened, I think I should have told him that it's easy to get diseases when you are not circumcised, so it's better to circumcise.

**FACILITATOR:** So why didn't you approach him in that manner at first, by explaining to him that it's important to circumcise because you don't easily get diseases, but instead you explained to him about your culture in Eastern Cape which is not his?

**PARTICIPANT**: Because I didn't have that information then.

**FACILITATOR:** Okay. So where did you get that information that when you are circumcised in minimizes the risks of getting diseases?

**PARTICIPANT:** I got the information from the radio and TV when they advertised, saying men should circumcise.

**FACILITATOR:** Okay. After you got the information on radio and on TV did you explain to him again or he just decided by himself to go and circumcised?

**PARTICIPANT**: I explained to him again but he had already decided that he will go circumcise.

**FACILITATOR:** So, you think what made him take this decision to go circumcise, is because of the information he got from TV and radio?

**PARTICIPANT:** Hm, I think so.

**FACILITATOR:** Okay, what do you think you could have avoided saying to him to motivate him to go circumcise?

**PARTICIPANT**:	I think since I told him about how we do things at home, I shouldn't have told him that, because that's not his culture.

**FACILITATOR:** So you think that made him feel...

**PARTICIPANT:** Maybe it didn't sit well with him.

**FACILITATOR:** Okay. Maybe what could you have prevented to say when encouraging him to go for circumcision besides telling him about your culture? Is there something else you could have tried to prevent when encouraging to go circumcise?

**PARTICIPANT:** No, without me not talking about my culture.

**FACILITATOR:** Okay, since you tried speaking to him about circumcision, encouraging him even thought it was difficult because he felt you heard it from other people, or that it's not his culture, how difficult was that on you?

**PARTICIPANT:** Yoh. It was difficult because there was a time when didn't trust each other.

**FACILITATOR:** What caused that? Was he thinking you doing some things or...

**PARTICIPANT:** Yes, he thought I was doing some things maybe I am cheating on him with someone who is circumcised. Yes.

**FACILITATOR:** Okay. So, how is the situation now at home, is there still mistrust or did you do something to build that trust again so he doesn't think that you telling him about circumcision because you cheating on him?

**PARTICIPANT:** No we fine now.

**FACILITATOR:** Hm.

**PARTICIPANT:** We fine now. Because I told him that it's not because I am doing other things, but it's what I hear from other people.

**FACILITATOR:** What would you say to your friend who also wants to tell her partner to go circumcise? Maybe what tips would you give her when trying to speak to her partner encouraging him to circumcise? Things that make it easier for a man to do circumcision?

**PARTICIPANT:** I would encourage her to tell her partner to go circumcise because these days there are lot of diseases so to prevent getting them he should go circumcise. So, that's how I would encourage her.

**FACILITATOR:** Which diseases are you talking about?

**PARTICIPANT:** HIV, STD and STI.

**FACILITATOR:** Do you know of different types of STI?

**PARTICIPANT:** I would the drop is the famous one. I don't about others.

**FACILITATOR:** How do you think male circumcision helps, you've mentioned that it minimizes the risk of getting HIV and sexually transmitted diseases. What else can you mention which you think is a right thing for a man to circumcise?

**PARTICIPANT:** I don't know besides diseases. I don't know.

**FACILITATOR:** Okay. Since your partner ended up going for circumcision, do you see any difference since he's been circumcised?

**PARTICIPANT:** It's the same as before. It's just fine.

**FACILITATOR:** When you say it's just fine what do you mean?

**PARTICIPANT:** Ehh…

**FACILITATOR:** Remember when you first started talking to him, he thought maybe you were cheating on him with a circumcised man as if there's a difference between a circumcised and uncircumcised man.

**PARTICIPANT:** No I don't see the difference. I don't know because I haven't been with.... but I don't see the difference.

**FACILITATOR:** When speaking to you, did he share certain things with you?

**PARTICIPANT:** No, he didn’t share anything.

**PARTICIPANT:** Okay. So, between people who are in a relationship who do you think should start this topic of circumcision, about the importance of a man to circumcise?

**PARTICIPANT:** I think it's me, because I have to encourage him.

**FACILITATOR:** Since you tried to encourage him as a woman and then your partner thought maybe you were cheating on him hence you also want him to go circumcise, do you think it's easier for a woman to start the topic when you are in a relationship?

**PARTICIPANT:** It's not easy but at the end he also saw that I was trying to encourage him. But it's not easy.

**FACILITATOR:** Okay. If a man starts the topic, since you mentioned that you as a woman should start the topic when you are in a relationship, so what's the difference when a woman starts it?

**PARTICIPANT:** If he starts the topic it's better because he knows that you also raised your views. There's an understanding.

**FACILITATOR:** When you do what?

**PARTICIPANT:** When you also raise your views... if he tells you that he wants to circumcise it becomes easier for him if you also say how you feel that he should go circumcise.

**FACILITATOR:** Okay. So if a man tells you that he wants to go circumcise, what views would you have?

**PARTICIPANT:** I would be happy that atleast he took the stand to go circumcise because our lives are not safe these days.

**FACILITATOR:** Okay. Alright. What important things would you like to say that we didn't mention in our interview?

**PARTICIPANT:** I think all men should circumcise. An uncircumcised man should take the step to go circumcise and minimize the diseases that are available these days.

**FACILITATOR:** So which easy ways can women use to encourage men to go circumcise, it can be their partner or brother? What can they do to encourage men to go circumcise?

**PARTICIPANT:** They can encourage them by talking to them and explain to them that they should go for circumcision because they also see that life is not safe these days. So after they have been circumcised, atleast we can try to minimize the disease that are available these days, try to finish them.

**FACILITATOR:** Okay. Is there something else you would like to say?

**PARTICIPANT:** No I think I am fine.

**FACILITATOR:** Okay. Okay, now we are going to move to our second activity, okay?

**PARTICIPANT:** Hm.
